# Deciphering the Genetic Landscape: Insights Into the Genomic Signatures of Changle Goose

**DOI:** 10.1111/eva.13768

**Published:** 2024-08-22

**Authors:** Hao Chen, Yan Wu, Yihao Zhu, Keyi Luo, Sumei Zheng, Hongbo Tang, Rui Xuan, Yuxuan Huang, Jiawei Li, Rui Xiong, Xinyan Fang, Lei Wang, Yujie Gong, Junjie Miao, Jing Zhou, Hongli Tan, Yanan Wang, Liping Wu, Jing Ouyang, Min Huang, Xueming Yan

**Affiliations:** ^1^ College of Life Sciences Jiangxi Science and Technology Normal University Nanchang China; ^2^ College of Animal Sciences & Technology Zhejiang A&F University Hangzhou China

**Keywords:** Changle goose, conservation genetics, genetic diversity, genomic analysis, meat quality, reproduction

## Abstract

The Changle goose (CLG), a Chinese indigenous breed, is celebrated for its adaptability, rapid growth, and premium meat quality. Despite its agricultural value, the exploration of its genomic attributes has been scant. Our study entailed whole‐genome resequencing of 303 geese across CLG and five other Chinese breeds, revealing distinct genetic diversity metrics. We discovered significant migration events from Xingguo gray goose to CLG and minor gene flow between them. We identified genomic regions through selective sweep analysis, correlating with CLG's unique traits. An elevated inbreeding coefficient in CLG, alongside reduced heterozygosity and rare single nucleotide polymorphisms (R_SNPs_), suggests a narrowed genetic diversity. Genomic regions related to reproduction, meat quality, and growth were identified, with the *GATA3* gene showing strong selection signals for meat quality. A non‐synonymous mutation in the *Sloc2a1* gene, which is associated with reproductive traits in the CLG, exhibited significant differences in allelic frequency. The roles of *CD82*, *CDH8*, and *PRKAB1* in growth and development, alongside *FABP4*, *FAF1*, *ESR1*, and *AKAP12* in reproduction, were highlighted. Additionally, *Cdkal1* and *Mfsd14a* may influence meat quality. This comprehensive genetic analysis underpins the unique genetic makeup of CLG, providing a basis for its conservation and informed breeding strategies.

## 1 Introduction

Chinese domestic geese predominantly descend from the wild swan goose (*Anser cygnoides*), with the exception of the Yili goose, which has its origins in the greylag goose (*Anser anser*) (Li et al. 2011). The domestication of geese has been documented for approximately 7000 years, with the specific domestication of Chinese geese initiated around 3499 years ago (Wen et al. 2023). This domestication, influenced by environmental pressures and artificial selection, has led to pronounced phenotypic and genomic alterations, manifesting in traits such as body size, feather morphology, and egg yield. Changle goose (CLG), an emblematic indigenous breed from Changle District, Fuzhou, Fujian Province, was introduced from northern China to its current southern habitat approximately 500 years ago, undergoing extensive breeding adaptations for coastal foraging. It boasts a distinctive appearance characterized by gray plumage and an upright posture. Renowned for its robust adaptability, rapid growth, and superior meat yield, the CLG also excels in liver fattening after intensive feeding. However, its pronounced broodiness and suboptimal egg production constrain the expansion of goose husbandry.

Research endeavors have delved into the genetic foundations of geese. Gao et al. (2022) identified 42 SNPs linked to reproduction using whole‐genome association analysis. In a subsequent study, 26 SNPs were pinpointed, associated with egg quality and reproductive performance (Gao et al. 2021). Zheng et al. (2023). elucidated the genes indicative of meat quality, growth, reproductive, and immunity traits in the Lingxian white goose. Ouyang et al. (2022) and Peng et al. (2018) analyzed the genetic diversity between domesticated goose breeds and their wild progenitors, revealing that domestic geese exhibit greater genetic diversity than their wild counterparts. While several studies have shed light on the domestication and genetic characteristics of geese at the genomic level, the knowledge pertaining to the genetic intricacies of CLG remains sparse.

Recognizing the significance of the CLG in the realm of poultry, this study aims to explore its genetic landscape. Despite its renowned meat quality and growth rate, the low egg production of CLG poses a challenge to the expansion of its population. Modern genomics and sequencing technologies offer a platform for in‐depth genetic exploration. However, no studies have yet harnessed whole‐genome sequencing (WGS) data to probe the genetic diversity, selection pressures, and population structure of CLG.

This research aims to fill this knowledge gap. By employing WGS data from 60 CLG and comparing it with data from five other Chinese local goose breeds (encompassing a total of 243 samples), we seek to elucidate the unique genetic makeup of CLG. Our objective is to highlight the importance of genetic resource conservation and to identify genes that have undergone positive selection during domestication and breeding. Such insights will be instrumental for future genetic and breeding studies, not only for CLG but also for other goose breeds.

## 2 Materials and Methods

### 2.1 Experimental Animals

In this study, we examined a cohort of 367 individuals spanning six Chinese indigenous goose breeds and two wild goose breeds (Table S1). The domesticated breeds comprised CLG (*n* = 60), Fengcheng gray goose (FCG; *n* = 50), Zhedong white goose (ZDW; *n* = 46), Minbei white goose (MBW; *n* = 25), Magang goose (MGG; *n* = 62), and Xingguo gray goose (XGG; *n* = 60). Notably, both FCG and XGG are indigenous to Jiangxi Province, whereas CLG is native to the Changle District of Fuzhou City in Fujian Province. ZDW, MBW, and MGG have their origins in Zhejiang, Fujian, and Guangdong Province, respectively. The wild species assessed were the swan goose (ACy; *n* = 59) and greylag goose (AAn; *n* = 5), both sourced from Poyang Lake in Jiangxi Province. To ensure genetic diversity in our analysis, all selected individuals were unrelated. Venous blood samples were drawn from beneath the wings of each goose. All experimental protocols were sanctioned by the Animal Ethics Committee of Jiangxi Science and Technology Normal University (3601020137931) and adhered rigorously to the Guidelines of the China Animal Welfare Association.

### 2.2 Whole‐Genome Resequencing

We extracted genomic DNA from the whole blood of the 367 geese using the standard phenol‐chloroform method. The integrity and purity of the DNA were verified via agarose gel electrophoresis and by ensuring an A260/280 ratio between 1.8 and 2.0. DNA samples meeting these criteria were processed for library construction (Paired‐end, 2 × 150 bp). Subsequently, whole‐genome resequencing was conducted on the Illumina NovaSeq 6000 platform at Novogene (Beijing, China). The average sequencing depth achieved for each individual goose was approximately 10‐fold.

### 2.3 SNP Calling and Annotation

Raw data were first subjected to quality control by removing adapter sequences and discarding reads with a quality score below 30 (*Q* < 30). The reference genome was indexed utilizing the “index” function of BWA v0.7.17 (Li and Durbin 2009). Subsequently, the clean data from all 367 samples were aligned to the high‐resolution Xingguo Gray goose reference genome (accession: GWHBAAW00000000) at the chromosome level (Ouyang et al. 2022) using the indexed reference file, producing alignment files in SAM format. These files were converted to BAM format using SAMtools v1.10 (Li et al. 2009). PCR duplicates were eliminated with the “rmdup” function, followed by BAM file sorting via the “sort” function. Variant calling was executed using the HaplotypeCaller tool in Sentieon (Kendig et al. 2019). The detected variants were further filtered using the VariantFiltration command of GATK v4.1 (McKenna et al. 2010) with the following parameter settings: “QD < 2.0 || FS > 60.0 || MQ < 40.0 || MQRankSum < −12.5 || ReadPosRankSum < −8.0 || SOR > 3.0” for SNPs, and “QD < 2.0 || FS > 200.0 || ReadPosRankSum < −20” for InDels. Only variants with a minor allele frequency (MAF) above 0.1 and a genotype call rate exceeding 0.9 were retained, as filtered by Plink v1.9 (Chang et al. 2015). The final set of variants was annotated using SnpEff v5.0 (Cingolani et al. 2012).

### 2.4 Structural Variations Detection and Analysis

Structural variations (SVs), excluding insertions, were detected using Lumpy v0.2.13 (Layer et al. 2014) with the default parameters specified as “lumpyexpress ‐B ‐S ‐D”. Following detection, genotyping of these SVs was performed with SVTyper v0.0.4 (Chiang et al. 2015). Variants shorter than 50 bp were subsequently filtered using the “filter” command in SURVIVOR v1.0.6 (Jeffares et al. 2017). The “merge” command in SURVIVOR, set with parameters “merge name 250 1 1 1 0 50” (Hamala et al. 2021), was employed to consolidate the detected SVs. This consolidation considers the distance between breakpoints, SV type, and strands. Post‐merging, deletions (DEL), and duplications (DUP) exceeding 100 kb, as well as inversions below 1 kb, were excluded. For this analysis, the breakpoint distance threshold was established at 250 bp. To validate the accuracy of the identified SVs, a subset of 20 random variants was selected and verified using the Integrative Genomics Viewer (IGV) tool.

### 2.5 Structural Variations Analysis

Different types of SVs were quantified, and the proportion of each SV type across various breeds was determined. Utilizing the binary SV data, a distance matrix was constructed with the “plink ‐‐distance‐matrix” command. The neighbor‐joining (NJ) tree was subsequently generated using the “neighbor” command in Phylip v3.69 (Retief 2000), with visualization facilitated by iTOL (https://itol.embl.de/). The same binary SV data was employed to establish a kinship matrix via the GCTA (Yang et al. 2011) software. Principal component analysis (PCA) was executed using the “‐‐pca” command in Plink and the first two principal components were visualized using R.

### 2.6 Genomic Diversity Analysis

Genetic diversity is indicative of an organism's adaptability to environmental shifts. In this context, we assessed population genetic diversity through 10 specific metrics. SNPs with a minor allele frequency (MAF) exceeding 0.2 within each breed were categorized as common variants (Csnp), whereas those with an MAF between 0 and 0.05 were deemed rare variants (Rsnp). The heterozygosity ratio is denoted by Pn. Utilizing Plink v1.9, we determined the heterozygosity ratio, expected heterozygosity (He), and observed heterozygosity (Ho) for each breed via the “‐‐freq,” “‐‐het,” and “‐‐hardy” commands, respectively. Inbreeding coefficients (F), derived from genomic heterozygosity, along with genetic distances within breeds (DST), and accumulated runs of homozygosity (F_ROH_), were computed using Plink v1.9. The nucleotide polymorphism (π), representing population polymorphism levels, was estimated genome‐wide using vcftools v0.1.16 (Danecek et al. 2011) employing a 20 kb sliding window with a 10 kb step size. R was utilized for the visualization of these genetic diversity metrics.

### 2.7 Population Structure Analysis

Following data filtration via the Plink software, employing the “‐‐maf 0.01” and “‐‐geno 0.1” parameters, a dataset comprising 8,060,412 SNPs was retained. This dataset facilitated the estimation of inter‐individual genetic relationships using Plink. The “‐‐distance‐matrix” command in Plink enabled the construction of a genetic distance matrix. Thereafter, a neighbor‐joining (NJ) tree was constructed using Phylip v3.69 and visualized via the iTOL. For PCA, the GCTA software was employed to generate a genetic relationship matrix (GRM) based on the genetic variability of all samples. The autosomal principal components were computed using the “‐‐pca” command, with visualization of the initial four components achieved through R. During the population structure assessment, variant sites underwent filtration with the “plink ‐‐indep‐pairwise 50 10 0.2” parameters. The filtered independent variants subsequently informed the estimation of sample population structure, executed using ADMIXTURE v1.3.0 (Alexander, Novembre, and Lange 2009) with standard parameters and encompassing 10,000 iterations. To detect gene flow between CLG and other Chinese local geese, we conducted a migration event analysis using Treemix v1.3 software (Pickrell and Pritchard 2012) with ACy as the root. Additionally, we performed a D‐test using Admixtools (Patterson et al. 2012) to further validate our findings.

### 2.8 Detection of Selective Signatures

To detect recent selection signals within the CLG genome, we employed a composite likelihood ratio test (CLR) to delineate regions exhibiting positive selection. Utilizing SweeD v4.0.0 software (Pavlidis et al. 2013), each autosome was segmented into 1 kb grids for this estimation. Regions falling within the top 0.1% were designated as candidate regions.

Furthermore, three methods were employed in this study to detect selective sweep regions within the CLG genome. Leveraging multiple detection techniques enhances accuracy by minimizing false positives and facilitating methodological cross‐validation. Fst values between CLG and five other breeds, as well as SNP‐level frequency differentials between groups, were computed using Plink. These values were averaged over 10 kb windows. Cross‐population composite likelihood ratio tests (XP‐CLR) between CLG and five other local Chinese goose breeds were executed using selscan v1.3.0 software (Szpiech and Hernandez 2014). Genes located in the overlapping regions, resulting from the top 1% Fst and XP‐CLR windows in comparison between CLG and other local breeds, were identified as candidate genes. Functional annotation and enrichment analysis of these candidate genes were conducted using Metascape (Zhou et al. 2019). Haplotype analyses of target regions incorporated two wild populations and 303 samples from six Chinese indigenous geese breeds. Haplotypes for the specified regions were generated using the fastPhase function within Beagle software (Browning and Browning 2007), and they were then grouped by population and visualized using the “pheatmap” package in R. SIFT (http://sift‐dna.org) was used to predict the effect of amino acid substitution on protein function. The Robetta tool (http://robetta.bakerlab.org/) was used to predict the three‐dimensional structure of proteins.

## 3 Results

### 3.1 Identification of SNPs and SVs

**FIGURE 1:**
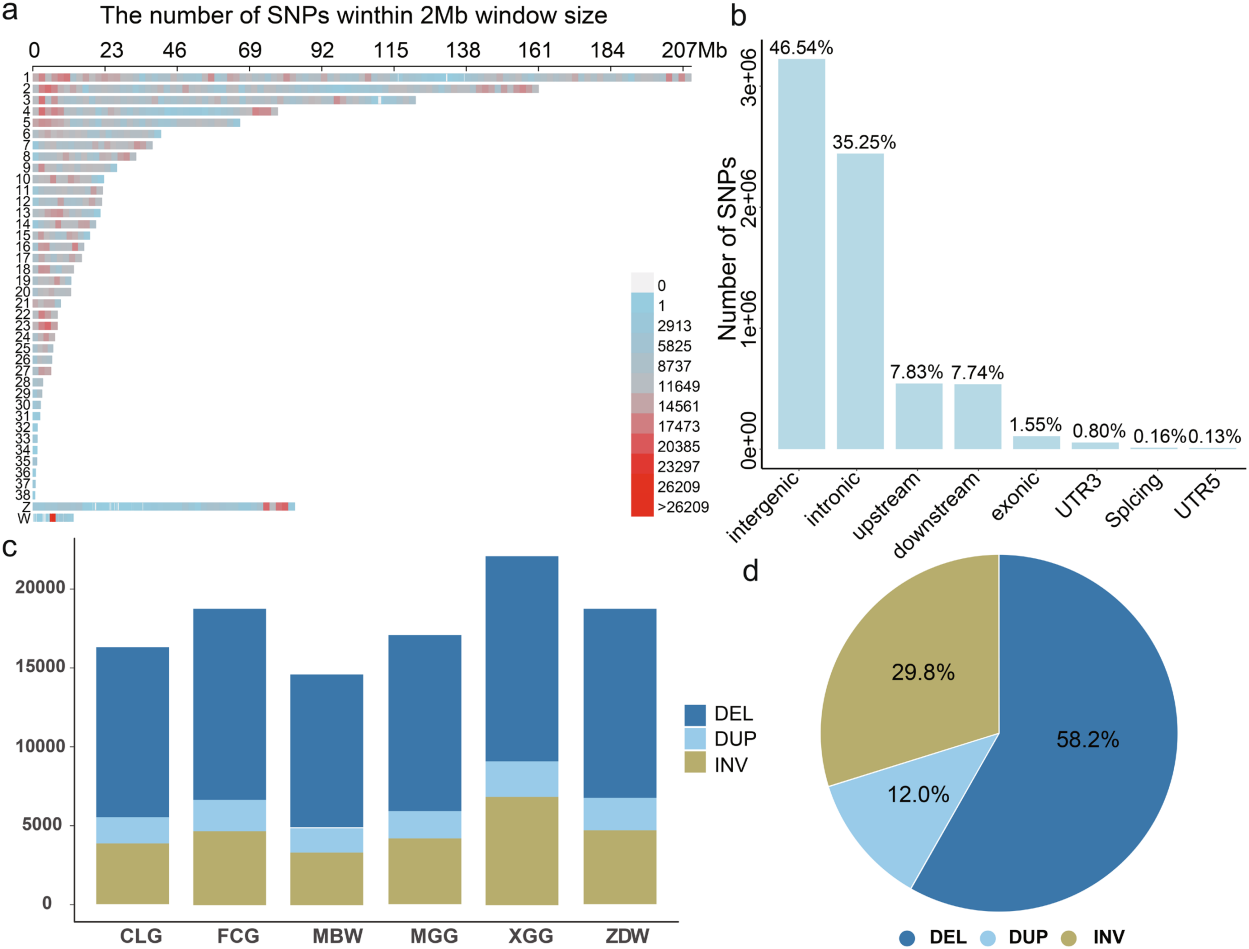
Genome‐wide distribution and annotation of SNPs and SVs in the Changle goose. (a) Depicts SNP density across the genome, calculated at 2 Mb intervals. (b) Illustrates the functional categorization of SNPs as determined by SnpEff, highlighting the distribution within various genomic contexts. (c) Details the distribution of SV types—deletions (DEL), duplications (DUP), and inversions (INV)—across six different goose breeds. (d) Presents the proportional representation of SVs on autosomal chromosomes within a cohort of 303 geese. Refer to Table 1 for breed abbreviations.

In this study, we conducted whole‐genome resequencing on 303 Chinese indigenous geese, which included 60 CLG, 50 Fengcheng gray geese (FCG), 25 Minbei white geese (MBW), 62 Magang geese (MGG), 60 Xingguo gray geese (XGG), and 46 Zhedong white geese (ZDW), achieving an average sequencing depth of 10× (Table S1). Utilizing conventional variant detection methodologies, we identified 10,425,683 variants from 367 samples (including 59 ACy and 5 AAn). After filtering out variants with an MAF of less than 0.01 and a call rate below 0.1, we retained 8,060,412 SNPs for further analysis. We initially isolated the SNP data for CLG, obtaining a total of 5,801,560 SNPs with an MAF greater than 0.05 and a call rate above 90% (Table S2). The genome‐wide SNP density was highest on Chromosome 23 and lowest on Chromosome 34, as visualized in 2 Mb genomic blocks (Figure 1a). Annotation of the SNPs revealed the highest enrichment in intergenic regions (46.54%), succeeded by intronic regions (35.25%), upstream regions (7.83%), downstream regions (7.74%), exons (1.55%), untranslated regions (0.93%), and splice site regions (0.13%) (Figure 1b). Pertaining to protein‐coding genes, we identified 33,558 missense variants, 70,562 synonymous variants, 51 initiator‐codon variants, 576 stop‐loss variants, 98 start‐loss variants, and 1194 stop‐gained variants (Table S3).

Employing the Lumpy software, we detected 69,982 structural variations (SVs) in 303 geese from six Chinese indigenous breeds. Rigorous length‐based filters were implemented to exclude SVs smaller than 50 bp or exceeding 100 kb, comprising 21,564 deletions (DEL), 5107 tandem duplications (DUP), and 13,466 inversions (INV). The XGG population exhibited the highest incidence of SVs, while the MBW showed the lowest (Figure 1c). Deletions were the most common, forming 58.2% (19,245) of the variations, inversions represented 29.8% (9854), and duplications comprised 12.0% (3950) (Figure 1d). In the CLG‐specific SV analysis, we identified 16,340 SVs, with 10,827 DEL, 1634 DUP, and 3870 INV (Figure 1c). Deletions emerged as the most prevalent SV type across all breeds examined. The observed distribution of SV types was consistent with the comprehensive dataset (Figure 1c, d). It is of particular significance that, aside from the MBW—which may be attributed to its limited sample size—the CLG exhibited a reduced diversity of SV types in comparison to other indigenous Chinese goose breeds. This observation implies that selective breeding practices may have played a role in shaping genomic diversity.

### 3.2 Genetic Diversity and Population Structure of Changle Goose

To thoroughly evaluate genomic variation within the CLG, we utilized 10 genetic diversity metrics, including rare SNPs (R_SNP_), nucleotide diversity (*π*), and heterozygosity rate (Het), as detailed in Table 1. Among the variants identified in CLG, 995,155 were categorized as rare, with a minor allele frequency (MAF) of less than 0.05, which was the smallest number identified among the Chinese indigenous breeds. Linkage disequilibrium (LD) analysis revealed the most extensive LD decay in CLG, suggestive of lower genetic diversity, a pattern followed in descending order by ZDW, MBW, MGG, XGG, and FCG (Figure S1). The nucleotide diversity of CLG was the lowest among the breeds, with a π value of 0.18, aligning with the observed LD decay patterns (Table 1; Figure S1). The inbreeding coefficient of CLG was significantly higher at *F* = 0.16 ± 0.04 compared to the other breeds, indicative of a greater degree of homozygosity (Figure S2a; Table 1). The expected heterozygosity (He), observed heterozygosity (Ho), intraspecific genetic distance (DST), proportion of polymorphic markers (Pn), and heterozygosity rate (Het) in CLG were 0.22, 0.24, 0.20, 0.85, and 0.36 ± 0.02, respectively, all of which were lower than those observed in the other five breeds (Figure S2b,c and Table 1). These metrics collectively underscore the lower genetic diversity within the CLG population.

**TABLE 1:** Comparative genetic diversity metrics among six goose breeds.

Breed	Num	C_SNP_	R_SNP_	Pn	He	Ho	*F*	DST	*F*_ROH_ (%)	*π* (%)	Het
CLG	60	3,204,699	995,155	0.85	0.24	0.22	0.16 ± 0.04	0.20 ± 0.08	8.01 ± 2.91	0.18	0.36 ± 0.02
FCG	50	3,332,392	1,254,540	0.92	0.25	0.24	0.09 ± 0.04	0.22 ± 0.04	8.22 ± 2.81	0.20	0.39 ± 0.02
MBW	25	3,047,446	1,242,899	0.85	0.24	0.23	0.14 ± 0.02	0.21 ± 0.02	8.02 ± 1.19	0.19	0.37 ± 0.01
MGG	62	3,282,717	1,160,171	0.88	0.24	0.22	0.14 ± 0.04	0.21 ± 0.03	7.85 ± 1.94	0.19	0.37 ± 0.02
XGG	60	3,337,484	1,042,829	0.91	0.25	0.24	0.08 ± 0.05	0.21 ± 0.06	8.71 ± 2.18	0.19	0.40 ± 0.02
ZDW	46	3,259,378	1,335,760	0.88	0.24	0.24	0.11 ± 0.03	0.21 ± 0.01	9.97 ± 1.37	0.19	0.38 ± 0.01

*Note:* Num represents the sample size of the breed, C_SNP_ denotes the number of common SNPs (MAF > 0.2), and R_SNP_ denotes rare SNPs (MAF < 0.05). Pn represents the proportion of polymorphic markers. He and Ho stand for expected and observed heterozygosity, respectively. *F* indicates the inbreeding coefficient, DST refers to the intraspecific genetic distance, and *F*_ROH_ is the proportion of the genome that is homozygous. π signifies the overall nucleotide diversity within the breed, and Het denotes the heterozygosity rate.

Abbreviations: CLG, Changle goose; FCG, Fengcheng gray goose; MBW, Minbei white goose; MGG, Magang goose; XGG, Xingguo gray goose and ZDW, Zhedong white goose.

**FIGURE 2:**
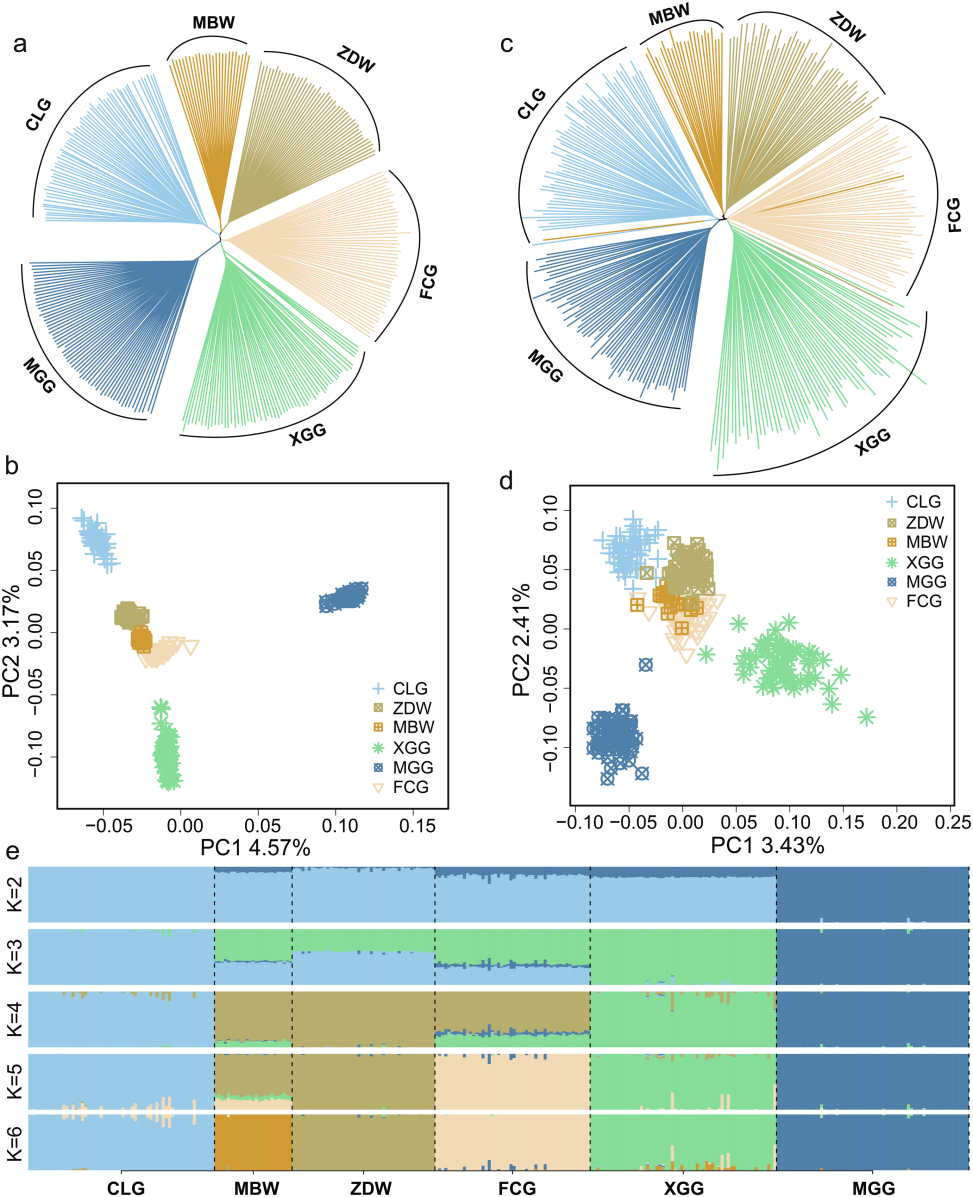
Genetic structure and phylogenetic relationships of six goose breeds based on autosomal variants. (a) The neighbor‐joining (NJ) phylogenetic tree, derived from SNP data of 303 geese. (b) Principal component analysis (PCA) results, accounting for 4.57% of the variance in PC1 and 3.17% in PC2. (c) The NJ phylogenetic tree constructed from the SV dataset for 303 geese. (d) PCA findings based on the SV dataset for 303 geese, which explain 3.43% of the variance in PC1 and 2.41% in PC2. (e) The genetic composition of goose breeds, as elucidated by ADMIXTURE analysis, spanning *K* values from 2 to 6.

To elucidate the phylogenetic relationships and genetic architecture of the CLG, neighbor‐joining (NJ) phylogenetic tree and principal component analysis (PCA) were systematically performed on our comprehensive datasets of single nucleotide polymorphisms (SNPs) and structural variations (SVs). The results revealed a concordant phylogenetic signal across both methods (Figure 2a,c,d). NJ tree analysis demonstrated breed‐specific clusters, indicating that CLG has a closer genetic relationship with MBW, ZDW, and FCG (Figure 2a,c). PCA clearly differentiated CLG from MGG, underscoring a pronounced genetic divergence likely influenced by geographic isolation (Figure 2b,d). Admixture analysis supported these findings, showing a clear separation between CLG and MGG at *K* = 2, with increasingly distinct genetic partitions for other breeds evident up to *K* = 6, in the following sequence: XGG at *K* = 3, ZDW at *K* = 4, FCG at *K* = 5, and MBW at *K* = 6, thus confirming the insights from the NJ tree and PCA (Figure 2e).

Treemix analysis revealed significant migration events from XGG to CLG (migration weight = 0.31) as well as to MBW (migration weight = 0.29) (Figure S3a). Additionally, the D‐test indicated significant gene flow between ZDW and MBW (|*z*‐score| = 8.18) and minor gene flow between CLG and XGG (|*z*‐score| = 1.19) (Figure S3b). These findings further corroborate the genetic relationships and population structure insights obtained from NJ tree and PCA analyses.

### 3.3 Selective Signals in the Changle Goose

**FIGURE 3:**
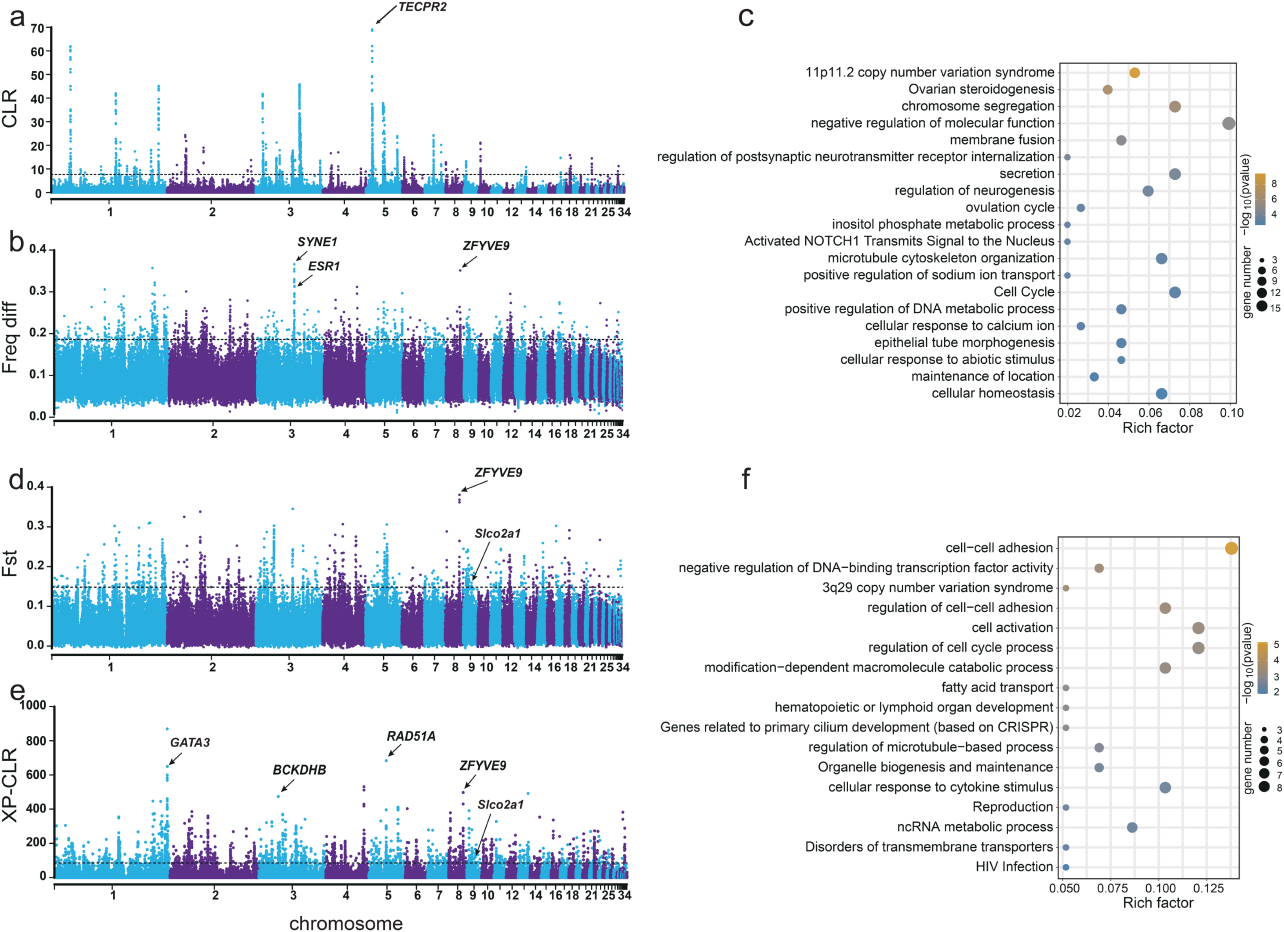
Genomic signatures of selection in the Changle goose (CLG). (a) Manhattan plot illustrating the composite likelihood ratio (CLR) for the CLG across autosomes, with a dashed line indicating the significance threshold for the top 0.1% of regions. (b) Frequency difference (Freq diff) plot across autosomes showing variation between the CLG and other Chinese goose breeds, with the dashed line marking the top 1% significance threshold. (c) Gene ontology (GO) term enrichment analysis for genes identified by both CLR and Freq diff, presenting the −log10 (*p* value) on the *y*‐axis. (d) Manhattan plot of the fixation index (Fst) indicating genomic differentiation between the CLG and other breeds. (e) Manhattan plot of cross‐population extended haplotype homozygosity (XP‐CLR) identifying regions suggestive of positive selection. (f) Enrichment analysis of GO terms for genes identified by both Fst and XP‐CLR, highlighting the biological processes potentially under selection.

To decipher the genetic differentiation of the CLG from other Chinese indigenous goose breeds, we employed multiple methodologies, including composite likelihood ratio (CLR), frequency difference (Freq diff), fixation index (Fst), and cross‐population composite likelihood ratio (XP‐CLR) tests, to detect regions under positive selection within the CLG genome (Figure 3). The CLR test revealed 965 regions harboring 486 annotated genes in the top 0.1%, which were deemed potential targets of selection (Figure 3a; Table S4). To elucidate the biological implications of these candidate genes, GO enrichment and KEGG pathway analyses were conducted, revealing pathways pertinent to chromatin segregation and the estrogen signaling pathway, as well as cell cycle processes that may be integral to the reproductive and growth attributes of CLG (Figure S4). Moreover, within the CLG population, the *TECPR2* gene on Chromosome 5 was observed to be under the strongest selection, indicating its probable significant influence on breed development. By applying a threshold for the top 1% of outlier windows, we identified 1027 candidate regions containing 328 genes, as indicated by Freq diff values (Figure 3b; Table S5). Genes such as *ESR1*, *ZFYVE9*, *SYNE1*, *FAF1*, and *CD82* were notably annotated. Further GO and KEGG pathway analyses comparing CLG to other indigenous breeds illuminated pathways instrumental in skeletal system development and mitotic cell cycle phase transition, likely influencing the growth profiles of the geese (Figure S5). The combined CLR and Freq diff approaches yielded 154 genes exhibiting significant signals (CLR top 0.1% and Freq diff top 1%), associated with processes such as ovarian steroidogenesis, chromosome segregation, ovulation cycle, and cell cycle, all of which may be crucial to the reproductive efficiency of CLG (Figure 3c). Importantly, the identification of *CD82* and *FAF1* genes, known for their roles in growth and reproduction, respectively, across two analytical methods suggests their significant selective benefit in CLG.

To enhance the detection of selection signals, we segmented the CLG genome into discrete, non‐overlapping 10 kb segments for comparative analysis with five other indigenous goose breeds. We identified 1008 candidate regions falling within the top 1% of the Fst, encompassing 311 candidate genes (Table S6). Notably, the region harboring the *ZFYVE9* gene exhibited the most pronounced selection signal (Fst = 0.37, Figure 3d). GO and KEGG analyses identified critical pathways involving the “regulation of cytokinesis” and “ovulation,” potentially correlating with the rapid growth and reduced egg production observed in CLG (Figure S6). XP‐CLR analysis further delineated 2035 candidate regions (top 1% XP‐CLR), covering 531 candidate genes (Table S7), with significant enrichment in “Separation of Sister Chromatids” and “regulation of cell cycle process” pathways (Figure S7). Genes including *GATA3*, *RAD51A*, and *ZFYVE9* displayed notable selection signals (Figure 3e), with *RAD51A* recognized for its regulatory role in meiosis (Khoo, Jolly, and Able 2008). Integrating findings from both Fst and XP‐CLR analyses, we highlighted 276 candidate regions, incorporating 58 genes (Table S8), predominantly associated with pathways involved in “fatty acid transport,” “reproduction,” and “cell cycle regulation” (Figure 3f). Additionally, we noted significant haplotypic variances in genes such as *PNPLA8*, *SLC35A3*, *FABP4*, and *ZFYVE9* between CLG and other populations (Figure S8). Furthermore, we found significant differences in the haplotypes of *PNPLA8*, *SLC35A3*, *FABP4*, and *ZFYVE9* genes between CLG and other goose breeds as well as wild populations (Figure S7).

### 3.4 Candidate Genes Under Selective Sweep in the Changle Goose

**FIGURE 4:**
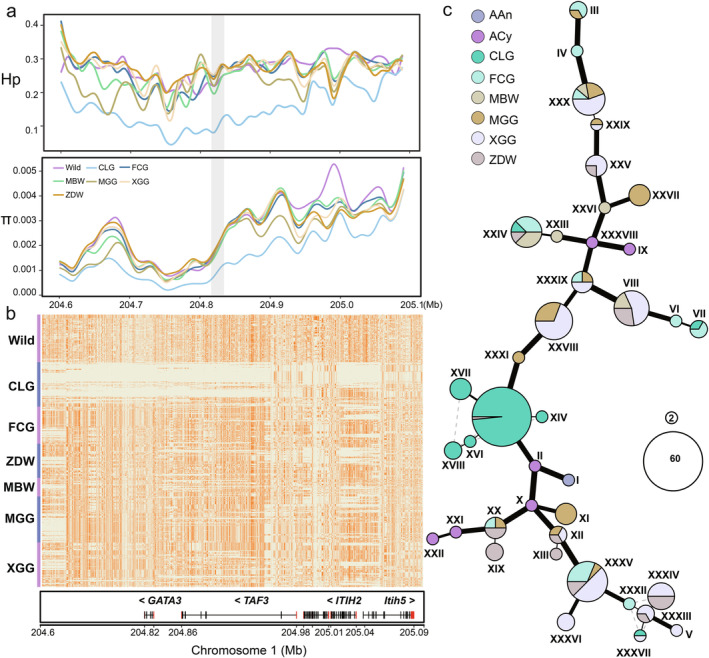
Genetic variation and haplotype structure of the *GATA3* gene in Changle goose (CLG) and comparative breeds. (a) Nucleotide diversity (π) and heterozygosity (Hp) are measured in the genomic region encompassing the *GATA3* gene across different goose populations. (b) Heatmap of haplotype distribution surrounding the *GATA3* gene, illustrating the allelic variation between CLG and other breeds, with major alleles marked in dark orange and minor alleles in honeydew. (c) Haplotype network for the *GATA3* gene, with a node representing a haplotype. Node size reflects the frequency of each haplotype, while the connections depict genetic distances, indicating the degree of allelic differentiation among haplotypes. Breed abbreviations refer to Table S1.

The CLG is renowned for its tender and flavorful meat, a trait highly valued by consumer. Our analysis of the genetic variation, particularly nucleotide diversity (π) and heterozygosity (Hp) proximal to the *GATA3* gene, revealed a diminished diversity in CLG, indicative of strong selective pressures within this genomic locale (Figure 4a). Haplotype analysis of *GATA3* in two wild goose species and six indigenous breeds revealed that CLG predominantly possesses a unique fixed haplotype (Figure 4b). Additionally, we constructed a haplotype network for *GATA3* (Figure 4c), which highlighted eight major haplotypes within CLG. The most frequent haplotype occurred 64 times across the analyzed goose breeds, with 63 times in CLG and one time in ZDW (Table S9). We also identified four haplotypes exclusive to CLG, underscoring the breed's distinct genetic makeup and suggesting it has been subjected to intensive artificial selection.

Further, the CLG displays a compact size and accelerated growth among Chinese indigenous breeds. Comparative genomic analysis revealed marked differences in the *CD82* gene haplotypes between CLG and their wild counterparts (Figure S9a). Moreover, the CLG exhibited the lowest nucleotide diversity and Hp within the *CD82* locus (Figure S9b), suggesting potential selective pressures acting on this gene. Haplotype network analysis identified five predominant haplotypes in the *CD82* gene among CLG populations (Figure S9c), which underscores the unique haplotypic patterns found in the CLG compared to other breeds and wild populations. Notably, the most prevalent haplotype was observed 25 times across three different breeds, with occurrences most frequent in CLG (*n* = 14), followed by FCG (*n* = 3), and XGG (*n* = 9), suggesting recent emergence and selection (Table S10). The absence of these predominant haplotypes in wild populations implies that they are novel derivatives, having emerged through recent mutational events followed by selection, which probably involved a substantial genetic hitchhiking effect, rather than these haplotypes being present in the ancestral gene pool.

**FIGURE 5:**
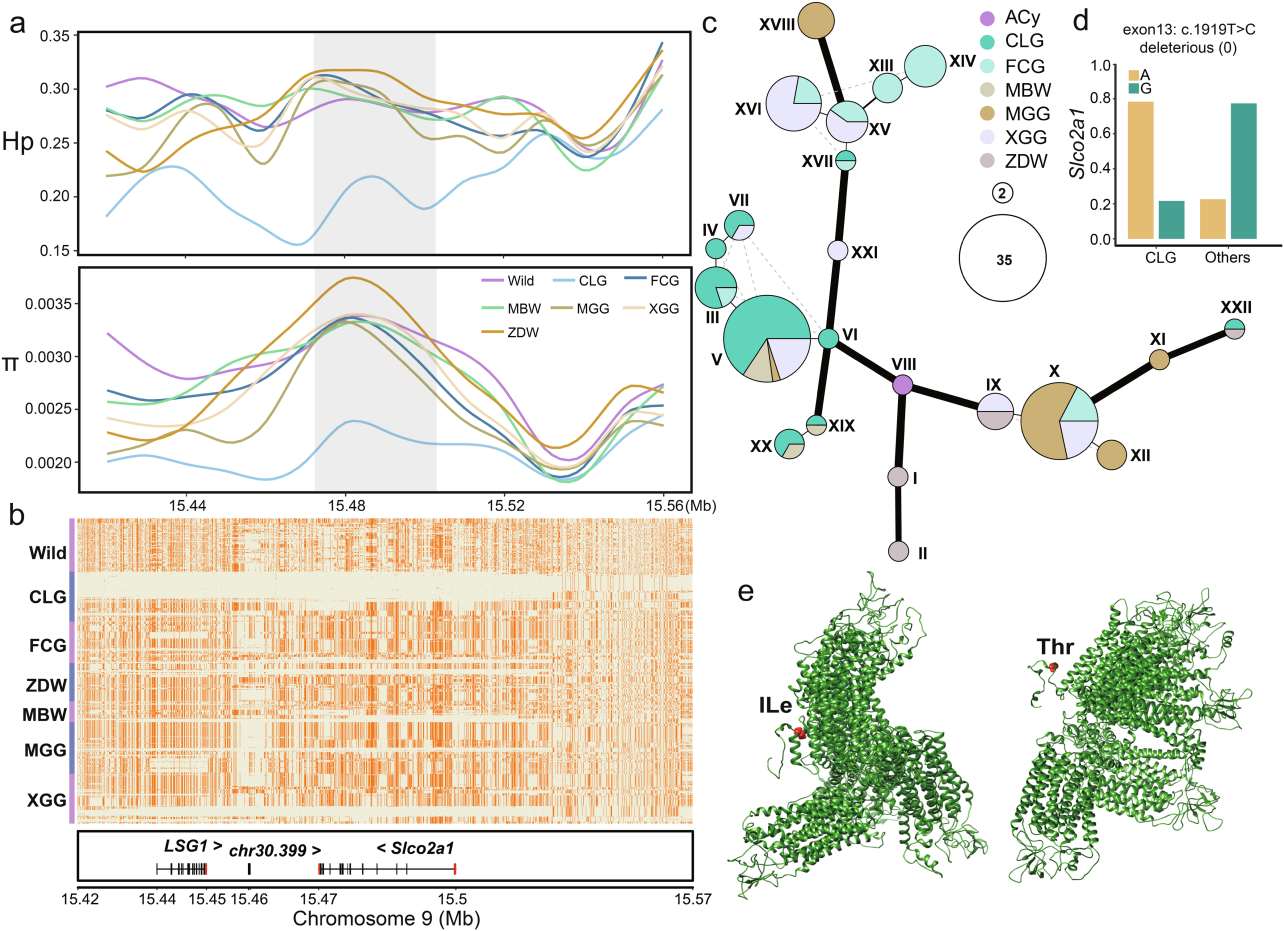
Genetic diversity and haplotype distribution of the *Sloc2a1* gene in Changle goose. (a) Graphical representation of nucleotide diversity (π) and heterozygosity (Hp) in the regions flanking the *Sloc2a1* gene. (b) Heatmap illustrating the haplotypes of the *Sloc2a1* gene in CLG compared with various goose populations, with major alleles shown in dark orange and minor alleles in honeydew. (c) Haplotype network diagram for the *Sloc2a1* gene, where each node represents a distinct haplotype and its size denotes the frequency. Lines between nodes reflect genetic distances, illustrating allelic divergence. (d) Bar graph showing the allele frequencies of missense mutations within the *Sloc2a1* gene across different goose breeds, with the deleterious impact of amino acid substitutions predicted by SIFT. (e) Conformational space prediction for the *Slco2a1* protein between CLG and other breeds based on structural modeling. Refer to Table S1 for breed abbreviations.

In terms of reproductive traits, the CLG is distinguished by pronounced broodiness and decreased egg production. Our findings demonstrate significantly lower π and Hp at the *Slco2a1* locus in CLG as compared to both wild types and other domestic breeds (Figure 5a). Haplotype heatmap analysis revealed distinctive haplotypic patterns in CLG, which differ from those observed in wild and other domestic populations (Figure 5b). The haplotype network for the *Slco2a1* gene disclosed nine major haplotypes within the CLG genome. The most prevalent haplotype appeared 23 times within CLG, contrasting with its presence in other breeds: four times in MBW, once in MGG, and seven times in XGG (Figure 5c; Table S11).

Further examining genetic divergence, we identified 30 SNPs within the *Slco2a1* gene, each with allele frequency differences greater than 0.5. Annotation revealed three synonymous and one non‐synonymous mutation (c.T1919C:p.I640T) in the coding region of *Slco2a1*, with the non‐synonymous mutation potentially impacting function (Figure 5d). Predictive assessments of deleteriousness identified this non‐synonymous mutation in *Slco2a1* as likely harmful, with a SIFT score of 0, indicating a possible impact on the *Slco2a1* protein structure due to an isoleucine‐to‐threonine (Ile‐to‐Thr) substitution (Figure 5e).

## 4 Discussion

The preservation of genetic resources in indigenous animals is pivotal for biodiversity conservation, as they often display remarkable adaptability to diverse environments. Such genetic diversity equips breeders with the means to conduct artificial selection and cultivate new breeds that align with societal demands (Hoffmann 2010). Nevertheless, the adverse impacts of environmental degradation, coupled with a narrow focus on the economic attributes of indigenous animal genetic resources in agriculture—while overlooking their ecological and cultural significance—has led to the decline and endangerment of vital breeds (Bruford 2004; Pouta, Tienhaara, and Ahtiainen 2014). The genomic analysis of indigenous animals, now enhanced by WGS, provides critical insights into the genetic underpinnings of traits valued both in traditional agriculture and for broader biodiversity conservation efforts, as evidenced in geese (Ouyang et al. 2022), ducks (Chen et al. 2023), and cattle (Lyu et al. 2023).

The CLG, a breed distinguished by its dual utility and gastronomically acclaimed meat, is a subject of consumer preference and has been under conservation since 1998, with concerted initiatives supported by government interventions to preserve its lineage. Understanding population structure and genetic diversity is fundamental to genetic resource assessment, as these parameters can reveal the effects of recent hybridization events, breeding practices, and the stewardship or exploitation of genetic resources (Guan et al. 2022). This study, through whole‐genome resequencing of 303 CLG and comparison with five other Chinese indigenous breeds, has delineated distinctive genomic features, offering insights into the breed's genetic landscape. The investigation of population structure and genetic diversity revealed that the CLG harbors lower nucleotide diversity, a decreased count of rare variants, reduced heterozygosity, and elevated linkage disequilibrium, alluding to a constricted genetic base. Such reduced polymorphism, as indicated by diminished π values, coupled with heightened inbreeding coefficients and slower decay of LD relative to other Chinese geese, points to a marked contraction in genetic diversity. Additionally, both SV‐ and SNP‐based population structure analyses indicate significant differences among these six Chinese local goose breeds, although their genetic distances are relatively close. Gene flow analysis revealed significant migration events from XGG to CLG, likely due to their geographical proximity, with XGG in Jiangxi Province adjacent to Fujian Province where CLG is found, suggesting that XGG may have contributed to the formation of CLG. CLG, MBW, and ZDW show close genetic distances, and gene flow analysis also indicates significant gene flow between MBW and ZDW, suggesting that plumage color correlates with greater genomic similarity. These findings underscore the imperative need for informed breeding strategies and comprehensive management practices to preserve the genetic diversity of the CLG. Diligent stewardship is essential to maintain the adaptability of this breed over time and to minimize the risk of genetic bottlenecking. Implementing such measures is crucial for the sustainability of agricultural biodiversity and for the protection of heritage breeds for future generations.

The CLG is highly valued for its meat, distinguished by tenderness and a rich flavor profile favored by consumers. Intramuscular fat (IMF), a key factor in meat quality, is known to enhance tenderness, juiciness, and flavor (Anderson et al. 2015; Luo et al. 2022; Zhang and Li et al. 2018), while inosine monophosphate (IMP) contributes significantly to the delicate flavor profile of meat, especially after thermal reactions with guanylate (Zhang et al. 2021; Zhang and Lu et al. 2018). In our study, we utilized CLR, Fst, Freq diff, and XP‐CLR analyses to uncover genomic regions under selective pressure within the CLG. Notably, variation within the *GATA3* gene was identified between CLG and other geese, with a reduced genetic diversity observed in CLG, suggesting a targeted selection for meat quality traits. The involvement of the *GATA3* gene in the browning of adipose tissue—through its interaction with proliferator‐activated receptor‐γ co‐activator‐1α (PGC‐1α) and subsequent upregulation of uncoupling protein‐1 (UCP‐1)—links it to fat regulation, which is instrumental in meat quality (Son et al. 2020). Moreover, our analysis revealed the presence of the *ACOT1* gene, previously associated with fat and protein content in water buffalo (Liu et al. 2018), now implicated in the lipid metabolism of CLG. Additionally, genes like *SLC35A3*, associated with abdominal fat deposition (Zhang et al. 2020), and *Cdkal1*, integral to mitochondrial function within adipose cells (Palmer et al. 2017), were also identified, underscoring their relevance to fat accumulation and distribution—a crucial aspect of meat quality. These genetic insights lay a foundation for future genomic exploration of the CLG and inform breeding strategies aimed at enhancing meat quality. Understanding the genomic basis of these desirable traits has the potential to enhance not only the CLG but also other poultry breeds, thereby aligning production more closely with consumer preferences and market demands.

In contrast to other Chinese indigenous goose breeds, the CLG is distinguished by its smaller stature and exceptionally rapid growth and development. Our genomic analysis has pinpointed several genes playing crucial roles in these distinctive growth traits. Notably, the *CD82* gene has been identified as a critical factor in developmental processes. Bergsm (Bergsma et al. 2018) observed that diminished expression of *CD82* adversely impacts skeletal development and leads to increased accumulation of adipose tissue in the bone marrow. This is corroborated by studies employing micro‐computed tomography and four‐point bending assays, which associate *CD82* deficiency with reduced bone size and structural integrity, while not affecting bone mineral density. In our study, the *CD82* gene in CLG exhibited significantly lower nucleotide diversity and heterozygosity levels compared to wild geese and other local breeds, suggesting strong selective pressures on this gene and potentially explaining the observed differences in body size between CLG and other breeds.

Furthermore, we noted that mutations in the *CDH8* gene are associated with an overgrowth phenotype (Ostrowski et al. 2019), and *PRKAB1* has been linked to growth traits in goats (Zhou et al. 2022) and broiler chickens (Jin et al. 2016), demonstrating correlations with body weight gain and feed intake. The identification of the *SLC35A3* gene, known for its role in skeletal development (Zhang et al. 2020), adds to the genetic factors influencing the physical growth of CLG. Additionally, the *FABP4* gene, involved in regulating steroid hormone secretion in goose granulosa cells (Fan et al. 2021), and its association with growth traits in Egyptian sheep (Shafey et al. 2020), underline the complex genetic basis of growth in CLG. The elucidation of these genetic markers offers a robust framework for advancing the understanding of growth and development in CLG, contributing significantly to the genetic improvement and variety formation of goose.

The CLG is characterized by marked broodiness and reduced egg production rates, which severely limits the large‐scale commercial farming of this breed. We have identified a suite of genes, including *Slco2a1*, *FABP4*, *ESR1*, *AKAP12*, and *FAF1*, which are intricately linked to reproductive processes. Notably, the *Slco2a1* gene is pivotal in reproductive biology, playing a critical role in luteolysis, maternal recognition of pregnancy, and parturition in livestock (Seo et al. 2014). It encodes a prostaglandin transporter protein, essential for prostaglandin (PG) transport, a process integral to key reproductive functions (Dorniak, Bazer, and Spencer 2011; Kanai et al. 1995; Khan, Carson, and Nelson 2008; Kraft et al. 2010; Sugimoto et al. 1997). The significance of this gene is further emphasized in its role in maintaining pregnancy in swine and facilitating parturition in murine models (Inagaki et al. 2020; Seo et al. 2014). Additionally, the *FABP4* gene, crucial in regulating steroid hormone secretion in goose ovarian granulosa cells (Fan et al. 2021), highlights the complex interplay of ovarian steroid hormones in oocyte production and development. The *ESR1* gene is involved in follicular development and is essential for the surge of luteinizing hormone (LH) prior to ovulation, and its absence can disrupt the estrous cycle in mice (Porteous and Herbison 2019). The *AKAP12* gene, also instrumental in ovarian function, has been linked to reduced litter size in goats due to a specific gene deletion (Kang et al. 2021). Furthermore, the expression of *FAF1* in oocytes at various stages of ovarian follicle development and its fluctuating expression in ovarian tissues underline its importance in the reproductive cycle (Wang et al. 2023). The association of the *ZFYVE9* gene with reproductive traits in pigs (Li et al. 2020) adds another layer to our understanding of reproductive genetics in CLG. The research on heat stress demonstrates that the c.3989G>A variant of the *TECPR2* gene correlates with heat tolerance in Chinese cattle (Ma et al. 2023), revealing potential genetic factors that might influence laying performance under thermal stress. This gene‐centric investigation enhances our understanding of the genetic factors influencing reproductive traits in CLG and elucidates the genetic underpinnings of its reproductive performance, thereby proving crucial for developing targeted strategies that are essential for improving reproductive efficiency, ensuring sustainable management, and facilitating preservation.

## 5 Conclusions

This study has effectively unraveled the unique genetic features of CLG, differentiating it from other Chinese goose breeds through detailed analyses of SNPs and SVs. Notably, CLG exhibits fewer SVs and reduced genetic diversity compared to its counterparts. Our research has been pivotal in identifying and clarifying the roles of key genes across various biological pathways. For instance, *GATA3*, *SLC35A3*, and *Cdkal1* have emerged as vital in fat regulation, while *Slco2a1*, *FABP4*, *ESR1*, and *AKAP12* play significant roles in follicular development. Additionally, genes such as *CDH8*, *PRKAB*, and *CD82* have been identified as influential in the growth and developmental processes of CLG. The results significantly advance our understanding of the genomic uniqueness of CLG and establish a solid theoretical foundation for future research endeavors. These findings underpin the importance of genetic resource assessment and inform the development of precise conservation strategies, thereby contributing significantly to the sustainable management and preservation of this unique breed.

## Ethics Statement

All experimental protocols were sanctioned by the Animal Ethics Committee of Jiangxi Science and Technology Normal University (3601020137931) and adhered rigorously to the Guidelines of China Animal Welfare Association.

## Conflicts of Interest

The authors declare no conflicts of interest.

## Supporting information


Data S1.

## Data Availability

The Xingguo gray goose genome used in this study has been deposited in the Genome Warehouse in BIG Data Center (https://bigd.big.ac.cn/gwh/) under accession number GWHBAAW00000000. The genome resequencing data for goose have been deposited in the National Genomics Data Center (NGDC) as BioProject PRJCA021154. The SNP dataset of domestic and wild geese has been deposited in the Genome Variation Map in BIG Data Center (https://bigd.big.ac.cn/gvm/) under accession number GVM000664.
