# Supplementary material for: Deciphering the Genetic Landscape: Insights Into the Genomic Signatures of Changle Goose
Source: Evol Appl. 2024 Aug 22;17(8):e13768. doi: 10.1111/eva.13768 (PMC11340016; doi:10.1111/eva.13768)
Supplement: Supplementary file 1 — Data S1. [file EVA-17-e13768-s001.docx]

Deciphering the genetic landscape: Insights into the genomic signatures of Changle goose

Hao Chen^1^^†^, Yan Wu^1†^, Yihao Zhu^1^, Keyi Luo^1^, Sumei Zheng^1^, Hongbo Tang^1^, Rui Xuan^1^, Yuxuan Huang^1^, Jiawei Li^1^, Rui Xiong^1^, Xinyan Fang^1^, Lei Wang^1^, Yujie Gong^1^, Junjie Miao^1^, Jing Zhou^1^, Hongli Tan^1^, Yanan Wang^1^, Liping Wu^1^, Jing Ouyang^1^, Ming Huang^2*^, Xueming Yan^1*^

^1^College of Life Sciences, Jiangxi Science and Technology Normal University, Nanchang 330013, China

^2^College of Animal Sciences & Technology, Zhejiang A&F University, Hangzhou, China

^†^These authors contributed equally to this study.

^*^Correspondence to: MinHuang0702@outlook.com; xuemingyan@hotmail.com

**Supplementary Figures**

**
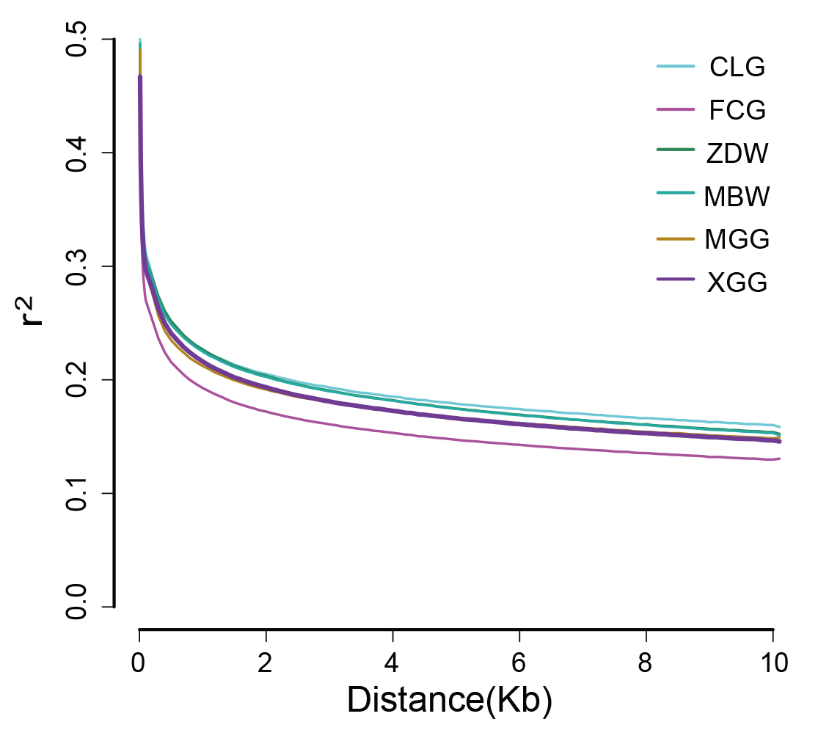
**

**Figure S1. Comparative analysis of Linkage Disequilibrium Decay (LD decay) among goose breeds.** The LD decay is measured by the squared correlation coefficient (r²) between pairs of single nucleotide polymorphisms (SNPs). Each line represents one of the six goose breeds analyzed: Changle goose (CLG), Fengcheng gray goose (FCG), Zhedong white goose (ZDW), Minbei white goose (MBW), Magang goose (MGG), and Xingguo gray goose (XGG).

**
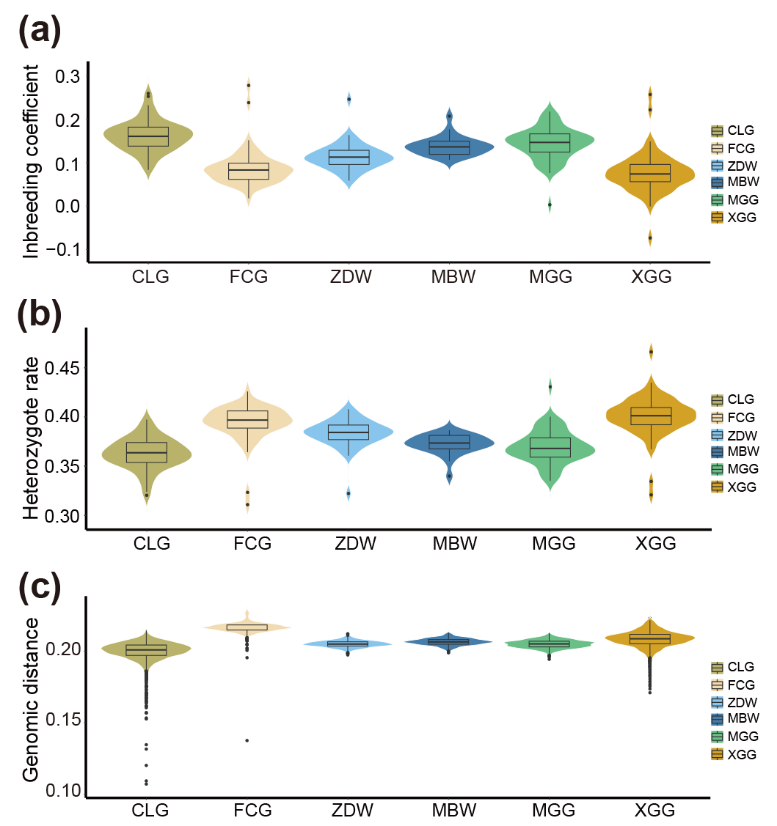
**

**Figure S2. Genetic variability metrics across six goose breeds.** (a) Displays the distribution of the inbreeding coefficient (F) for each breed, indicating the degree of homozygosity within the populations. (b) Depicts the heterozygosity rate of each breed, reflecting the genetic diversity present within each group. (c) Shows the genomic distance at a whole-genome scale for each breed. For breed abbreviations, please refer to Table 1.


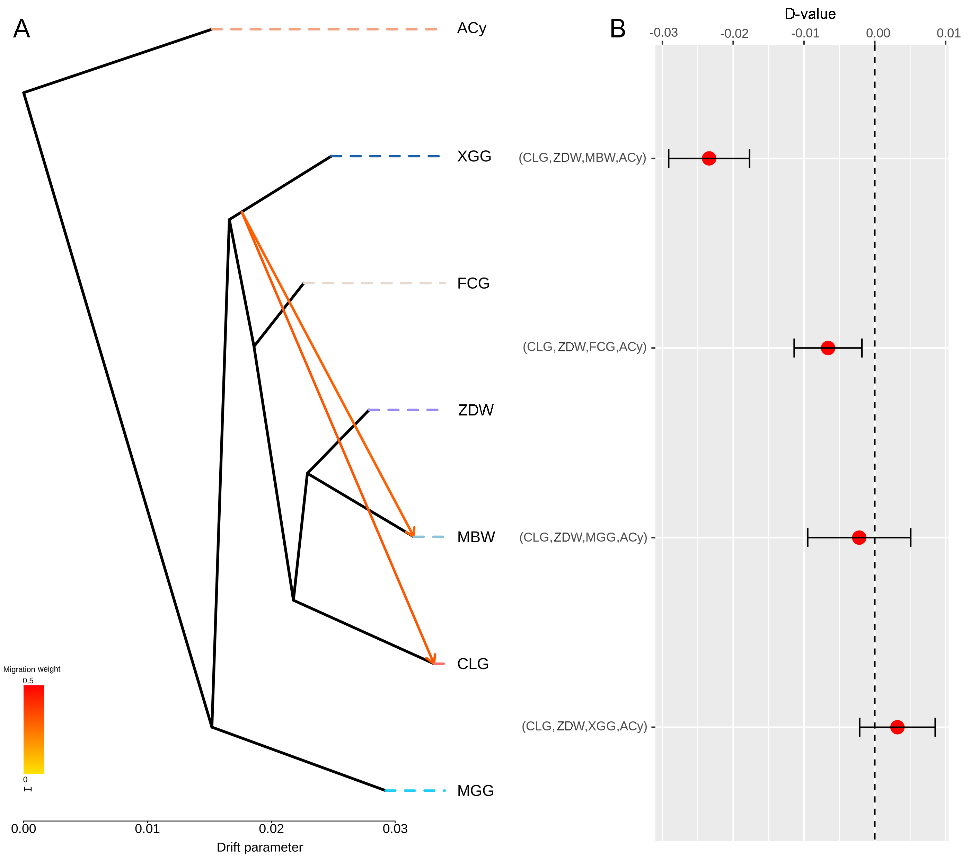


**Figure S3. Gene flow between Changle geese and other geese.** (a) Treemix analysis with ACy as the root. An arrowed line indicates a migration event between two groups. (b) D-test modeled as (CLG, ZDW, other breed, ACy). The other breed category includes MBW, FCG, MGG, and XGG.

**
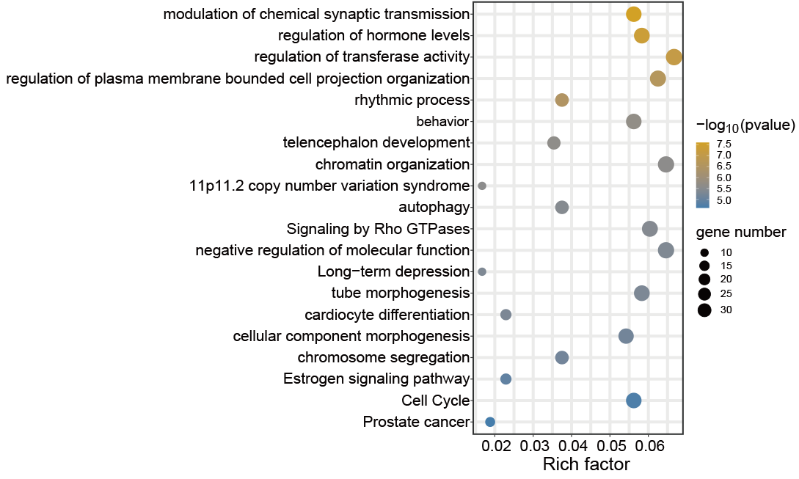
**

**Figure S4. Enrichment analysis of candidate genes identified by the CLR.**

**
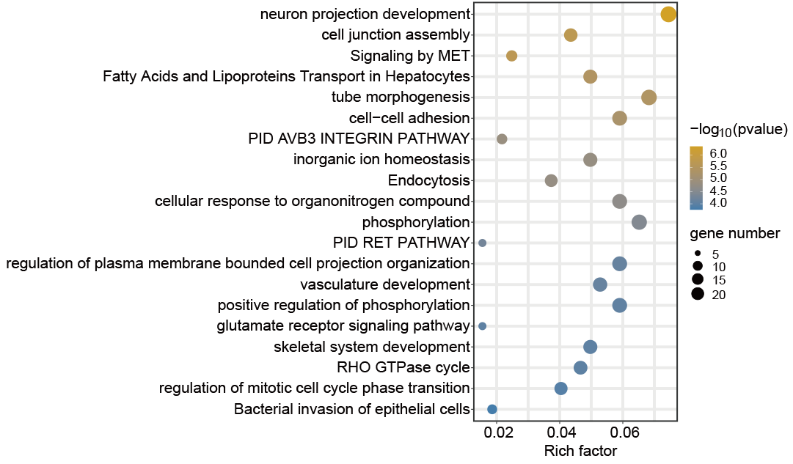
**

**Figure S5. Enrichment analysis of candidate genes identified by the Freq diff.**

**
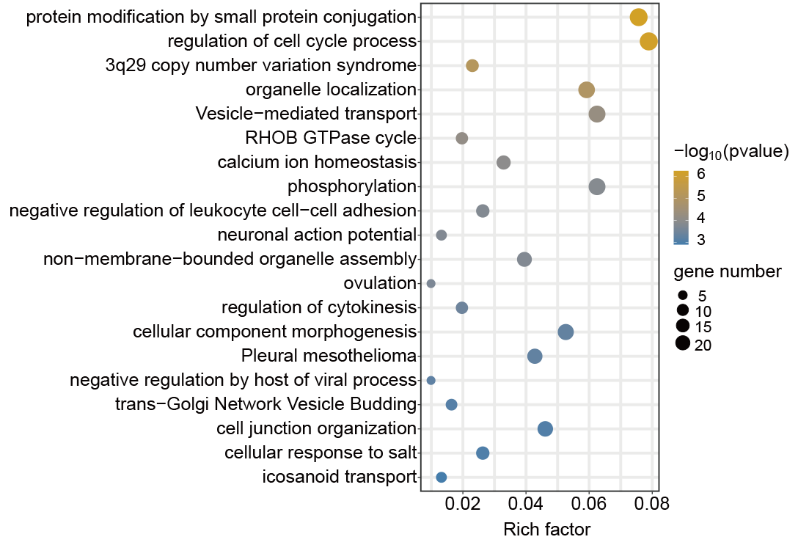
**

**Figure S6. Enrichment analysis of candidate genes identified by the Fst.**

**
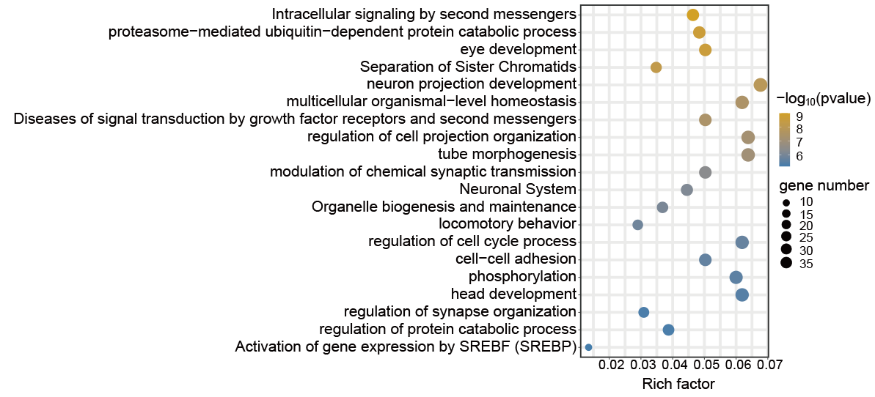
**

**Figure S7. Enrichment analysis of candidate genes identified by the XP-CLR.**


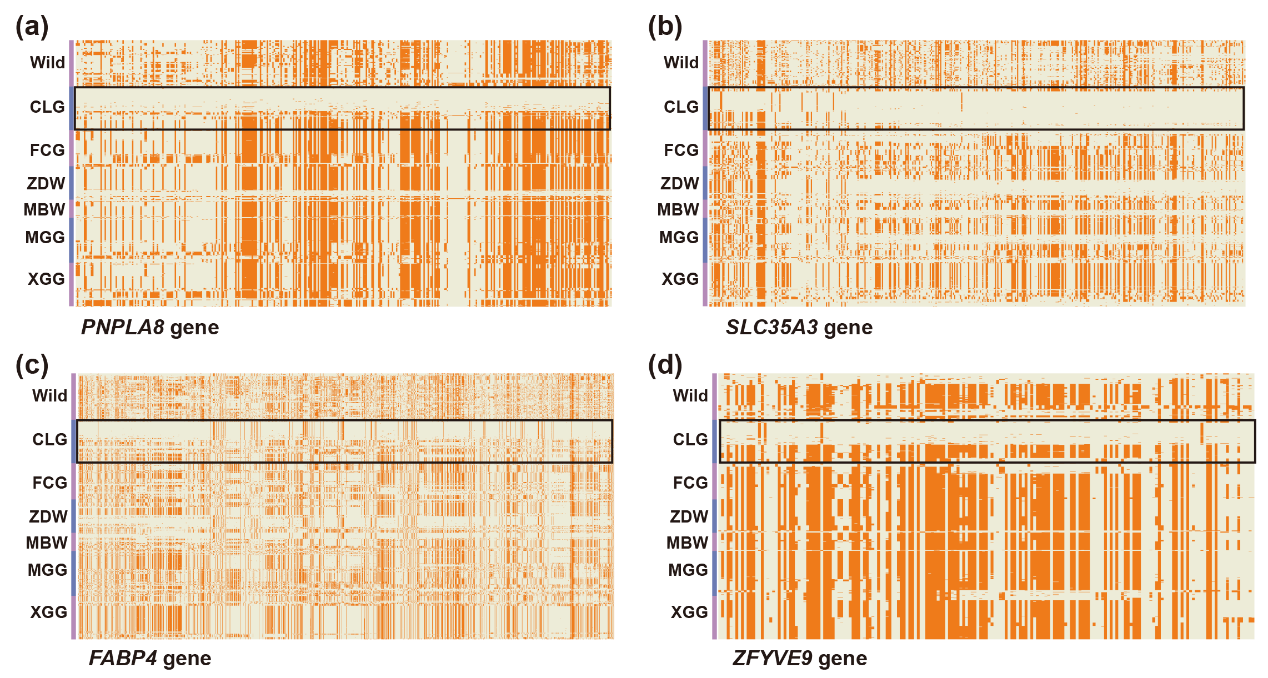
 **Figure S8. Haplotype visualization for selective genes in wild and domestic geese populations.** Panels a to d display the haplotypes of the *PNPLA8*, *SLC35A3*, *FABP4*, and *ZFYVE9* genes, respectively. Major alleles are represented in beige, and minor alleles are in coral. The black box highlights the haplotypes found within the CLG population. ACy denotes *Anser cygnoides* and AAn denotes *Anser anser*. The breed abbreviation, please refer to Table 1.


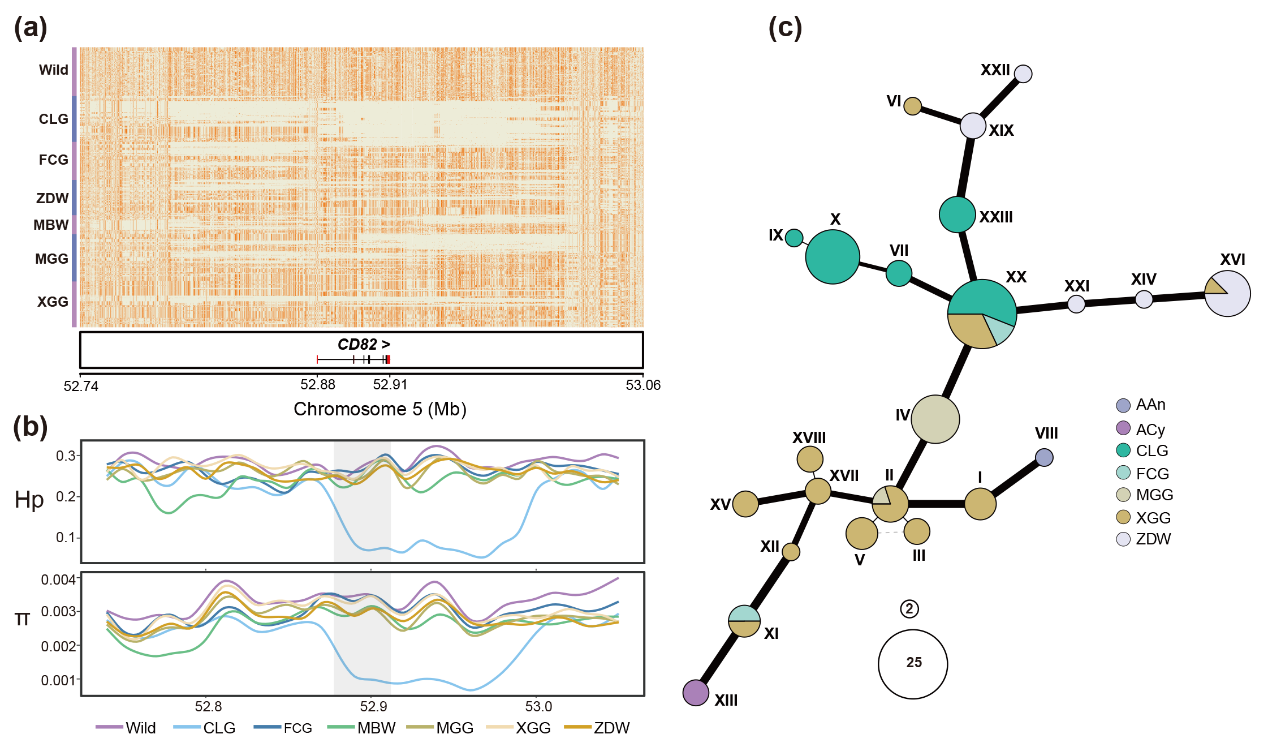
 **Figure S9. Genetic structure and diversity of the *CD82* gene across goose populations.** (a) Allelic variation in the *CD82* locus across various goose populations. The heatmap depicts a comparison of haplotypes between CLG and other breeds, with major alleles denoted in dark orange and minor alleles in honeydew. The distinct genetic makeup of CLG in the context of *CD82* is highlighted. (b) The nucleotide diversity (π) and heterozygosity (Hp) indices surrounding the *CD82* gene are illustrated for each breed. These metrics provide insights into the genetic variation and potential selective pressures on the *CD82* locus. (c) Haplotype network for the *CD82* gene. Each node represents a haplotype, scaled in proportion to its frequency within the population, and connections indicate genetic distances between haplotypes. Breed abbreviations are as per Table S1.

**Supplementary Tables**

**Table S1. Information of 367 avian samples spanning wild and domesticated geese populations.**

| Type | Population | Abbreviation | Number |
| --- | --- | --- | --- |
| Wild | *Anser anser* | AAn | 5 |
| Wild | *Anser cygnoides* | ACy | 59 |
| Domesticated | Changle goose | CLG | 60 |
| Domesticated | Fengcheng gray goose | FCG | 50 |
| Domesticated | Minbei white goose | MBW | 25 |
| Domesticated | Magang goose | MGG | 62 |
| Domesticated | Xingguo gray goose | XGG | 60 |
| Domesticated | Zhedong white goose | ZDW | 46 |

**Table S2. The distribution of variants in the Changle goose (CLG).**

| Chromosome | Length | Variants | Variant rate |
| --- | --- | --- | --- |
| 1 | 209,620,174 | 1,063,923 | 0.51% |
| 2 | 161,039,598 | 816,070 | 0.51% |
| 3 | 121,834,228 | 615,835 | 0.51% |
| 4 | 77,974,103 | 358,320 | 0.46% |
| 5 | 65,921,219 | 338,907 | 0.51% |
| 6 | 40,747,672 | 227,065 | 0.56% |
| 7 | 38,069,285 | 224,839 | 0.59% |
| 8 | 32,877,965 | 188,760 | 0.57% |
| 9 | 26,787,924 | 161,143 | 0.60% |
| 10 | 22,597,973 | 137,466 | 0.61% |
| 11 | 22,240,343 | 131,155 | 0.59% |
| 12 | 21,986,313 | 112,591 | 0.51% |
| 13 | 21,518,999 | 120,985 | 0.56% |
| 14 | 20,088,054 | 125,027 | 0.62% |
| 15 | 18,112,843 | 106,414 | 0.59% |
| 16 | 16,305,488 | 109,648 | 0.67% |
| 17 | 15,520,627 | 93,730 | 0.60% |
| 18 | 13,049,523 | 82,518 | 0.63% |
| 19 | 12,194,549 | 82,373 | 0.68% |
| 20 | 12,134,004 | 82,212 | 0.68% |
| 21 | 8,883,100 | 61,784 | 0.70% |
| 22 | 7,890,303 | 56,174 | 0.71% |
| 23 | 7,862,079 | 64,444 | 0.82% |
| 24 | 7,025,632 | 51,467 | 0.73% |
| 25 | 6,441,300 | 43,984 | 0.68% |
| 26 | 6,092,936 | 37,698 | 0.62% |
| 27 | 5,778,200 | 38,961 | 0.67% |
| 28 | 3,186,445 | 17,528 | 0.55% |
| 29 | 2,916,307 | 14,526 | 0.50% |
| 30 | 2,484,068 | 10,376 | 0.42% |
| 31 | 2,265,387 | 3,481 | 0.15% |
| 32 | 1,463,973 | 1,215 | 0.08% |
| 33 | 1,448,066 | 4,027 | 0.28% |
| 34 | 1,395,719 | 675 | 0.05% |
| 35 | 1,291,483 | 5,759 | 0.45% |
| 36 | 901,259 | 1,242 | 0.14% |
| 37 | 873,800 | 3,020 | 0.35% |
| 38 | 673,561 | 385 | 0.06% |
| 39 | 83,392,258 | 205,833 | 0.25% |
| Total | 1,107,366,133 | 5,801,560 | 0.52% |

**Table S3. Annotation of CLG-specific Single Nucleotide Polymorphisms (SNPs).**

| Type | Number | percent |
| --- | --- | --- |
| 3’ UTR variant | 55,187 | 0.795% |
| 5’ UTR variant (start codon gained) | 1,378 | 0.020% |
| 5’ UTR variant | 7,775 | 0.112% |
| Downstream variant | 536,207 | 7.725% |
| Start codon variant | 51 | 0.001% |
| Intergenic region variant | 3,224,216 | 46.450% |
| Intron variant | 2,452,218 | 35.328% |
| Missense variant | 33,558 | 0.483% |
| Splice variant (acceptor) | 297 | 0.004% |
| Splice variant (donor) | 177 | 0.003% |
| Splice variant (region) | 12,332 | 0.178% |
| Start codon lost | 98 | 0.001% |
| Stop codon gained | 1,194 | 0.017% |
| Stop codon lost | 576 | 0.008% |
| Stop variant (retained) | 461 | 0.007% |
| Synonymous variant | 70,562 | 1.017% |
| Upstream variant | 542,603 | 7.817% |

**Table S4. Genomic regions exhibiting significant Composite Likelihood Ratio (CLR) values in the Changle goose**

| Chromosome | Start Position (bp) | End Position (bp) | CLR Value | Associated Gene(s) |
| --- | --- | --- | --- | --- |
| 1 | 32738977 | 33171175 | 10.8505 | *OLFM4* |
| 1 | 32757649 | 33154885 | 12.2140 | *OLFM4* |
| 1 | 34858053 | 36238738 | 61.7172 | *PCDH17* |
| 1 | 34860278 | 36238525 | 61.9223 | *PCDH17, DDB_G0269086, DIAPH3* |
| 1 | 34873074 | 36227886 | 60.4521 | *PCDH17, DDB_G0269086, DIAPH3* |
| 1 | 34874942 | 36220331 | 59.8793 | *PCDH17, DDB_G0269086, DIAPH3* |
| 1 | 34899606 | 36208259 | 57.2893 | *PCDH17, DDB_G0269086, DIAPH3* |
| 1 | 34906037 | 36189798 | 56.3098 | *PCDH17, DDB_G0269086, DIAPH3* |
| 1 | 34933074 | 36172409 | 52.2730 | *PCDH17, DDB_G0269086, DIAPH3* |
| 1 | 34944330 | 36146744 | 50.9300 | *PCDH17, DDB_G0269086, DIAPH3* |
| 1 | 34962813 | 36421691 | 30.9777 | *PCDH17, DDB_G0269086, DIAPH3, TDRD3* |
| 1 | 34963900 | 36422435 | 32.6323 | *PCDH17, DDB_G0269086, DIAPH3, TDRD3* |
| 1 | 34966361 | 36424010 | 32.2277 | *PCDH17, DDB_G0269086, DIAPH3, TDRD3* |
| 1 | 34980713 | 36126291 | 45.0503 | *PCDH17, DDB_G0269086, DIAPH3* |
| 1 | 34998883 | 36392086 | 29.9273 | *PCDH17, DDB_G0269086, DIAPH3, TDRD3* |
| 1 | 35001762 | 36087599 | 43.6161 | *PCDH17, DDB_G0269086, DIAPH3* |
| 1 | 35014041 | 36369115 | 26.4352 | *PCDH17, DDB_G0269086, DIAPH3, TDRD3* |
| 1 | 35023968 | 36370892 | 25.5777 | *PCDH17, DDB_G0269086, DIAPH3, TDRD3* |
| 1 | 35072035 | 36036781 | 35.1786 | *PCDH17, DDB_G0269086, DIAPH3* |
| 1 | 35075875 | 36319245 | 18.4629 | *PCDH17, DDB_G0269086, DIAPH3, TDRD3* |
| 1 | 35084572 | 36297168 | 15.2837 | *PCDH17, DDB_G0269086, DIAPH3, TDRD3* |
| 1 | 35096145 | 35953313 | 15.3933 | *DDB_G0269086* |
| 1 | 35099593 | 35947288 | 15.3804 | *DDB_G0269086* |
| 1 | 35106822 | 35979990 | 34.5622 | *DDB_G0269086* |
| 1 | 35118701 | 35925222 | 8.2616 | *DDB_G0269086* |
| 1 | 35127777 | 35911171 | 7.9623 | *DDB_G0269086* |
| 1 | 35144448 | 35890876 | 14.3488 | *DDB_G0269086* |
| 1 | 35144614 | 35908122 | 21.6741 | *DDB_G0269086* |
| 1 | 35181444 | 35847335 | 11.3480 | *DDB_G0269086* |
| 1 | 35197710 | 35856877 | 16.6948 | *DDB_G0269086* |
| 1 | 35213533 | 35896631 | 22.9302 | *DDB_G0269086* |
| 1 | 35216074 | 35811546 | 12.4003 | *DDB_G0269086* |
| 1 | 35216074 | 36163302 | 10.7074 | *DDB_G0269086, DIAPH3* |
| 1 | 35227572 | 35858783 | 24.5281 | *DDB_G0269086* |
| 1 | 35254526 | 35744203 | 9.4962 | *DDB_G0269086* |
| 1 | 35261903 | 35734724 | 10.7228 | *DDB_G0269086* |
| 1 | 35337270 | 36035307 | 11.9006 | *DDB_G0269086, DIAPH3* |
| 1 | 35372585 | 35709613 | 11.2962 | *DDB_G0269086* |
| 1 | 35415837 | 35729069 | 12.7075 | *DDB_G0269086* |
| 1 | 35433096 | 35690099 | 11.4890 | *DDB_G0269086* |
| 1 | 35440534 | 35990376 | 12.0671 | *DDB_G0269086* |
| 1 | 35446902 | 35690099 | 14.0402 | *DDB_G0269086* |
| 1 | 35453938 | 35977675 | 10.9754 | *DDB_G0269086* |
| 1 | 35461320 | 35601572 | 7.7702 | *DDB_G0269086* |
| 1 | 35474216 | 35690099 | 12.1866 | *DDB_G0269086* |
| 1 | 35479866 | 35949925 | 9.0662 | *DDB_G0269086* |
| 1 | 35572937 | 35730794 | 8.4684 | *DDB_G0269086* |
| 1 | 35587323 | 35754321 | 8.0192 | *DDB_G0269086* |
| 1 | 90159881 | 90362879 | 10.9860 | *Atp6ap2, MED14* |
| 1 | 90206285 | 90304078 | 7.8215 | *Atp6ap2, MED14* |
| 1 | 90489017 | 90741484 | 9.4478 | *chr38.1197, DDX3X, NYX, Cask, chr38.12000* |
| 1 | 93946687 | 94210895 | 7.6466 | *DSCAM* |
| 1 | 116504605 | 117147354 | 8.5847 | *ALCAM, CBLB* |
| 1 | 116504605 | 117184548 | 9.9487 | *CBLB* |
| 1 | 116504605 | 117202987 | 10.4506 | *ALCAM, CBLB* |
| 1 | 116504605 | 117219124 | 8.1550 | *ALCAM, CBLB* |
| 1 | 116504605 | 117220286 | 10.5345 | *CBLB, ALCAM* |
| 1 | 116504605 | 117227858 | 9.1424 | *CBLB, ALCAM* |
| 1 | 116504605 | 117232700 | 10.4100 | *CBLB* |
| 1 | 116504605 | 117233861 | 10.1533 | *CBLB* |
| 1 | 116504605 | 117238157 | 9.7527 | *ALCAM, CBLB* |
| 1 | 116590653 | 117068271 | 10.1674 | *ALCAM, CBLB* |
| 1 | 116590653 | 117070126 | 10.7638 | *CBLB* |
| 1 | 116590653 | 117092629 | 10.9756 | *ALCAM, CBLB* |
| 1 | 116590653 | 117106548 | 10.7035 | *CBLB* |
| 1 | 116590653 | 117107431 | 9.8921 | *ALCAM, CBLB* |
| 1 | 116590653 | 117108757 | 8.9428 | *ALCAM, CBLB* |
| 1 | 116713386 | 116942348 | 10.6443 | *ALCAM* |
| 1 | 116713386 | 116943295 | 8.5936 | *ALCAM* |
| 1 | 116713386 | 116971551 | 9.1577 | *ALCAM, CBLB* |
| 1 | 116713386 | 117057702 | 9.7980 | *ALCAM* |
| 1 | 116723999 | 116911773 | 8.7610 | *ALCAM* |
| 1 | 116983257 | 118491676 | 41.0269 | *ALCAM, CBLB, BBX, CD47, IFT57, HHLA2, Myh6, RCJMB04_1n3, SH2D1B, chr38.1750, TRAT1, Hjurp, GUCA1A, C3orf85* |
| 1 | 116993318 | 118480890 | 38.6103 | *ALCAM, CBLB, BBX, CD47, IFT57, HHLA2, Myh6, RCJMB04_1n3, SH2D1B, chr38.1750, TRAT1, Hjurp, GUCA1A, C3orf85* |
| 1 | 116995033 | 118483057 | 41.9555 | *ALCAM, CBLB, BBX, CD47, IFT57, HHLA2, Myh6, RCJMB04_1n3, SH2D1B, chr38.1750, TRAT1, Hjurp, GUCA1A, C3orf85* |
| 1 | 117009322 | 118461384 | 34.6667 | *ALCAM, CBLB, BBX, CD47, IFT57, HHLA2, Myh6, RCJMB04_1n3, SH2D1B, chr38.1750, TRAT1, Hjurp, GUCA1A, C3orf85* |
| 1 | 117010454 | 118469412 | 41.2992 | *ALCAM, CBLB, BBX, CD47, IFT57, HHLA2, Myh6, RCJMB04_1n3, SH2D1B, chr38.1750, TRAT1, Hjurp, GUCA1A, C3orf85* |
| 1 | 117031240 | 118450275 | 38.9577 | *CBLB, BBX, CD47, IFT57, Myh6, RCJMB04_1n3, SH2D1B, chr38.1750, TRAT1, Hjurp, GUCA1A, C3orf85* |
| 1 | 117041454 | 118431623 | 29.3391 | *CBLB, BBX, CD47, IFT57, HHLA2, Myh6, RCJMB04_1n3, SH2D1B, chr38.1750, TRAT1, Hjurp, GUCA1A, C3orf85* |
| 1 | 117072168 | 118412010 | 34.9151 | *CBLB, BBX, CD47, IFT57, HHLA2, Myh6, RCJMB04_1n3, SH2D1B, chr38.1750, TRAT1, Hjurp, GUCA1A, C3orf85* |
| 1 | 117105643 | 118362897 | 22.7603 | *CBLB, BBX, CD47, IFT57, HHLA2, Myh6, RCJMB04_1n3, SH2D1B, chr38.1750, TRAT1, Hjurp, GUCA1A, C3orf85* |
| 1 | 117122801 | 118363785 | 28.9989 | *CBLB, BBX, CD47, IFT57, HHLA2, Myh6, RCJMB04_1n3, SH2D1B, chr38.1750, TRAT1, Hjurp, GUCA1A, C3orf85* |
| 1 | 117159984 | 118306781 | 14.6497 | *CBLB, BBX, CD47, IFT57, HHLA2, Myh6, RCJMB04_1n3, SH2D1B, chr38.1750, TRAT1, Hjurp, GUCA1A, C3orf85* |
| 1 | 117202042 | 118288514 | 21.1337 | *BBX, CD47, IFT57, HHLA2, Myh6, RCJMB04_1n3, SH2D1B, chr38.1750, TRAT1, Hjurp, GUCA1A, C3orf85* |
| 1 | 117221821 | 118451900 | 37.5428 | *BBX, CD47, IFT57, HHLA2, Myh6, RCJMB04_1n3, SH2D1B, chr38.1750, TRAT1, Hjurp, GUCA1A, C3orf85* |
| 1 | 117227858 | 118443882 | 34.2467 | *BBX, CD47, IFT57, HHLA2, Myh6, RCJMB04_1n3, SH2D1B, chr38.1750, TRAT1, Hjurp, GUCA1A, C3orf85* |
| 1 | 117244185 | 118431623 | 33.3248 | *BBX, CD47, IFT57, HHLA2, Myh6, RCJMB04_1n3, SH2D1B, chr38.1750, TRAT1, Hjurp, GUCA1A, C3orf85* |
| 1 | 117250059 | 118420200 | 21.4634 | *BBX, CD47, IFT57, HHLA2, Myh6, RCJMB04_1n3, SH2D1B, chr38.1750, TRAT1, Hjurp, GUCA1A, C3orf85* |
| 1 | 117276717 | 118400771 | 16.7086 | *BBX, CD47, IFT57, HHLA2, Myh6, RCJMB04_1n3, SH2D1B, chr38.1750, TRAT1, Hjurp, GUCA1A, C3orf85* |
| 1 | 117361648 | 118287906 | 16.5201 | *BBX, CD47, IFT57, HHLA2, Myh6, RCJMB04_1n3, SH2D1B, chr38.1750, TRAT1, Hjurp, GUCA1A, C3orf85* |
| 1 | 117365777 | 118285289 | 9.6323 | *BBX, CD47, IFT57, HHLA2, Myh6, RCJMB04_1n3, SH2D1B, chr38.1750, TRAT1, Hjurp, GUCA1A, C3orf85* |
| 1 | 117376508 | 118268640 | 20.7793 | *BBX, CD47, IFT57, HHLA2, Myh6, RCJMB04_1n3, SH2D1B, chr38.1750, TRAT1, Hjurp, GUCA1A, C3orf85* |
| 1 | 117395825 | 118224135 | 14.8287 | *BBX, CD47, IFT57, HHLA2, Myh6, RCJMB04_1n3, SH2D1B, chr38.1750, TRAT1, Hjurp, GUCA1A, C3orf85* |
| 1 | 117397427 | 118092483 | 13.1903 | *BBX, CD47, IFT57, HHLA2, Myh6, RCJMB04_1n3, SH2D1B, chr38.1750, TRAT1, Hjurp, GUCA1A* |
| 1 | 117536164 | 118058042 | 11.2368 | *BBX, CD47, IFT57, HHLA2, Myh6, RCJMB04_1n3, SH2D1B, chr38.1750, TRAT1, Hjurp* |
| 1 | 117546976 | 118050875 | 21.7942 | *BBX, CD47, IFT57, HHLA2, Myh6, RCJMB04_1n3, SH2D1B, chr38.1750, TRAT1* |
| 1 | 117554382 | 117971737 | 18.6180 | *BBX, CD47, IFT57, HHLA2, Myh6, RCJMB04_1n3, SH2D1B* |
| 1 | 117574114 | 117917757 | 8.0786 | *BBX, CD47, IFT57, HHLA2, Myh6* |
| 1 | 117584991 | 117942841 | 8.6934 | *BBX, CD47, IFT57, HHLA2, Myh6* |
| 1 | 117586280 | 117937631 | 12.6788 | *BBX, CD47, IFT57, HHLA2, Myh6* |
| 1 | 117637658 | 118161347 | 8.9025 | *BBX, CD47, IFT57, HHLA2, Myh6, RCJMB04_1n3, SH2D1B, chr38.1750, TRAT1, Hjurp, GUCA1A* |
| 1 | 117643194 | 117956704 | 11.7540 | *BBX, CD47, IFT57, HHLA2, Myh6, RCJMB04_1n3* |
| 1 | 117650254 | 117909816 | 13.4457 | *BBX, CD47, IFT57, HHLA2, Myh6* |
| 1 | 117651438 | 117937631 | 8.3175 | *BBX, CD47, IFT57, HHLA2, Myh6* |
| 1 | 117664881 | 117850863 | 9.0534 | *BBX, CD47, IFT57* |
| 1 | 117705436 | 117858231 | 10.6386 | *BBX, CD47, IFT57* |
| 1 | 117712827 | 117974846 | 8.9660 | *BBX, CD47, IFT57, HHLA2, Myh6, RCJMB04_1n3, SH2D1B* |
| 1 | 117729102 | 117819143 | 8.5843 | *BBX, CD47* |
| 1 | 117840819 | 118140900 | 11.0456 | *IFT57, HHLA2, Myh6, RCJMB04_1n3, SH2D1B, chr38.1750, TRAT1, Hjurp, GUCA1A* |
| 1 | 117847884 | 118131517 | 8.3783 | *IFT57, HHLA2, Myh6, RCJMB04_1n3, SH2D1B, chr38.1750, TRAT1, Hjurp, GUCA1A* |
| 1 | 117867802 | 118180506 | 9.4728 | *HHLA2, Myh6, RCJMB04_1n3, SH2D1B, chr38.1750, TRAT1, Hjurp, GUCA1A* |
| 1 | 117879751 | 118112960 | 9.6899 | *Myh6, RCJMB04_1n3, SH2D1B, chr38.1750, TRAT1, Hjurp, GUCA1A* |
| 1 | 117908321 | 118203458 | 8.6053 | *Myh6, RCJMB04_1n3, SH2D1B, chr38.1750, TRAT1, Hjurp, GUCA1A, C3orf85* |
| 1 | 117937631 | 118147984 | 8.4464 | *RCJMB04_1n3, SH2D1B, chr38.1750, TRAT1, Hjurp, GUCA1A* |
| 1 | 118036389 | 118269490 | 15.8248 | *TRAT1, Hjurp, GUCA1A, C3orf85* |
| 1 | 118040708 | 118263203 | 11.7673 | *TRAT1, Hjurp, GUCA1A, C3orf85* |
| 1 | 123245481 | 123477768 | 7.9878 | *POU1F1, CHMP2B* |
| 1 | 123261738 | 123500111 | 8.2219 | *POU1F1, CHMP2B* |
| 1 | 123265489 | 123469828 | 13.7166 | *POU1F1, CHMP2B* |
| 1 | 123269924 | 123469828 | 12.2148 | *POU1F1, CHMP2B* |
| 1 | 123276722 | 123464166 | 11.3197 | *POU1F1, CHMP2B* |
| 1 | 125477692 | 125807361 | 21.2498 | *chr38.1846, Gbe1* |
| 1 | 125484309 | 125804084 | 18.3565 | *Gbe1* |
| 1 | 137529075 | 137752629 | 11.2342 | *ZC3HAV1, ETV6* |
| 1 | 137531799 | 137696219 | 8.9901 | *ZC3HAV1, ETV6* |
| 1 | 137531799 | 137706001 | 9.1102 | *ZC3HAV1, ETV6* |
| 1 | 150041420 | 150313861 | 8.8298 | *IGF1, TH, chr38.2170, Pah, ASCL1* |
| 1 | 150048838 | 150309350 | 10.3057 | *IGF1, TH, chr38.2170, Pah, ASCL1* |
| 1 | 150572588 | 150809564 | 8.6437 | *NT5DC3, chr38.2180, HSP90B1, EPS8, TDG, GLT8D2, HCFC2, NFYB, chr38.2187, chr38.2188, TXNRD1, CHST11* |
| 1 | 150573494 | 150802035 | 8.1382 | *NT5DC3, chr38.2180, HSP90B1, EPS8, TDG, GLT8D2, HCFC2, NFYB, chr38.2187, chr38.2188, TXNRD1, CHST11* |
| 1 | 151730663 | 151855178 | 9.7038 | *RIC8B, TMEM263, MTERF2, CRY1* |
| 1 | 151736300 | 151875184 | 17.3909 | *TMEM263, MTERF2, CRY1* |
| 1 | 151737063 | 151852374 | 10.8788 | *TMEM263, MTERF2, CRY1* |
| 1 | 151737814 | 151833651 | 7.8850 | *TMEM263, MTERF2, CRY1* |
| 1 | 154754883 | 155038706 | 13.7895 | *SYNGR1, Tab1, MGAT3, chr38.2333, chr38.2334, MIEF1, Mief1, ATF4, RPS19BP1, Cacna1i* |
| 1 | 154777716 | 155077692 | 10.1207 | *SYNGR1, Tab1, MGAT3, chr38.2333, chr38.2334, MIEF1, Mief1, ATF4, RPS19BP1, Cacna1i* |
| 1 | 154781589 | 155075648 | 8.0864 | *SYNGR1, Tab1, MGAT3, chr38.2333, chr38.2334, MIEF1, Mief1, ATF4, RPS19BP1, Cacna1i* |
| 1 | 154788699 | 155003008 | 7.7019 | *Tab1, MGAT3, chr38.2333, chr38.2334, MIEF1, Mief1, ATF4, RPS19BP1, Cacna1i* |
| 1 | 154880303 | 154959607 | 8.9225 | *Cacna1i* |
| 1 | 161032710 | 161429945 | 8.0693 | *NUDT4, NUDT4B, UBE2N, MRPL42, SOCS2, chr38.2412, CRADD, PLXNC1* |
| 1 | 161036209 | 161424128 | 8.1508 | *NUDT4, NUDT4B, UBE2N, MRPL42, SOCS2, chr38.2412, CRADD, PLXNC1* |
| 1 | 161095735 | 161389693 | 7.7053 | *NUDT4, NUDT4B, UBE2N, MRPL42, SOCS2, chr38.2412, CRADD, PLXNC1* |
| 1 | 161110498 | 161385159 | 9.4633 | *NUDT4B, UBE2N, MRPL42, SOCS2, chr38.2412, CRADD, PLXNC1* |
| 1 | 180930703 | 181514270 | 12.8793 | *PPP1R3A, fd96Cb, FOXP2* |
| 1 | 180931210 | 181511901 | 14.7964 | *PPP1R3A, fd96Cb, FOXP2* |
| 1 | 180934191 | 181512815 | 10.9682 | *PPP1R3A, fd96Cb, FOXP2* |
| 1 | 180935248 | 181505500 | 16.5269 | *PPP1R3A, fd96Cb, FOXP2* |
| 1 | 180938715 | 181509618 | 9.0426 | *PPP1R3A, fd96Cb, FOXP2* |
| 1 | 180942198 | 181497441 | 17.8044 | *PPP1R3A, fd96Cb, FOXP2* |
| 1 | 180952673 | 181484547 | 18.0637 | *PPP1R3A, fd96Cb, FOXP2* |
| 1 | 180983540 | 181451465 | 16.4612 | *PPP1R3A, fd96Cb, FOXP2* |
| 1 | 181056592 | 181377025 | 13.3484 | *FOXP2* |
| 1 | 194455722 | 195810292 | 10.8574 | *PRKAR2B, PIK3CG, CCDC71L, NAMPT, CDHR3, ATXN7L1, Efcab10, RINT1, PUS7, SRPK2, KMT2E, LHFPL3, Orc5, RELN* |
| 1 | 194463776 | 195794970 | 7.7868 | *PRKAR2B, PIK3CG, CCDC71L, NAMPT, CDHR3, ATXN7L1, Efcab10, RINT1, PUS7, SRPK2, KMT2E, LHFPL3, Orc5, RELN* |
| 1 | 194466365 | 195794970 | 9.4154 | *PRKAR2B, PIK3CG, CCDC71L, NAMPT, CDHR3, ATXN7L1, Efcab10, RINT1, PUS7, SRPK2, KMT2E, LHFPL3, Orc5, RELN* |
| 1 | 194466614 | 195802414 | 12.1448 | *PRKAR2B, PIK3CG, CCDC71L, NAMPT, CDHR3, ATXN7L1, Efcab10, RINT1, PUS7, SRPK2, KMT2E, LHFPL3, Orc5, RELN* |
| 1 | 194475991 | 195810292 | 27.1429 | *PRKAR2B, PIK3CG, CCDC71L, NAMPT, CDHR3, ATXN7L1, Efcab10, RINT1, PUS7, SRPK2, KMT2E, LHFPL3, Orc5, RELN* |
| 1 | 194483965 | 195794970 | 18.0748 | *PRKAR2B, PIK3CG, CCDC71L, NAMPT, CDHR3, ATXN7L1, Efcab10, RINT1, PUS7, SRPK2, KMT2E, LHFPL3, Orc5, RELN* |
| 1 | 194486682 | 195794970 | 20.6973 | *PRKAR2B, PIK3CG, CCDC71L, NAMPT, CDHR3, ATXN7L1, Efcab10, RINT1, PUS7, SRPK2, KMT2E, LHFPL3, Orc5, RELN* |
| 1 | 194488572 | 195781165 | 13.0347 | *PRKAR2B, PIK3CG, CCDC71L, NAMPT, CDHR3, ATXN7L1, Efcab10, RINT1, PUS7, SRPK2, KMT2E, LHFPL3, Orc5, RELN* |
| 1 | 194493346 | 195781165 | 11.2375 | *PRKAR2B, PIK3CG, CCDC71L, NAMPT, CDHR3, ATXN7L1, Efcab10, RINT1, PUS7, SRPK2, KMT2E, LHFPL3, Orc5, RELN* |
| 1 | 194493346 | 195794970 | 30.9857 | *PRKAR2B, PIK3CG, CCDC71L, NAMPT, CDHR3, ATXN7L1, Efcab10, RINT1, PUS7, SRPK2, KMT2E, LHFPL3, Orc5, RELN* |
| 1 | 194494860 | 195781165 | 15.7940 | *PRKAR2B, PIK3CG, CCDC71L, NAMPT, CDHR3, ATXN7L1, Efcab10, RINT1, PUS7, SRPK2, KMT2E, LHFPL3, Orc5, RELN* |
| 1 | 194499183 | 195781165 | 15.3359 | *PRKAR2B, PIK3CG, CCDC71L, NAMPT, CDHR3, ATXN7L1, Efcab10, RINT1, PUS7, SRPK2, KMT2E, LHFPL3, Orc5, RELN* |
| 1 | 194507647 | 195766386 | 13.1707 | *PRKAR2B, PIK3CG, CCDC71L, NAMPT, CDHR3, ATXN7L1, Efcab10, RINT1, PUS7, SRPK2, KMT2E, LHFPL3, Orc5, RELN* |
| 1 | 194510317 | 195777622 | 33.5245 | *PIK3CG, CCDC71L, NAMPT, CDHR3, ATXN7L1, Efcab10, RINT1, PUS7, SRPK2, KMT2E, LHFPL3, Orc5, RELN* |
| 1 | 194512469 | 195777622 | 35.2275 | *PIK3CG, CCDC71L, NAMPT, CDHR3, ATXN7L1, Efcab10, RINT1, PUS7, SRPK2, KMT2E, LHFPL3, Orc5, RELN* |
| 1 | 194529843 | 195765093 | 35.9103 | *PIK3CG, CCDC71L, NAMPT, CDHR3, ATXN7L1, Efcab10, RINT1, PUS7, SRPK2, KMT2E, LHFPL3, Orc5, RELN* |
| 1 | 194558149 | 195735657 | 35.1868 | *PIK3CG, CCDC71L, NAMPT, CDHR3, ATXN7L1, Efcab10, RINT1, PUS7, SRPK2, KMT2E, LHFPL3, Orc5* |
| 1 | 194587223 | 195711192 | 30.9325 | *CCDC71L, NAMPT, CDHR3, ATXN7L1, Efcab10, RINT1, PUS7, SRPK2, KMT2E, LHFPL3, Orc5* |
| 1 | 194609866 | 195690625 | 31.4120 | *NAMPT, CDHR3, ATXN7L1, Efcab10, RINT1, PUS7, SRPK2, KMT2E, LHFPL3, Orc5* |
| 1 | 194653728 | 195649133 | 35.5805 | *NAMPT, CDHR3, ATXN7L1, Efcab10, RINT1, PUS7, SRPK2, KMT2E, KMT2E, LHFPL3* |
| 1 | 194685011 | 195620739 | 35.7419 | *NAMPT, CDHR3, ATXN7L1, Efcab10, RINT1, PUS7, SRPK2, KMT2E, LHFPL3* |
| 1 | 194718904 | 195587634 | 33.1399 | *NAMPT, CDHR3, ATXN7L1, Efcab10, RINT1, PUS7, SRPK2, KMT2E, LHFPL3* |
| 1 | 194719968 | 195711192 | 43.2758 | *NAMPT, CDHR3, ATXN7L1, Efcab10, RINT1, PUS7, SRPK2, KMT2E, LHFPL3, Orc5* |
| 1 | 194722493 | 195711192 | 38.1692 | *NAMPT, CDHR3, ATXN7L1, Efcab10, RINT1, PUS7, SRPK2, KMT2E, LHFPL3, Orc5* |
| 1 | 194726457 | 195710559 | 28.6401 | *NAMPT, CDHR3, ATXN7L1, Efcab10, RINT1, PUS7, SRPK2, KMT2E, LHFPL3, Orc5* |
| 1 | 194728794 | 195701654 | 45.1332 | *NAMPT, CDHR3, ATXN7L1, Efcab10, RINT1, PUS7, SRPK2, KMT2E, LHFPL3, Orc5* |
| 1 | 194741896 | 195687069 | 44.1154 | *NAMPT, CDHR3, ATXN7L1, Efcab10, RINT1, PUS7, SRPK2, KMT2E, LHFPL3, Orc5* |
| 1 | 194766725 | 195660329 | 39.6670 | *CDHR3, ATXN7L1, Efcab10, RINT1, PUS7, SRPK2, KMT2E, LHFPL3* |
| 1 | 194771974 | 195534827 | 26.5994 | *CDHR3, ATXN7L1, Efcab10, RINT1, PUS7, SRPK2, KMT2E, LHFPL3* |
| 1 | 194806565 | 195620568 | 30.5663 | *CDHR3, ATXN7L1, Efcab10, RINT1, PUS7, SRPK2, KMT2E, LHFPL3* |
| 1 | 194837433 | 195473555 | 11.9102 | *CDHR3, ATXN7L1, Efcab10, RINT1, PUS7, SRPK2, KMT2E, LHFPL3* |
| 1 | 194872099 | 195551132 | 9.9125 | *ATXN7L1, Efcab10, RINT1, PUS7, SRPK2, KMT2E, LHFPL3* |
| 1 | 195108842 | 195211316 | 10.1044 | *SRPK2, KMT2E* |
| 1 | 195108842 | 195391886 | 8.5567 | *SRPK2, KMT2E* |
| 1 | 195123979 | 195368869 | 8.4318 | *SRPK2, KMT2E* |
| 1 | 195123979 | 195371681 | 7.8875 | *SRPK2, KMT2E* |
| 1 | 195170151 | 195300569 | 8.4904 | *SRPK2, KMT2E* |
| 2 | 18304275 | 18556460 | 11.6548 | *Sybu, EBAG9, NUDCD1, TRHR* |
| 2 | 18319677 | 18534780 | 7.6767 | *Sybu, EBAG9, NUDCD1, TRHR* |
| 2 | 26549657 | 26703657 | 8.5421 | *OSGIN2, slc7a6, Ripk2* |
| 2 | 33409680 | 33818850 | 24.3405 | *LY96, TMEM70, Eloc, ube2w, Stau2, RDH10* |
| 2 | 33415306 | 33811275 | 23.5482 | *LY96, TMEM70, Eloc, ube2w, Stau2, RDH10* |
| 2 | 33416216 | 33814063 | 15.0361 | *LY96, TMEM70, Eloc, ube2w, Stau2, RDH10* |
| 2 | 33451858 | 33754518 | 8.6383 | *LY96, TMEM70, Eloc, ube2w, Stau2, RDH10* |
| 2 | 33454896 | 33770207 | 12.8790 | *LY96, TMEM70, Eloc, ube2w, Stau2, RDH10* |
| 2 | 33458843 | 33775386 | 12.0277 | *LY96, TMEM70, Eloc, ube2w, Stau2, RDH10* |
| 2 | 33478552 | 33771954 | 15.8427 | *Eloc, ube2w, Stau2, RDH10* |
| 2 | 33494764 | 33753584 | 7.8739 | *ube2w, Stau2, RDH10* |
| 2 | 33546230 | 33716974 | 8.4050 | *Stau2* |
| 2 | 33850119 | 34413869 | 9.5044 | *SBSPON, TERF1, KCNB2, TRPA1, MSC* |
| 2 | 33850119 | 34414318 | 10.1448 | *SBSPON, TERF1, KCNB2, TRPA1, MSC* |
| 2 | 33851547 | 34408427 | 8.7953 | *SBSPON, TERF1, KCNB2, TRPA1, MSC* |
| 2 | 33852980 | 34413345 | 10.6113 | *SBSPON, TERF1, KCNB2, TRPA1, MSC* |
| 2 | 33855000 | 34403083 | 8.0944 | *SBSPON, TERF1, KCNB2, TRPA1, MSC* |
| 2 | 33862032 | 34407607 | 8.0944 | *SBSPON, TERF1, KCNB2, TRPA1, MSC* |
| 2 | 33875778 | 34394535 | 9.6012 | *TERF1, KCNB2, TRPA1, MSC* |
| 2 | 34032088 | 34182396 | 7.9160 | *Kcnb2* |
| 2 | 34034662 | 34267436 | 11.2577 | *Kcnb2, TRPA1* |
| 2 | 34038363 | 34265866 | 10.2404 | *Kcnb2, TRPA1* |
| 2 | 34247658 | 34297211 | 8.6007 | *TRPA1* |
| 2 | 34557797 | 35632258 | 7.7846 | *EYA1, lactb2, tram1l1, NCOA2, PRDM14, SLCO5A1, SULF1* |
| 2 | 34569759 | 35618266 | 7.6411 | *EYA1, lactb2, tram1l1, NCOA2, PRDM14, SLCO5A1, SULF1* |
| 2 | 34578867 | 35613551 | 7.8492 | *EYA1, lactb2, tram1l1, NCOA2, PRDM14, SLCO5A1, SULF1* |
| 2 | 34584542 | 35609635 | 7.6500 | *EYA1, lactb2, tram1l1, NCOA2, PRDM14, SLCO5A1, SULF1* |
| 2 | 34707182 | 35506939 | 12.0803 | *lactb2, tram1l1, NCOA2, PRDM14, SLCO5A1, SULF1* |
| 2 | 34749716 | 35471303 | 14.4269 | *lactb2, tram1l1, NCOA2, PRDM14, SLCO5A1, SULF1* |
| 2 | 34838148 | 35380182 | 14.0500 | *lactb2, tram1l1, NCOA2, PRDM14, SLCO5A1, SULF1* |
| 2 | 34941692 | 35304653 | 13.9295 | *NCOA2, PRDM14, SLCO5A1, SULF1* |
| 2 | 34946693 | 35335557 | 17.4607 | *NCOA2, PRDM14, SLCO5A1, SULF1* |
| 2 | 34951571 | 35296036 | 17.9651 | *NCOA2, PRDM14, SLCO5A1, SULF1* |
| 2 | 34954986 | 35329295 | 18.5773 | *NCOA2, PRDM14, SLCO5A1, SULF1* |
| 2 | 34960009 | 35260366 | 13.1952 | *NCOA2, PRDM14, SLCO5A1* |
| 2 | 34960009 | 35320008 | 12.2651 | *NCOA2, PRDM14, SLCO5A1, SULF1* |
| 2 | 35029766 | 35237199 | 15.5582 | *NCOA2, PRDM14, SLCO5A1* |
| 2 | 35029766 | 35243086 | 15.5648 | *NCOA2, PRDM14, SLCO5A1* |
| 2 | 35029766 | 35244496 | 10.6340 | *NCOA2, PRDM14, SLCO5A1* |
| 2 | 35029766 | 35279828 | 12.9559 | *NCOA2, PRDM14, SLCO5A1* |
| 2 | 35066829 | 35171120 | 8.2130 | *NCOA2, PRDM14* |
| 2 | 35066829 | 35171813 | 12.0657 | *NCOA2, PRDM14* |
| 2 | 35066829 | 35286561 | 11.0552 | *NCOA2, PRDM14, SLCO5A1* |
| 2 | 35066829 | 35292490 | 21.8415 | *NCOA2, PRDM14, SLCO5A1, SULF1* |
| 2 | 35066829 | 35298446 | 17.8586 | *NCOA2, PRDM14, SLCO5A1, SULF1* |
| 2 | 35103595 | 35240077 | 13.9510 | *PRDM14, SLCO5A1* |
| 2 | 35103595 | 35253931 | 14.8833 | *PRDM14, SLCO5A1* |
| 2 | 35103595 | 35279197 | 12.4998 | *PRDM14, SLCO5A1* |
| 2 | 35111399 | 35208833 | 9.8544 | *PRDM14, SLCO5A1* |
| 2 | 56216202 | 56276971 | 9.4570 | *LDLRAD4* |
| 2 | 58474637 | 58516773 | 8.5784 | *DSEL* |
| 2 | 66549713 | 67233740 | 18.9936 | *irx1-a, irx2* |
| 2 | 66556873 | 67224186 | 17.6560 | *irx1-a, irx2* |
| 2 | 66575742 | 67178626 | 17.0994 | *irx1-a* |
| 2 | 66580930 | 67178626 | 12.6712 | *irx1-a* |
| 2 | 66624380 | 67160442 | 12.3905 | *irx1-a* |
| 2 | 146476169 | 146580746 | 9.4542 | *CUL2, CREM, CCNY* |
| 2 | 146491467 | 146568419 | 9.0442 | *CUL2, CREM, CCNY* |
| 2 | 153139916 | 153427565 | 8.9183 | *ACTR3B, XRCC2* |
| 3 | 12613220 | 14660689 | 41.0025 | *FAM49A, chr36.282, MYCN, DDX1, NBAS, Lratd1, chr36.290, TRIB2* |
| 3 | 12615710 | 14664678 | 38.0667 | *FAM49A, chr36.282, MYCN, DDX1, NBAS, Lratd1, chr36.290, TRIB2* |
| 3 | 12618500 | 14664678 | 41.7913 | *FAM49A, chr36.282, MYCN, DDX1, NBAS, Lratd1, chr36.290, TRIB2* |
| 3 | 12625362 | 14652021 | 35.6615 | *FAM49A, chr36.282, MYCN, DDX1, NBAS, Lratd1, chr36.290, TRIB2* |
| 3 | 12626145 | 14649865 | 41.3862 | *FAM49A, chr36.282, MYCN, DDX1, NBAS, Lratd1, chr36.290, TRIB2* |
| 3 | 12628701 | 14643360 | 37.4963 | *FAM49A, chr36.282, MYCN, DDX1, NBAS, Lratd1, chr36.290, TRIB2* |
| 3 | 12631139 | 14653043 | 40.7438 | *FAM49A, chr36.282, MYCN, DDX1, NBAS, Lratd1, chr36.290, TRIB2* |
| 3 | 12657289 | 14627737 | 36.6302 | *FAM49A, chr36.282, MYCN, DDX1, NBAS, Lratd1, chr36.290* |
| 3 | 12659120 | 14609928 | 31.2589 | *FAM49A, chr36.282, MYCN, DDX1, NBAS, Lratd1* |
| 3 | 12694636 | 14572783 | 21.6171 | *FAM49A, chr36.282, MYCN, DDX1, NBAS, Lratd1* |
| 3 | 12711025 | 14577133 | 29.8359 | *FAM49A, chr36.282, MYCN, DDX1, NBAS, Lratd1* |
| 3 | 12819579 | 14907579 | 13.4272 | *MYCN, DDX1, NBAS, Lratd1, chr36.290, TRIB2* |
| 3 | 12824058 | 14903364 | 12.1117 | *MYCN, DDX1, NBAS, Lratd1, chr36.290, TRIB2* |
| 3 | 12826357 | 14903364 | 8.9605 | *MYCN, DDX1, NBAS, Lratd1, chr36.290, TRIB2* |
| 3 | 12846754 | 14876669 | 10.8901 | *MYCN, DDX1, NBAS, Lratd1, chr36.290, TRIB2* |
| 3 | 12847887 | 14877500 | 13.0387 | *MYCN, DDX1, NBAS, Lratd1, chr36.290, TRIB2* |
| 3 | 12967097 | 14324204 | 21.1318 | *MYCN, DDX1, NBAS, Lratd1* |
| 3 | 13080333 | 14211070 | 11.9610 | *MYCN, DDX1, NBAS, Lratd1* |
| 3 | 13387690 | 13783622 | 16.0966 | *NBAS* |
| 3 | 13431063 | 13742379 | 9.8162 | *NBAS* |
| 3 | 13471065 | 13858449 | 12.4666 | *NBAS, Lratd1* |
| 3 | 13489405 | 13842804 | 10.0835 | *NBAS* |
| 3 | 13489405 | 14450844 | 18.0302 | *NBAS, Lratd1* |
| 3 | 13493848 | 14445655 | 15.2588 | *NBAS, Lratd1* |
| 3 | 13499162 | 14436362 | 16.5698 | *NBAS, Lratd1* |
| 3 | 13520786 | 13997144 | 19.5384 | *NBAS, Lratd1* |
| 3 | 13522344 | 13997144 | 20.8801 | *NBAS, Lratd1* |
| 3 | 13535032 | 14412529 | 11.6863 | *NBAS, Lratd1* |
| 3 | 13548190 | 13961450 | 13.3840 | *NBAS, Lratd1* |
| 3 | 13549571 | 13961450 | 15.3111 | *NBAS, Lratd1* |
| 3 | 13553091 | 13997144 | 25.1880 | *NBAS, Lratd1* |
| 3 | 13560695 | 14388889 | 9.7791 | *NBAS, Lratd1* |
| 3 | 13569658 | 13979172 | 19.5587 | *Lratd1* |
| 3 | 13574853 | 14400425 | 10.6514 | *Lratd1* |
| 3 | 13582268 | 14394540 | 11.3554 | *Lratd1* |
| 3 | 13584070 | 14384216 | 8.8325 | *Lratd1* |
| 3 | 13602207 | 14377263 | 8.3954 | *Lratd1* |
| 3 | 13625133 | 13902789 | 7.7485 | *Lratd1* |
| 3 | 13648230 | 13881317 | 14.2018 | *Lratd1* |
| 3 | 13648617 | 14212902 | 7.6748 | *Lratd1* |
| 3 | 13659434 | 14204269 | 7.7962 | *Lratd1* |
| 3 | 13739194 | 14171973 | 16.2009 | *Lratd1* |
| 3 | 13748282 | 14161550 | 13.8054 | *Lratd1* |
| 3 | 13748800 | 14178415 | 9.0188 | *Lratd1* |
| 3 | 13766303 | 14159313 | 9.4733 | *Lratd1* |
| 3 | 13773236 | 14140158 | 8.6579 | *Lratd1* |
| 3 | 13812257 | 14075378 | 12.0879 | *Lratd1* |
| 3 | 13847572 | 14186433 | 7.8465 | *Lratd1* |
| 3 | 16633771 | 16751909 | 7.9134 | *mboat2* |
| 3 | 18518199 | 19156601 | 20.3471 | *COLEC11, Rps7, RNASEH1, ADI1, Trappc12* |
| 3 | 18543980 | 19131451 | 11.6121 | *COLEC11, Rps7, RNASEH1, ADI1, Trappc12* |
| 3 | 18598299 | 19123382 | 12.1998 | *COLEC11, Rps7, RNASEH1, ADI1, Trappc12* |
| 3 | 18609290 | 19060702 | 18.7732 | *COLEC11* |
| 3 | 18652589 | 19165008 | 9.4181 | *COLEC11, Rps7, RNASEH1, ADI1, Trappc12* |
| 3 | 18671862 | 19053758 | 12.8725 | *COLEC11* |
| 3 | 18675717 | 19144126 | 9.7907 | *COLEC11, Rps7, RNASEH1, ADI1, Trappc12* |
| 3 | 37151246 | 37882729 | 14.3760 | *Ripply2, CYB5R4, MRAP2, CEP162, TBX18, Hunk, Ltv1, NT5E, SNX14* |
| 3 | 37154136 | 37877106 | 13.8166 | *Ripply2, CYB5R4, MRAP2, CEP162, TBX18, Hunk, Ltv1, NT5E, SNX14* |
| 3 | 37154632 | 37880888 | 10.3224 | *Ripply2, CYB5R4, MRAP2, CEP162, TBX18, Hunk, Ltv1, NT5E, SNX14* |
| 3 | 37173258 | 37866384 | 9.0474 | *CYB5R4, MRAP2, CEP162, TBX18, Hunk, Ltv1, NT5E, SNX14* |
| 3 | 37177787 | 37852021 | 9.3161 | *CYB5R4, MRAP2, CEP162, TBX18, Hunk, Ltv1, NT5E, SNX14* |
| 3 | 37291722 | 37627455 | 11.1934 | *CEP162, TBX18* |
| 3 | 37292520 | 37625535 | 12.1157 | *CEP162, TBX18* |
| 3 | 37293683 | 37634881 | 10.3994 | *CEP162, TBX18* |
| 3 | 37332081 | 37623008 | 13.5646 | *TBX18* |
| 3 | 37351796 | 37630084 | 12.9624 | *TBX18* |
| 3 | 37408239 | 37551502 | 9.5203 | *TBX18* |
| 3 | 37903637 | 38079381 | 11.3128 | *SYNCRIP, , chr36.537* |
| 3 | 37934686 | 38083701 | 14.1566 | *chr36.537* |
| 3 | 37940588 | 38251236 | 14.5997 | *5HT1E, CGA* |
| 3 | 37969461 | 38252759 | 21.1862 | *5HT1E, CGA* |
| 3 | 37992911 | 38198631 | 8.2599 | *5HT1E* |
| 3 | 37998934 | 38220973 | 10.8836 | *5HT1E* |
| 3 | 38223190 | 38404957 | 10.1617 | *CGA, ZNF292, GJB7, SMIM8, QtsA-12155, CFAP206, SLC35A1, RARS2* |
| 3 | 38264529 | 38377530 | 10.7096 | *ZNF292, GJB7, SMIM8, QtsA-12155, CFAP206* |
| 3 | 43527453 | 43732337 | 8.6839 | *POU3F2, FBXL4* |
| 3 | 43534318 | 43747555 | 13.5054 | *POU3F2, FBXL4* |
| 3 | 43534813 | 43935418 | 15.7392 | *POU3F2, FBXL4, FAXC, COQ3, PNISR, USP45, CCNC, Prdm13* |
| 3 | 43544169 | 43937547 | 13.4668 | *FBXL4, FAXC, COQ3, PNISR, USP45, CCNC, Prdm13* |
| 3 | 43546467 | 43936301 | 15.6236 | *FBXL4, FAXC, COQ3, PNISR, USP45, CCNC, Prdm13* |
| 3 | 43548434 | 43919409 | 12.7042 | *FBXL4, FAXC, COQ3, PNISR, USP45, CCNC* |
| 3 | 43578514 | 43923431 | 17.9199 | *FBXL4, FAXC, COQ3, PNISR, USP45, CCNC* |
| 3 | 43607122 | 43896511 | 8.1087 | *FBXL4, FAXC, COQ3, PNISR, USP45* |
| 3 | 43624204 | 43830266 | 12.0232 | *FAXC, COQ3, PNISR* |
| 3 | 43624204 | 43918696 | 14.3233 | *FAXC, COQ3, PNISR, USP45, CCNC* |
| 3 | 43646334 | 43785536 | 8.4060 | *FAXC* |
| 3 | 43650095 | 43800604 | 8.4666 | *FAXC, COQ3* |
| 3 | 43664246 | 43895572 | 14.5473 | *FAXC, COQ3, PNISR, USP45* |
| 3 | 43682119 | 43804411 | 9.5148 | *FAXC, COQ3* |
| 3 | 43686434 | 43807696 | 9.0850 | *FAXC, COQ3* |
| 3 | 43696192 | 43843600 | 7.6522 | *FAXC, COQ3, PNISR, USP45* |
| 3 | 48205765 | 48634213 | 7.7710 | *SEC63, OSTM1, NR2E1, Snx3, AFG1L, FOXO3, chr36.647* |
| 3 | 48206357 | 48631962 | 8.5335 | *SEC63, OSTM1, NR2E1, Snx3, AFG1L, FOXO3, chr36.647* |
| 3 | 48207968 | 48628094 | 8.8393 | *SEC63, OSTM1, NR2E1, Snx3, AFG1L, FOXO3, chr36.647* |
| 3 | 48218717 | 48616199 | 8.4978 | *SEC63, OSTM1, NR2E1, Snx3, AFG1L, FOXO3, chr36.647* |
| 3 | 48282311 | 48391491 | 8.1186 | *OSTM1, NR2E1, Snx3, AFG1L* |
| 3 | 65395607 | 65720779 | 14.7097 | *ZDHHC14, TMEM242, ARID1B,* |
| 3 | 65398356 | 65607717 | 14.0967 | *ZDHHC14, TMEM242, ARID1B,* |
| 3 | 65410584 | 65703846 | 15.3756 | *ZDHHC14, TMEM242, ARID1B,* |
| 3 | 65459732 | 65511951 | 11.0146 | *TMEM242* |
| 3 | 65478333 | 65595940 | 13.0776 | *TMEM242, ARID1B* |
| 3 | 65520967 | 65609258 | 7.8866 | *ARID1B* |
| 3 | 66892442 | 68808150 | 14.4875 | *CNKSR3, IPCEF1, OPRM1, RGS17, MTRF1L, FBXO5, VIP, myct1, SYNE1, ESR1, CCDC170, ARMT1, RMND1, chr36.917, chr36.918, ZBTB2, chr36.920, chr36.921, AKAP12, MTHFD1L, PLEKHG1* |
| 3 | 66898257 | 68804559 | 15.8492 | *CNKSR3, IPCEF1, OPRM1, RGS17, MTRF1L, FBXO5, VIP, myct1, SYNE1, ESR1, CCDC170, ARMT1, RMND1, chr36.917, chr36.918, ZBTB2, chr36.920, chr36.921, AKAP12, MTHFD1L, PLEKHG1* |
| 3 | 66898257 | 68811999 | 14.8914 | *CNKSR3, IPCEF1, OPRM1, RGS17, MTRF1L, FBXO5, VIP, myct1, SYNE1, ESR1, CCDC170, ARMT1, RMND1, chr36.917, chr36.918, ZBTB2, chr36.920, chr36.921, AKAP12, MTHFD1L, PLEKHG1, IYD* |
| 3 | 66898257 | 68813045 | 15.9615 | *CNKSR3, IPCEF1, OPRM1, RGS17, MTRF1L, FBXO5, VIP, myct1, SYNE1, ESR1, CCDC170, ARMT1, RMND1, chr36.917, chr36.918, ZBTB2, chr36.920, chr36.921, AKAP12, MTHFD1L, PLEKHG1, IYD* |
| 3 | 66904415 | 68799922 | 16.4573 | *CNKSR3, IPCEF1, OPRM1, RGS17, MTRF1L, FBXO5, VIP, myct1, SYNE1, ESR1, CCDC170, ARMT1, RMND1, chr36.917, chr36.918, ZBTB2, chr36.920, chr36.921, AKAP12, MTHFD1L, PLEKHG1* |
| 3 | 66909703 | 68802851 | 12.9948 | *CNKSR3, IPCEF1, OPRM1, RGS17, MTRF1L, FBXO5, VIP, myct1, SYNE1, ESR1, CCDC170, ARMT1, RMND1, chr36.917, chr36.918, ZBTB2, chr36.920, chr36.921, AKAP12, MTHFD1L, PLEKHG1* |
| 3 | 66911509 | 68788267 | 11.8439 | *CNKSR3, IPCEF1, OPRM1, RGS17, MTRF1L, FBXO5, VIP, myct1, SYNE1, ESR1, CCDC170, ARMT1, RMND1, chr36.917, chr36.918, ZBTB2, chr36.920, chr36.921, AKAP12, MTHFD1L, PLEKHG1* |
| 3 | 66911509 | 68795729 | 16.4773 | *CNKSR3, IPCEF1, OPRM1, RGS17, MTRF1L, FBXO5, VIP, myct1, SYNE1, ESR1, CCDC170, ARMT1, RMND1, chr36.917, chr36.918, ZBTB2, chr36.920, chr36.921, AKAP12, MTHFD1L, PLEKHG1* |
| 3 | 66919120 | 68774953 | 11.0499 | *CNKSR3, IPCEF1, OPRM1, RGS17, MTRF1L, FBXO5, VIP, myct1, SYNE1, ESR1, CCDC170, ARMT1, RMND1, chr36.917, chr36.918, ZBTB2, chr36.920, chr36.921, AKAP12, MTHFD1L, PLEKHG1* |
| 3 | 66925401 | 68789388 | 9.6843 | *CNKSR3, IPCEF1, OPRM1, RGS17, MTRF1L, FBXO5, VIP, myct1, SYNE1, ESR1, CCDC170, ARMT1, RMND1, chr36.917, chr36.918, ZBTB2, chr36.920, chr36.921, AKAP12, MTHFD1L, PLEKHG1* |
| 3 | 66925633 | 68764422 | 12.9603 | *CNKSR3, IPCEF1, OPRM1, RGS17, MTRF1L, FBXO5, VIP, myct1, SYNE1, ESR1, CCDC170, ARMT1, RMND1, chr36.917, chr36.918, ZBTB2, chr36.920, chr36.921, AKAP12, MTHFD1L, PLEKHG1* |
| 3 | 66930043 | 68757433 | 13.4834 | *IPCEF1, OPRM1, RGS17, MTRF1L, FBXO5, VIP, myct1, SYNE1, ESR1, CCDC170, ARMT1, RMND1, chr36.917, chr36.918, ZBTB2, chr36.920, chr36.921, AKAP12, MTHFD1L, PLEKHG1* |
| 3 | 66968334 | 68718150 | 13.1086 | *IPCEF1, OPRM1, RGS17, MTRF1L, FBXO5, VIP, myct1, SYNE1, ESR1, CCDC170, ARMT1, RMND1, chr36.917, chr36.918, ZBTB2, chr36.920, chr36.921, AKAP12, MTHFD1L, PLEKHG1* |
| 3 | 66978118 | 68749306 | 10.6039 | *IPCEF1, OPRM1, RGS17, MTRF1L, FBXO5, VIP, myct1, SYNE1, ESR1, CCDC170, ARMT1, RMND1, chr36.917, chr36.918, ZBTB2, chr36.920, chr36.921, AKAP12, MTHFD1L, PLEKHG1* |
| 3 | 66991862 | 68738406 | 13.4798 | *IPCEF1, OPRM1, RGS17, MTRF1L, FBXO5, VIP, myct1, SYNE1, ESR1, CCDC170, ARMT1, RMND1, chr36.917, chr36.918, ZBTB2, chr36.920, chr36.921, AKAP12, MTHFD1L, PLEKHG1* |
| 3 | 66995136 | 68690032 | 11.9682 | *IPCEF1, OPRM1, RGS17, MTRF1L, FBXO5, VIP, myct1, SYNE1, ESR1, CCDC170, ARMT1, RMND1, chr36.917, chr36.918, ZBTB2, chr36.920, chr36.921, AKAP12, MTHFD1L, PLEKHG1* |
| 3 | 67003790 | 68728769 | 15.2080 | *IPCEF1, OPRM1, RGS17, MTRF1L, FBXO5, VIP, myct1, SYNE1, ESR1, CCDC170, ARMT1, RMND1, chr36.917, chr36.918, ZBTB2, chr36.920, chr36.921, AKAP12, MTHFD1L, PLEKHG1* |
| 3 | 67018048 | 68716655 | 16.2305 | *OPRM1, RGS17, MTRF1L, FBXO5, VIP, myct1, SYNE1, ESR1, CCDC170, ARMT1, RMND1, chr36.917, chr36.918, ZBTB2, chr36.920, chr36.921, AKAP12, MTHFD1L, PLEKHG1* |
| 3 | 67036745 | 68700337 | 16.7498 | *OPRM1, RGS17, MTRF1L, FBXO5, VIP, myct1, SYNE1, ESR1, CCDC170, ARMT1, RMND1, chr36.917, chr36.918, ZBTB2, chr36.920, chr36.921, AKAP12, MTHFD1L, PLEKHG1* |
| 3 | 67051721 | 68686884 | 16.8573 | *OPRM1, RGS17, MTRF1L, FBXO5, VIP, myct1, SYNE1, ESR1, CCDC170, ARMT1, RMND1, chr36.917, chr36.918, ZBTB2, chr36.920, chr36.921, AKAP12, MTHFD1L, PLEKHG1* |
| 3 | 67054217 | 68629352 | 10.0233 | *RGS17, MTRF1L, FBXO5, VIP, myct1, SYNE1, ESR1, CCDC170, ARMT1, RMND1, chr36.917, chr36.918, ZBTB2, chr36.920, chr36.921, AKAP12, MTHFD1L, PLEKHG1* |
| 3 | 67068479 | 68672700 | 16.5782 | *RGS17, MTRF1L, FBXO5, VIP, myct1, SYNE1, ESR1, CCDC170, ARMT1, RMND1, chr36.917, chr36.918, ZBTB2, chr36.920, chr36.921, AKAP12, MTHFD1L, PLEKHG1* |
| 3 | 67088487 | 68657025 | 15.8730 | *RGS17, MTRF1L, FBXO5, VIP, myct1, SYNE1, ESR1, CCDC170, ARMT1, RMND1, chr36.917, chr36.918, ZBTB2, chr36.920, chr36.921, AKAP12, MTHFD1L, PLEKHG1* |
| 3 | 67105956 | 68638552 | 14.6627 | *RGS17, MTRF1L, FBXO5, VIP, myct1, SYNE1, ESR1, CCDC170, ARMT1, RMND1, chr36.917, chr36.918, ZBTB2, chr36.920, chr36.921, AKAP12, MTHFD1L, PLEKHG1* |
| 3 | 67131717 | 68622290 | 12.7009 | *RGS17, MTRF1L, FBXO5, VIP, myct1, SYNE1, ESR1, CCDC170, ARMT1, RMND1, chr36.917, chr36.918, ZBTB2, chr36.920, chr36.921, AKAP12, MTHFD1L, PLEKHG1* |
| 3 | 67190123 | 68558398 | 9.4537 | *RGS17, MTRF1L, FBXO5, VIP, myct1, SYNE1, ESR1, CCDC170, ARMT1, RMND1, chr36.917, chr36.918, ZBTB2, chr36.920, chr36.921, AKAP12, MTHFD1L* |
| 3 | 67260763 | 68211856 | 7.8677 | *RGS17, MTRF1L, FBXO5, VIP, myct1, SYNE1, ESR1* |
| 3 | 67267642 | 68346721 | 8.7228 | *RGS17, MTRF1L, FBXO5, VIP, myct1, SYNE1, ESR1, CCDC170, ARMT1, RMND1* |
| 3 | 67279745 | 68336520 | 8.3083 | *RGS17, MTRF1L, FBXO5, VIP, myct1, SYNE1, ESR1, CCDC170, ARMT1* |
| 3 | 67288306 | 68183191 | 8.0407 | *RGS17, MTRF1L, FBXO5, VIP, myct1, SYNE1, ESR1* |
| 3 | 67314744 | 68167849 | 8.0640 | *RGS17, MTRF1L, FBXO5, VIP, myct1, SYNE1, ESR1* |
| 3 | 67321297 | 68202273 | 17.7492 | *RGS17, MTRF1L, FBXO5, VIP, myct1, SYNE1, ESR1* |
| 3 | 67323495 | 68202273 | 17.7606 | *RGS17, MTRF1L, FBXO5, VIP, myct1, SYNE1, ESR1* |
| 3 | 67335677 | 68183191 | 16.9483 | *RGS17, MTRF1L, FBXO5, VIP, myct1, SYNE1, ESR1* |
| 3 | 67344547 | 68139373 | 7.8534 | *RGS17, MTRF1L, FBXO5, VIP, myct1, SYNE1, ESR1* |
| 3 | 67344547 | 68183191 | 16.9034 | *RGS17, MTRF1L, FBXO5, VIP, myct1, SYNE1, ESR1* |
| 3 | 67348678 | 68408194 | 9.2623 | *RGS17, MTRF1L, FBXO5, VIP, myct1, SYNE1, ESR1, CCDC170, ARMT1, RMND1, chr36.917, chr36.918, ZBTB2, chr36.920, chr36.921, AKAP12* |
| 3 | 67358205 | 68161191 | 15.3342 | *RGS17, MTRF1L, FBXO5, VIP, myct1, SYNE1, ESR1* |
| 3 | 67379192 | 68139373 | 14.9434 | *RGS17, MTRF1L, FBXO5, VIP, myct1, SYNE1, ESR1* |
| 3 | 67389746 | 68202273 | 10.4371 | *RGS17, MTRF1L, FBXO5, VIP, myct1, SYNE1, ESR1* |
| 3 | 67393091 | 68117846 | 12.6002 | *RGS17, MTRF1L, FBXO5, VIP, myct1, SYNE1, ESR1* |
| 3 | 67393091 | 68365896 | 9.4965 | *RGS17, MTRF1L, FBXO5, VIP, myct1, SYNE1, ESR1, CCDC170, ARMT1, RMND1, chr36.917, chr36.918, ZBTB2* |
| 3 | 67426726 | 68101506 | 11.1015 | *RGS17, MTRF1L, FBXO5, VIP, myct1, SYNE1, ESR1* |
| 3 | 67452065 | 68139373 | 10.3755 | *RGS17, MTRF1L, FBXO5, VIP, myct1, SYNE1, ESR1* |
| 3 | 67469853 | 67670517 | 7.6753 | *RGS17, MTRF1L, FBXO5* |
| 3 | 67521336 | 68039670 | 12.5325 | *RGS17, MTRF1L, FBXO5, VIP, myct1, SYNE1* |
| 3 | 67537507 | 68025323 | 12.1308 | *MTRF1L, FBXO5, VIP, myct1, SYNE1* |
| 3 | 67609751 | 67973072 | 7.9496 | *VIP, myct1, SYNE1* |
| 3 | 67629924 | 68139373 | 10.9137 | *VIP, myct1, SYNE1, ESR1* |
| 3 | 67647525 | 68117846 | 11.3598 | *VIP, myct1, SYNE1, ESR1* |
| 3 | 67654958 | 68139373 | 13.6516 | *VIP, myct1, SYNE1, ESR1* |
| 3 | 67654958 | 68151886 | 12.8166 | *VIP, myct1, SYNE1, ESR1* |
| 3 | 67670517 | 68128492 | 13.1999 | *VIP, myct1, SYNE1, ESR1* |
| 3 | 67670517 | 68139373 | 11.0564 | *VIP, myct1, SYNE1, ESR1* |
| 3 | 67694715 | 67897939 | 8.8451 | *myct1, SYNE1* |
| 3 | 67694715 | 68112811 | 8.1988 | *myct1, SYNE1, ESR1* |
| 3 | 67725261 | 68076902 | 10.7270 | *myct1, SYNE1, ESR1* |
| 3 | 67767556 | 68012837 | 7.6921 | *SYNE1* |
| 3 | 67779993 | 68000896 | 10.7428 | *SYNE1* |
| 3 | 71525336 | 71693162 | 13.8515 | *SF3B5, FNDC1* |
| 3 | 71526794 | 71689782 | 13.4475 | *SF3B5, FNDC1* |
| 3 | 71600725 | 71703666 | 9.5356 | *FNDC1* |
| 3 | 79395379 | 84737406 | 35.5511 | *SLC35F3, COA6, TARBP1, Irf2bp2, chr36.1092, chr36.1093, TOMM20, RBM34, ARID4B, GGPS1, tbce, B3GALNT2, GNG4, LYST, NID1, GPR137B, Ero1b, EDARADD, LGALS8, HEATR1, ACTN2, MTR, CHRM3, FMN2, GREM2, Rgs7, PIGM, chr36.1122, OPN3, WDR64, chr36.1125, EXO1, MAP1LC3C, LRIF1, PLD5, CEP170, Sdccag8, AKT3, ZBTB18, ADSS, DESI2, Cox20, HNRNPU, Efcab2, KIF26B, SMYD3, chr36.1143, TFB2M, CNST, Sccpdh, AHCTF1* |
| 3 | 79400715 | 84744093 | 43.1637 | *SLC35F3, COA6, TARBP1, Irf2bp2, chr36.1092, chr36.1093, TOMM20, RBM34, ARID4B, GGPS1, tbce, B3GALNT2, GNG4, LYST, NID1, GPR137B, Ero1b, EDARADD, LGALS8, HEATR1, ACTN2, MTR, CHRM3, FMN2, GREM2, Rgs7, PIGM, chr36.1122, OPN3, WDR64, chr36.1125, EXO1, MAP1LC3C, LRIF1, PLD5, CEP170, Sdccag8, AKT3, ZBTB18, ADSS, DESI2, Cox20, HNRNPU, Efcab2, KIF26B, SMYD3, chr36.1143, TFB2M, CNST, Sccpdh, AHCTF1* |
| 3 | 79401563 | 84732936 | 37.9665 | *SLC35F3, COA6, TARBP1, Irf2bp2, chr36.1092, chr36.1093, TOMM20, RBM34, ARID4B, GGPS1, tbce, B3GALNT2, GNG4, LYST, NID1, GPR137B, Ero1b, EDARADD, LGALS8, HEATR1, ACTN2, MTR, CHRM3, FMN2, GREM2, Rgs7, PIGM, chr36.1122, OPN3, WDR64, chr36.1125, EXO1, MAP1LC3C, LRIF1, PLD5, CEP170, Sdccag8, AKT3, ZBTB18, ADSS, DESI2, Cox20, HNRNPU, Efcab2, KIF26B, SMYD3, chr36.1143, TFB2M, CNST, Sccpdh, AHCTF1* |
| 3 | 79403658 | 84734795 | 41.0955 | *SLC35F3, COA6, TARBP1, Irf2bp2, chr36.1092, chr36.1093, TOMM20, RBM34, ARID4B, GGPS1, tbce, B3GALNT2, GNG4, LYST, NID1, GPR137B, Ero1b, EDARADD, LGALS8, HEATR1, ACTN2, MTR, CHRM3, FMN2, GREM2, Rgs7, PIGM, chr36.1122, OPN3, WDR64, chr36.1125, EXO1, MAP1LC3C, LRIF1, PLD5, CEP170, Sdccag8, AKT3, ZBTB18, ADSS, DESI2, Cox20, HNRNPU, Efcab2, KIF26B, SMYD3, chr36.1143, TFB2M, CNST, Sccpdh, AHCTF1* |
| 3 | 79405870 | 84730774 | 39.7622 | *SLC35F3, COA6, TARBP1, Irf2bp2, chr36.1092, chr36.1093, TOMM20, RBM34, ARID4B, GGPS1, tbce, B3GALNT2, GNG4, LYST, NID1, GPR137B, Ero1b, EDARADD, LGALS8, HEATR1, ACTN2, MTR, CHRM3, FMN2, GREM2, Rgs7, PIGM, chr36.1122, OPN3, WDR64, chr36.1125, EXO1, MAP1LC3C, LRIF1, PLD5, CEP170, Sdccag8, AKT3, ZBTB18, ADSS, DESI2, Cox20, HNRNPU, Efcab2, KIF26B, SMYD3, chr36.1143, TFB2M, CNST, Sccpdh, AHCTF1* |
| 3 | 79405870 | 84735494 | 42.0813 | *SLC35F3, COA6, TARBP1, Irf2bp2, chr36.1092, chr36.1093, TOMM20, RBM34, ARID4B, GGPS1, tbce, B3GALNT2, GNG4, LYST, NID1, GPR137B, Ero1b, EDARADD, LGALS8, HEATR1, ACTN2, MTR, CHRM3, FMN2, GREM2, Rgs7, PIGM, chr36.1122, OPN3, WDR64, chr36.1125, EXO1, MAP1LC3C, LRIF1, PLD5, CEP170, Sdccag8, AKT3, ZBTB18, ADSS, DESI2, Cox20, HNRNPU, Efcab2, KIF26B, SMYD3, chr36.1143, TFB2M, CNST, Sccpdh, AHCTF1* |
| 3 | 79405870 | 84736971 | 42.7426 | *SLC35F3, COA6, TARBP1, Irf2bp2, chr36.1092, chr36.1093, TOMM20, RBM34, ARID4B, GGPS1, tbce, B3GALNT2, GNG4, LYST, NID1, GPR137B, Ero1b, EDARADD, LGALS8, HEATR1, ACTN2, MTR, CHRM3, FMN2, GREM2, Rgs7, PIGM, chr36.1122, OPN3, WDR64, chr36.1125, EXO1, MAP1LC3C, LRIF1, PLD5, CEP170, Sdccag8, AKT3, ZBTB18, ADSS, DESI2, Cox20, HNRNPU, Efcab2, KIF26B, SMYD3, chr36.1143, TFB2M, CNST, Sccpdh, AHCTF1* |
| 3 | 79406758 | 84738811 | 43.3435 | *SLC35F3, COA6, TARBP1, Irf2bp2, chr36.1092, chr36.1093, TOMM20, RBM34, ARID4B, GGPS1, tbce, B3GALNT2, GNG4, LYST, NID1, GPR137B, Ero1b, EDARADD, LGALS8, HEATR1, ACTN2, MTR, CHRM3, FMN2, GREM2, Rgs7, PIGM, chr36.1122, OPN3, WDR64, chr36.1125, EXO1, MAP1LC3C, LRIF1, PLD5, CEP170, Sdccag8, AKT3, ZBTB18, ADSS, DESI2, Cox20, HNRNPU, Efcab2, KIF26B, SMYD3, chr36.1143, TFB2M, CNST, Sccpdh, AHCTF1* |
| 3 | 79412720 | 84735494 | 43.2917 | *SLC35F3, COA6, TARBP1, Irf2bp2, chr36.1092, chr36.1093, TOMM20, RBM34, ARID4B, GGPS1, tbce, B3GALNT2, GNG4, LYST, NID1, GPR137B, Ero1b, EDARADD, LGALS8, HEATR1, ACTN2, MTR, CHRM3, FMN2, GREM2, Rgs7, PIGM, chr36.1122, OPN3, WDR64, chr36.1125, EXO1, MAP1LC3C, LRIF1, PLD5, CEP170, Sdccag8, AKT3, ZBTB18, ADSS, DESI2, Cox20, HNRNPU, Efcab2, KIF26B, SMYD3, chr36.1143, TFB2M, CNST, Sccpdh, AHCTF1* |
| 3 | 79415871 | 84734795 | 43.0556 | *chr36.1122, OPN3, WDR64, chr36.1125, EXO1, MAP1LC3C, LRIF1, PLD5, CEP170, Sdccag8, AKT3, ZBTB18, ADSS, DESI2, Cox20, HNRNPU, Efcab2, KIF26B, SMYD3, chr36.1143, TFB2M, CNST, Sccpdh, AHCTF1* |
| 3 | 79421884 | 84730774 | 42.6002 | *SLC35F3, COA6, TARBP1, Irf2bp2, chr36.1092, chr36.1093, TOMM20, RBM34, ARID4B, GGPS1, tbce, B3GALNT2, GNG4, LYST, NID1, GPR137B, Ero1b, EDARADD, LGALS8, HEATR1, ACTN2, MTR, CHRM3, FMN2, GREM2, Rgs7, PIGM, chr36.1122, OPN3, WDR64, chr36.1125, EXO1, MAP1LC3C, LRIF1, PLD5, CEP170, Sdccag8, AKT3, ZBTB18, ADSS, DESI2, Cox20, HNRNPU, Efcab2, KIF26B, SMYD3, chr36.1143, TFB2M, CNST, Sccpdh, AHCTF1* |
| 3 | 79427600 | 84727012 | 41.9678 | *SLC35F3, COA6, TARBP1, Irf2bp2, chr36.1092, chr36.1093, TOMM20, RBM34, ARID4B, GGPS1, tbce, B3GALNT2, GNG4, LYST, NID1, GPR137B, Ero1b, EDARADD, LGALS8, HEATR1, ACTN2, MTR, CHRM3, FMN2, GREM2, Rgs7, PIGM, chr36.1122, OPN3, WDR64, chr36.1125, EXO1, MAP1LC3C, LRIF1, PLD5, CEP170, Sdccag8, AKT3, ZBTB18, ADSS, DESI2, Cox20, HNRNPU, Efcab2, KIF26B, SMYD3, chr36.1143, TFB2M, CNST, Sccpdh, AHCTF1* |
| 3 | 79430414 | 84725812 | 41.1270 | *SLC35F3, COA6, TARBP1, Irf2bp2, chr36.1092, chr36.1093, TOMM20, RBM34, ARID4B, GGPS1, tbce, B3GALNT2, GNG4, LYST, NID1, GPR137B, Ero1b, EDARADD, LGALS8, HEATR1, ACTN2, MTR, CHRM3, FMN2, GREM2, Rgs7, PIGM, chr36.1122, OPN3, WDR64, chr36.1125, EXO1, MAP1LC3C, LRIF1, PLD5, CEP170, Sdccag8, AKT3, ZBTB18, ADSS, DESI2, Cox20, HNRNPU, Efcab2, KIF26B, SMYD3, chr36.1143, TFB2M, CNST, Sccpdh, AHCTF1* |
| 3 | 79434945 | 84723673 | 40.0582 | *SLC35F3, COA6, TARBP1, Irf2bp2, chr36.1092, chr36.1093, TOMM20, RBM34, ARID4B, GGPS1, tbce, B3GALNT2, GNG4, LYST, NID1, GPR137B, Ero1b, EDARADD, LGALS8, HEATR1, ACTN2, MTR, CHRM3, FMN2, GREM2, Rgs7, PIGM, chr36.1122, OPN3, WDR64, chr36.1125, EXO1, MAP1LC3C, LRIF1, PLD5, CEP170, Sdccag8, AKT3, ZBTB18, ADSS, DESI2, Cox20, HNRNPU, Efcab2, KIF26B, SMYD3, chr36.1143, TFB2M, CNST, Sccpdh, AHCTF1* |
| 3 | 79437452 | 84721811 | 38.7747 | *SLC35F3, COA6, TARBP1, Irf2bp2, chr36.1092, chr36.1093, TOMM20, RBM34, ARID4B, GGPS1, tbce, B3GALNT2, GNG4, LYST, NID1, GPR137B, Ero1b, EDARADD, LGALS8, HEATR1, ACTN2, MTR, CHRM3, FMN2, GREM2, Rgs7, PIGM, chr36.1122, OPN3, WDR64, chr36.1125, EXO1, MAP1LC3C, LRIF1, PLD5, CEP170, Sdccag8, AKT3, ZBTB18, ADSS, DESI2, Cox20, HNRNPU, Efcab2, KIF26B, SMYD3, chr36.1143, TFB2M, CNST, Sccpdh, AHCTF1* |
| 3 | 79438855 | 84690829 | 32.1270 | *SLC35F3, COA6, TARBP1, Irf2bp2, chr36.1092, chr36.1093, TOMM20, RBM34, ARID4B, GGPS1, tbce, B3GALNT2, GNG4, LYST, NID1, GPR137B, Ero1b, EDARADD, LGALS8, HEATR1, ACTN2, MTR, CHRM3, FMN2, GREM2, Rgs7, PIGM, chr36.1122, OPN3, WDR64, chr36.1125, EXO1, MAP1LC3C, LRIF1, PLD5, CEP170, Sdccag8, AKT3, ZBTB18, ADSS, DESI2, Cox20, HNRNPU, Efcab2, KIF26B, SMYD3, chr36.1143, TFB2M, CNST, Sccpdh, AHCTF1* |
| 3 | 79441922 | 84720523 | 37.2813 | *chr36.1122, OPN3, WDR64, chr36.1125, EXO1, MAP1LC3C, LRIF1, PLD5, CEP170, Sdccag8, AKT3, ZBTB18, ADSS, DESI2, Cox20, HNRNPU, Efcab2, KIF26B, SMYD3, chr36.1143, TFB2M, CNST, Sccpdh, AHCTF1* |
| 3 | 79452226 | 84676433 | 26.6062 | *SLC35F3, COA6, TARBP1, Irf2bp2, chr36.1092, chr36.1093, TOMM20, RBM34, ARID4B, GGPS1, tbce, B3GALNT2, GNG4, LYST, NID1, GPR137B, Ero1b, EDARADD, LGALS8, HEATR1, ACTN2, MTR, CHRM3, FMN2, GREM2, Rgs7, PIGM, chr36.1122, OPN3, WDR64, chr36.1125, EXO1, MAP1LC3C, LRIF1, PLD5, CEP170, Sdccag8, AKT3, ZBTB18, ADSS, DESI2, Cox20, HNRNPU, Efcab2, KIF26B, SMYD3, chr36.1143, TFB2M, CNST, Sccpdh, AHCTF1* |
| 3 | 79454190 | 84710396 | 35.5401 | *SLC35F3, COA6, TARBP1, Irf2bp2, chr36.1092, chr36.1093, TOMM20, RBM34, ARID4B, GGPS1, tbce, B3GALNT2, GNG4, LYST, NID1, GPR137B, Ero1b, EDARADD, LGALS8, HEATR1, ACTN2, MTR, CHRM3, FMN2, GREM2, Rgs7, PIGM, chr36.1122, OPN3, WDR64, chr36.1125, EXO1, MAP1LC3C, LRIF1, PLD5, CEP170, Sdccag8, AKT3, ZBTB18, ADSS, DESI2, Cox20, HNRNPU, Efcab2, KIF26B, SMYD3, chr36.1143, TFB2M, CNST, Sccpdh, AHCTF1* |
| 3 | 79464361 | 84703957 | 31.3259 | *SLC35F3, COA6, TARBP1, Irf2bp2, chr36.1092, chr36.1093, TOMM20, RBM34, ARID4B, GGPS1, tbce, B3GALNT2, GNG4, LYST, NID1, GPR137B, Ero1b, EDARADD, LGALS8, HEATR1, ACTN2, MTR, CHRM3, FMN2, GREM2, Rgs7, PIGM, chr36.1122, OPN3, WDR64, chr36.1125, EXO1, MAP1LC3C, LRIF1, PLD5, CEP170, Sdccag8, AKT3, ZBTB18, ADSS, DESI2, Cox20, HNRNPU, Efcab2, KIF26B, SMYD3, chr36.1143, TFB2M, CNST, Sccpdh, AHCTF1* |
| 3 | 79464537 | 84701380 | 33.5690 | *SLC35F3, COA6, TARBP1, Irf2bp2, chr36.1092, chr36.1093, TOMM20, RBM34, ARID4B, GGPS1, tbce, B3GALNT2, GNG4, LYST, NID1, GPR137B, Ero1b, EDARADD, LGALS8, HEATR1, ACTN2, MTR, CHRM3, FMN2, GREM2, Rgs7, PIGM, chr36.1122, OPN3, WDR64, chr36.1125, EXO1, MAP1LC3C, LRIF1, PLD5, CEP170, Sdccag8, AKT3, ZBTB18, ADSS, DESI2, Cox20, HNRNPU, Efcab2, KIF26B, SMYD3, chr36.1143, TFB2M, CNST, Sccpdh, AHCTF1* |
| 3 | 79465752 | 84660793 | 9.2123 | *SLC35F3, COA6, TARBP1, Irf2bp2, chr36.1092, chr36.1093, TOMM20, RBM34, ARID4B, GGPS1, tbce, B3GALNT2, GNG4, LYST, NID1, GPR137B, Ero1b, EDARADD, LGALS8, HEATR1, ACTN2, MTR, CHRM3, FMN2, GREM2, Rgs7, PIGM, chr36.1122, OPN3, WDR64, chr36.1125, EXO1, MAP1LC3C, LRIF1, PLD5, CEP170, Sdccag8, AKT3, ZBTB18, ADSS, DESI2, Cox20, HNRNPU, Efcab2, KIF26B, SMYD3, chr36.1143, TFB2M, CNST, Sccpdh, AHCTF1* |
| 3 | 79471567 | 84698218 | 28.8017 | *SLC35F3, COA6, TARBP1, Irf2bp2, chr36.1092, chr36.1093, TOMM20, RBM34, ARID4B, GGPS1, tbce, B3GALNT2, GNG4, LYST, NID1, GPR137B, Ero1b, EDARADD, LGALS8, HEATR1, ACTN2, MTR, CHRM3, FMN2, GREM2, Rgs7, PIGM, chr36.1122, OPN3, WDR64, chr36.1125, EXO1, MAP1LC3C, LRIF1, PLD5, CEP170, Sdccag8, AKT3, ZBTB18, ADSS, DESI2, Cox20, HNRNPU, Efcab2, KIF26B, SMYD3, chr36.1143, TFB2M, CNST, Sccpdh, AHCTF1* |
| 3 | 79477244 | 84837639 | 33.0371 | *SLC35F3, COA6, TARBP1, Irf2bp2, chr36.1092, chr36.1093, TOMM20, RBM34, ARID4B, GGPS1, tbce, B3GALNT2, GNG4, LYST, NID1, GPR137B, Ero1b, EDARADD, LGALS8, HEATR1, ACTN2, MTR, CHRM3, FMN2, GREM2, Rgs7, PIGM, chr36.1122, OPN3, WDR64, chr36.1125, EXO1, MAP1LC3C, LRIF1, PLD5, CEP170, Sdccag8, AKT3, ZBTB18, ADSS, DESI2, Cox20, HNRNPU, Efcab2, KIF26B, SMYD3, chr36.1143, TFB2M, CNST, Sccpdh, AHCTF1, SRD5A2, MEMO1* |
| 3 | 79478373 | 84846151 | 43.6755 | *SLC35F3, COA6, TARBP1, Irf2bp2, chr36.1092, chr36.1093, TOMM20, RBM34, ARID4B, GGPS1, tbce, B3GALNT2, GNG4, LYST, NID1, GPR137B, Ero1b, EDARADD, LGALS8, HEATR1, ACTN2, MTR, CHRM3, FMN2, GREM2, Rgs7, PIGM, chr36.1122, OPN3, WDR64, chr36.1125, EXO1, MAP1LC3C, LRIF1, PLD5, CEP170, Sdccag8, AKT3, ZBTB18, ADSS, DESI2, Cox20, HNRNPU, Efcab2, KIF26B, SMYD3, chr36.1143, TFB2M, CNST, Sccpdh, AHCTF1, SRD5A2, MEMO1* |
| 3 | 79479107 | 84833258 | 29.0520 | *SLC35F3, COA6, TARBP1, Irf2bp2, chr36.1092, chr36.1093, TOMM20, RBM34, ARID4B, GGPS1, tbce, B3GALNT2, GNG4, LYST, NID1, GPR137B, Ero1b, EDARADD, LGALS8, HEATR1, ACTN2, MTR, CHRM3, FMN2, GREM2, Rgs7, PIGM, chr36.1122, OPN3, WDR64, chr36.1125, EXO1, MAP1LC3C, LRIF1, PLD5, CEP170, Sdccag8, AKT3, ZBTB18, ADSS, DESI2, Cox20, HNRNPU, Efcab2, KIF26B, SMYD3, chr36.1143, TFB2M, CNST, Sccpdh, AHCTF1, SRD5A2, MEMO1* |
| 3 | 79480119 | 84644005 | 22.6544 | *SLC35F3, COA6, TARBP1, Irf2bp2, chr36.1092, chr36.1093, TOMM20, RBM34, ARID4B, GGPS1, tbce, B3GALNT2, GNG4, LYST, NID1, GPR137B, Ero1b, EDARADD, LGALS8, HEATR1, ACTN2, MTR, CHRM3, FMN2, GREM2, Rgs7, PIGM, chr36.1122, OPN3, WDR64, chr36.1125, EXO1, MAP1LC3C, LRIF1, PLD5, CEP170, Sdccag8, AKT3, ZBTB18, ADSS, DESI2, Cox20, HNRNPU, Efcab2, KIF26B, SMYD3, chr36.1143, TFB2M, CNST* |
| 3 | 79482735 | 84638031 | 29.5450 | *SLC35F3, COA6, TARBP1, Irf2bp2, chr36.1092, chr36.1093, TOMM20, RBM34, ARID4B, GGPS1, tbce, B3GALNT2, GNG4, LYST, NID1, GPR137B, Ero1b, EDARADD, LGALS8, HEATR1, ACTN2, MTR, CHRM3, FMN2, GREM2, Rgs7, PIGM, chr36.1122, OPN3, WDR64, chr36.1125, EXO1, MAP1LC3C, LRIF1, PLD5, CEP170, Sdccag8, AKT3, ZBTB18, ADSS, DESI2, Cox20, HNRNPU, Efcab2, KIF26B, SMYD3, chr36.1143, TFB2M, CNST* |
| 3 | 79482735 | 84834422 | 36.1816 | *SLC35F3, COA6, TARBP1, Irf2bp2, chr36.1092, chr36.1093, TOMM20, RBM34, ARID4B, GGPS1, tbce, B3GALNT2, GNG4, LYST, NID1, GPR137B, Ero1b, EDARADD, LGALS8, HEATR1, ACTN2, MTR, CHRM3, FMN2, GREM2, Rgs7, PIGM, chr36.1122, OPN3, WDR64, chr36.1125, EXO1, MAP1LC3C, LRIF1, PLD5, CEP170, Sdccag8, AKT3, ZBTB18, ADSS, DESI2, Cox20, HNRNPU, Efcab2, KIF26B, SMYD3, chr36.1143, TFB2M, CNST, Sccpdh, AHCTF1, SRD5A2, MEMO1* |
| 3 | 79482735 | 84835840 | 38.7407 | *SLC35F3, COA6, TARBP1, Irf2bp2, chr36.1092, chr36.1093, TOMM20, RBM34, ARID4B, GGPS1, tbce, B3GALNT2, GNG4, LYST, NID1, GPR137B, Ero1b, EDARADD, LGALS8, HEATR1, ACTN2, MTR, CHRM3, FMN2, GREM2, Rgs7, PIGM, chr36.1122, OPN3, WDR64, chr36.1125, EXO1, MAP1LC3C, LRIF1, PLD5, CEP170, Sdccag8, AKT3, ZBTB18, ADSS, DESI2, Cox20, HNRNPU, Efcab2, KIF26B, SMYD3, chr36.1143, TFB2M, CNST, Sccpdh, AHCTF1, SRD5A2, MEMO1* |
| 3 | 79483161 | 84835840 | 40.7631 | *SLC35F3, COA6, TARBP1, Irf2bp2, chr36.1092, chr36.1093, TOMM20, RBM34, ARID4B, GGPS1, tbce, B3GALNT2, GNG4, LYST, NID1, GPR137B, Ero1b, EDARADD, LGALS8, HEATR1, ACTN2, MTR, CHRM3, FMN2, GREM2, Rgs7, PIGM, chr36.1122, OPN3, WDR64, chr36.1125, EXO1, MAP1LC3C, LRIF1, PLD5, CEP170, Sdccag8, AKT3, ZBTB18, ADSS, DESI2, Cox20, HNRNPU, Efcab2, KIF26B, SMYD3, chr36.1143, TFB2M, CNST, Sccpdh, AHCTF1, SRD5A2, MEMO1* |
| 3 | 79485803 | 84824987 | 23.5935 | *SLC35F3, COA6, TARBP1, Irf2bp2, chr36.1092, chr36.1093, TOMM20, RBM34, ARID4B, GGPS1, tbce, B3GALNT2, GNG4, LYST, NID1, GPR137B, Ero1b, EDARADD, LGALS8, HEATR1, ACTN2, MTR, CHRM3, FMN2, GREM2, Rgs7, PIGM, chr36.1122, OPN3, WDR64, chr36.1125, EXO1, MAP1LC3C, LRIF1, PLD5, CEP170, Sdccag8, AKT3, ZBTB18, ADSS, DESI2, Cox20, HNRNPU, Efcab2, KIF26B, SMYD3, chr36.1143, TFB2M, CNST, Sccpdh, AHCTF1, SRD5A2* |
| 3 | 79487417 | 84839433 | 44.6542 | *SLC35F3, COA6, TARBP1, Irf2bp2, chr36.1092, chr36.1093, TOMM20, RBM34, ARID4B, GGPS1, tbce, B3GALNT2, GNG4, LYST, NID1, GPR137B, Ero1b, EDARADD, LGALS8, HEATR1, ACTN2, MTR, CHRM3, FMN2, GREM2, Rgs7, PIGM, chr36.1122, OPN3, WDR64, chr36.1125, EXO1, MAP1LC3C, LRIF1, PLD5, CEP170, Sdccag8, AKT3, ZBTB18, ADSS, DESI2, Cox20, HNRNPU, Efcab2, KIF26B, SMYD3, chr36.1143, TFB2M, CNST, Sccpdh, AHCTF1, SRD5A2, MEMO1* |
| 3 | 79487984 | 84816466 | 9.4161 | *SLC35F3, COA6, TARBP1, Irf2bp2, chr36.1092, chr36.1093, TOMM20, RBM34, ARID4B, GGPS1, tbce, B3GALNT2, GNG4, LYST, NID1, GPR137B, Ero1b, EDARADD, LGALS8, HEATR1, ACTN2, MTR, CHRM3, FMN2, GREM2, Rgs7, PIGM, chr36.1122, OPN3, WDR64, chr36.1125, EXO1, MAP1LC3C, LRIF1, PLD5, CEP170, Sdccag8, AKT3, ZBTB18, ADSS, DESI2, Cox20, HNRNPU, Efcab2, KIF26B, SMYD3, chr36.1143, TFB2M, CNST, Sccpdh, AHCTF1, SRD5A2* |
| 3 | 79488127 | 84834422 | 42.3877 | *SLC35F3, COA6, TARBP1, Irf2bp2, chr36.1092, chr36.1093, TOMM20, RBM34, ARID4B, GGPS1, tbce, B3GALNT2, GNG4, LYST, NID1, GPR137B, Ero1b, EDARADD, LGALS8, HEATR1, ACTN2, MTR, CHRM3, FMN2, GREM2, Rgs7, PIGM, chr36.1122, OPN3, WDR64, chr36.1125, EXO1, MAP1LC3C, LRIF1, PLD5, CEP170, Sdccag8, AKT3, ZBTB18, ADSS, DESI2, Cox20, HNRNPU, Efcab2, KIF26B, SMYD3, chr36.1143, TFB2M, CNST, Sccpdh, AHCTF1, SRD5A2, MEMO1* |
| 3 | 79488808 | 84633079 | 27.5828 | *SLC35F3, COA6, TARBP1, Irf2bp2, chr36.1092, chr36.1093, TOMM20, RBM34, ARID4B, GGPS1, tbce, B3GALNT2, GNG4, LYST, NID1, GPR137B, Ero1b, EDARADD, LGALS8, HEATR1, ACTN2, MTR, CHRM3, FMN2, GREM2, Rgs7, PIGM, chr36.1122, OPN3, WDR64, chr36.1125, EXO1, MAP1LC3C, LRIF1, PLD5, CEP170, Sdccag8, AKT3, ZBTB18, ADSS, DESI2, Cox20, HNRNPU, Efcab2, KIF26B, SMYD3, chr36.1143, TFB2M, CNST* |
| 3 | 79491920 | 84680289 | 25.9641 | *SLC35F3, COA6, TARBP1, Irf2bp2, chr36.1092, chr36.1093, TOMM20, RBM34, ARID4B, GGPS1, tbce, B3GALNT2, GNG4, LYST, NID1, GPR137B, Ero1b, EDARADD, LGALS8, HEATR1, ACTN2, MTR, CHRM3, FMN2, GREM2, Rgs7, PIGM, chr36.1122, OPN3, WDR64, chr36.1125, EXO1, MAP1LC3C, LRIF1, PLD5, CEP170, Sdccag8, AKT3, ZBTB18, ADSS, DESI2, Cox20, HNRNPU, Efcab2, KIF26B, SMYD3, chr36.1143, TFB2M, CNST, Sccpdh, AHCTF1* |
| 3 | 79493840 | 84834422 | 45.3266 | *SLC35F3, COA6, TARBP1, Irf2bp2, chr36.1092, chr36.1093, TOMM20, RBM34, ARID4B, GGPS1, tbce, B3GALNT2, GNG4, LYST, NID1, GPR137B, Ero1b, EDARADD, LGALS8, HEATR1, ACTN2, MTR, CHRM3, FMN2, GREM2, Rgs7, PIGM, chr36.1122, OPN3, WDR64, chr36.1125, EXO1, MAP1LC3C, LRIF1, PLD5, CEP170, Sdccag8, AKT3, ZBTB18, ADSS, DESI2, Cox20, HNRNPU, Efcab2, KIF26B, SMYD3, chr36.1143, TFB2M, CNST, Sccpdh, AHCTF1, SRD5A2, MEMO1* |
| 3 | 79494407 | 84814573 | 14.0832 | *SLC35F3, COA6, TARBP1, Irf2bp2, chr36.1092, chr36.1093, TOMM20, RBM34, ARID4B, GGPS1, tbce, B3GALNT2, GNG4, LYST, NID1, GPR137B, Ero1b, EDARADD, LGALS8, HEATR1, ACTN2, MTR, CHRM3, FMN2, GREM2, Rgs7, PIGM, chr36.1122, OPN3, WDR64, chr36.1125, EXO1, MAP1LC3C, LRIF1, PLD5, CEP170, Sdccag8, AKT3, ZBTB18, ADSS, DESI2, Cox20, HNRNPU, Efcab2, KIF26B, SMYD3, chr36.1143, TFB2M, CNST, Sccpdh, AHCTF1, SRD5A2* |
| 3 | 79495163 | 84811287 | 10.8046 | *SLC35F3, COA6, TARBP1, Irf2bp2, chr36.1092, chr36.1093, TOMM20, RBM34, ARID4B, GGPS1, tbce, B3GALNT2, GNG4, LYST, NID1, GPR137B, Ero1b, EDARADD, LGALS8, HEATR1, ACTN2, MTR, CHRM3, FMN2, GREM2, Rgs7, PIGM, chr36.1122, OPN3, WDR64, chr36.1125, EXO1, MAP1LC3C, LRIF1, PLD5, CEP170, Sdccag8, AKT3, ZBTB18, ADSS, DESI2, Cox20, HNRNPU, Efcab2, KIF26B, SMYD3, chr36.1143, TFB2M, CNST, Sccpdh, AHCTF1, SRD5A2* |
| 3 | 79496103 | 84835840 | 45.8329 | *SLC35F3, COA6, TARBP1, Irf2bp2, chr36.1092, chr36.1093, TOMM20, RBM34, ARID4B, GGPS1, tbce, B3GALNT2, GNG4, LYST, NID1, GPR137B, Ero1b, EDARADD, LGALS8, HEATR1, ACTN2, MTR, CHRM3, FMN2, GREM2, Rgs7, PIGM, chr36.1122, OPN3, WDR64, chr36.1125, EXO1, MAP1LC3C, LRIF1, PLD5, CEP170, Sdccag8, AKT3, ZBTB18, ADSS, DESI2, Cox20, HNRNPU, Efcab2, KIF26B, SMYD3, chr36.1143, TFB2M, CNST, Sccpdh, AHCTF1, SRD5A2, MEMO1* |
| 3 | 79499506 | 84834980 | 45.6667 | *SLC35F3, COA6, TARBP1, Irf2bp2, chr36.1092, chr36.1093, TOMM20, RBM34, ARID4B, GGPS1, tbce, B3GALNT2, GNG4, LYST, NID1, GPR137B, Ero1b, EDARADD, LGALS8, HEATR1, ACTN2, MTR, CHRM3, FMN2, GREM2, Rgs7, PIGM, chr36.1122, OPN3, WDR64, chr36.1125, EXO1, MAP1LC3C, LRIF1, PLD5, CEP170, Sdccag8, AKT3, ZBTB18, ADSS, DESI2, Cox20, HNRNPU, Efcab2, KIF26B, SMYD3, chr36.1143, TFB2M, CNST, Sccpdh, AHCTF1, SRD5A2, MEMO1* |
| 3 | 79502031 | 84835840 | 44.4311 | *SLC35F3, COA6, TARBP1, Irf2bp2, chr36.1092, chr36.1093, TOMM20, RBM34, ARID4B, GGPS1, tbce, B3GALNT2, GNG4, LYST, NID1, GPR137B, Ero1b, EDARADD, LGALS8, HEATR1, ACTN2, MTR, CHRM3, FMN2, GREM2, Rgs7, PIGM, chr36.1122, OPN3, WDR64, chr36.1125, EXO1, MAP1LC3C, LRIF1, PLD5, CEP170, Sdccag8, AKT3, ZBTB18, ADSS, DESI2, Cox20, HNRNPU, Efcab2, KIF26B, SMYD3, chr36.1143, TFB2M, CNST, Sccpdh, AHCTF1, SRD5A2, MEMO1* |
| 3 | 79502801 | 84833565 | 45.1685 | *SLC35F3, COA6, TARBP1, Irf2bp2, chr36.1092, chr36.1093, TOMM20, RBM34, ARID4B, GGPS1, tbce, B3GALNT2, GNG4, LYST, NID1, GPR137B, Ero1b, EDARADD, LGALS8, HEATR1, ACTN2, MTR, CHRM3, FMN2, GREM2, Rgs7, PIGM, chr36.1122, OPN3, WDR64, chr36.1125, EXO1, MAP1LC3C, LRIF1, PLD5, CEP170, Sdccag8, AKT3, ZBTB18, ADSS, DESI2, Cox20, HNRNPU, Efcab2, KIF26B, SMYD3, chr36.1143, TFB2M, CNST, Sccpdh, AHCTF1, SRD5A2, MEMO1* |
| 3 | 79504541 | 84835840 | 43.2990 | *SLC35F3, COA6, TARBP1, Irf2bp2, chr36.1092, chr36.1093, TOMM20, RBM34, ARID4B, GGPS1, tbce, B3GALNT2, GNG4, LYST, NID1, GPR137B, Ero1b, EDARADD, LGALS8, HEATR1, ACTN2, MTR, CHRM3, FMN2, GREM2, Rgs7, PIGM, chr36.1122, OPN3, WDR64, chr36.1125, EXO1, MAP1LC3C, LRIF1, PLD5, CEP170, Sdccag8, AKT3, ZBTB18, ADSS, DESI2, Cox20, HNRNPU, Efcab2, KIF26B, SMYD3, chr36.1143, TFB2M, CNST, Sccpdh, AHCTF1, SRD5A2, MEMO1* |
| 3 | 79505316 | 84612938 | 30.1870 | *SLC35F3, COA6, TARBP1, Irf2bp2, chr36.1092, chr36.1093, TOMM20, RBM34, ARID4B, GGPS1, tbce, B3GALNT2, GNG4, LYST, NID1, GPR137B, Ero1b, EDARADD, LGALS8, HEATR1, ACTN2, MTR, CHRM3, FMN2, GREM2, Rgs7, PIGM, chr36.1122, PN3, WDR64, chr36.1125, EXO1, MAP1LC3C, LRIF1, PLD5, CEP170, Sdccag8, AKT3, ZBTB18, ADSS, DESI2, Cox20, HNRNPU, Efcab2, KIF26B, SMYD3, chr36.1143, TFB2M, CNST* |
| 3 | 79507126 | 84607076 | 29.0022 | *SLC35F3, COA6, TARBP1, Irf2bp2, chr36.1092, chr36.1093, TOMM20, RBM34, ARID4B, GGPS1, tbce, B3GALNT2, GNG4, LYST, NID1, GPR137B, Ero1b, EDARADD, LGALS8, HEATR1, ACTN2, MTR, CHRM3, FMN2, GREM2, Rgs7, PIGM, chr36.1122, OPN3, WDR64, chr36.1125, EXO1, MAP1LC3C, LRIF1, PLD5, CEP170, Sdccag8, AKT3, ZBTB18, ADSS, DESI2, Cox20, HNRNPU, Efcab2, KIF26B, SMYD3, chr36.1143, TFB2M, CNST* |
| 3 | 79508503 | 84608039 | 29.9815 | *SLC35F3, COA6, TARBP1, Irf2bp2, chr36.1092, chr36.1093, TOMM20, RBM34, ARID4B, GGPS1, tbce, B3GALNT2, GNG4, LYST, NID1, GPR137B, Ero1b, EDARADD, LGALS8, HEATR1, ACTN2, MTR, CHRM3, FMN2, GREM2, Rgs7, PIGM, chr36.1122, OPN3, WDR64, chr36.1125, EXO1, MAP1LC3C, LRIF1, PLD5, CEP170, Sdccag8, AKT3, ZBTB18, ADSS, DESI2, Cox20, HNRNPU, Efcab2, KIF26B, SMYD3, chr36.1143, TFB2M, CNST* |
| 3 | 79513168 | 84598463 | 27.3285 | *SLC35F3, COA6, TARBP1, Irf2bp2, chr36.1092, chr36.1093, TOMM20, RBM34, ARID4B, GGPS1, tbce, B3GALNT2, GNG4, LYST, NID1, GPR137B, Ero1b, EDARADD, LGALS8, HEATR1, ACTN2, MTR, CHRM3, FMN2, GREM2, Rgs7, PIGM, chr36.1122, OPN3, WDR64, chr36.1125, EXO1, MAP1LC3C, LRIF1, PLD5, CEP170, Sdccag8, AKT3, ZBTB18, ADSS, DESI2, Cox20, HNRNPU, Efcab2, KIF26B, SMYD3, chr36.1143, TFB2M, CNST* |
| 3 | 79518078 | 84824657 | 41.7577 | *SLC35F3, COA6, TARBP1, Irf2bp2, chr36.1092, chr36.1093, TOMM20, RBM34, ARID4B, GGPS1, tbce, B3GALNT2, GNG4, LYST, NID1, GPR137B, Ero1b, EDARADD, LGALS8, HEATR1, ACTN2, MTR, CHRM3, FMN2, GREM2, Rgs7, PIGM, chr36.1122, OPN3, WDR64, chr36.1125, EXO1, MAP1LC3C, LRIF1, PLD5, CEP170, Sdccag8, AKT3, ZBTB18, ADSS, DESI2, Cox20, HNRNPU, Efcab2, KIF26B, SMYD3, chr36.1143, TFB2M, CNST, Sccpdh, AHCTF1, SRD5A2* |
| 3 | 79521155 | 84589885 | 24.8072 | *SLC35F3, COA6, TARBP1, Irf2bp2, chr36.1092, chr36.1093, TOMM20, RBM34, ARID4B, GGPS1, tbce, B3GALNT2, GNG4, LYST, NID1, GPR137B, Ero1b, EDARADD, LGALS8, HEATR1, ACTN2, MTR, CHRM3, FMN2, GREM2, Rgs7, PIGM, chr36.1122, OPN3, WDR64, chr36.1125, EXO1, MAP1LC3C, LRIF1, PLD5, CEP170, Sdccag8, AKT3, ZBTB18, ADSS, DESI2, Cox20, HNRNPU, Efcab2, KIF26B, SMYD3, chr36.1143, TFB2M, CNST* |
| 3 | 79521547 | 84587131 | 21.2089 | *SLC35F3, COA6, TARBP1, Irf2bp2, chr36.1092, chr36.1093, TOMM20, RBM34, ARID4B, GGPS1, tbce, B3GALNT2, GNG4, LYST, NID1, GPR137B, Ero1b, EDARADD, LGALS8, HEATR1, ACTN2, MTR, CHRM3, FMN2, GREM2, Rgs7, PIGM, chr36.1122, OPN3, WDR64, chr36.1125, EXO1, MAP1LC3C, LRIF1, PLD5, CEP170, Sdccag8, AKT3, ZBTB18, ADSS, DESI2, Cox20, HNRNPU, Efcab2, KIF26B, SMYD3, chr36.1143, TFB2M, CNST* |
| 3 | 79539615 | 84634946 | 22.8045 | *SLC35F3, COA6, TARBP1, Irf2bp2, chr36.1092, chr36.1093, TOMM20, RBM34, ARID4B, GGPS1, tbce, B3GALNT2, GNG4, LYST, NID1, GPR137B, Ero1b, EDARADD, LGALS8, HEATR1, ACTN2, MTR, CHRM3, FMN2, GREM2, Rgs7, PIGM, chr36.1122, OPN3, WDR64, chr36.1125, EXO1, MAP1LC3C, LRIF1, PLD5, CEP170, Sdccag8, AKT3, ZBTB18, ADSS, DESI2, Cox20, HNRNPU, Efcab2, KIF26B, SMYD3, chr36.1143, TFB2M, CNST* |
| 3 | 79540679 | 84565295 | 15.8888 | *SLC35F3, COA6, TARBP1, Irf2bp2, chr36.1092, chr36.1093, TOMM20, RBM34, ARID4B, GGPS1, tbce, B3GALNT2, GNG4, LYST, NID1, GPR137B, Ero1b, EDARADD, LGALS8, HEATR1, ACTN2, MTR, CHRM3, FMN2, GREM2, Rgs7, PIGM, chr36.1122, OPN3, WDR64, chr36.1125, EXO1, MAP1LC3C, LRIF1, PLD5, CEP170, Sdccag8, AKT3, ZBTB18, ADSS, DESI2, Cox20, HNRNPU, Efcab2, KIF26B, SMYD3, chr36.1143* |
| 3 | 79544926 | 84800006 | 39.7341 | *SLC35F3, COA6, TARBP1, Irf2bp2, chr36.1092, chr36.1093, TOMM20, RBM34, ARID4B, GGPS1, tbce, B3GALNT2, GNG4, LYST, NID1, GPR137B, Ero1b, EDARADD, LGALS8, HEATR1, ACTN2, MTR, CHRM3, FMN2, GREM2, Rgs7, PIGM, chr36.1122, OPN3, WDR64, chr36.1125, EXO1, MAP1LC3C, LRIF1, PLD5, CEP170, Sdccag8, AKT3, ZBTB18, ADSS, DESI2, Cox20, HNRNPU, Efcab2, KIF26B, SMYD3, chr36.1143, TFB2M, CNST, Sccpdh, AHCTF1, SRD5A2* |
| 3 | 79545530 | 84631348 | 19.3216 | *SLC35F3, COA6, TARBP1, Irf2bp2, chr36.1092, chr36.1093, TOMM20, RBM34, ARID4B, GGPS1, tbce, B3GALNT2, GNG4, LYST, NID1, GPR137B, Ero1b, EDARADD, LGALS8, HEATR1, ACTN2, MTR, CHRM3, FMN2, GREM2, Rgs7, PIGM, chr36.1122, OPN3, WDR64, chr36.1125, EXO1, MAP1LC3C, LRIF1, PLD5, CEP170, Sdccag8, AKT3, ZBTB18, ADSS, DESI2, Cox20, HNRNPU, Efcab2, KIF26B, SMYD3, chr36.1143, TFB2M, CNST* |
| 3 | 79552240 | 84794403 | 37.0786 | *SLC35F3, COA6, TARBP1, Irf2bp2, chr36.1092, chr36.1093, TOMM20, RBM34, ARID4B, GGPS1, tbce, B3GALNT2, GNG4, LYST, NID1, GPR137B, Ero1b, EDARADD, LGALS8, HEATR1, ACTN2, MTR, CHRM3, FMN2, GREM2, Rgs7, PIGM, chr36.1122, OPN3, WDR64, chr36.1125, EXO1, MAP1LC3C, LRIF1, PLD5, CEP170, Sdccag8, AKT3, ZBTB18, ADSS, DESI2, Cox20, HNRNPU, Efcab2, KIF26B, SMYD3, chr36.1143, TFB2M, CNST, Sccpdh, AHCTF1, SRD5A2* |
| 3 | 79553036 | 84795700 | 33.5161 | *SLC35F3, COA6, TARBP1, Irf2bp2, chr36.1092, chr36.1093, TOMM20, RBM34, ARID4B, GGPS1, tbce, B3GALNT2, GNG4, LYST, NID1, GPR137B, Ero1b, EDARADD, LGALS8, HEATR1, ACTN2, MTR, CHRM3, FMN2, GREM2, Rgs7, PIGM, chr36.1122, OPN3, WDR64, chr36.1125, EXO1, MAP1LC3C, LRIF1, PLD5, CEP170, Sdccag8, AKT3, ZBTB18, ADSS, DESI2, Cox20, HNRNPU, Efcab2, KIF26B, SMYD3, chr36.1143, TFB2M, CNST, Sccpdh, AHCTF1, SRD5A2* |
| 3 | 79562269 | 84616189 | 15.3842 | *SLC35F3, COA6, TARBP1, Irf2bp2, chr36.1092, chr36.1093, TOMM20, RBM34, ARID4B, GGPS1, tbce, B3GALNT2, GNG4, LYST, NID1, GPR137B, Ero1b, EDARADD, LGALS8, HEATR1, ACTN2, MTR, CHRM3, FMN2, GREM2, Rgs7, PIGM, chr36.1122, OPN3, WDR64, chr36.1125, EXO1, MAP1LC3C, LRIF1, PLD5, CEP170, Sdccag8, AKT3, ZBTB18, ADSS, DESI2, Cox20, HNRNPU, Efcab2, KIF26B, SMYD3, chr36.1143, TFB2M, CNST* |
| 3 | 79566561 | 84784237 | 28.4849 | *SLC35F3, COA6, TARBP1, Irf2bp2, chr36.1092, chr36.1093, TOMM20, RBM34, ARID4B, GGPS1, tbce, B3GALNT2, GNG4, LYST, NID1, GPR137B, Ero1b, EDARADD, LGALS8, HEATR1, ACTN2, MTR, CHRM3, FMN2, GREM2, Rgs7, PIGM, chr36.1122, OPN3, WDR64, chr36.1125, EXO1, MAP1LC3C, LRIF1, PLD5, CEP170, Sdccag8, AKT3, ZBTB18, ADSS, DESI2, Cox20, HNRNPU, Efcab2, KIF26B, SMYD3, chr36.1143, TFB2M, CNST, Sccpdh, AHCTF1, SRD5A2* |
| 3 | 79579397 | 84772836 | 20.3775 | *SLC35F3, COA6, TARBP1, Irf2bp2, chr36.1092, chr36.1093, TOMM20, RBM34, ARID4B, GGPS1, tbce, B3GALNT2, GNG4, LYST, NID1, GPR137B, Ero1b, EDARADD, LGALS8, HEATR1, ACTN2, MTR, CHRM3, FMN2, GREM2, Rgs7, PIGM, chr36.1122, OPN3, WDR64, chr36.1125, EXO1, MAP1LC3C, LRIF1, PLD5, CEP170, Sdccag8, AKT3, ZBTB18, ADSS, DESI2, Cox20, HNRNPU, Efcab2, KIF26B, SMYD3, chr36.1143, TFB2M, CNST, Sccpdh, AHCTF1, SRD5A2* |
| 3 | 79600523 | 84756368 | 10.3286 | *COA6, TARBP1, Irf2bp2, chr36.1092, chr36.1093, TOMM20, RBM34, ARID4B, GGPS1, tbce, B3GALNT2, GNG4, LYST, NID1, GPR137B, Ero1b, EDARADD, LGALS8, HEATR1, ACTN2, MTR, CHRM3, FMN2, GREM2, Rgs7, PIGM, chr36.1122, OPN3, WDR64, chr36.1125, EXO1, MAP1LC3C, LRIF1, PLD5, CEP170, Sdccag8, AKT3, ZBTB18, ADSS, DESI2, Cox20, HNRNPU, Efcab2, KIF26B, SMYD3, chr36.1143, TFB2M, CNST, Sccpdh, AHCTF1, SRD5A2* |
| 3 | 79602917 | 84755322 | 21.9130 | *COA6, TARBP1, Irf2bp2, chr36.1092, chr36.1093, TOMM20, RBM34, ARID4B, GGPS1, tbce, B3GALNT2, GNG4, LYST, NID1, GPR137B, Ero1b, EDARADD, LGALS8, HEATR1, ACTN2, MTR, CHRM3, FMN2, GREM2, Rgs7, PIGM, chr36.1122, OPN3, WDR64, chr36.1125, EXO1, MAP1LC3C, LRIF1, PLD5, CEP170, Sdccag8, AKT3, ZBTB18, ADSS, DESI2, Cox20, HNRNPU, Efcab2, KIF26B, SMYD3, chr36.1143, TFB2M, CNST, Sccpdh, AHCTF1* |
| 3 | 79606206 | 84574305 | 10.9878 | *TARBP1, Irf2bp2, chr36.1092, chr36.1093, TOMM20, RBM34, ARID4B, GGPS1, tbce, B3GALNT2, GNG4, LYST, NID1, GPR137B, Ero1b, EDARADD, LGALS8, HEATR1, ACTN2, MTR, CHRM3, FMN2, GREM2, Rgs7, PIGM, chr36.1122, OPN3, WDR64, chr36.1125, EXO1, MAP1LC3C, LRIF1, PLD5, CEP170, Sdccag8, AKT3, ZBTB18, ADSS, DESI2, Cox20, HNRNPU, Efcab2, KIF26B, SMYD3, chr36.1143, TFB2M* |
| 3 | 79621581 | 84738811 | 27.2777 | *TARBP1, Irf2bp2, chr36.1092, chr36.1093, TOMM20, RBM34, ARID4B, GGPS1, tbce, B3GALNT2, GNG4, LYST, NID1, GPR137B, Ero1b, EDARADD, LGALS8, HEATR1, ACTN2, MTR, CHRM3, FMN2, GREM2, Rgs7, PIGM, chr36.1122, OPN3, WDR64, chr36.1125, EXO1, MAP1LC3C, LRIF1, PLD5, CEP170, Sdccag8, AKT3, ZBTB18, ADSS, DESI2, Cox20, HNRNPU, Efcab2, KIF26B, SMYD3, chr36.1143, TFB2M, CNST, Sccpdh, AHCTF1* |
| 3 | 79626804 | 84460551 | 15.0041 | *TARBP1, Irf2bp2, chr36.1092, chr36.1093, TOMM20, RBM34, ARID4B, GGPS1, tbce, B3GALNT2, GNG4, LYST, NID1, GPR137B, Ero1b, EDARADD, LGALS8, HEATR1, ACTN2, MTR, CHRM3, FMN2, GREM2, Rgs7, PIGM, chr36.1122, OPN3, WDR64, chr36.1125, EXO1, MAP1LC3C, LRIF1, PLD5, CEP170, Sdccag8, AKT3, ZBTB18, ADSS, DESI2, Cox20, HNRNPU, Efcab2, KIF26B, SMYD3* |
| 3 | 79635642 | 84732936 | 30.3569 | *TARBP1, Irf2bp2, chr36.1092, chr36.1093, TOMM20, RBM34, ARID4B, GGPS1, tbce, B3GALNT2, GNG4, LYST, NID1, GPR137B, Ero1b, EDARADD, LGALS8, HEATR1, ACTN2, MTR, CHRM3, FMN2, GREM2, Rgs7, PIGM, chr36.1122, OPN3, WDR64, chr36.1125, EXO1, MAP1LC3C, LRIF1, PLD5, CEP170, Sdccag8, AKT3, ZBTB18, ADSS, DESI2, Cox20, HNRNPU, Efcab2, KIF26B, SMYD3, chr36.1143, TFB2M, CNST, Sccpdh, AHCTF1* |
| 3 | 79636196 | 84454233 | 20.0910 | *TARBP1, Irf2bp2, chr36.1092, chr36.1093, TOMM20, RBM34, ARID4B, GGPS1, tbce, B3GALNT2, GNG4, LYST, NID1, GPR137B, Ero1b, EDARADD, LGALS8, HEATR1, ACTN2, MTR, CHRM3, FMN2, GREM2, Rgs7, PIGM, chr36.1122, OPN3, WDR64, chr36.1125, EXO1, MAP1LC3C, LRIF1, PLD5, CEP170, Sdccag8, AKT3, ZBTB18, ADSS, DESI2, Cox20, HNRNPU, Efcab2, KIF26B, SMYD3* |
| 3 | 79645058 | 84719560 | 32.1957 | *TARBP1, Irf2bp2, chr36.1092, chr36.1093, TOMM20, RBM34, ARID4B, GGPS1, tbce, B3GALNT2, GNG4, LYST, NID1, GPR137B, Ero1b, EDARADD, LGALS8, HEATR1, ACTN2, MTR, CHRM3, FMN2, GREM2, Rgs7, PIGM, chr36.1122, OPN3, WDR64, chr36.1125, EXO1, MAP1LC3C, LRIF1, PLD5, CEP170, Sdccag8, AKT3, ZBTB18, ADSS, DESI2, Cox20, HNRNPU, Efcab2, KIF26B, SMYD3, chr36.1143, TFB2M, CNST, Sccpdh, AHCTF1* |
| 3 | 79645281 | 84441163 | 26.1603 | *TARBP1, Irf2bp2, chr36.1092, chr36.1093, TOMM20, RBM34, ARID4B, GGPS1, tbce, B3GALNT2, GNG4, LYST, NID1, GPR137B, Ero1b, EDARADD, LGALS8, HEATR1, ACTN2, MTR, CHRM3, FMN2, GREM2, Rgs7, PIGM, chr36.1122, OPN3, WDR64, chr36.1125, EXO1, MAP1LC3C, LRIF1, PLD5, CEP170, Sdccag8, AKT3, ZBTB18, ADSS, DESI2, Cox20, HNRNPU, Efcab2, KIF26B, SMYD3* |
| 3 | 79647103 | 84441617 | 23.6265 | *TARBP1, Irf2bp2, chr36.1092, chr36.1093, TOMM20, RBM34, ARID4B, GGPS1, tbce, B3GALNT2, GNG4, LYST, NID1, GPR137B, Ero1b, EDARADD, LGALS8, HEATR1, ACTN2, MTR, CHRM3, FMN2, GREM2, Rgs7, PIGM, chr36.1122, OPN3, WDR64, chr36.1125, EXO1, MAP1LC3C, LRIF1, PLD5, CEP170, Sdccag8, AKT3, ZBTB18, ADSS, DESI2, Cox20, HNRNPU, Efcab2, KIF26B, SMYD3* |
| 3 | 79648591 | 84717765 | 33.1171 | *TARBP1, Irf2bp2, chr36.1092, chr36.1093, TOMM20, RBM34, ARID4B, GGPS1, tbce, B3GALNT2, GNG4, LYST, NID1, GPR137B, Ero1b, EDARADD, LGALS8, HEATR1, ACTN2, MTR, CHRM3, FMN2, GREM2, Rgs7, PIGM, chr36.1122, OPN3, WDR64, chr36.1125, EXO1, MAP1LC3C, LRIF1, PLD5, CEP170, Sdccag8, AKT3, ZBTB18, ADSS, DESI2, Cox20, HNRNPU, Efcab2, KIF26B, SMYD3, chr36.1143, TFB2M, CNST, Sccpdh, AHCTF1* |
| 3 | 79659626 | 84424924 | 27.9061 | *Irf2bp2, chr36.1092, chr36.1093, TOMM20, RBM34, ARID4B, GGPS1, tbce, B3GALNT2, GNG4, LYST, NID1, GPR137B, Ero1b, EDARADD, LGALS8, HEATR1, ACTN2, MTR, CHRM3, FMN2, GREM2, Rgs7, PIGM, AHCTF1, chr36.1122, OPN3, WDR64, chr36.1125, EXO1, MAP1LC3C, LRIF1, PLD5, CEP170, Sdccag8, AKT3, ZBTB18, ADSS, DESI2, Cox20, HNRNPU, Efcab2, KIF26B, SMYD3, chr36.1143, TFB2M, CNST, Sccpdh* |
| 3 | 79671950 | 84411331 | 29.0916 | *Irf2bp2, chr36.1092, chr36.1093, TOMM20, RBM34, ARID4B, GGPS1, tbce, B3GALNT2, GNG4, LYST, NID1, GPR137B, Ero1b, EDARADD, LGALS8, HEATR1, ACTN2, MTR, CHRM3, FMN2, GREM2, Rgs7, PIGM, chr36.1122, OPN3, WDR64, chr36.1125, EXO1, MAP1LC3C, LRIF1, PLD5, CEP170, Sdccag8, AKT3, ZBTB18, ADSS, DESI2, Cox20, HNRNPU, Efcab2, KIF26B, SMYD3* |
| 3 | 79679241 | 84689027 | 33.4226 | *Irf2bp2, chr36.1092, chr36.1093, TOMM20, RBM34, ARID4B, GGPS1, tbce, B3GALNT2, GNG4, LYST, NID1, GPR137B, Ero1b, EDARADD, LGALS8, HEATR1, ACTN2, MTR, CHRM3, FMN2, GREM2, Rgs7, PIGM, chr36.1122, OPN3, WDR64, chr36.1125, EXO1, MAP1LC3C, LRIF1, PLD5, CEP170, Sdccag8, AKT3, ZBTB18, ADSS, DESI2, Cox20, HNRNPU, Efcab2, KIF26B, SMYD3, chr36.1143, TFB2M, CNST, Sccpdh, AHCTF1* |
| 3 | 79689567 | 84391184 | 29.7715 | *Irf2bp2, chr36.1092, chr36.1093, TOMM20, RBM34, ARID4B, GGPS1, tbce, B3GALNT2, GNG4, LYST, NID1, GPR137B, Ero1b, EDARADD, LGALS8, HEATR1, ACTN2, MTR, CHRM3, FMN2, GREM2, Rgs7, PIGM, chr36.1122, OPN3, WDR64, chr36.1125, EXO1, MAP1LC3C, LRIF1, PLD5, CEP170, Sdccag8, AKT3, ZBTB18, ADSS, DESI2, Cox20, HNRNPU, Efcab2, KIF26B, SMYD3* |
| 3 | 79709521 | 84662185 | 33.0437 | *Irf2bp2, chr36.1092, chr36.1093, TOMM20, RBM34, ARID4B, GGPS1, tbce, B3GALNT2, GNG4, LYST, NID1, GPR137B, Ero1b, EDARADD, LGALS8, HEATR1, ACTN2, MTR, CHRM3, FMN2, GREM2, Rgs7, PIGM, chr36.1122, OPN3, WDR64, chr36.1125, EXO1, MAP1LC3C, LRIF1, PLD5, CEP170, Sdccag8, AKT3, ZBTB18, ADSS, DESI2, Cox20, HNRNPU, Efcab2, KIF26B, SMYD3, chr36.1143, TFB2M, CNST, Sccpdh, AHCTF1* |
| 3 | 79716026 | 84656619 | 32.0689 | *chr36.1092, chr36.1093, TOMM20, RBM34, ARID4B, GGPS1, tbce, B3GALNT2, GNG4, LYST, NID1, GPR137B, Ero1b, EDARADD, LGALS8, HEATR1, ACTN2, MTR, CHRM3, FMN2, GREM2, Rgs7, PIGM, chr36.1122, OPN3, WDR64, chr36.1125, EXO1, MAP1LC3C, LRIF1, PLD5, CEP170, Sdccag8, AKT3, ZBTB18, ADSS, DESI2, Cox20, HNRNPU, Efcab2, KIF26B, SMYD3, chr36.1143, TFB2M, CNST, Sccpdh* |
| 3 | 79720733 | 84653801 | 30.2639 | *chr36.1092, chr36.1093, TOMM20, RBM34, ARID4B, GGPS1, tbce, B3GALNT2, GNG4, LYST, NID1, GPR137B, Ero1b, EDARADD, LGALS8, HEATR1, ACTN2, MTR, CHRM3, FMN2, GREM2, Rgs7, PIGM, chr36.1122, OPN3, WDR64, chr36.1125, EXO1, MAP1LC3C, LRIF1, PLD5, CEP170, Sdccag8, AKT3, ZBTB18, ADSS, DESI2, Cox20, HNRNPU, Efcab2, KIF26B, SMYD3, chr36.1143, TFB2M, CNST, Sccpdh* |
| 3 | 79752517 | 84621776 | 27.2697 | *chr36.1092, chr36.1093, TOMM20, RBM34, ARID4B, GGPS1, tbce, B3GALNT2, GNG4, LYST, NID1, GPR137B, Ero1b, EDARADD, LGALS8, HEATR1, ACTN2, MTR, CHRM3, FMN2, GREM2, Rgs7, PIGM, CNST, chr36.1122, OPN3, WDR64, chr36.1125, EXO1, MAP1LC3C, LRIF1, PLD5, CEP170, Sdccag8, AKT3, ZBTB18, ADSS, DESI2, Cox20, HNRNPU, Efcab2, KIF26B, SMYD3, chr36.1143, TFB2M* |
| 3 | 79758300 | 84319803 | 30.0429 | *chr36.1092, chr36.1093, TOMM20, RBM34, ARID4B, GGPS1, tbce, B3GALNT2, GNG4, LYST, NID1, GPR137B, Ero1b, EDARADD, LGALS8, HEATR1, ACTN2, MTR, CHRM3, FMN2, GREM2, Rgs7, PIGM, chr36.1122, OPN3, WDR64, chr36.1125, EXO1, MAP1LC3C, LRIF1, PLD5, CEP170, Sdccag8, AKT3, ZBTB18, ADSS, DESI2, Cox20, HNRNPU, Efcab2, KIF26B, SMYD3* |
| 3 | 79760758 | 84312991 | 29.3365 | *chr36.1092, chr36.1093, TOMM20, RBM34, ARID4B, GGPS1, tbce, B3GALNT2, GNG4, LYST, NID1, GPR137B, Ero1b, EDARADD, LGALS8, HEATR1, ACTN2, MTR, CHRM3, FMN2, GREM2, Rgs7, PIGM, chr36.1122, OPN3, WDR64, chr36.1125, EXO1, MAP1LC3C, LRIF1, PLD5, CEP170, Sdccag8, AKT3, ZBTB18, ADSS, DESI2, Cox20, HNRNPU, Efcab2, KIF26B, SMYD3* |
| 3 | 79762874 | 84312991 | 29.8906 | *chr36.1092, chr36.1093, TOMM20, RBM34, ARID4B, GGPS1, tbce, B3GALNT2, GNG4, LYST, NID1, GPR137B, Ero1b, EDARADD, LGALS8, HEATR1, ACTN2, MTR, CHRM3, FMN2, GREM2, Rgs7, PIGM, chr36.1122, OPN3, WDR64, chr36.1125, EXO1, MAP1LC3C, LRIF1, PLD5, CEP170, Sdccag8, AKT3, ZBTB18, ADSS, DESI2, Cox20, HNRNPU, Efcab2, KIF26B, SMYD3* |
| 3 | 79765229 | 84613463 | 20.3124 | *chr36.1092, chr36.1093, TOMM20, RBM34, ARID4B, GGPS1, tbce, B3GALNT2, GNG4, LYST, NID1, GPR137B, Ero1b, EDARADD, LGALS8, HEATR1, ACTN2, MTR, CHRM3, FMN2, GREM2, Rgs7, PIGM, chr36.1122, OPN3, WDR64, chr36.1125, EXO1, MAP1LC3C, LRIF1, PLD5, CEP170, Sdccag8, AKT3, ZBTB18, ADSS, DESI2, Cox20, HNRNPU, Efcab2, KIF26B, SMYD3, chr36.1143, TFB2M, CNST* |
| 3 | 79766602 | 84305433 | 28.3245 | *chr36.1092, chr36.1093, TOMM20, RBM34, ARID4B, GGPS1, tbce, B3GALNT2, GNG4, LYST, NID1, GPR137B, Ero1b, EDARADD, LGALS8, HEATR1, ACTN2, MTR, CHRM3, FMN2, GREM2, Rgs7, PIGM, chr36.1122, OPN3, WDR64, chr36.1125, EXO1, MAP1LC3C, LRIF1, PLD5, CEP170, Sdccag8, AKT3, ZBTB18, ADSS, DESI2, Cox20, HNRNPU, Efcab2, KIF26B, SMYD3* |
| 3 | 79793793 | 84276724 | 26.8725 | *chr36.1092, chr36.1093, TOMM20, RBM34, ARID4B, GGPS1, tbce, B3GALNT2, GNG4, LYST, NID1, GPR137B, Ero1b, EDARADD, LGALS8, HEATR1, ACTN2, MTR, CHRM3, FMN2, GREM2, Rgs7, PIGM, chr36.1122, OPN3, WDR64, chr36.1125, EXO1, MAP1LC3C, LRIF1, PLD5, CEP170, Sdccag8, AKT3, ZBTB18, ADSS, DESI2, Cox20, HNRNPU, Efcab2, KIF26B, SMYD3* |
| 3 | 79797440 | 84583050 | 21.5783 | *chr36.1092, chr36.1093, TOMM20, RBM34, ARID4B, GGPS1, tbce, B3GALNT2, GNG4, LYST, NID1, GPR137B, Ero1b, EDARADD, LGALS8, HEATR1, ACTN2, MTR, CHRM3, FMN2, GREM2, Rgs7, PIGM, chr36.1122, OPN3, WDR64, chr36.1125, EXO1, MAP1LC3C, LRIF1, PLD5, CEP170, Sdccag8, AKT3, ZBTB18, ADSS, DESI2, Cox20, HNRNPU, Efcab2, KIF26B, SMYD3, chr36.1143, TFB2M* |
| 3 | 79816341 | 84566225 | 25.1563 | *chr36.1092, chr36.1093, TOMM20, RBM34, ARID4B, GGPS1, tbce, B3GALNT2, GNG4, LYST, NID1, GPR137B, Ero1b, EDARADD, LGALS8, HEATR1, ACTN2, MTR, CHRM3, FMN2, GREM2, Rgs7, PIGM, chr36.1122, OPN3, WDR64, chr36.1125, EXO1, MAP1LC3C, LRIF1, PLD5, CEP170, Sdccag8, AKT3, ZBTB18, ADSS, DESI2, Cox20, HNRNPU, Efcab2, KIF26B, SMYD3, chr36.1143, TFB2M* |
| 3 | 79832440 | 84236160 | 24.8997 | *chr36.1092, chr36.1093, TOMM20, RBM34, ARID4B, GGPS1, tbce, B3GALNT2, GNG4, LYST, NID1, GPR137B, Ero1b, EDARADD, LGALS8, HEATR1, ACTN2, MTR, CHRM3, FMN2, GREM2, Rgs7, PIGM, chr36.1122, OPN3, WDR64, chr36.1125, EXO1, MAP1LC3C, LRIF1, PLD5, CEP170, Sdccag8, AKT3, ZBTB18, ADSS, DESI2, Cox20, HNRNPU, Efcab2, KIF26B, SMYD3* |
| 3 | 79834229 | 84550422 | 25.9556 | *chr36.1092, chr36.1093, TOMM20, RBM34, ARID4B, GGPS1, tbce, B3GALNT2, GNG4, LYST, NID1, GPR137B, Ero1b, EDARADD, LGALS8, HEATR1, ACTN2, MTR, CHRM3, FMN2, GREM2, Rgs7, PIGM, chr36.1122, OPN3, WDR64, chr36.1125, EXO1, MAP1LC3C, LRIF1, PLD5, CEP170, Sdccag8, AKT3, ZBTB18, ADSS, DESI2, Cox20, HNRNPU, Efcab2, KIF26B, SMYD3* |
| 3 | 79842744 | 84225114 | 22.3657 | *chr36.1092, chr36.1093, TOMM20, RBM34, ARID4B, GGPS1, tbce, B3GALNT2, GNG4, LYST, NID1, GPR137B, Ero1b, EDARADD, LGALS8, HEATR1, ACTN2, MTR, CHRM3, FMN2, GREM2, Rgs7, PIGM, chr36.1122, OPN3, WDR64, chr36.1125, EXO1, MAP1LC3C, LRIF1, PLD5, CEP170, Sdccag8, AKT3, ZBTB18, ADSS, DESI2, Cox20, HNRNPU, Efcab2, KIF26B, SMYD3* |
| 3 | 79844736 | 84541005 | 25.6464 | *chr36.1092, chr36.1093, TOMM20, RBM34, ARID4B, GGPS1, tbce, B3GALNT2, GNG4, LYST, NID1, GPR137B, Ero1b, EDARADD, LGALS8, HEATR1, ACTN2, MTR, CHRM3, FMN2, GREM2, Rgs7, PIGM, chr36.1122, OPN3, WDR64, chr36.1125, EXO1, MAP1LC3C, LRIF1, PLD5, CEP170, Sdccag8, AKT3, ZBTB18, ADSS, DESI2, Cox20, HNRNPU, Efcab2, KIF26B, SMYD3* |
| 3 | 79860822 | 84527822 | 24.6297 | *chr36.1092, chr36.1093, TOMM20, RBM34, ARID4B, GGPS1, tbce, B3GALNT2, GNG4, LYST, NID1, GPR137B, Ero1b, EDARADD, LGALS8, HEATR1, ACTN2, MTR, CHRM3, FMN2, GREM2, Rgs7, PIGM, chr36.1122, OPN3, WDR64, chr36.1125, EXO1, MAP1LC3C, LRIF1, PLD5, CEP170, Sdccag8, AKT3, ZBTB18, ADSS, DESI2, Cox20, HNRNPU, Efcab2, KIF26B, SMYD3* |
| 3 | 79873709 | 84190819 | 19.0658 | *chr36.1093, TOMM20, RBM34, ARID4B, GGPS1, tbce, B3GALNT2, GNG4, LYST, NID1, GPR137B, Ero1b, EDARADD, LGALS8, HEATR1, ACTN2, MTR, CHRM3, FMN2, GREM2, Rgs7, PIGM, chr36.1122, OPN3, WDR64, chr36.1125, EXO1, MAP1LC3C, LRIF1, PLD5, CEP170, Sdccag8, AKT3, ZBTB18, ADSS, DESI2, Cox20, HNRNPU, Efcab2, KIF26B, SMYD3* |
| 3 | 79882943 | 84507011 | 23.0291 | *chr36.1093, TOMM20, RBM34, ARID4B, GGPS1, tbce, B3GALNT2, GNG4, LYST, NID1, GPR137B, Ero1b, EDARADD, LGALS8, HEATR1, ACTN2, MTR, CHRM3, FMN2, GREM2, Rgs7, PIGM, chr36.1122, OPN3, WDR64, chr36.1125, EXO1, MAP1LC3C, LRIF1, PLD5, CEP170, Sdccag8, AKT3, ZBTB18, ADSS, DESI2, Cox20, HNRNPU, Efcab2, KIF26B, SMYD3* |
| 3 | 79889516 | 84172944 | 14.7105 | *TOMM20, RBM34, ARID4B, GGPS1, tbce, B3GALNT2, GNG4, LYST, NID1, GPR137B, Ero1b, EDARADD, LGALS8, HEATR1, ACTN2, MTR, CHRM3, FMN2, GREM2, Rgs7, PIGM, chr36.1122, OPN3, WDR64, chr36.1125, EXO1, MAP1LC3C, LRIF1, PLD5, CEP170, Sdccag8, AKT3, ZBTB18, ADSS, DESI2, Cox20, HNRNPU, Efcab2, KIF26B, SMYD3* |
| 3 | 79911291 | 84149095 | 8.5941 | *ARID4B, GGPS1, tbce, B3GALNT2, GNG4, LYST, NID1, GPR137B, Ero1b, EDARADD, LGALS8, HEATR1, ACTN2, MTR, CHRM3, FMN2, GREM2, Rgs7, PIGM, chr36.1122, OPN3, WDR64, chr36.1125, EXO1, MAP1LC3C, LRIF1, PLD5, CEP170, Sdccag8, AKT3, ZBTB18, ADSS, DESI2, Cox20, HNRNPU, Efcab2, KIF26B* |
| 3 | 79945116 | 84446365 | 20.9385 | *ARID4B, GGPS1, tbce, B3GALNT2, GNG4, LYST, NID1, GPR137B, Ero1b, EDARADD, LGALS8, HEATR1, ACTN2, MTR, CHRM3, FMN2, GREM2, Rgs7, PIGM, chr36.1122, OPN3, WDR64, chr36.1125, EXO1, MAP1LC3C, LRIF1, PLD5, CEP170, Sdccag8, AKT3, ZBTB18, ADSS, DESI2, Cox20, HNRNPU, Efcab2, KIF26B, SMYD3* |
| 3 | 79978513 | 84415978 | 18.2780 | *ARID4B, GGPS1, tbce, B3GALNT2, GNG4, LYST, NID1, GPR137B, Ero1b, EDARADD, LGALS8, HEATR1, ACTN2, MTR, CHRM3, FMN2, GREM2, Rgs7, PIGM, chr36.1122, OPN3, WDR64, chr36.1125, EXO1, MAP1LC3C, LRIF1, PLD5, CEP170, Sdccag8, AKT3, ZBTB18, ADSS, DESI2, Cox20, HNRNPU, Efcab2, KIF26B, SMYD3* |
| 3 | 79985181 | 84411331 | 15.0005 | *ARID4B, GGPS1, tbce, B3GALNT2, GNG4, LYST, NID1, GPR137B, Ero1b, EDARADD, LGALS8, HEATR1, ACTN2, MTR, CHRM3, FMN2, GREM2, Rgs7, PIGM, chr36.1122, OPN3, WDR64, chr36.1125, EXO1, MAP1LC3C, LRIF1, PLD5, CEP170, Sdccag8, AKT3, ZBTB18, ADSS, DESI2, Cox20, HNRNPU, Efcab2, KIF26B, SMYD3* |
| 3 | 80032296 | 84366136 | 10.8187 | *tbce, B3GALNT2, GNG4, LYST, NID1, GPR137B, Ero1b, EDARADD, LGALS8, HEATR1, ACTN2, MTR, CHRM3, FMN2, GREM2, Rgs7, PIGM, chr36.1122, OPN3, WDR64, chr36.1125, EXO1, MAP1LC3C, LRIF1, PLD5, CEP170, Sdccag8, AKT3, ZBTB18, ADSS, DESI2, Cox20, HNRNPU, Efcab2, KIF26B, SMYD3* |
| 3 | 80240209 | 83794251 | 11.5062 | *NID1, GPR137B, Ero1b, EDARADD, LGALS8, HEATR1, ACTN2, MTR, CHRM3, FMN2, GREM2, Rgs7, PIGM, chr36.1122, OPN3, WDR64, chr36.1125, EXO1, MAP1LC3C, LRIF1, PLD5, CEP170, Sdccag8, AKT3, ZBTB18, ADSS, DESI2, Cox20, HNRNPU* |
| 3 | 80257077 | 83775247 | 15.6195 | *NID1, GPR137B, Ero1b, EDARADD, LGALS8, HEATR1, ACTN2, MTR, CHRM3, FMN2, GREM2, Rgs7, PIGM, chr36.1122, OPN3, WDR64, chr36.1125, EXO1, MAP1LC3C, LRIF1, PLD5, CEP170, Sdccag8, AKT3, ZBTB18, ADSS, DESI2, Cox20, HNRNPU* |
| 3 | 80272808 | 83758341 | 17.6612 | *NID1, GPR137B, Ero1b, EDARADD, LGALS8, HEATR1, ACTN2, MTR, CHRM3, FMN2, GREM2, Rgs7, PIGM, chr36.1122, OPN3, WDR64, chr36.1125, EXO1, MAP1LC3C, LRIF1, PLD5, CEP170, Sdccag8, AKT3, ZBTB18, ADSS, DESI2, Cox20* |
| 3 | 80281898 | 83746588 | 18.0855 | *NID1, GPR137B, Ero1b, EDARADD, LGALS8, HEATR1, ACTN2, MTR, CHRM3, FMN2, GREM2, Rgs7, PIGM, chr36.1122, OPN3, WDR64, chr36.1125, EXO1, MAP1LC3C, LRIF1, PLD5, CEP170, Sdccag8, AKT3, ZBTB18, ADSS, DESI2* |
| 3 | 80298814 | 83727631 | 16.5082 | *NID1, GPR137B, Ero1b, EDARADD, LGALS8, HEATR1, ACTN2, MTR, CHRM3, FMN2, GREM2, Rgs7, PIGM, chr36.1122, OPN3, WDR64, chr36.1125, EXO1, MAP1LC3C, LRIF1, PLD5, CEP170, Sdccag8, AKT3, ZBTB18, ADSS, DESI2* |
| 3 | 80309525 | 83714798 | 9.9863 | *GPR137B, Ero1b, EDARADD, LGALS8, HEATR1, ACTN2, MTR, CHRM3, FMN2, GREM2, Rgs7, PIGM, ADSS chr36.1122, OPN3, WDR64, chr36.1125, EXO1, MAP1LC3C, LRIF1, PLD5, CEP170, Sdccag8, AKT3, ZBTB18* |
| 3 | 80324543 | 83699530 | 10.1968 | *GPR137B, Ero1b, EDARADD, LGALS8, HEATR1, ACTN2, MTR, CHRM3, FMN2, GREM2, Rgs7, PIGM, ADSS chr36.1122, OPN3, WDR64, chr36.1125, EXO1, MAP1LC3C, LRIF1, PLD5, CEP170, Sdccag8, AKT3, ZBTB18* |
| 3 | 80342257 | 83678424 | 20.1689 | *Ero1b, EDARADD, LGALS8, HEATR1, ACTN2, MTR, CHRM3, FMN2, GREM2, Rgs7, PIGM, chr36.1122, OPN3, WDR64, chr36.1125, EXO1, MAP1LC3C, LRIF1, PLD5, CEP170, Sdccag8, AKT3, ZBTB18, ADSS* |
| 3 | 80345173 | 83673292 | 24.7686 | *Ero1b, EDARADD, LGALS8, HEATR1, ACTN2, MTR, CHRM3, FMN2, GREM2, Rgs7, PIGM, chr36.1122, OPN3, WDR64, chr36.1125, EXO1, MAP1LC3C, LRIF1, PLD5, CEP170, Sdccag8, AKT3, ZBTB18, ADSS* |
| 3 | 80361551 | 83654812 | 27.6288 | *Ero1b, EDARADD, LGALS8, HEATR1, ACTN2, MTR, CHRM3, FMN2, GREM2, Rgs7, PIGM, chr36.1122, OPN3, WDR64, chr36.1125, EXO1, MAP1LC3C, LRIF1, PLD5, CEP170, Sdccag8, AKT3, ZBTB18* |
| 3 | 80381867 | 83632214 | 29.4966 | *EDARADD, LGALS8, HEATR1, ACTN2, MTR, CHRM3, FMN2, GREM2, Rgs7, PIGM, chr36.1122, OPN3, WDR64, chr36.1125, EXO1, MAP1LC3C, LRIF1, PLD5, CEP170, Sdccag8, AKT3, ZBTB18* |
| 3 | 80403864 | 83608749 | 30.6494 | *LGALS8, HEATR1, ACTN2, MTR, CHRM3, FMN2, GREM2, Rgs7, PIGM, chr36.1122, OPN3, WDR64, chr36.1125, EXO1, MAP1LC3C, LRIF1, PLD5, CEP170, Sdccag8, AKT3, ZBTB18* |
| 3 | 80421863 | 83588449 | 31.2432 | *HEATR1, ACTN2, MTR, CHRM3, FMN2, GREM2, Rgs7, PIGM, chr36.1122, OPN3, WDR64, chr36.1125, EXO1, MAP1LC3C, LRIF1, PLD5, CEP170, Sdccag8, AKT3, ZBTB18* |
| 3 | 80448332 | 83560325 | 31.3460 | *ACTN2, MTR, CHRM3, FMN2, GREM2, Rgs7, PIGM, chr36.1122, OPN3, WDR64, chr36.1125, EXO1, MAP1LC3C, LRIF1, PLD5, CEP170, Sdccag8, AKT3, ZBTB18* |
| 3 | 80464942 | 83541548 | 30.9728 | *ACTN2, MTR, CHRM3, FMN2, GREM2, Rgs7, PIGM, chr36.1122, OPN3, WDR64, chr36.1125, EXO1, MAP1LC3C, LRIF1, PLD5, CEP170, Sdccag8, AKT3* |
| 3 | 80488144 | 83516246 | 30.1311 | *ACTN2, MTR, CHRM3, FMN2, GREM2, Rgs7, PIGM, chr36.1122, OPN3, WDR64, chr36.1125, EXO1, MAP1LC3C, LRIF1, PLD5, CEP170, Sdccag8, AKT3* |
| 3 | 80509655 | 83493895 | 28.7654 | *ACTN2, MTR, CHRM3, FMN2, GREM2, Rgs7, PIGM, chr36.1122, OPN3, WDR64, chr36.1125, EXO1, MAP1LC3C, LRIF1, PLD5, CEP170, Sdccag8, AKT3* |
| 3 | 80533173 | 83466959 | 26.8625 | *MTR, CHRM3, FMN2, GREM2, Rgs7, PIGM, chr36.1122, OPN3, WDR64, chr36.1125, EXO1, MAP1LC3C, LRIF1, PLD5, CEP170, Sdccag8, AKT3* |
| 3 | 80558625 | 83440351 | 24.2446 | *MTR, CHRM3, FMN2, GREM2, Rgs7, PIGM, chr36.1122, OPN3, WDR64, chr36.1125, EXO1, MAP1LC3C, LRIF1, PLD5, CEP170, Sdccag8, AKT3* |
| 3 | 80582351 | 83414182 | 20.7025 | *MTR, CHRM3, FMN2, GREM2, Rgs7, PIGM, chr36.1122, OPN3, WDR64, chr36.1125, EXO1, MAP1LC3C, LRIF1, PLD5, CEP170, Sdccag8, AKT3* |
| 3 | 80610096 | 83383796 | 15.5944 | *CHRM3, FMN2, GREM2, Rgs7, PIGM, chr36.1122, OPN3, WDR64, chr36.1125, EXO1, MAP1LC3C, LRIF1, PLD5, CEP170, Sdccag8, AKT3* |
| 3 | 81298620 | 83128196 | 14.6443 | *CHRM3, FMN2, GREM2, Rgs7, PIGM, chr36.1122, OPN3, WDR64, chr36.1125, EXO1, MAP1LC3C, LRIF1, PLD5, CEP170* |
| 3 | 81312728 | 83111868 | 10.7946 | *CHRM3, FMN2, GREM2, Rgs7, PIGM, chr36.1122, OPN3, WDR64, chr36.1125, EXO1, MAP1LC3C, LRIF1, PLD5* |
| 3 | 81314576 | 83115558 | 19.9104 | *CHRM3, FMN2, GREM2, Rgs7, PIGM, chr36.1122, OPN3, WDR64, chr36.1125, EXO1, MAP1LC3C, LRIF1, PLD5, CEP170* |
| 3 | 81320786 | 83108744 | 17.6094 | *CHRM3, FMN2, GREM2, Rgs7, PIGM, chr36.1122, OPN3, WDR64, chr36.1125, EXO1, MAP1LC3C, LRIF1, PLD5* |
| 3 | 81321923 | 83110791 | 21.7022 | *CHRM3, FMN2, GREM2, Rgs7, PIGM, chr36.1122, OPN3, WDR64, chr36.1125, EXO1, MAP1LC3C, LRIF1, PLD5* |
| 3 | 81336468 | 83098137 | 23.0553 | *CHRM3, FMN2, GREM2, Rgs7, PIGM, chr36.1122, OPN3, WDR64, chr36.1125, EXO1, MAP1LC3C, LRIF1, PLD5* |
| 3 | 81353935 | 82274517 | 17.5604 | *CHRM3, FMN2, GREM2, Rgs7* |
| 3 | 81355927 | 83081783 | 24.0205 | *CHRM3, FMN2, GREM2, Rgs7, PIGM, chr36.1122, OPN3, WDR64, chr36.1125, EXO1, MAP1LC3C, LRIF1, PLD5* |
| 3 | 81359969 | 83079042 | 24.6506 | *CHRM3, FMN2, GREM2, Rgs7, PIGM, chr36.1122, OPN3, WDR64, chr36.1125, EXO1, MAP1LC3C, LRIF1, PLD5* |
| 3 | 81374539 | 82251643 | 14.7667 | *CHRM3, FMN2, GREM2, Rgs7* |
| 3 | 81387026 | 83053831 | 24.9229 | *CHRM3, FMN2, GREM2, Rgs7, PIGM, chr36.1122, OPN3, WDR64, chr36.1125, EXO1, MAP1LC3C, LRIF1, PLD5* |
| 3 | 81399098 | 83043329 | 24.7963 | *CHRM3, FMN2, GREM2, Rgs7, PIGM, chr36.1122, OPN3, WDR64, chr36.1125, EXO1, MAP1LC3C, LRIF1, PLD5* |
| 3 | 81424897 | 83019758 | 24.2322 | *CHRM3, FMN2, GREM2, Rgs7, PIGM, chr36.1122, OPN3, WDR64, chr36.1125, EXO1, MAP1LC3C, LRIF1, PLD5* |
| 3 | 81445954 | 83001102 | 23.0618 | *CHRM3, FMN2, GREM2, Rgs7, PIGM, chr36.1122, OPN3, WDR64, chr36.1125, EXO1, MAP1LC3C, LRIF1, PLD5* |
| 3 | 81463751 | 82984657 | 21.0184 | *CHRM3, FMN2, GREM2, Rgs7, PIGM, chr36.1122, OPN3, WDR64, chr36.1125, EXO1, MAP1LC3C, LRIF1, PLD5* |
| 3 | 81470296 | 82096206 | 8.6821 | *CHRM3, FMN2* |
| 3 | 81495290 | 82955023 | 17.2325 | *CHRM3, FMN2, GREM2, Rgs7, PIGM, chr36.1122, OPN3, WDR64, chr36.1125, EXO1, MAP1LC3C, LRIF1, PLD5* |
| 3 | 81546214 | 82636760 | 9.7284 | *CHRM3, FMN2, GREM2, Rgs7, PIGM, chr36.1122, OPN3, WDR64* |
| 3 | 81553407 | 82629320 | 9.3415 | *CHRM3, FMN2, GREM2, Rgs7, PIGM, chr36.1122, OPN3, WDR64* |
| 3 | 81556370 | 82898106 | 10.9606 | *CHRM3, FMN2, GREM2, Rgs7, PIGM, chr36.1122, OPN3, WDR64, chr36.1125, EXO1, MAP1LC3C, LRIF1, PLD5* |
| 3 | 81565107 | 82028417 | 15.3333 | *CHRM3, FMN2* |
| 3 | 81565661 | 82620307 | 8.5020 | *CHRM3, FMN2, GREM2, Rgs7, PIGM, chr36.1122, OPN3, WDR64* |
| 3 | 81585338 | 82870993 | 17.6409 | *CHRM3, FMN2, GREM2, Rgs7, PIGM, chr36.1122, OPN3, WDR64, chr36.1125, EXO1, MAP1LC3C, LRIF1, PLD5* |
| 3 | 81589364 | 82018584 | 13.5649 | *CHRM3, FMN2* |
| 3 | 81608786 | 82023525 | 11.6744 | *CHRM3, FMN2* |
| 3 | 81613276 | 82845057 | 20.2401 | *CHRM3, FMN2, GREM2, Rgs7, PIGM, chr36.1122, OPN3, WDR64, chr36.1125, EXO1, MAP1LC3C, LRIF1, PLD5* |
| 3 | 81635422 | 82561349 | 8.5610 | *CHRM3, FMN2, GREM2, Rgs7, PIGM, chr36.1122, OPN3* |
| 3 | 81655106 | 82543375 | 10.5942 | *CHRM3, FMN2, GREM2, Rgs7, PIGM, chr36.1122* |
| 3 | 81657351 | 82803860 | 21.3637 | *CHRM3, FMN2, GREM2, Rgs7, PIGM, chr36.1122, OPN3, WDR64, chr36.1125, EXO1, MAP1LC3C, LRIF1* |
| 3 | 81670216 | 82590924 | 11.0958 | *CHRM3, FMN2, GREM2, Rgs7, PIGM, chr36.1122, OPN3, WDR64* |
| 3 | 81670919 | 82593032 | 10.7900 | *CHRM3, FMN2, GREM2, Rgs7, PIGM, chr36.1122, OPN3, WDR64* |
| 3 | 81672868 | 82587643 | 11.1011 | *CHRM3, FMN2, GREM2, Rgs7, PIGM, chr36.1122, OPN3, WDR64* |
| 3 | 81676805 | 82589494 | 10.2574 | *CHRM3, FMN2, GREM2, Rgs7, PIGM, chr36.1122, OPN3, WDR64* |
| 3 | 81678326 | 82520933 | 11.6548 | *CHRM3, FMN2, GREM2, Rgs7, PIGM, chr36.1122* |
| 3 | 81683772 | 81983952 | 19.7032 | *CHRM3, FMN2* |
| 3 | 81683772 | 82574230 | 10.6016 | *CHRM3, FMN2, GREM2, Rgs7, PIGM, chr36.1122, OPN3* |
| 3 | 81683772 | 82583353 | 9.5045 | *CHRM3, FMN2, GREM2, Rgs7, PIGM, chr36.1122, OPN3, WDR64* |
| 3 | 81690436 | 81975602 | 17.4930 | *CHRM3, FMN2* |
| 3 | 81694043 | 82577159 | 8.4770 | *CHRM3, FMN2, GREM2, Rgs7, PIGM, chr36.1122, OPN3* |
| 3 | 81695962 | 82559709 | 9.0661 | *CHRM3, FMN2, GREM2, Rgs7, PIGM, chr36.1122, OPN3* |
| 3 | 81700284 | 82762403 | 21.6137 | *CHRM3, FMN2, GREM2, Rgs7, PIGM, chr36.1122, OPN3, WDR64, chr36.1125, EXO1, MAP1LC3C, LRIF1* |
| 3 | 81705901 | 82208748 | 7.6810 | *CHRM3, FMN2, GREM2, Rgs7* |
| 3 | 81710784 | 82491209 | 12.0552 | *CHRM3, FMN2, GREM2, Rgs7* |
| 3 | 81717647 | 82201951 | 7.7303 | *CHRM3, FMN2, GREM2* |
| 3 | 81734910 | 82728743 | 21.2462 | *CHRM3, FMN2, GREM2, Rgs7, PIGM, chr36.1122, OPN3, WDR64, chr36.1125, EXO1, MAP1LC3C, LRIF1* |
| 3 | 81735712 | 82885496 | 11.1307 | *CHRM3, FMN2, GREM2, Rgs7, PIGM, chr36.1122, OPN3, WDR64, chr36.1125, EXO1, MAP1LC3C, LRIF1, PLD5* |
| 3 | 81737533 | 81932891 | 10.6008 | *CHRM3* |
| 3 | 81739785 | 82511075 | 9.4019 | *CHRM3, FMN2, GREM2, Rgs7, PIGM, chr36.1122* |
| 3 | 81739785 | 82874531 | 9.9524 | *CHRM3, FMN2, GREM2, Rgs7, PIGM, chr36.1122, OPN3, WDR64, chr36.1125, EXO1, MAP1LC3C, LRIF1, PLD5* |
| 3 | 81751857 | 82454127 | 11.8939 | *CHRM3, FMN2, GREM2, Rgs7* |
| 3 | 81757193 | 82491209 | 9.8608 | *CHRM3, FMN2, GREM2, Rgs7* |
| 3 | 81762143 | 82860186 | 12.3474 | *CHRM3, FMN2, GREM2, Rgs7, PIGM, chr36.1122, OPN3, WDR64, chr36.1125, EXO1, MAP1LC3C, LRIF1, PLD5* |
| 3 | 81764476 | 82848048 | 9.9616 | *CHRM3, FMN2, GREM2, Rgs7, PIGM, chr36.1122, OPN3, WDR64, chr36.1125, EXO1, MAP1LC3C, LRIF1, PLD5* |
| 3 | 81773549 | 82692200 | 20.3320 | *CHRM3, FMN2, GREM2, Rgs7, PIGM, chr36.1122, OPN3, WDR64, chr36.1125, EXO1* |
| 3 | 81779085 | 82846243 | 9.1956 | *CHRM3, FMN2, GREM2, Rgs7, PIGM, chr36.1122, OPN3, WDR64, chr36.1125, EXO1, MAP1LC3C, LRIF1, PLD5* |
| 3 | 81787294 | 82462482 | 8.2341 | *CHRM3, FMN2, GREM2, Rgs7* |
| 3 | 81807265 | 82399490 | 11.1812 | *CHRM3, FMN2, GREM2, Rgs7* |
| 3 | 81815060 | 82654906 | 18.8159 | *CHRM3, FMN2, GREM2, Rgs7, PIGM, chr36.1122, OPN3, WDR64, chr36.1125, EXO1* |
| 3 | 81850384 | 82620307 | 16.5521 | *CHRM3, FMN2, GREM2, Rgs7, PIGM, chr36.1122, OPN3, WDR64* |
| 3 | 81865733 | 82343621 | 9.6938 | *CHRM3, FMN2, GREM2, Rgs7* |
| 3 | 81899028 | 82573676 | 13.2872 | *CHRM3, FMN2, GREM2, Rgs7, PIGM, chr36.1122, OPN3* |
| 3 | 81957047 | 82520933 | 8.6111 | *FMN2, GREM2, Rgs7, PIGM, chr36.1122* |
| 3 | 82331039 | 82491209 | 8.1645 | *Rgs7* |
| 3 | 82337530 | 82487016 | 9.5690 | *Rgs7* |
| 3 | 84197667 | 84383182 | 10.1488 | *SMYD3* |
| 3 | 84249502 | 84357036 | 8.3312 | *SMYD3* |
| 3 | 116912476 | 117602664 | 7.6960 | *PLCB4, Lamp5, Pak5, Bdh1, Ankef1, Snap25, MKKS, SLX4IP, chr36.1692, JAG1* |
| 3 | 116912476 | 117605478 | 9.3746 | *PLCB4, Lamp5, Pak5, Bdh1, Ankef1, Snap25, MKKS, SLX4IP, chr36.1692, JAG1* |
| 3 | 116926613 | 117540082 | 9.6069 | *PLCB4, Lamp5, Pak5, Bdh1, Ankef1, Snap25, MKKS, SLX4IP, chr36.1692, JAG1* |
| 3 | 116927419 | 117535454 | 9.3929 | *PLCB4, Lamp5, Pak5, Bdh1, Ankef1, Snap25, MKKS, SLX4IP, chr36.1692, JAG1* |
| 3 | 116927419 | 117541600 | 10.8245 | *PLCB4, Lamp5, Pak5, Bdh1, Ankef1, Snap25, MKKS, SLX4IP, chr36.1692, JAG1* |
| 3 | 116928610 | 117532372 | 12.5539 | *PLCB4, Lamp5, Pak5, Bdh1, Ankef1, Snap25, MKKS, SLX4IP, chr36.1692, JAG1* |
| 3 | 116929035 | 117590225 | 8.1949 | *PLCB4, Lamp5, Pak5, Bdh1, Ankef1, Snap25, MKKS, SLX4IP, chr36.1692, JAG1* |
| 3 | 116932512 | 117538941 | 7.8927 | *PLCB4, Lamp5, Pak5, Bdh1, Ankef1, Snap25, MKKS, SLX4IP, chr36.1692, JAG1* |
| 3 | 116933363 | 117525898 | 13.5358 | *PLCB4, Lamp5, Pak5, Bdh1, Ankef1, Snap25, MKKS, SLX4IP, chr36.1692, JAG1* |
| 3 | 116934409 | 117570790 | 8.9169 | *PLCB4, Lamp5, Pak5, Bdh1, Ankef1, Snap25, MKKS, SLX4IP, chr36.1692, JAG1* |
| 3 | 116940626 | 117516405 | 13.4667 | *PLCB4, Lamp5, Pak5, Bdh1, Ankef1, Snap25, MKKS, SLX4IP, chr36.1692, JAG1* |
| 3 | 116945880 | 117557305 | 8.1046 | *PLCB4, Lamp5, Pak5, Bdh1, Ankef1, Snap25, MKKS, SLX4IP, chr36.1692, JAG1* |
| 3 | 116949090 | 117525898 | 8.7861 | *PLCB4, Lamp5, Pak5, Bdh1, Ankef1, Snap25, MKKS, SLX4IP, chr36.1692, JAG1* |
| 3 | 116954612 | 117500589 | 12.3242 | *PLCB4, Lamp5, Pak5, Bdh1, Ankef1, Snap25, MKKS, SLX4IP, chr36.1692, JAG1* |
| 3 | 116986434 | 117466754 | 9.4155 | *Lamp5, Pak5, Bdh1, Ankef1, Snap25, MKKS, SLX4IP, chr36.1692, JAG1* |
| 3 | 117041263 | 117379045 | 12.1806 | *Bdh1, Ankef1, Snap25, MKKS, SLX4IP, chr36.1692, JAG1* |
| 3 | 117046745 | 117376380 | 10.4909 | *Bdh1, Ankef1, Snap25, MKKS, SLX4IP* |
| 3 | 117099046 | 117293489 | 8.0341 | *Bdh1, Ankef1, Snap25* |
| 3 | 117107193 | 117289805 | 7.9345 | *Bdh1, Ankef1, Snap25* |
| 4 | 15652158 | 15702774 | 10.6475 | *C1QTNF7, CC2D2A* |
| 4 | 15684584 | 16257019 | 8.7815 | *CC2D2A, FBXL5, BST1, chr35.304, Cd38, Fgfbp1, FGFBP2, prom1a, TAPT1, LDB2* |
| 4 | 15938864 | 16150462 | 11.4191 | *TAPT1, LDB2* |
| 4 | 15946362 | 16118728 | 10.0555 | *TAPT1, LDB2* |
| 4 | 27934466 | 28301142 | 17.0029 | *RASL11B, SCFD2, chr35.460, FIP1L1, LNX1* |
| 4 | 27945164 | 28292036 | 11.0298 | *RASL11B, SCFD2, chr35.460, FIP1L1, LNX1* |
| 4 | 27997937 | 28206656 | 9.5870 | *SCFD2, chr35.460, FIP1L1* |
| 4 | 68813816 | 69322235 | 7.9430 | *PDLIM5, BMPR1B, UNC5C* |
| 4 | 68834642 | 69303895 | 8.4994 | *BMPR1B, UNC5C* |
| 4 | 77747541 | 77830766 | 8.8792 | *CDS1, WDFY3* |
| 5 | 8990247 | 9132025 | 9.3256 | *AHNAK2, PLD4, Cep170b* |
| 5 | 11085613 | 13218449 | 37.3829 | *CDC42BPB, AMN, chr34.245, Traf3, RCOR1, Ankrd9, TECPR2, CINP, ZNF839, MOK, WDR20, HSP90AA1, chr34.260.2, chr34.261, chr34.262, DYNC1H1, PPP2R5C, chr34.265, DIO3, DLK1, BEGAIN, WDR25, WARS, Slc25a47, Slc25a29, chr34.278, YY1, Degs2, EVL, EML1, PKDCC, CYP46A1, Hhipl1, ccdc85c, CCNK, SETD3* |
| 5 | 11094958 | 13205118 | 37.5300 | *CDC42BPB, AMN, chr34.245, Traf3, RCOR1, Ankrd9, TECPR2, CINP, ZNF839, MOK, WDR20, HSP90AA1, chr34.260.2, chr34.261, chr34.262, DYNC1H1, Dync1h1, PPP2R5C, chr34.265, DIO3, DLK1, BEGAIN, WDR25, WARS, Slc25a47, Slc25a29, chr34.278, YY1, Degs2, EVL, EML1, PKDCC, CYP46A1, Hhipl1, ccdc85c, CCNK* |
| 5 | 11097718 | 13204338 | 43.0638 | *CDC42BPB, AMN, chr34.245, Traf3, RCOR1, Ankrd9, TECPR2, CINP, ZNF839, MOK, WDR20, HSP90AA1, chr34.260.2, chr34.261, chr34.262, DYNC1H1, Dync1h1, PPP2R5C, chr34.265, DIO3, DLK1, BEGAIN, WDR25, WARS, Slc25a47, Slc25a29, chr34.278, YY1, Degs2, EVL, EML1, PKDCC, CYP46A1, Hhipl1, ccdc85c, CCNK* |
| 5 | 11104589 | 13204338 | 46.2537 | *CDC42BPB, AMN, chr34.245, Traf3, RCOR1, Ankrd9, TECPR2, CINP, ZNF839, MOK, WDR20, HSP90AA1, chr34.260.2, chr34.261, chr34.262, DYNC1H1, Hhipl1, ccdc85c, CCNK, Dync1h1, PPP2R5C, chr34.265, DIO3, DLK1, BEGAIN, WDR25, WARS, Slc25a47, Slc25a29, chr34.278, YY1, Degs2, EVL, EML1, PKDCC, CYP46A1* |
| 5 | 11109265 | 13196845 | 48.9428 | *CDC42BPB, AMN, chr34.245, Traf3, RCOR1, Ankrd9, TECPR2, CINP, ZNF839, MOK, WDR20, HSP90AA1, chr34.260.2, chr34.261, chr34.262, DYNC1H1, Hhipl1, ccdc85c, CCNK, Dync1h1, PPP2R5C, chr34.265, DIO3, DLK1, BEGAIN, WDR25, WARS, Slc25a47, Slc25a29, chr34.278, YY1, Degs2, EVL, EML1, PKDCC, CYP46A1* |
| 5 | 11126799 | 13183607 | 31.5641 | *CDC42BPB, AMN, chr34.245, Traf3, RCOR1, Ankrd9, TECPR2, CINP, ZNF839, MOK, WDR20, HSP90AA1, chr34.260.2, chr34.261, chr34.262, DYNC1H1, CYP46A1, Hhipl1, Dync1h1, PPP2R5C, chr34.265, DIO3, DLK1, BEGAIN, WDR25, WARS, Slc25a47, Slc25a29, chr34.278, YY1, Degs2, EVL, EML1, PKDCC* |
| 5 | 11138100 | 13174038 | 16.3753 | *CDC42BPB, AMN, chr34.245, Traf3, RCOR1, Ankrd9, TECPR2, CINP, ZNF839, MOK, WDR20, HSP90AA1, chr34.260.2, chr34.261, chr34.262, DYNC1H1, CYP46A1, Hhipl1, ccdc85c, Dync1h1, PPP2R5C, chr34.265, DIO3, DLK1, BEGAIN, WDR25, WARS, Slc25a47, Slc25a29, chr34.278, YY1, Degs2, EVL, EML1, PKDCC* |
| 5 | 11170665 | 13143316 | 14.0175 | *chr34.245, Traf3, RCOR1, Ankrd9, TECPR2, CINP, ZNF839, MOK, WDR20, HSP90AA1, chr34.260.2, chr34.261, chr34.262, DYNC1H1, Dync1h1, PPP2R5C, chr34.265, DIO3, DLK1, BEGAIN, WDR25, WARS, Slc25a47, Slc25a29, chr34.278, YY1, Degs2, EVL, EML1, PKDCC, CYP46A1, Hhipl1, ccdc85c* |
| 5 | 11192286 | 13123155 | 8.8400 | *Traf3, RCOR1, Ankrd9, TECPR2, CINP, ZNF839, MOK, WDR20, HSP90AA1, chr34.260.2, chr34.261, chr34.262, DYNC1H1, Dync1h1, PPP2R5C, chr34.265, DIO3, DLK1, BEGAIN, WDR25, WARS, Slc25a47, Slc25a29, chr34.278, YY1, Degs2, EVL, EML1, PKDCC, CYP46A1, Hhipl1, ccdc85c* |
| 5 | 11439636 | 12796551 | 18.9362 | *TECPR2, CINP, ZNF839, MOK, WDR20, HSP90AA1, chr34.260.2, chr34.261, chr34.262, DYNC1H1, PPP2R5C, chr34.265, DIO3, DLK1, BEGAIN, WDR25, WARS, Slc25a47, Slc25a29, chr34.278, YY1, Degs2, EVL* |
| 5 | 11451206 | 12787226 | 15.0938 | *TECPR2, CINP, ZNF839, MOK, WDR20, HSP90AA1, chr34.260.2, chr34.261, chr34.262, DYNC1H1, PPP2R5C, chr34.265, DIO3, DLK1, BEGAIN, WDR25, WARS, Slc25a47, Slc25a29, chr34.278, YY1, Degs2, EVL* |
| 5 | 11451744 | 12485987 | 13.1800 | *TECPR2, CINP, ZNF839, MOK, WDR20, HSP90AA1, chr34.260.2, chr34.261, chr34.262, DYNC1H1, PPP2R5C, chr34.265, DIO3, DLK1, BEGAIN,* |
| 5 | 11452157 | 12485987 | 13.0315 | *TECPR2, CINP, ZNF839, MOK, WDR20, HSP90AA1, chr34.260.2, chr34.261, chr34.262, DYNC1H1, PPP2R5C, chr34.265, DIO3, DLK1, BEGAIN,* |
| 5 | 11462810 | 12482817 | 11.4808 | *TECPR2, CINP, ZNF839, MOK, WDR20, HSP90AA1, chr34.260.2, chr34.261, chr34.262, DYNC1H1, PPP2R5C, chr34.265, DIO3, DLK1, BEGAIN,* |
| 5 | 11466308 | 12459710 | 12.0405 | *TECPR2, CINP, ZNF839, MOK, WDR20, HSP90AA1, chr34.260.2, chr34.261, chr34.262, DYNC1H1, PPP2R5C, chr34.265, DIO3, DLK1, BEGAIN,* |
| 5 | 11466762 | 12965178 | 69.0624 | *TECPR2, CINP, ZNF839, MOK, WDR20, HSP90AA1, chr34.260.2, chr34.261, chr34.262, DYNC1H1, PPP2R5C, chr34.265, DIO3, DLK1, BEGAIN, WDR25, WARS, Slc25a47, Slc25a29, chr34.278, YY1, Degs2, EVL, EML1* |
| 5 | 11469376 | 12764859 | 11.7576 | *TECPR2, CINP, ZNF839, MOK, WDR20, HSP90AA1, chr34.260.2, chr34.261, chr34.262, DYNC1H1, PPP2R5C, chr34.265, DIO3, DLK1, BEGAIN, WDR25, WARS, Slc25a47, Slc25a29, chr34.278, YY1, Degs2, EVL* |
| 5 | 11472907 | 12955319 | 62.0230 | *TECPR2, CINP, ZNF839, MOK, WDR20, HSP90AA1, chr34.260.2, chr34.261, chr34.262, DYNC1H1, PPP2R5C, chr34.265, DIO3, DLK1, BEGAIN, WDR25, WARS, Slc225a47, Slc25a29, chr34.278, YY1, Degs2, EVL, EML1* |
| 5 | 11476149 | 12957562 | 59.9653 | *TECPR2, CINP, ZNF839, MOK, WDR20, HSP90AA1, chr34.260.2, chr34.261, chr34.262, DYNC1H1, PPP2R5C, chr34.265, DIO3, DLK1, BEGAIN, WDR25, WARS, Slc225a47, Slc25a29, chr34.278, YY1, Degs2, EVL, EML1* |
| 5 | 11477045 | 12952913 | 68.5887 | *TECPR2, CINP, ZNF839, MOK, WDR20, HSP90AA1, chr34.260.2, chr34.261, chr34.262, DYNC1H1, PPP2R5C, chr34.265, DIO3, DLK1, BEGAIN, WDR25, WARS, Slc225a47, Slc25a29, chr34.278, YY1, Degs2, EVL, EML1* |
| 5 | 11488600 | 12442116 | 9.5070 | *CINP, ZNF839, MOK, WDR20, HSP90AA1, chr34.260.2, chr34.261, chr34.262, DYNC1H1, PPP2R5C, chr34.265, DIO3, DLK1, BEGAIN,* |
| 5 | 11492883 | 12442116 | 8.0606 | *CINP, ZNF839, MOK, WDR20, HSP90AA1, chr34.260.2, chr34.261, chr34.262, DYNC1H1, PPP2R5C, chr34.265, DIO3, DLK1, BEGAIN,* |
| 5 | 11495798 | 12930087 | 49.2659 | *CINP, ZNF839, MOK, WDR20, HSP90AA1, chr34.260.2, chr34.261, chr34.262, DYNC1H1, PPP2R5C, chr34.265, DIO3, DLK1, BEGAIN, WDR25, WARS, Slc225a47, Slc25a29, chr34.278, YY1, Degs2, EVL, EML1* |
| 5 | 11522520 | 12913748 | 56.9118 | *MOK, WDR20, HSP90AA1, chr34.260.2, chr34.261, chr34.262, DYNC1H1, PPP2R5C, chr34.265, DIO3, DLK1, BEGAIN, WDR25, WARS, Slc225a47, Slc25a29, chr34.278, YY1, Degs2, EVL, EML1* |
| 5 | 11555277 | 12882830 | 55.3921 | *WDR20, HSP90AA1, chr34.260.2, chr34.261, chr34.262, DYNC1H1, EML1, PPP2R5C, chr34.265, DIO3, DLK1, BEGAIN, WDR25, WARS, Slc225a47, Slc25a29, chr34.278, YY1, Degs2, EVL* |
| 5 | 11564760 | 12859400 | 28.3691 | *WDR20, HSP90AA1, chr34.260.2, chr34.261, chr34.262, DYNC1H1, EML1, PPP2R5C, chr34.265, DIO3, DLK1, BEGAIN, WDR25, WARS, Slc225a47, Slc25a29, chr34.278, YY1, Degs2, EVL* |
| 5 | 11655346 | 12784771 | 43.5125 | *DYNC1H1, PPP2R5C, chr34.265, DIO3, DLK1, BEGAIN, WDR25, WARS, Slc225a47, Slc25a29, chr34.278, YY1, Degs2, EVL* |
| 5 | 11675184 | 12568930 | 13.1251 | *PPP2R5C, chr34.265, DIO3, DLK1, BEGAIN, WDR25* |
| 5 | 11771695 | 12365357 | 21.7399 | *DIO3, DLK1* |
| 5 | 11771770 | 12365357 | 15.4373 | *DIO3, DLK1* |
| 5 | 11773478 | 12036538 | 8.8369 | *DIO3* |
| 5 | 11785071 | 12365357 | 22.3688 | *DIO3, DLK1* |
| 5 | 11786782 | 12966110 | 34.4820 | *DIO3, DLK1, BEGAIN, WDR25, WARS, Slc25a47, Slc25a29, chr34.278, YY1, Degs2, EVL, EML1* |
| 5 | 11786782 | 12967425 | 32.9731 | *DIO3, DLK1, BEGAIN, WDR25, WARS, Slc25a47, Slc25a29, chr34.278, YY1, Degs2, EVL, EML1* |
| 5 | 11791826 | 12965073 | 31.1023 | *DIO3, DLK1, BEGAIN, WDR25, WARS, Slc25a47, Slc25a29, chr34.278, YY1, Degs2, EVL, EML1* |
| 5 | 11800344 | 12949586 | 35.5426 | *DIO3, DLK1, BEGAIN, WDR25, WARS, Slc25a47, Slc25a29, chr34.278, YY1, Degs2, EVL, EML1* |
| 5 | 11810545 | 12938187 | 36.0326 | *DIO3, DLK1, BEGAIN, WDR25, WARS, Slc25a47, Slc25a29, chr34.278, YY1, Degs2, EVL, EML1* |
| 5 | 11810545 | 12949586 | 28.9675 | *DIO3, DLK1, BEGAIN, WDR25, WARS, Slc25a47, Slc25a29, chr34.278, YY1, Degs2, EVL, EML1* |
| 5 | 11822077 | 12343791 | 18.6634 | *DLK1* |
| 5 | 11827157 | 12933286 | 26.7421 | *DLK1, BEGAIN, WDR25, WARS, Slc25a47, Slc25a29, chr34.278, YY1, Degs2, EVL, EML1* |
| 5 | 11839710 | 12923231 | 24.4356 | *DLK1, BEGAIN, WDR25, WARS, Slc25a47, Slc25a29, chr34.278, YY1, Degs2, EVL, EML1* |
| 5 | 11840540 | 12905467 | 35.8940 | *DLK1, BEGAIN, WDR25, WARS, Slc25a47, Slc25a29, chr34.278, YY1, Degs2, EVL, EML1* |
| 5 | 11848759 | 12711622 | 33.8847 | *DLK1, BEGAIN, WDR25, WARS, Slc25a47, Slc25a29, chr34.278, YY1, Degs2, EVL* |
| 5 | 11862146 | 12578965 | 24.1329 | *DLK1, BEGAIN, WDR25* |
| 5 | 11867965 | 12896109 | 22.1341 | *DLK1, BEGAIN, WDR25, WARS, Slc25a47, Slc25a29, chr34.278, YY1, Degs2, EVL, EML1* |
| 5 | 11881459 | 12863790 | 35.0450 | *DLK1, BEGAIN, WDR25, WARS, Slc25a47, Slc25a29, chr34.278, YY1, Degs2, EVL, EML1* |
| 5 | 11890616 | 12880102 | 19.8440 | *DLK1, BEGAIN, WDR25, WARS, Slc25a47, Slc25a29, chr34.278, YY1, Degs2, EVL, EML1* |
| 5 | 11899757 | 12322647 | 12.5848 | *DLK1* |
| 5 | 11899757 | 12658979 | 25.3378 | *DLK1, BEGAIN, WDR25, WARS, Slc25a47, Slc25a29, chr34.278, YY1* |
| 5 | 11907239 | 12442116 | 28.7657 | *DLK1, BEGAIN* |
| 5 | 11910593 | 12857808 | 17.5850 | *DLK1, BEGAIN, WDR25, WARS, Slc25a47, Slc25a29, chr34.278, YY1, Degs2, EVL, EML1* |
| 5 | 11912232 | 12442116 | 28.7826 | *DLK1, BEGAIN* |
| 5 | 11914353 | 12828311 | 33.3098 | *DLK1, BEGAIN, WDR25, WARS, Slc25a47, Slc25a29, chr34.278, YY1, Degs2, EVL* |
| 5 | 11924995 | 12643261 | 12.3873 | *DLK1, BEGAIN, WDR25, WARS, Slc25a47, Slc25a29, chr34.278, YY1* |
| 5 | 11929525 | 12238631 | 10.1892 | *DLK1* |
| 5 | 11935894 | 12837751 | 15.3423 | *DLK1, BEGAIN, WDR25, WARS, Slc25a47, Slc25a29, chr34.278, YY1, Degs2, EVL* |
| 5 | 11942948 | 12281071 | 8.6345 | *DLK1* |
| 5 | 11945908 | 12365357 | 8.0776 | *DLK1* |
| 5 | 11954301 | 12786923 | 30.2532 | *DLK1, BEGAIN, WDR25, WARS, Slc25a47, Slc25a29, chr34.278, YY1, Degs2, EVL* |
| 5 | 11956115 | 12816070 | 13.1150 | *DLK1, BEGAIN, WDR25, WARS, Slc25a47, Slc25a29, chr34.278, YY1, Degs2, EVL* |
| 5 | 11959610 | 12700273 | 35.8423 | *DLK1, BEGAIN, WDR25, WARS, Slc25a47, Slc25a29, chr34.278, YY1, Degs2, EVL* |
| 5 | 11976039 | 12365357 | 20.0517 | *DLK1* |
| 5 | 11976039 | 12690832 | 34.9727 | *DLK1, BEGAIN, WDR25, WARS, Slc25a47, Slc25a29, chr34.278, YY1, Degs2* |
| 5 | 11981308 | 12793844 | 10.9114 | *DLK1, BEGAIN, WDR25, WARS, Slc25a47, Slc25a29, chr34.278, YY1, Degs2, EVL* |
| 5 | 11984357 | 12548110 | 24.4054 | *DLK1, BEGAIN, WDR25* |
| 5 | 11985099 | 12365357 | 18.5957 | *DLK1* |
| 5 | 11999356 | 12740495 | 25.2366 | *DLK1, BEGAIN, WDR25, WARS, Slc25a47, Slc25a29, chr34.278, YY1, Degs2, EVL* |
| 5 | 12005388 | 12650849 | 31.6517 | *DLK1, BEGAIN, WDR25, WARS, Slc25a47, Slc25a29, chr34.278, YY1* |
| 5 | 12005455 | 12770500 | 8.7622 | *DLK1, BEGAIN, WDR25, WARS, Slc25a47, Slc25a29, chr34.278, YY1, Degs2, EVL* |
| 5 | 12016922 | 12516185 | 22.8412 | *DLK1, BEGAIN, WDR25* |
| 5 | 12022348 | 12646836 | 32.4297 | *DLK1, BEGAIN, WDR25, WARS, Slc25a47, Slc25a29, chr34.278, YY1* |
| 5 | 12072560 | 12663585 | 17.7237 | *DLK1, BEGAIN, WDR25, WARS, Slc25a47, Slc25a29, chr34.278, YY1* |
| 5 | 12086727 | 12365357 | 15.8809 | *DLK1* |
| 5 | 12094712 | 12319743 | 12.6619 | *DLK1* |
| 5 | 12112823 | 12492182 | 16.8204 | *DLK1, BEGAIN* |
| 5 | 12129279 | 12339159 | 12.4184 | *DLK1* |
| 5 | 12130668 | 12257258 | 8.3758 | *DLK1* |
| 5 | 12132580 | 12351789 | 13.4405 | *DLK1* |
| 5 | 12138271 | 12274366 | 10.0426 | *DLK1* |
| 5 | 12138271 | 12348055 | 8.5966 | *DLK1* |
| 5 | 12140044 | 12442116 | 13.3100 | *DLK1, BEGAIN* |
| 5 | 12161346 | 12340807 | 16.6019 | *DLK1* |
| 5 | 12164080 | 12236854 | 9.7826 | *DLK1* |
| 5 | 12167356 | 12513201 | 13.8387 | *DLK1, BEGAIN, WDR25* |
| 5 | 12168536 | 12235425 | 8.5838 | *DLK1* |
| 5 | 12173951 | 12512958 | 14.9896 | *DLK1, BEGAIN, WDR25* |
| 5 | 12210933 | 12523032 | 14.5155 | *, BEGAIN, WDR25* |
| 5 | 12247705 | 12459710 | 12.2761 | *BEGAIN* |
| 5 | 12251592 | 12459710 | 17.0296 | *BEGAIN* |
| 5 | 12278377 | 12442116 | 7.9752 | *BEGAIN* |
| 5 | 13295997 | 13749576 | 13.2379 | *BCL11B* |
| 5 | 13312975 | 13731072 | 11.0906 | *BCL11B* |
| 5 | 13371907 | 13675815 | 7.8933 | *BCL11B* |
| 5 | 13435875 | 13607223 | 8.0766 | *BCL11B* |
| 5 | 17839691 | 17916037 | 9.7189 | *KCNK13* |
| 5 | 30841960 | 31135197 | 11.1794 | *Eps8l2, TMEM80, Deaf1, DRD4, SCT, CDHR5, BUB1B, Pak6, PLCB2* |
| 5 | 30845375 | 31127415 | 9.0236 | *Eps8l2, TMEM80, Deaf1, DRD4, SCT, CDHR5, BUB1B, Pak6, PLCB2* |
| 5 | 30846598 | 31131152 | 13.4399 | *Eps8l2, TMEM80, Deaf1, DRD4, SCT, CDHR5, BUB1B, Pak6, PLCB2* |
| 5 | 30912636 | 31085329 | 16.1998 | *TMEM80, Deaf1, DRD4, SCT, CDHR5, BUB1B, Pak6, PLCB2* |
| 5 | 31131152 | 31795366 | 35.3441 | *PLCB2, INAFM2, chr34.686, CCDC9B, DISP2, Knstrn, IVD, Bahd1, BAHD1, chr34.695, CHST14, Prlhr, Ccdc32, PCMT1, Rpusd2, KNL1, RAD51A, CYP1B1, RMDN3, Gchfr, DNAJC17, UBR1, RERG, MRPL21, IGHMBP2, Syt12, PTDSS1, chr34.713, ANO9, SIGIRR, B4GALNT4* |
| 5 | 31132867 | 31797291 | 34.7346 | *PLCB2, INAFM2, chr34.686, CCDC9B, DISP2, Knstrn, IVD, Bahd1, BAHD1, chr34.695, CHST14, Prlhr, Ccdc32, Syt12, PCMT1, Rpusd2, KNL1, RAD51A, CYP1B1, RMDN3, Gchfr, DNAJC17, UBR1, RERG, MRPL21, IGHMBP2, PTDSS1, chr34.713, ANO9, SIGIRR, B4GALNT4* |
| 5 | 31133977 | 31793986 | 35.0295 | *PLCB2, INAFM2, chr34.686, CCDC9B, DISP2, Knstrn, IVD, Bahd1, BAHD1, chr34.695, CHST14, Prlhr, Ccdc32, PCMT1, Rpusd2, KNL1, RAD51A, CYP1B1, RMDN3, Gchfr, DNAJC17, UBR1, RERG, MRPL21, IGHMBP2, Syt12, PTDSS1, chr34.713, ANO9, SIGIRR, B4GALNT4* |
| 5 | 31134243 | 31789667 | 35.7738 | *PLCB2, INAFM2, chr34.686, CCDC9B, DISP2, Knstrn, IVD, Bahd1, BAHD1, chr34.695, CHST14, Prlhr, Ccdc32, PCMT1, Rpusd2, KNL1, RAD51A, CYP1B1, RMDN3, Gchfr, DNAJC17, UBR1, RERG, MRPL21, IGHMBP2, Syt12, PTDSS1, chr34.713, ANO9, SIGIRR, B4GALNT4* |
| 5 | 31136541 | 31795259 | 34.2693 | *PLCB2, INAFM2, chr34.686, CCDC9B, DISP2, Knstrn, IVD, Bahd1, BAHD1, chr34.695, CHST14, Prlhr, Ccdc32, PCMT1, Rpusd2, KNL1, RAD51A, CYP1B1, RMDN3, Gchfr, DNAJC17, UBR1, RERG, MRPL21, IGHMBP2, Syt12, PTDSS1, chr34.713, ANO9, SIGIRR, B4GALNT4* |
| 5 | 31139189 | 31782558 | 36.3207 | *PLCB2, INAFM2, chr34.686, CCDC9B, DISP2, Knstrn, IVD, Bahd1, BAHD1, chr34.695, CHST14, Prlhr, Ccdc32, PCMT1, Rpusd2, KNL1, RAD51A, Syt12, CYP1B1, RMDN3, Gchfr, DNAJC17, UBR1, RERG, MRPL21, IGHMBP2, PTDSS1, chr34.713, ANO9, SIGIRR* |
| 5 | 31140908 | 31793160 | 33.1817 | *PLCB2, INAFM2, chr34.686, CCDC9B, DISP2, Knstrn, IVD, Bahd1, BAHD1, chr34.695, CHST14, Prlhr, Ccdc32, PCMT1, Rpusd2, KNL1, RAD51A, CYP1B1, RMDN3, Gchfr, DNAJC17, UBR1, RERG, MRPL21, IGHMBP2, Syt12, PTDSS1, chr34.713, ANO9, SIGIRR, B4GALNT4* |
| 5 | 31147959 | 31788097 | 30.3857 | *INAFM2, chr34.686, CCDC9B, DISP2, Knstrn, IVD, Bahd1, BAHD1, chr34.695, CHST14, Prlhr, Ccdc32, PCMT1, Rpusd2, KNL1, RAD51A, CYP1B1, RMDN3, Gchfr, DNAJC17, UBR1, RERG, MRPL21, IGHMBP2, Syt12, PTDSS1, chr34.713, ANO9, SIGIRR* |
| 5 | 31149413 | 31770388 | 36.9618 | *INAFM2, chr34.686, CCDC9B, DISP2, Knstrn, IVD, Bahd1, BAHD1, chr34.695, CHST14, Prlhr, Ccdc32, PCMT1, Rpusd2, KNL1, RAD51A, CYP1B1, RMDN3, Gchfr, DNAJC17, UBR1, RERG, MRPL21, IGHMBP2, Syt12, PTDSS1, chr34.713, ANO9, SIGIRR* |
| 5 | 31157362 | 31780309 | 21.2667 | *INAFM2, chr34.686, CCDC9B, DISP2, Knstrn, IVD, Bahd1, BAHD1, chr34.695, CHST14, Prlhr, Ccdc32, PCMT1, Rpusd2, KNL1, RAD51A, CYP1B1, RMDN3, Gchfr, DNAJC17, UBR1, RERG, MRPL21, IGHMBP2, Syt12, PTDSS1, chr34.713, ANO9, SIGIRR* |
| 5 | 31161798 | 31755422 | 37.5663 | *chr34.686, CCDC9B, DISP2, Knstrn, IVD, Bahd1, BAHD1, chr34.695, CHST14, Prlhr, Ccdc32, PCMT1, Rpusd2, KNL1, RAD51A, CYP1B1, RMDN3, Gchfr, DNAJC17, UBR1, RERG, MRPL21, IGHMBP2, Syt12, PTDSS1, chr34.713* |
| 5 | 31174379 | 31742102 | 37.8104 | *chr34.686, CCDC9B, DISP2, Knstrn, IVD, Bahd1, BAHD1, chr34.695, CHST14, Prlhr, Ccdc32, PCMT1, Rpusd2, KNL1, RAD51A, CYP1B1, RMDN3, Gchfr, DNAJC17, UBR1, RERG, PTDSS1, MRPL21, IGHMBP2, Syt12, chr34.713* |
| 5 | 31174379 | 31766122 | 15.4161 | *chr34.686, CCDC9B, DISP2, Knstrn, IVD, Bahd1, BAHD1, chr34.695, CHST14, Prlhr, Ccdc32, PCMT1, Rpusd2, KNL1, RAD51A, CYP1B1, RMDN3, Gchfr, DNAJC17, UBR1, RERG, MRPL21, IGHMBP2, Syt12, PTDSS1, chr34.713, ANO9* |
| 5 | 31191393 | 31722926 | 37.1296 | *CCDC9B, DISP2, Knstrn, IVD, Bahd1, BAHD1, chr34.695, CHST14, Prlhr, Ccdc32, PCMT1, Rpusd2, KNL1, RAD51A, CYP1B1, RMDN3, Gchfr, DNAJC17, UBR1, RERG, MRPL21, IGHMBP2, Syt12, PTDSS1, chr34.713* |
| 5 | 31209975 | 31700705 | 34.0916 | *CCDC9B, DISP2, Knstrn, IVD, Bahd1, BAHD1, chr34.695, CHST14, Prlhr, Ccdc32, PCMT1, Rpusd2, KNL1, RAD51A, CYP1B1, RMDN3, Gchfr, DNAJC17, UBR1, RERG, MRPL21, IGHMBP2, Syt12, PTDSS1* |
| 5 | 31239618 | 31671379 | 24.9962 | *DISP2, Knstrn, IVD, Bahd1, BAHD1, chr34.695, CHST14, Prlhr, Ccdc32, PCMT1, Rpusd2, KNL1, RAD51A, CYP1B1, RMDN3, Gchfr, DNAJC17, UBR1, RERG, MRPL21, IGHMBP2* |
| 5 | 31287641 | 31564297 | 25.1945 | *Bahd1, BAHD1, chr34.695, CHST14, Prlhr, Ccdc32, PCMT1, Rpusd2, KNL1, RAD51A, CYP1B1, RMDN3, Gchfr, DNAJC17* |
| 5 | 31291930 | 31561751 | 10.7656 | *Bahd1, BAHD1, chr34.695, CHST14, Prlhr, Ccdc32, PCMT1, Rpusd2, KNL1, RAD51A, CYP1B1, RMDN3, Gchfr, DNAJC17* |
| 5 | 31316377 | 31548956 | 12.5017 | *Bahd1, BAHD1, chr34.695, CHST14, Prlhr, Ccdc32, PCMT1, Rpusd2, KNL1, RAD51A, CYP1B1, RMDN3, Gchfr, DNAJC17* |
| 5 | 31321717 | 31527924 | 9.7966 | *Bahd1, BAHD1, chr34.695, CHST14, Prlhr, Ccdc32, PCMT1, Rpusd2, KNL1, RAD51A, CYP1B1, RMDN3* |
| 5 | 31358700 | 31607180 | 10.5149 | *CHST14, Prlhr, Ccdc32, PCMT1, Rpusd2, KNL1, RAD51A, CYP1B1, RMDN3, Gchfr, DNAJC17, UBR1* |
| 5 | 31365173 | 31602777 | 8.5762 | *Prlhr, Ccdc32, PCMT1, Rpusd2, KNL1, RAD51A, CYP1B1, RMDN3, Gchfr, DNAJC17, UBR1* |
| 5 | 31366251 | 31597919 | 10.2463 | *Prlhr, Ccdc32, PCMT1, Rpusd2, KNL1, RAD51A, CYP1B1, RMDN3, Gchfr, DNAJC17, UBR1* |
| 5 | 31369390 | 31520361 | 8.0276 | *Prlhr, Ccdc32, PCMT1, Rpusd2, KNL1, RAD51A, CYP1B1, RMDN3* |
| 5 | 31394837 | 31550821 | 12.9870 | *Ccdc32, PCMT1, Rpusd2, KNL1, RAD51A, CYP1B1, RMDN3, Gchfr, DNAJC17* |
| 5 | 31406937 | 31453807 | 14.9508 | *PCMT1, Rpusd2, KNL1* |
| 5 | 31421569 | 31476282 | 9.8331 | *KNL1, RAD51A, CYP1B1* |
| 5 | 31469247 | 31497519 | 8.6089 | *CYP1B1, RMDN3* |
| 5 | 32121606 | 32350193 | 11.1085 | *LRP4, CKAP5, F2, Arhgap1, chr34.749, ATG13chr34.752, chr34.753, AMBRA1* |
| 5 | 32155703 | 32317931 | 7.9850 | *CKAP5, F2, Arhgap1, chr34.749, ATG13, chr34.752, chr34.753, AMBRA1* |
| 5 | 32600017 | 35579195 | 16.6424 | *PHF21A, LARGE2, chr34.762, Pex16, C11orf94, Mapk8ip1, CRY2, Slc35c1, ASTL, PGF, Zfyve19, PPP1R14D, Spint1, Rhov, vps18, DLL4, CHAC1, Ino80, Exd1, CHP1, chr34.786, OIP5, NUSAP1, NDUFAF1, Rtf1, chr34.791, ALK, MGA, RPAP1, tmem151b, TYRO3, mapkbp1, chr34.801, Jmjd7, ACOT1, Pla2g4b, SPTBN5, EHD4, PLA2G4E, VPS39, Tmem87a, GANC, CAPN3, ZNF106, Snap23, Lrrc57, HAUS2, DPF3, STARD9, chr34.823, Cdan1, TSNARE1, TTBK2, Slc35c1, UBR1, TMEM62, PTGR2, ELMSAN1, Acot3, ACOT1, RIOX1, NUMB, chr34.840, PAPLN, PSEN1, RBM25, ZFYVE1, DCAF4* |
| 5 | 32653168 | 35521676 | 31.6627 | *PHF21A, LARGE2, chr34.762, Pex16, C11orf94, Mapk8ip1, CRY2, Slc35c1, ASTL, PGF, Zfyve19, PPP1R14D, Spint1, Rhov, vps18, DLL4, CHAC1, Ino80, Exd1, CHP1, chr34.786, OIP5, NUSAP1, NDUFAF1, Rtf1, chr34.791, chr34.823* |
|  |  |  |  | *Chst8, ITPKA, ALK, RPAP1, tmem151b, TYRO3, MGA, mapkbp1, chr34.801, Jmjd7, ACOT1, Pla2g4b, SPTBN5, EHD4, PLA2G4E, VPS39, Tmem87a, GANC, CAPN3, ZNF106, Snap23, Lrrc57, HAUS2, STARD9, Cdan1, TSNARE1, TTBK2, Slc35c1, UBR1, TMEM62, PTGR2, ELMSAN1, Acot3, ACOT1, RIOX1, NUMB, chr34.840, PAPLN, PSEN1, RBM25, ZFYVE1, DCAF4, DPF3* |
| 5 | 32654889 | 35521676 | 26.1052 | *PHF21A, LARGE2, chr34.762, Pex16, C11orf94, Mapk8ip1, CRY2, Slc35c1, ASTL, PGF, Zfyve19, PPP1R14D, Spint1, Rhov, vps18, DLL4, CHAC1, Ino80, Exd1, CHP1, chr34.786, OIP5, NUSAP1, NDUFAF1, Rtf1, chr34.791, chr34.823, Chst8, ITPKA, ALK, RPAP1, tmem151b, TYRO3, MGA, mapkbp1, chr34.801, Jmjd7, Cdan1, TSNARE1, TTBK2, Slc35c1, UBR1, TMEM62, PTGR2, ELMSAN1, Acot3, ACOT1, RIOX1, NUMB, chr34.840, PAPLN, PSEN1, RBM25, ZFYVE1, DCAF4, DPF3* |
| 5 | 32680824 | 35492745 | 34.8062 | *PHF21A, LARGE2, chr34.762, Pex16, C11orf94, Mapk8ip1, CRY2, Slc35c1, ASTL, PGF, Zfyve19, PPP1R14D, Spint1, Rhov, vps18, DLL4, CHAC1, Ino80, Exd1, CHP1, chr34.786, OIP5, NUSAP1, NDUFAF1, Rtf1, chr34.791, chr34.823, Chst8, ITPKA, ALK, RPAP1, tmem151b, TYRO3, MGA, mapkbp1, chr34.801, Jmjd7, ACOT1, Pla2g4b, SPTBN5, EHD4, PLA2G4E, Pla2g4e, VPS39, Tmem87a, GANC, CAPN3, ZNF106, Snap23, Lrrc57, HAUS2, STARD9, Cdan1, TSNARE1, TTBK2, Slc35c1, UBR1, TMEM62, PTGR2, ELMSAN1, Acot3, ACOT1, RIOX1, NUMB, chr34.840, PAPLN, PSEN1, RBM25, ZFYVE1, DCAF4* |
| 5 | 32710769 | 35461310 | 36.1729 | *Pex16, C11orf94, Mapk8ip1, CRY2, Slc35c1, ASTL, PGF, Zfyve19, PPP1R14D, Spint1, Rhov, vps18, DLL4, CHAC1, Ino80, Exd1, CHP1, chr34.786, OIP5, NUSAP1, NDUFAF1, Rtf1, chr34.791, chr34.823, Chst8, ITPKA, ALK, RPAP1, tmem151b, TYRO3, MGA, mapkbp1, chr34.801, Jmjd7, ACOT1, Pla2g4b, SPTBN5, EHD4, PLA2G4E, Pla2g4e, VPS39, Tmem87a, GANC, CAPN3, ZNF106, Snap23, Lrrc57, HAUS2, STARD9, TSNARE1, Cdan1, TTBK2, Slc35c1, UBR1, TMEM62, PTGR2, ELMSAN1, Acot3, ACOT1, RIOX1, NUMB, chr34.840, PAPLN, PSEN1, RBM25, ZFYVE1* |
| 5 | 32778121 | 35391766 | 35.9348 | *ASTL, PGF, Zfyve19, PPP1R14D, Spint1, Rhov, vps18, DLL4, CHAC1, Ino80, Exd1, CHP1, chr34.786, OIP5, NUSAP1, NDUFAF1, Rtf1, chr34.791, Chst8, ITPKA, ALK, RPAP1, tmem151b, TYRO3, MGA, mapkbp1, chr34.801, Jmjd7, Acot3, ACOT1, Pla2g4b, SPTBN5, EHD4, PLA2G4E, Pla2g4e, VPS39, Tmem87a, GANC, CAPN3, ZNF106, Snap23, Lrrc57, HAUS2, STARD9, chr34.823, Cdan1, TSNARE1, TTBK2, Slc35c1, UBR1, TMEM62, PTGR2, ELMSAN1, PSEN1, RIOX1, NUMB, chr34.840, PAPLN* |
| 5 | 32802518 | 35365456 | 34.1167 | *ASTL, PGF, Zfyve19, PPP1R14D, Spint1, Rhov, vps18, DLL4, CHAC1, Ino80, Exd1, CHP1, chr34.786, OIP5, NUSAP1, NDUFAF1, Rtf1, chr34.791, Chst8, ITPKA, ALK, RPAP1, tmem151b, TYRO3, MGA, mapkbp1, chr34.801, ACOT1, Pla2g4b, SPTBN5, EHD4, PLA2G4E, Pla2g4e, VPS39, Tmem87a, GANC, CAPN3, ZNF106, Snap23, Lrrc57, HAUS2, STARD9, chr34.823, Cdan1, TSNARE1, TTBK2, Slc35c1, UBR1, TMEM62, PTGR2, ELMSAN1, Acot3, ACOT1, RIOX1, NUMB, chr34.840, PAPLN* |
| 5 | 32806179 | 35358893 | 30.6488 | *ASTL, PGF, Zfyve19, PPP1R14D, Spint1, Rhov, vps18, DLL4, CHAC1, Ino80, Exd1, CHP1, chr34.786, OIP5, NUSAP1, NDUFAF1, Rtf1, chr34.791, Chst8, ITPKA, ALK, RPAP1, tmem151b, TYRO3, MGA, mapkbp1, chr34.801, Jmjd7, Acot3, ACOT1, Pla2g4b, SPTBN5, EHD4, PLA2G4E, Pla2g4e, VPS39, Tmem87a, GANC, CAPN3, ZNF106, Snap23, Lrrc57, HAUS2, STARD9, chr34.823, Cdan1, TSNARE1, TTBK2, Slc35c1, UBR1, TMEM62, PTGR2, ELMSAN1, RIOX1, NUMB, chr34.840, PAPLN* |
| 5 | 32840708 | 35323090 | 25.2093 | *ASTL, PGF, Zfyve19, PPP1R14D, Spint1, Rhov, vps18, DLL4, CHAC1, Ino80, Exd1, CHP1, chr34.786, OIP5, NUSAP1, NDUFAF1, Rtf1, chr34.791, Chst8, ITPKA, ALK, RPAP1, tmem151b, TYRO3, MGA, mapkbp1, chr34.801, Jmjd7, Acot3, ACOT1, Pla2g4b, SPTBN5, EHD4, PLA2G4E, Pla2g4e, VPS39, Tmem87a, GANC, CAPN3, ZNF106, Snap23, Lrrc57, HAUS2, STARD9, chr34.823, Cdan1, TSNARE1, TTBK2, Slc35c1, UBR1, TMEM62, PTGR2, ELMSAN1, RIOX1, NUMB, chr34.840, PAPLN* |
| 5 | 32881937 | 35274175 | 17.1255 | *ASTL, PGF, Zfyve19, PPP1R14D, Spint1, Rhov, vps18, DLL4, CHAC1, Ino80, Exd1, CHP1, chr34.786, OIP5, NUSAP1, NDUFAF1, Rtf1, chr34.791, Chst8, ITPKA, ALK, RPAP1, tmem151b, TYRO3, MGA, mapkbp1, chr34.801, Jmjd7, Acot3, ACOT1, Pla2g4b, SPTBN5, EHD4, PLA2G4E, Pla2g4e, VPS39, Tmem87a, GANC, CAPN3, ZNF106, Snap23, Lrrc57, HAUS2, STARD9, chr34.823, Cdan1, TSNARE1, TTBK2, Slc35c1, UBR1, TMEM62, PTGR2, ELMSAN1, RIOX1, NUMB, chr34.840* |
| 5 | 33393830 | 34706001 | 9.3479 | *CHP1, chr34.786, OIP5, NUSAP1, NDUFAF1, Rtf1, chr34.791, Chst8, ITPKA, ALK, RPAP1, tmem151b, TYRO3, MGA, mapkbp1, chr34.801, Jmjd7, ACOT1, Pla2g4b, SPTBN5, EHD4, PLA2G4E, PLA2G4F, VPS39, Tmem87a, GANC, CAPN3, ZNF106, Snap23, Lrrc57, HAUS2* |
| 5 | 33404715 | 34693269 | 17.4432 | *OIP5, NUSAP1, NDUFAF1, Rtf1, chr34.791, Chst8, ITPKA, ALK, RPAP1, tmem151b, TYRO3, MGA, mapkbp1, chr34.801, Jmjd7, ACOT1, Pla2g4b, SPTBN5, EHD4, PLA2G4E, PLA2G4F, VPS39, Tmem87a, GANC, CAPN3, ZNF106, Snap23* |
| 5 | 33448251 | 34647375 | 17.2082 | *Rtf1, chr34.791, Chst8, ITPKA, ALK, RPAP1, tmem151b, TYRO3, MGA, mapkbp1, chr34.801, Jmjd7, ACOT1, Pla2g4b, SPTBN5, EHD4, PLA2G4E, PLA2G4F, VPS39, Tmem87a, GANC, CAPN3, ZNF106* |
| 5 | 33637860 | 34453867 | 13.5981 | *ALK, RPAP1, tmem151b, TYRO3, MGA, mapkbp1, chr34.801, Jmjd7, ACOT1, Pla2g4b, SPTBN5, EHD4, PLA2G4E* |
| 5 | 33753799 | 34336402 | 14.2537 | *RPAP1, tmem151b, TYRO3, MGA, mapkbp1, chr34.801, Jmjd7, ACOT1, Pla2g4b, SPTBN5, EHD4, PLA2G4E* |
| 5 | 33798637 | 34249542 | 22.4177 | *tmem151b, TYRO3, MGA, mapkbp1, chr34.801, Jmjd7, ACOT1, Pla2g4b, SPTBN5, EHD4, PLA2G4E* |
| 5 | 33808154 | 34259633 | 10.7433 | *TYRO3, MGA, mapkbp1, chr34.801, Jmjd7, ACOT1, Pla2g4b, SPTBN5, EHD4, PLA2G4E* |
| 5 | 33809542 | 34132166 | 15.8742 | *TYRO3, MGA, mapkbp1, chr34.801, Jmjd7, ACOT1, Pla2g4b, SPTBN5* |
| 5 | 33811552 | 34234235 | 12.5818 | *TYRO3, MGA, mapkbp1, chr34.801, Jmjd7, ACOT1, Pla2g4b, SPTBN5, EHD4* |
| 5 | 33853912 | 34194690 | 12.9933 | *MGA, mapkbp1, chr34.801, Jmjd7, ACOT1, Pla2g4b, SPTBN5* |
| 5 | 33878909 | 34064773 | 13.4042 | *MGA, mapkbp1, chr34.801, Jmjd7, ACOT1* |
| 5 | 33899025 | 34130475 | 11.0556 | *MGA, mapkbp1, chr34.801, Jmjd7, ACOT1, Pla2g4b, SPTBN5* |
| 5 | 33899025 | 34616917 | 24.8937 | *MGA, mapkbp1, chr34.801, Jmjd7, ACOT1, Pla2g4b, SPTBN5, EHD4, PLA2G4E, PLA2G4F, VPS39, Tmem87a, GANC, CAPN3* |
| 5 | 33903886 | 34607436 | 22.8582 | *MGA, mapkbp1, chr34.801, Jmjd7, ACOT1, Pla2g4b, SPTBN5, EHD4, PLA2G4E, PLA2G4F, VPS39, Tmem87a, GANC, CAPN3* |
| 5 | 33911804 | 34176106 | 11.3554 | *MGA, mapkbp1, chr34.801, Jmjd7, ACOT1, Pla2g4b, SPTBN5* |
| 5 | 33921983 | 34592813 | 13.2829 | *MGA, mapkbp1, chr34.801, Jmjd7, ACOT1, Pla2g4b, SPTBN5, EHD4, PLA2G4E, PLA2G4F, VPS39, Tmem87a, GANC* |
| 5 | 33924885 | 34102384 | 10.1038 | *mapkbp1, chr34.801, Jmjd7, ACOT1, Pla2g4b* |
| 5 | 33928790 | 34054856 | 7.8283 | *mapkbp1, chr34.801, Jmjd7, ACOT1* |
| 5 | 33979254 | 34104294 | 10.3925 | *mapkbp1, chr34.801, Jmjd7, ACOT1, Pla2g4b* |
| 5 | 33988465 | 34093001 | 9.5518 | *mapkbp1, chr34.801, Jmjd7, ACOT1, Pla2g4b* |
| 5 | 34120576 | 34429179 | 7.7883 | *SPTBN5, EHD4, PLA2G4E* |
| 5 | 34126604 | 34321547 | 8.9684 | *SPTBN5, EHD4, PLA2G4E* |
| 5 | 34137147 | 34415030 | 8.3115 | *SPTBN5, EHD4, PLA2G4E* |
| 5 | 34147054 | 34515528 | 10.4927 | *SPTBN5, EHD4, PLA2G4E, PLA2G4F* |
| 5 | 34170289 | 34688000 | 11.2322 | *SPTBN5, EHD4, PLA2G4E, PLA2G4F, VPS39, Tmem87a, GANC, CAPN3, ZNF106, Snap23* |
| 5 | 34184002 | 34673245 | 28.5177 | *SPTBN5, EHD4, PLA2G4E, PLA2G4F, VPS39, Tmem87a, GANC, CAPN3, ZNF106* |
| 5 | 34186493 | 34340669 | 8.7091 | *SPTBN5, EHD4, PLA2G4E* |
| 5 | 34244289 | 34610560 | 9.3773 | *EHD4, PLA2G4E, PLA2G4F, VPS39, Tmem87a, GANC, CAPN3* |
| 5 | 34247843 | 34558145 | 20.9561 | *PLA2G4E, PLA2G4F, VPS39, Tmem87a, VPS39* |
| 5 | 34257255 | 34551145 | 20.8785 | *PLA2G4E, PLA2G4F, VPS39* |
| 5 | 34347835 | 34490002 | 19.2746 | *PLA2G4E* |
| 5 | 34352293 | 34735405 | 7.7329 | *PLA2G4E, PLA2G4F, VPS39, Tmem87a, GANC, CAPN3, ZNF106, Snap23, Lrrc57, HAUS2, STARD9* |
| 5 | 34357581 | 34560106 | 10.1317 | *PLA2G4F, VPS39, Tmem87a* |
| 5 | 34378790 | 34457613 | 9.0984 | *Pla2g4e* |
| 5 | 34383755 | 34601825 | 8.5673 | *Pla2g4e, PLA2G4F, VPS39, Tmem87a, GANC* |
| 5 | 34425700 | 34501225 | 9.9036 | *Pla2g4e* |
| 5 | 34434011 | 34608402 | 9.7647 | *Pla2g4e, PLA2G4F, VPS39, Tmem87a, GANC, CAPN3* |
| 5 | 34434487 | 34610936 | 9.0594 | *Pla2g4e, PLA2G4F, VPS39, Tmem87a, GANC, CAPN3* |
| 5 | 34895201 | 35234438 | 8.5345 | *Ttbk2, Slc35c1, UBR1, Tmem62, PTGR2, ELMSAN1, Acot3, ACOT1, RIOX1, NUMB, chr34.840* |
| 5 | 34898496 | 35233364 | 9.5055 | *Ttbk2, Slc35c1, UBR1, Tmem62, PTGR2, ELMSAN1, Acot3, ACOT1, RIOX1, NUMB, chr34.840* |
| 5 | 38733271 | 38770303 | 7.8689 | *SRP14, EIF2AK4* |
| 5 | 47172782 | 47244685 | 8.4156 | *PPP6R3, chr34.994* |
| 5 | 52821398 | 53022151 | 10.0805 | *CD82* |
| 5 | 56905531 | 57896003 | 18.1911 | *FIBIN, Bbox1, CCDC34, LGR4, LIN7C, BDNF, KIF18A, METTL15* |
| 5 | 56909988 | 57889346 | 23.1682 | *FIBIN, Bbox1, CCDC34, LGR4, LIN7C, BDNF, KIF18A, METTL15* |
| 5 | 56925289 | 57872030 | 23.8517 | *FIBIN, Bbox1, CCDC34, LGR4, LIN7C, BDNF, KIF18A, METTL15* |
| 5 | 56930860 | 57808066 | 18.0298 | *FIBIN, Bbox1, CCDC34, LGR4, LIN7C, BDNF, KIF18A, METTL15* |
| 5 | 56934008 | 57803335 | 17.8500 | *Bbox1, CCDC34, LGR4, LIN7C, BDNF, KIF18A, METTL15* |
| 5 | 56936179 | 57805263 | 17.6396 | *Bbox1, CCDC34, LGR4, LIN7C, BDNF, KIF18A, METTL15* |
| 5 | 56939660 | 57795767 | 17.1904 | *Bbox1, CCDC34, LGR4, LIN7C, BDNF, KIF18A, METTL15* |
| 5 | 56940794 | 57803335 | 16.4424 | *Bbox1, CCDC34, LGR4, LIN7C, BDNF, KIF18A, METTL15* |
| 5 | 56946602 | 57798611 | 13.8700 | *Bbox1, CCDC34, LGR4, LIN7C, BDNF, KIF18A, METTL15* |
| 5 | 56951946 | 57781607 | 16.0371 | *Bbox1, CCDC34, LGR4, LIN7C, BDNF, KIF18A, METTL15* |
| 5 | 56961007 | 57834537 | 21.0608 | *Bbox1, CCDC34, LGR4, LIN7C, BDNF, KIF18A, METTL15* |
| 5 | 56966812 | 57764694 | 14.2054 | *Bbox1, CCDC34, LGR4, LIN7C, BDNF, KIF18A, METTL15* |
| 5 | 56966812 | 57780948 | 8.5324 | *Bbox1, CCDC34, LGR4, LIN7C, BDNF, KIF18A, METTL15* |
| 5 | 56985344 | 57743820 | 11.3019 | *Bbox1, CCDC34, LGR4, LIN7C, BDNF, KIF18A, METTL15* |
| 5 | 57049783 | 57743820 | 15.0327 | *CCDC34, LGR4, LIN7C, BDNF, KIF18A, METTL15* |
| 5 | 57127240 | 57574197 | 8.7862 | *LGR4, LIN7C, BDNF, KIF18A, METTL15* |
| 5 | 57128693 | 57574539 | 7.6394 | *LGR4, LIN7C, BDNF, KIF18A, METTL15* |
| 5 | 57142051 | 57507938 | 16.7867 | *LGR4, LIN7C, BDNF, KIF18A, METTL15* |
| 5 | 57145741 | 57507477 | 15.2014 | *LGR4, LIN7C, BDNF, KIF18A, METTL15* |
| 5 | 57148439 | 57501162 | 15.9904 | *LGR4, LIN7C, BDNF, KIF18A, METTL15* |
| 5 | 57152197 | 57530793 | 12.4605 | *LGR4, LIN7C, BDNF, KIF18A, METTL15* |
| 5 | 57165079 | 57515093 | 8.8037 | *LGR4, LIN7C, BDNF, KIF18A, METTL15* |
| 5 | 57167894 | 57479250 | 11.9999 | *LGR4, LIN7C, BDNF, KIF18A, METTL15* |
| 5 | 57174682 | 57480788 | 8.6315 | *LIN7C, BDNF, KIF18A, METTL15* |
| 5 | 57184968 | 57715297 | 7.9358 | *LIN7C, BDNF, KIF18A, METTL15* |
| 5 | 57208810 | 57477199 | 8.4012 | *BDNF, KIF18A, METTL15* |
| 5 | 57244661 | 57525613 | 9.9262 | *KIF18A, METTL15* |
| 5 | 57248706 | 57519592 | 8.3331 | *KIF18A, METTL15* |
| 5 | 57268318 | 57525613 | 8.3424 | *KIF18A, METTL15* |
| 5 | 57430946 | 57678788 | 10.3763 | *METTL15* |
| 5 | 57436748 | 57674989 | 8.2126 | *METTL15* |
| 5 | 57446155 | 57649766 | 7.6487 | *METTL15* |
| 5 | 57447849 | 57584471 | 13.0619 | *METTL15* |
| 5 | 57450495 | 57653710 | 8.0736 | *METTL15* |
| 5 | 57452865 | 57564645 | 9.4556 | *METTL15* |
| 5 | 57457768 | 57657691 | 8.5694 | *METTL15* |
| 5 | 57460851 | 57624886 | 11.4295 | *METTL15* |
| 6 | 3484313 | 4161040 | 7.8379 | *ZEB2, GTDC1* |
| 6 | 3486946 | 4156395 | 8.2661 | *ZEB2, GTDC1* |
| 6 | 3495037 | 4147227 | 8.7794 | *ZEB2, GTDC1* |
| 6 | 3496155 | 4142961 | 9.3914 | *ZEB2, GTDC1* |
| 6 | 3511163 | 4125968 | 10.1174 | *ZEB2, GTDC1* |
| 6 | 3524879 | 4111140 | 10.9198 | *ZEB2* |
| 6 | 3541139 | 4091779 | 11.7900 | *ZEB2* |
| 6 | 3563413 | 4068265 | 12.5623 | *ZEB2* |
| 6 | 3570953 | 4006176 | 9.8399 | *ZEB2* |
| 6 | 3571562 | 4007370 | 12.5593 | *ZEB2* |
| 6 | 3573734 | 4001427 | 14.8532 | *ZEB2* |
| 6 | 3583266 | 4045779 | 12.7330 | *ZEB2* |
| 6 | 3585173 | 3996044 | 13.7666 | *ZEB2* |
| 6 | 3589075 | 3984106 | 13.6331 | *ZEB2* |
| 6 | 3614874 | 4012305 | 10.8319 | *ZEB2* |
| 6 | 6379224 | 6454232 | 8.6560 | *cyp27c1, THSD7B* |
| 6 | 18010836 | 18215670 | 12.1572 | *DPP4, FAP, KCNH7* |
| 6 | 18031788 | 18219358 | 10.2087 | *DPP4, FAP, KCNH7* |
| 6 | 18043893 | 18187259 | 12.5993 | *DPP4, FAP, KCNH7* |
| 6 | 18091438 | 18144384 | 8.0307 | *FAP* |
| 7 | 15963742 | 16472645 | 24.0422 | *Prkg1, A1CF, ASAH2, SGMS1, chr32.228, MINPP1, PAPSS2, ATAD1, PTEN, RNLS* |
| 7 | 15964285 | 16473581 | 24.2864 | *Prkg1, A1CF, ASAH2, SGMS1, chr32.228, MINPP1, PAPSS2, ATAD1, PTEN, RNLS* |
| 7 | 15974571 | 16459786 | 20.0465 | *A1CF, ASAH2, SGMS1, chr32.228, MINPP1, PAPSS2, ATAD1, PTEN, RNLS* |
| 7 | 15977301 | 16463063 | 19.4066 | *A1CF, ASAH2, SGMS1, chr32.228, MINPP1, PAPSS2, ATAD1, PTEN, RNLS* |
| 7 | 15978430 | 16063080 | 8.1941 | *A1CF, ASAH2, SGMS1* |
| 7 | 15979771 | 16063080 | 8.8011 | *A1CF, ASAH2, SGMS1* |
| 7 | 15989151 | 16443154 | 9.1968 | *Prkg1, A1CF, ASAH2, SGMS1, chr32.228, MINPP1, PAPSS2, ATAD1, PTEN, RNLS* |
| 7 | 16029535 | 16388803 | 7.7949 | *ASAH2, SGMS1, chr32.228, MINPP1, PAPSS2, ATAD1, PTEN* |
| 7 | 16032851 | 16384158 | 8.2418 | *ASAH2, SGMS1, chr32.228, MINPP1, PAPSS2, ATAD1, PTEN* |
| 7 | 16034142 | 16464629 | 21.3286 | *ASAH2, SGMS1, chr32.228, MINPP1, PAPSS2, ATAD1, PTEN, RNLS* |
| 7 | 16036576 | 16460697 | 13.6023 | *ASAH2, SGMS1, chr32.228, MINPP1, PAPSS2, ATAD1, PTEN, RNLS* |
| 7 | 16037794 | 16464514 | 22.2299 | *ASAH2, SGMS1, chr32.228, MINPP1, PAPSS2, ATAD1, PTEN, RNLS* |
| 7 | 16047460 | 16457064 | 21.0289 | *ASAH2, SGMS1, chr32.228, MINPP1, PAPSS2, ATAD1, PTEN, RNLS* |
| 7 | 16049960 | 16442106 | 11.6968 | *ASAH2, SGMS1, chr32.228, MINPP1, PAPSS2, ATAD1, PTEN, RNLS* |
| 7 | 16060704 | 16446083 | 11.4564 | *SGMS1, chr32.228, MINPP1, PAPSS2, ATAD1, PTEN, RNLS* |
| 7 | 16066356 | 16386832 | 13.9494 | *SGMS1, chr32.228, MINPP1, PAPSS2, ATAD1, PTEN* |
| 7 | 16066356 | 16419854 | 16.6196 | *SGMS1, chr32.228, MINPP1, PAPSS2, ATAD1, PTEN, RNLS* |
| 7 | 16081614 | 16428716 | 13.9498 | *SGMS1, chr32.228, MINPP1, PAPSS2, ATAD1, PTEN, RNLS* |
| 7 | 16089092 | 16394947 | 15.7554 | *SGMS1, chr32.228, MINPP1, PAPSS2, ATAD1, PTEN* |
| 7 | 16103209 | 16347392 | 9.2019 | *SGMS1, chr32.228, MINPP1, PAPSS2, ATAD1, PTEN* |
| 7 | 16106513 | 16347792 | 10.4345 | *SGMS1, chr32.228, MINPP1, PAPSS2, ATAD1, PTEN* |
| 7 | 16138653 | 16317451 | 9.8037 | *SGMS1, chr32.228, MINPP1, PAPSS2, ATAD1, PTEN* |
| 7 | 16139859 | 16378361 | 13.5974 | *SGMS1, chr32.228, MINPP1, PAPSS2, ATAD1, PTEN* |
| 7 | 16172229 | 16310051 | 8.0836 | *MINPP1, PAPSS2, ATAD1, PTEN* |
| 7 | 16177861 | 16291852 | 11.9067 | *MINPP1, PAPSS2, ATAD1, PTEN* |
| 7 | 16181528 | 16350497 | 17.6508 | *MINPP1, PAPSS2, ATAD1, PTEN* |
| 7 | 16186886 | 16343672 | 12.9775 | *MINPP1, PAPSS2, ATAD1, PTEN* |
| 7 | 16191769 | 16328805 | 10.7101 | *MINPP1, PAPSS2, ATAD1, PTEN* |
| 7 | 16203619 | 16276940 | 7.9419 | *PAPSS2, ATAD1, PTEN* |
| 7 | 29758550 | 30238773 | 20.1274 | *Atrnl1, chr32.552, chr32.553, GFRA1* |
| 7 | 29773539 | 30222778 | 11.9558 | *Atrnl1, chr32.552, chr32.553, GFRA1* |
| 7 | 29778532 | 30220649 | 17.0296 | *Atrnl1, chr32.552, chr32.553, GFRA1* |
| 7 | 29788071 | 30171252 | 7.7497 | *Atrnl1, chr32.552* |
| 7 | 29788071 | 30173147 | 11.9805 | *Atrnl1, chr32.552, chr32.553* |
| 7 | 29832288 | 30161720 | 11.2350 | *Atrnl1, chr32.552* |
| 7 | 29854547 | 30136631 | 11.3426 | *Atrnl1* |
| 7 | 29862507 | 30140930 | 8.6081 | *Atrnl1* |
| 8 | 400802 | 1618928 | 12.7940 | *ZNF644, HFM1, CDC7, TGFBR3, BRDT, EPHX4, KIAA1107, GLMN, RPAP2, GFI1, EVI5L, RPL5, DIPK1A, MTF2, TMED5, CCDC18, RDH8, DR1, Fnbp1l, BCAR3, DNTTIP2, GCLM, TECR, ABCA4, arhgap29, Abcd3, F3* |
| 8 | 400802 | 1622180 | 9.5290 | *ZNF644, HFM1, CDC7, TGFBR3, BRDT, EPHX4, KIAA1107, GLMN, RPAP2, GFI1, EVI5L, RPL5, DIPK1A, MTF2, TMED5, CCDC18, RDH8, DR1, Fnbp1l, BCAR3, DNTTIP2, GCLM, TECR, ABCA4, arhgap29, Abcd3, F3* |
| 8 | 415235 | 1606167 | 9.8948 | *ZNF644, HFM1, CDC7, TGFBR3, BRDT, EPHX4, KIAA1107, GLMN, RPAP2, GFI1, EVI5L, RPL5, DIPK1A, MTF2, TMED5, CCDC18, RDH8, DR1, Fnbp1l, BCAR3, DNTTIP2, GCLM, TECR, ABCA4, arhgap29, Abcd3, F3* |
| 8 | 415235 | 1606516 | 11.8642 | *ZNF644, HFM1, CDC7, TGFBR3, BRDT, EPHX4, KIAA1107, GLMN, RPAP2, GFI1, EVI5L, RPL5, DIPK1A, MTF2, TMED5, CCDC18, RDH8, DR1, Fnbp1l, BCAR3, DNTTIP2, GCLM, TECR, ABCA4, arhgap29, Abcd3, F3* |
| 8 | 420281 | 1597696 | 12.2234 | *ZNF644, HFM1, CDC7, TGFBR3, BRDT, EPHX4, KIAA1107, GLMN, RPAP2, GFI1, EVI5L, RPL5, DIPK1A, MTF2, TMED5, CCDC18, RDH8, DR1, Fnbp1l, BCAR3, DNTTIP2, GCLM, TECR, ABCA4, arhgap29, Abcd3, F3* |
| 8 | 12300159 | 12326002 | 8.8459 | *COP1* |
| 8 | 21196579 | 21310690 | 9.7925 | *SZT2, Hyi, PTPRF* |
| 9 | 4081793 | 4491569 | 11.6267 | *PDCD10, chr30.95, SERPINI1, GOLIM4, MEGF6, MECOM* |
| 9 | 4084546 | 4486468 | 11.9589 | *PDCD10, chr30.95, SERPINI1, GOLIM4, MEGF6, MECOM* |
| 9 | 4084925 | 4490295 | 10.6055 | *PDCD10, chr30.95, SERPINI1, GOLIM4, MEGF6, MECOM* |
| 9 | 4090132 | 4479162 | 12.0212 | *PDCD10, chr30.95, SERPINI1, GOLIM4, MEGF6* |
| 9 | 4102342 | 4463840 | 11.1578 | *SERPINI1, GOLIM4, MEGF6* |
| 9 | 4119782 | 4441250 | 13.2630 | *SERPINI1, GOLIM4, MEGF6* |
| 9 | 4125357 | 4433854 | 12.7871 | *SERPINI1, GOLIM4, MEGF6* |
| 9 | 4136038 | 4420789 | 11.5351 | *SERPINI1, GOLIM4, MEGF6* |
| 9 | 4157179 | 4398226 | 10.7116 | *GOLIM4, MEGF6* |
| 9 | 4190676 | 4332666 | 11.4036 | *GOLIM4* |
| 9 | 4208454 | 4324810 | 7.6382 | *GOLIM4* |
| 9 | 4211810 | 4324810 | 9.1242 | *GOLIM4* |
| 10 | 3324414 | 3947255 | 21.0992 | *CNTN3, PDZRN3* |
| 10 | 3327758 | 3941808 | 8.6237 | *CNTN3, PDZRN3* |
| 10 | 3345571 | 3927970 | 20.7027 | *CNTN3, PDZRN3* |
| 10 | 3485836 | 3753887 | 15.2084 | *CNTN3* |
| 10 | 3494098 | 3842774 | 18.3663 | *CNTN3* |
| 10 | 3498326 | 3693447 | 13.7780 | *CNTN3* |
| 10 | 3514823 | 3628658 | 8.2707 | *CNTN3* |
| 10 | 3522487 | 3816751 | 15.4108 | *CNTN3* |
| 10 | 3529702 | 3707635 | 9.3671 | *CNTN3* |
| 10 | 3540217 | 3817434 | 13.2946 | *CNTN3* |
| 10 | 3554488 | 3661113 | 10.2322 | *CNTN3* |
| 10 | 3567898 | 3662898 | 8.7260 | *CNTN3* |
| 17 | 5949047 | 6369119 | 14.7546 | *PI4KA, HIC2, Ube2l3, ydjc, SDF2L1, Top3b, PPM1F, Mapk1* |
| 17 | 5951708 | 6364491 | 8.0117 | *PI4KA, HIC2, Ube2l3, ydjc, SDF2L1, Top3b, PPM1F* |
| 17 | 5964109 | 6356063 | 14.6264 | *HIC2, Ube2l3, ydjc, SDF2L1, Top3b, PPM1F* |
| 17 | 6088511 | 6253689 | 8.4954 | *Ube2l3, ydjc, SDF2L1* |
| 18 | 967258 | 1004197 | 7.9759 | *DNAH9* |
| 18 | 7080262 | 7568611 | 9.1918 | *KIF2A* |
| 18 | 7096652 | 7549979 | 7.8294 | *KIF2A* |
| 18 | 7156783 | 7472510 | 10.1349 | *KIF2A* |
| 18 | 7216355 | 7402129 | 8.8469 | *KIF2A* |
| 18 | 7221878 | 7489346 | 15.8391 | *KIF2A* |
| 18 | 7300037 | 7405914 | 8.5543 | *KIF2A* |
| 18 | 8879760 | 9169187 | 8.0852 | *TEX2, PECAM1, MILR1, POLG2, Ddx5, CEP95, SMURF2, KPNA2, C17orf58, BPTF* |
| 18 | 8881419 | 9169187 | 8.6524 | *TEX2, PECAM1, MILR1, POLG2, Ddx5, CEP95, SMURF2, KPNA2, C17orf58, BPTF* |
| 18 | 8903044 | 9153919 | 10.0306 | *PECAM1, MILR1, POLG2, Ddx5, CEP95, SMURF2, KPNA2, C17orf58, BPTF* |
| 18 | 8927750 | 9148686 | 14.5492 | *PECAM1, MILR1, POLG2, Ddx5, CEP95, SMURF2, KPNA2, C17orf58, BPTF* |
| 18 | 8952142 | 9121017 | 11.1787 | *PECAM1, MILR1, POLG2, Ddx5, CEP95, SMURF2, KPNA2, C17orf58, BPTF* |
| 22 | 836193 | 1009958 | 10.6949 | *chr16.27, Ctps1, SCMH1* |
| 22 | 836878 | 1009958 | 14.4511 | *chr16.27, Ctps1, SCMH1* |
| 22 | 837455 | 1022968 | 9.2372 | *chr16.27, Ctps1, SCMH1* |
| 22 | 849650 | 994594 | 10.7168 | *chr16.27, Ctps1, SCMH1* |
| 22 | 864655 | 972026 | 8.1958 | *chr16.27* |
| 22 | 879804 | 984373 | 12.2208 | *chr16.27, Ctps1* |
| 22 | 883612 | 972026 | 11.9210 | *chr16.27* |
| 22 | 893503 | 972026 | 9.4503 | *chr16.27* |
| 30 | 946367 | 1099554 | 7.6853 | *MVP, ACADM* |
| 30 | 958310 | 1077499 | 9.0291 | *MVP* |
| 30 | 974322 | 1060760 | 8.9705 | *MVP* |

Note: The table lists the genomic regions with the top 0.1% CLR values, which are suggestive of positive selection in the Changle goose. CLR Value indicates the strength of the selection signal, and the Associated Gene(s) column identifies the genes found within these regions. Chromosome positions are given in base pairs (bp).

**Table S5.** **Genomic regions with significant frequency difference in the Changle goose (CLG) compared to other breeds.**

| Chromosome | Start Position (bp) | End Position (bp) | Frequency Difference | Number of SNPs | Associated Gene(s) |
| --- | --- | --- | --- | --- | --- |
| 1 | 3820001 | 3830000 | 0.1938 | 94 | *Ints4* |
| 1 | 8850001 | 8860000 | 0.1871 | 112 | *GRM5* |
| 1 | 18430001 | 18440000 | 0.2422 | 61 | *chr38.347, PSPC1* |
| 1 | 26140001 | 26150000 | 0.1914 | 60 | *TRPC4* |
| 1 | 29960001 | 29970000 | 0.1885 | 92 | *Lrch1* |
| 1 | 43170001 | 43180000 | 0.1934 | 36 | *Dis3* |
| 1 | 56420001 | 56430000 | 0.1998 | 99 | *UBAC2* |
| 1 | 68290001 | 68300000 | 0.1894 | 107 | *Pdcl3* |
| 1 | 68300001 | 68310000 | 0.2637 | 45 | *Mkln1* |
| 1 | 68340001 | 68350000 | 0.1876 | 94 | *CHST10* |
| 1 | 79210001 | 79220000 | 0.1984 | 83 | *TCEANC* |
| 1 | 80760001 | 80770000 | 0.1915 | 62 | *CTPS2* |
| 1 | 92350001 | 92360000 | 0.2389 | 92 | *AGPAT3* |
| 1 | 92360001 | 92370000 | 0.3057 | 91 | *AGPAT3* |
| 1 | 92370001 | 92380000 | 0.1940 | 102 | *AGPAT3* |
| 1 | 92440001 | 92450000 | 0.1862 | 122 | *AGPAT3* |
| 1 | 92440001 | 92450000 | 0.1862 | 122 | *chr38.1225* |
| 1 | 92450001 | 92460000 | 0.2003 | 199 | *chr38.1225* |
| 1 | 92470001 | 92480000 | 0.2161 | 122 | *chr38.1225* |
| 1 | 92490001 | 92500000 | 0.2024 | 149 | *Pdxk* |
| 1 | 92500001 | 92510000 | 0.2753 | 130 | *Pdxk* |
| 1 | 92510001 | 92520000 | 0.2147 | 108 | *Pdxk* |
| 1 | 92520001 | 92530000 | 0.2354 | 87 | *Pdxk* |
| 1 | 92530001 | 92540000 | 0.1955 | 122 | *Pdxk* |
| 1 | 95840001 | 95850000 | 0.1915 | 108 | *HLCS* |
| 1 | 99990001 | 100000000 | 0.2129 | 117 | *Cyyr1* |
| 1 | 100010001 | 100020000 | 0.1995 | 147 | *app* |
| 1 | 100020001 | 100030000 | 0.2230 | 93 | *app* |
| 1 | 100030001 | 100040000 | 0.1919 | 120 | *app* |
| 1 | 104560001 | 104570000 | 0.1903 | 85 | *ELOVL4* |
| 1 | 116920001 | 116930000 | 0.1886 | 50 | *ALCAM* |
| 1 | 116930001 | 116940000 | 0.2053 | 62 | *ALCAM* |
| 1 | 116960001 | 116970000 | 0.2107 | 55 | *ALCAM* |
| 1 | 117080001 | 117090000 | 0.1954 | 56 | *CBLB* |
| 1 | 117670001 | 117680000 | 0.2107 | 85 | *BBX* |
| 1 | 117680001 | 117690000 | 0.2189 | 52 | *BBX* |
| 1 | 117700001 | 117710000 | 0.2647 | 58 | *BBX* |
| 1 | 117750001 | 117760000 | 0.1860 | 92 | *BBX* |
| 1 | 117940001 | 117950000 | 0.2274 | 61 | *RCJMB04_1n3* |
| 1 | 122350001 | 122360000 | 0.1888 | 53 | *ILDR2* |
| 1 | 122530001 | 122540000 | 0.2698 | 108 | *PTGFRN* |
| 1 | 126950001 | 126960000 | 0.2501 | 25 | *Robo1* |
| 1 | 126960001 | 126970000 | 0.2227 | 44 | *Robo1* |
| 1 | 126970001 | 126980000 | 0.2401 | 23 | *Robo1* |
| 1 | 126980001 | 126990000 | 0.2894 | 33 | *Robo1* |
| 1 | 128730001 | 128740000 | 0.1881 | 15 | *LIPH* |
| 1 | 136090001 | 136100000 | 0.1956 | 46 | *KIAA0930* |
| 1 | 136090001 | 136100000 | 0.1956 | 46 | *Adck2* |
| 1 | 142910001 | 142920000 | 0.1964 | 86 | *DYRK4* |
| 1 | 145470001 | 145480000 | 0.1884 | 46 | *CAPRIN2* |
| 1 | 145550001 | 145560000 | 0.1992 | 131 | *chr38.2089* |
| 1 | 145550001 | 145560000 | 0.1992 | 131 | *chr38.2090* |
| 1 | 145570001 | 145580000 | 0.1880 | 126 | *DENND5B* |
| 1 | 145590001 | 145600000 | 0.2022 | 155 | *DENND5B* |
| 1 | 148530001 | 148540000 | 0.2333 | 82 | *KDM7A* |
| 1 | 152440001 | 152450000 | 0.1910 | 122 | *chr38.2223* |
| 1 | 155570001 | 155580000 | 0.1880 | 138 | *ST13* |
| 1 | 157500001 | 157510000 | 0.2163 | 84 | *SLC6A15* |
| 1 | 157620001 | 157630000 | 0.1943 | 55 | *LRRIQ1* |
| 1 | 157640001 | 157650000 | 0.2140 | 47 | *LRRIQ1* |
| 1 | 161160001 | 161170000 | 0.1919 | 104 | *MRPL42* |
| 1 | 163460001 | 163470000 | 0.1939 | 88 | *Anks1b* |
| 1 | 165960001 | 165970000 | 0.2112 | 101 | *3-Sep* |
| 1 | 169860001 | 169870000 | 0.2041 | 79 | *TRHDE* |
| 1 | 171230001 | 171240000 | 0.1942 | 109 | *FRS2* |
| 1 | 173160001 | 173170000 | 0.1995 | 90 | *Tbk1* |
| 1 | 176250001 | 176260000 | 0.2547 | 63 | *SCAF11* |
| 1 | 177650001 | 177660000 | 0.1863 | 131 | *ZCRB1* |
| 1 | 177730001 | 177740000 | 0.1910 | 152 | *GXYLT1* |
| 1 | 178700001 | 178710000 | 0.2083 | 131 | *PNPLA8* |
| 1 | 178710001 | 178720000 | 0.2384 | 86 | *PNPLA8* |
| 1 | 178720001 | 178730000 | 0.2434 | 105 | *PNPLA8* |
| 1 | 178720001 | 178730000 | 0.2069 | 105 | *ARSD* |
| 1 | 178730001 | 178740000 | 0.1947 | 121 | *ARSD* |
| 1 | 178750001 | 178760000 | 0.2232 | 157 | *ARSD* |
| 1 | 179610001 | 179620000 | 0.2074 | 91 | *IMMP2L* |
| 1 | 179620001 | 179630000 | 0.1864 | 83 | *IMMP2L* |
| 1 | 179660001 | 179670000 | 0.2232 | 63 | *IMMP2L* |
| 1 | 179680001 | 179690000 | 0.1930 | 78 | *IMMP2L* |
| 1 | 179710001 | 179720000 | 0.2158 | 95 | *IMMP2L* |
| 1 | 179730001 | 179740000 | 0.2068 | 90 | *IMMP2L* |
| 1 | 179810001 | 179820000 | 0.1952 | 83 | *IMMP2L* |
| 1 | 179820001 | 179830000 | 0.2366 | 103 | *IMMP2L* |
| 1 | 179830001 | 179840000 | 0.2488 | 92 | *IMMP2L* |
| 1 | 179840001 | 179850000 | 0.2488 | 95 | *IMMP2L* |
| 1 | 179850001 | 179860000 | 0.2453 | 85 | *IMMP2L* |
| 1 | 179860001 | 179870000 | 0.2205 | 122 | *IMMP2L* |
| 1 | 179870001 | 179880000 | 0.2262 | 133 | *IMMP2L* |
| 1 | 180360001 | 180370000 | 0.1899 | 140 | *ZNF277* |
| 1 | 180540001 | 180550000 | 0.1862 | 107 | *TMEM168* |
| 1 | 180570001 | 180580000 | 0.1860 | 160 | *BMT2* |
| 1 | 180700001 | 180710000 | 0.1946 | 154 | *chr38.2723* |
| 1 | 182590001 | 182600000 | 0.1989 | 144 | *CAPZA2* |
| 1 | 183700001 | 183710000 | 0.1878 | 45 | *DDB_G0269086* |
| 1 | 184440001 | 184450000 | 0.1996 | 68 | *ING3* |
| 1 | 184660001 | 184670000 | 0.1889 | 99 | *FAM3C* |
| 1 | 185390001 | 185400000 | 0.2367 | 103 | *Cadps2* |
| 1 | 186890001 | 186900000 | 0.1893 | 96 | *GRM8* |
| 1 | 186950001 | 186960000 | 0.1915 | 72 | *GRM8* |
| 1 | 186960001 | 186970000 | 0.2138 | 96 | *GRM8* |
| 1 | 187010001 | 187020000 | 0.2078 | 80 | *GRM8* |
| 1 | 187030001 | 187040000 | 0.2070 | 68 | *GRM8* |
| 1 | 187050001 | 187060000 | 0.2032 | 87 | *GRM8* |
| 1 | 187070001 | 187080000 | 0.2161 | 95 | *GRM8* |
| 1 | 187100001 | 187110000 | 0.2093 | 93 | *GRM8* |
| 1 | 204870001 | 204880000 | 0.2051 | 151 | *TAF3* |
| 1 | 204910001 | 204920000 | 0.2032 | 163 | *TAF3* |
| 1 | 204930001 | 204940000 | 0.2397 | 153 | *TAF3* |
| 1 | 204940001 | 204950000 | 0.2199 | 115 | *TAF3* |
| 1 | 206950001 | 206960000 | 0.2012 | 159 | *Chchd3* |
| 1 | 206960001 | 206970000 | 0.2627 | 145 | *Chchd3* |
| 2 | 9230001 | 9240000 | 0.1867 | 111 | *ADCY8* |
| 2 | 9240001 | 9250000 | 0.2033 | 76 | *ADCY8* |
| 2 | 11430001 | 11440000 | 0.2248 | 110 | *Nsmce2* |
| 2 | 11440001 | 11450000 | 0.2025 | 108 | *Nsmce2* |
| 2 | 11450001 | 11460000 | 0.1872 | 125 | *Nsmce2* |
| 2 | 11460001 | 11470000 | 0.2082 | 104 | *Nsmce2* |
| 2 | 11470001 | 11480000 | 0.2026 | 80 | *Nsmce2* |
| 2 | 16450001 | 16460000 | 0.2068 | 20 | *Csmd3* |
| 2 | 16580001 | 16590000 | 0.1863 | 21 | *Csmd3* |
| 2 | 16600001 | 16610000 | 0.2017 | 37 | *Csmd3* |
| 2 | 16610001 | 16620000 | 0.1913 | 38 | *Csmd3* |
| 2 | 23910001 | 23920000 | 0.1888 | 104 | *PTDSS1* |
| 2 | 29870001 | 29880000 | 0.2178 | 144 | *FABP4* |
| 2 | 29890001 | 29900000 | 0.1897 | 150 | *FABP4* |
| 2 | 31060001 | 31070000 | 0.1992 | 106 | *PKIA* |
| 2 | 31830001 | 31840000 | 0.2061 | 55 | *Pex2* |
| 2 | 31940001 | 31950000 | 0.1956 | 35 | *ZFHX4* |
| 2 | 36910001 | 36920000 | 0.2013 | 110 | *ARMC1* |
| 2 | 59170001 | 59180000 | 0.1957 | 72 | *CCDC102B* |
| 2 | 59480001 | 59490000 | 0.2154 | 90 | *DOK6* |
| 2 | 61630001 | 61640000 | 0.1875 | 76 | *C18orf63* |
| 2 | 61630001 | 61640000 | 0.1875 | 76 | *C18orf63* |
| 2 | 61640001 | 61650000 | 0.1991 | 106 | *C18orf63* |
| 2 | 61690001 | 61700000 | 0.2079 | 178 | *CNDP2* |
| 2 | 61690001 | 61700000 | 0.2079 | 178 | *chr37.774* |
| 2 | 61730001 | 61740000 | 0.2237 | 131 | *Cndp1* |
| 2 | 63810001 | 63820000 | 0.2031 | 83 | *Pard6g* |
| 2 | 63830001 | 63840000 | 0.1893 | 91 | *Pard6g* |
| 2 | 63830001 | 63840000 | 0.1893 | 91 | *Pard6g* |
| 2 | 63840001 | 63850000 | 0.2132 | 136 | *Bloc1s4* |
| 2 | 64370001 | 64380000 | 0.1945 | 93 | *ERP44* |
| 2 | 64640001 | 64650000 | 0.1984 | 108 | *AHRR* |
| 2 | 65490001 | 65500000 | 0.1885 | 68 | *GABBR2* |
| 2 | 65510001 | 65520000 | 0.1893 | 92 | *GABBR2* |
| 2 | 65550001 | 65560000 | 0.2365 | 54 | *GABBR2* |
| 2 | 65560001 | 65570000 | 0.2034 | 82 | *GABBR2* |
| 2 | 65570001 | 65580000 | 0.1868 | 78 | *GABBR2* |
| 2 | 65610001 | 65620000 | 0.2161 | 103 | *GABBR2* |
| 2 | 65690001 | 65700000 | 0.2045 | 79 | *GABBR2* |
| 2 | 74900001 | 74910000 | 0.1998 | 110 | *TENT4A* |
| 2 | 76920001 | 76930000 | 0.1926 | 96 | *CTNND2* |
| 2 | 80460001 | 80470000 | 0.2156 | 85 | *SERPINB10* |
| 2 | 102990001 | 103000000 | 0.2009 | 50 | *TPK1* |
| 2 | 112090001 | 112100000 | 0.1981 | 54 | *Sall3* |
| 2 | 112720001 | 112730000 | 0.1897 | 110 | *KCNG2* |
| 2 | 115820001 | 115830000 | 0.2005 | 117 | *Col6a4* |
| 2 | 116940001 | 116950000 | 0.1911 | 92 | *CNOT10* |
| 2 | 117000001 | 117010000 | 0.2021 | 118 | *DYNC1LI1* |
| 2 | 117940001 | 117950000 | 0.2124 | 88 | *RBMS3* |
| 2 | 129470001 | 129480000 | 0.1891 | 103 | *BZW2* |
| 2 | 129970001 | 129980000 | 0.2150 | 37 | *MEOX2* |
| 2 | 134870001 | 134880000 | 0.1939 | 89 | *CASD1* |
| 2 | 138330001 | 138340000 | 0.2217 | 146 | *FAM171A1* |
| 2 | 139280001 | 139290000 | 0.2239 | 120 | *STAM* |
| 2 | 139280001 | 139290000 | 0.2239 | 120 | *Stam* |
| 2 | 139420001 | 139430000 | 0.1906 | 85 | *MRC1* |
| 2 | 139420001 | 139430000 | 0.1906 | 85 | *MRC1* |
| 2 | 139570001 | 139580000 | 0.1868 | 96 | *SLC39A12* |
| 2 | 139580001 | 139590000 | 0.1942 | 61 | *SLC39A12* |
| 2 | 139700001 | 139710000 | 0.2193 | 71 | *Cacnb2* |
| 2 | 140550001 | 140560000 | 0.2028 | 55 | *Plxdc2* |
| 2 | 143140001 | 143150000 | 0.2205 | 108 | *APBB1IP* |
| 2 | 143190001 | 143200000 | 0.2013 | 49 | *APBB1IP* |
| 2 | 143200001 | 143210000 | 0.2460 | 67 | *APBB1IP* |
| 2 | 143210001 | 143220000 | 0.2026 | 83 | *APBB1IP* |
| 2 | 143210001 | 143220000 | 0.2026 | 83 | *selO* |
| 2 | 143230001 | 143240000 | 0.2401 | 98 | *selO* |
| 2 | 152390001 | 152400000 | 0.1921 | 106 | *PAXIP1* |
| 3 | 940001 | 950000 | 0.1900 | 120 | *RAB10* |
| 3 | 4120001 | 4130000 | 0.1875 | 86 | *PKHD1* |
| 3 | 5660001 | 5670000 | 0.1863 | 135 | *SUPT3H* |
| 3 | 6540001 | 6550000 | 0.1878 | 179 | *CD2AP* |
| 3 | 6550001 | 6560000 | 0.1860 | 120 | *CD2AP* |
| 3 | 8140001 | 8150000 | 0.2387 | 119 | *KLHL29* |
| 3 | 15560001 | 15570000 | 0.2054 | 128 | *KCNF1* |
| 3 | 15650001 | 15660000 | 0.2136 | 74 | *PDIA6* |
| 3 | 15650001 | 15660000 | 0.2136 | 74 | *Atp6v1c2* |
| 3 | 15670001 | 15680000 | 0.2032 | 129 | *Atp6v1c2* |
| 3 | 19920001 | 19930000 | 0.1996 | 76 | *Pxdn* |
| 3 | 22640001 | 22650000 | 0.1922 | 48 | *SPARC* |
| 3 | 28870001 | 28880000 | 0.1878 | 100 | *eys* |
| 3 | 28890001 | 28900000 | 0.2424 | 70 | *eys* |
| 3 | 28980001 | 28990000 | 0.1902 | 94 | *eys* |
| 3 | 31530001 | 31540000 | 0.2048 | 73 | *SDHAF4* |
| 3 | 34980001 | 34990000 | 0.2129 | 58 | *Phip* |
| 3 | 37890001 | 37900000 | 0.2043 | 50 | *SYNCRIP* |
| 3 | 45130001 | 45140000 | 0.2308 | 89 | *Grik2* |
| 3 | 58320001 | 58330000 | 0.2097 | 88 | *LAMA2* |
| 3 | 64320001 | 64330000 | 0.1952 | 125 | *AIG1* |
| 3 | 65480001 | 65490000 | 0.1880 | 142 | *TMEM242* |
| 3 | 65720001 | 65730000 | 0.2029 | 75 | *Arid1b* |
| 3 | 66730001 | 66740000 | 0.2030 | 77 | *SCAF8* |
| 3 | 67540001 | 67550000 | 0.2116 | 62 | *MTRF1L* |
| 3 | 67540001 | 67550000 | 0.2116 | 62 | *FBXO5* |
| 3 | 67680001 | 67690000 | 0.2279 | 58 | *VIP* |
| 3 | 67720001 | 67730000 | 0.2170 | 93 | *myct1* |
| 3 | 67730001 | 67740000 | 0.2513 | 85 | *myct1* |
| 3 | 67760001 | 67770000 | 0.2127 | 42 | *SYNE1* |
| 3 | 67770001 | 67780000 | 0.2478 | 60 | *SYNE1* |
| 3 | 67780001 | 67790000 | 0.2053 | 51 | *SYNE1* |
| 3 | 67790001 | 67800000 | 0.1890 | 43 | *SYNE1* |
| 3 | 67810001 | 67820000 | 0.2107 | 45 | *SYNE1* |
| 3 | 67830001 | 67840000 | 0.3270 | 39 | *SYNE1* |
| 3 | 67840001 | 67850000 | 0.3489 | 69 | *SYNE1* |
| 3 | 67840001 | 67850000 | 0.3489 | 69 | *SYNE1* |
| 3 | 67850001 | 67860000 | 0.3660 | 78 | *SYNE1* |
| 3 | 67860001 | 67870000 | 0.3303 | 43 | *SYNE1* |
| 3 | 67870001 | 67880000 | 0.3547 | 55 | *SYNE1* |
| 3 | 67870001 | 67880000 | 0.3547 | 55 | *SYNE1* |
| 3 | 67880001 | 67890000 | 0.2932 | 66 | *SYNE1* |
| 3 | 67880001 | 67890000 | 0.2932 | 66 | *SYNE1* |
| 3 | 67890001 | 67900000 | 0.2958 | 77 | *SYNE1* |
| 3 | 67950001 | 67960000 | 0.2055 | 41 | *SYNE1* |
| 3 | 67990001 | 68000000 | 0.2137 | 38 | *SYNE1* |
| 3 | 68000001 | 68010000 | 0.2146 | 28 | *SYNE1* |
| 3 | 68010001 | 68020000 | 0.2299 | 43 | *SYNE1* |
| 3 | 68020001 | 68030000 | 0.2883 | 76 | *SYNE1* |
| 3 | 68030001 | 68040000 | 0.2728 | 39 | *SYNE1* |
| 3 | 68040001 | 68050000 | 0.2001 | 49 | *SYNE1* |
| 3 | 68070001 | 68080000 | 0.2419 | 61 | *ESR1* |
| 3 | 68080001 | 68090000 | 0.1897 | 59 | *ESR1* |
| 3 | 68150001 | 68160000 | 0.2952 | 47 | *ESR1* |
| 3 | 68160001 | 68170000 | 0.3140 | 62 | *ESR1* |
| 3 | 68170001 | 68180000 | 0.2776 | 58 | *ESR1* |
| 3 | 68180001 | 68190000 | 0.2963 | 33 | *ESR1* |
| 3 | 68190001 | 68200000 | 0.3117 | 41 | *ESR1* |
| 3 | 68270001 | 68280000 | 0.2331 | 123 | *CCDC170* |
| 3 | 68280001 | 68290000 | 0.2002 | 97 | *CCDC170* |
| 3 | 77130001 | 77140000 | 0.1979 | 133 | *FAM120B* |
| 3 | 77350001 | 77360000 | 0.2003 | 28 | *ARV1* |
| 3 | 86180001 | 86190000 | 0.2019 | 89 | *CRIM1* |
| 3 | 86190001 | 86200000 | 0.2226 | 109 | *CRIM1* |
| 3 | 86200001 | 86210000 | 0.1939 | 51 | *CRIM1* |
| 3 | 86240001 | 86250000 | 0.1922 | 29 | *CRIM1* |
| 3 | 86300001 | 86310000 | 0.1913 | 80 | *CRIM1* |
| 3 | 86330001 | 86340000 | 0.1985 | 115 | *CRIM1* |
| 3 | 86370001 | 86380000 | 0.2462 | 65 | *Fez2* |
| 3 | 86410001 | 86420000 | 0.1973 | 101 | *OPN5* |
| 3 | 86430001 | 86440000 | 0.1952 | 116 | *OPN5* |
| 3 | 86530001 | 86540000 | 0.2031 | 75 | *STRN* |
| 3 | 86580001 | 86590000 | 0.1900 | 86 | *STRN* |
| 3 | 86580001 | 86590000 | 0.1900 | 86 | *HEATR5B* |
| 3 | 91240001 | 91250000 | 0.1874 | 113 | *chr36.1257* |
| 3 | 92360001 | 92370000 | 0.1913 | 98 | *SRSF7* |
| 3 | 94020001 | 94030000 | 0.2089 | 96 | *PLEKHH2* |
| 3 | 94160001 | 94170000 | 0.1953 | 96 | *THADA* |
| 3 | 97960001 | 97970000 | 0.1903 | 134 | *Cnih4* |
| 3 | 97960001 | 97970000 | 0.1903 | 134 | *NVL* |
| 3 | 102530001 | 102540000 | 0.1868 | 144 | *VASH2* |
| 3 | 102540001 | 102550000 | 0.2046 | 178 | *FLVCR1* |
| 3 | 102720001 | 102730000 | 0.2157 | 105 | *Tmem63a* |
| 3 | 105770001 | 105780000 | 0.2212 | 105 | *NRXN1* |
| 3 | 107690001 | 107700000 | 0.2049 | 99 | *Acss1* |
| 3 | 107950001 | 107960000 | 0.2100 | 50 | *Entpd6* |
| 3 | 112160001 | 112170000 | 0.2527 | 33 | *GZF1* |
| 3 | 112180001 | 112190000 | 0.1880 | 50 | *GZF1* |
| 3 | 112180001 | 112190000 | 0.1880 | 50 | *Napb* |
| 3 | 113960001 | 113970000 | 0.1973 | 37 | *VRK2* |
| 3 | 118140001 | 118150000 | 0.1904 | 97 | *SPTLC3* |
| 4 | 1370001 | 1380000 | 0.1977 | 118 | *Exoc6b* |
| 4 | 8890001 | 8900000 | 0.2076 | 118 | *POLN* |
| 4 | 8980001 | 8990000 | 0.2008 | 202 | *HAUS3* |
| 4 | 9250001 | 9260000 | 0.1947 | 139 | *CFAP99* |
| 4 | 9300001 | 9310000 | 0.1981 | 102 | *CFAP99* |
| 4 | 9300001 | 9310000 | 0.1981 | 102 | *Rnf4* |
| 4 | 9880001 | 9890000 | 0.1925 | 113 | *RGS12* |
| 4 | 13130001 | 13140000 | 0.2049 | 78 | *STK32B* |
| 4 | 13230001 | 13240000 | 0.1886 | 100 | *STK32B* |
| 4 | 13250001 | 13260000 | 0.2269 | 91 | *STK32B* |
| 4 | 25250001 | 25260000 | 0.2065 | 179 | *slc30a9* |
| 4 | 31740001 | 31750000 | 0.2337 | 46 | *PRKG2* |
| 4 | 31750001 | 31760000 | 0.2305 | 53 | *PRKG2* |
| 4 | 32600001 | 32610000 | 0.1921 | 115 | *LIN54* |
| 4 | 36370001 | 36380000 | 0.1892 | 82 | *Pgrmc2* |
| 4 | 36370001 | 36380000 | 0.1892 | 82 | *chr35.602* |
| 4 | 40490001 | 40500000 | 0.1982 | 77 | *MGARP* |
| 4 | 45440001 | 45450000 | 0.2050 | 58 | *ARFIP1* |
| 4 | 45450001 | 45460000 | 0.1916 | 92 | *ARFIP1* |
| 4 | 60570001 | 60580000 | 0.3116 | 57 | *TUSC3* |
| 4 | 60580001 | 60590000 | 0.2958 | 55 | *TUSC3* |
| 4 | 61110001 | 61120000 | 0.2610 | 42 | *SGCZ* |
| 4 | 61130001 | 61140000 | 0.2352 | 60 | *SGCZ* |
| 4 | 61150001 | 61160000 | 0.2094 | 28 | *SGCZ* |
| 4 | 61850001 | 61860000 | 0.1916 | 80 | *MRPL1* |
| 4 | 63650001 | 63660000 | 0.2020 | 57 | *Mroh2b* |
| 4 | 67140001 | 67150000 | 0.1975 | 84 | *CCSER1* |
| 4 | 67250001 | 67260000 | 0.1973 | 100 | *CCSER1* |
| 4 | 77240001 | 77250000 | 0.1993 | 87 | *PRSS12* |
| 5 | 10840001 | 10850000 | 0.1865 | 151 | *EXOC3L2* |
| 5 | 16030001 | 16040000 | 0.1983 | 111 | *Asb2* |
| 5 | 16960001 | 16970000 | 0.2038 | 61 | *TRIP11* |
| 5 | 21530001 | 21540000 | 0.2348 | 68 | *CEP128* |
| 5 | 21910001 | 21920000 | 0.2059 | 68 | *TMEM251* |
| 5 | 27270001 | 27280000 | 0.2030 | 100 | *MUC5B* |
| 5 | 30910001 | 30920000 | 0.2056 | 80 | *TMEM80* |
| 5 | 30910001 | 30920000 | 0.2056 | 80 | *Deaf1* |
| 5 | 30920001 | 30930000 | 0.2117 | 67 | *Deaf1* |
| 5 | 31110001 | 31120000 | 0.1968 | 59 | *PLCB2* |
| 5 | 31140001 | 31150000 | 0.1992 | 96 | *PLCB2* |
| 5 | 31410001 | 31420000 | 0.2019 | 104 | *PCMT1* |
| 5 | 31410001 | 31420000 | 0.2019 | 104 | *Rpusd2* |
| 5 | 31410001 | 31420000 | 0.2019 | 104 | *KNL1* |
| 5 | 31420001 | 31430000 | 0.2378 | 94 | *KNL1* |
| 5 | 31430001 | 31440000 | 0.2755 | 114 | *KNL1* |
| 5 | 31440001 | 31450000 | 0.2602 | 123 | *KNL1* |
| 5 | 31450001 | 31460000 | 0.2522 | 105 | *RAD51A* |
| 5 | 31460001 | 31470000 | 0.2750 | 156 | *RAD51A* |
| 5 | 31470001 | 31480000 | 0.2032 | 112 | *CYP1B1* |
| 5 | 31490001 | 31500000 | 0.2489 | 125 | *RMDN3* |
| 5 | 31510001 | 31520000 | 0.2268 | 127 | *RMDN3* |
| 5 | 34610001 | 34620000 | 0.1959 | 72 | *CAPN3* |
| 5 | 34790001 | 34800000 | 0.2450 | 65 | *STARD9* |
| 5 | 34790001 | 34800000 | 0.2450 | 65 | *STARD9* |
| 5 | 34970001 | 34980000 | 0.1870 | 36 | *UBR1* |
| 5 | 35200001 | 35210000 | 0.1996 | 93 | *NUMB* |
| 5 | 35230001 | 35240000 | 0.1973 | 114 | *NUMB* |
| 5 | 35200001 | 35210000 | 0.2021 | 93 | *chr34.840* |
| 5 | 35320001 | 35330000 | 0.2021 | 48 | *PAPLN* |
| 5 | 36900001 | 36910000 | 0.1941 | 85 | *Smoc1* |
| 5 | 37580001 | 37590000 | 0.1889 | 70 | *RAD51B* |
| 5 | 37930001 | 37940000 | 0.2247 | 91 | *RAD51B* |
| 5 | 38090001 | 38100000 | 0.2564 | 105 | *arg2-a* |
| 5 | 44330001 | 44340000 | 0.2056 | 109 | *HECTD1* |
| 5 | 49800001 | 49810000 | 0.2334 | 118 | *LDLRAD3* |
| 5 | 52660001 | 52670000 | 0.2161 | 118 | *EXT2* |
| 5 | 52910001 | 52920000 | 0.2003 | 132 | *CD82* |
| 5 | 56460001 | 56470000 | 0.2005 | 109 | *LUZP2* |
| 5 | 56520001 | 56530000 | 0.1890 | 97 | *LUZP2* |
| 5 | 58680001 | 58690000 | 0.1922 | 94 | *ELP4* |
| 5 | 61630001 | 61640000 | 0.1968 | 67 | *SOX6* |
| 5 | 64160001 | 64170000 | 0.2224 | 38 | *SBF2* |
| 5 | 64180001 | 64190000 | 0.1953 | 37 | *SBF2* |
| 5 | 64850001 | 64860000 | 0.2211 | 36 | *GALNT18* |
| 5 | 64990001 | 65000000 | 0.2231 | 31 | *GALNT18* |
| 5 | 65090001 | 65100000 | 0.2132 | 19 | *USP47* |
| 5 | 65110001 | 65120000 | 0.2021 | 19 | *USP47* |
| 5 | 65120001 | 65130000 | 0.2060 | 17 | *USP47* |
| 5 | 65130001 | 65140000 | 0.2106 | 20 | *USP47* |
| 5 | 65140001 | 65150000 | 0.2002 | 22 | *DKK3* |
| 5 | 65150001 | 65160000 | 0.2594 | 38 | *DKK3* |
| 5 | 65160001 | 65170000 | 0.2169 | 21 | *DKK3* |
| 5 | 65220001 | 65230000 | 0.2966 | 53 | *Mical2* |
| 5 | 65230001 | 65240000 | 0.2329 | 42 | *Mical2* |
| 5 | 65240001 | 65250000 | 0.2525 | 28 | *Mical2* |
| 6 | 14250001 | 14260000 | 0.2386 | 99 | *GLI2* |
| 6 | 16710001 | 16720000 | 0.1866 | 163 | *TGFBR2* |
| 6 | 20140001 | 20150000 | 0.1966 | 85 | *Stk39* |
| 6 | 20160001 | 20170000 | 0.2071 | 87 | *Stk39* |
| 6 | 20230001 | 20240000 | 0.1860 | 107 | *Stk39* |
| 6 | 25980001 | 25990000 | 0.2158 | 75 | *IDH1* |
| 6 | 31330001 | 31340000 | 0.1919 | 82 | *COL6A3* |
| 6 | 31340001 | 31350000 | 0.2321 | 104 | *COL6A3* |
| 6 | 31810001 | 31820000 | 0.1896 | 111 | *FN1* |
| 7 | 4880001 | 4890000 | 0.2155 | 103 | *RET* |
| 7 | 8940001 | 8950000 | 0.1906 | 45 | *CCSER2* |
| 7 | 17430001 | 17440000 | 0.2133 | 103 | *GBF1* |
| 7 | 17440001 | 17450000 | 0.2005 | 87 | *GBF1* |
| 7 | 18790001 | 18800000 | 0.2001 | 79 | *FRMPD2* |
| 7 | 18810001 | 18820000 | 0.2085 | 164 | *FRMPD2* |
| 7 | 19000001 | 19010000 | 0.1971 | 112 | *ARHGAP22* |
| 7 | 23790001 | 23800000 | 0.1969 | 141 | *Dpcd* |
| 7 | 23790001 | 23800000 | 0.1969 | 141 | *POLL* |
| 7 | 25280001 | 25290000 | 0.2004 | 113 | *SLK* |
| 7 | 25280001 | 25290000 | 0.2004 | 113 | *COL17A1* |
| 7 | 30500001 | 30510000 | 0.1943 | 150 | *HSPA12A* |
| 7 | 32980001 | 32990000 | 0.1957 | 122 | *ATE1* |
| 8 | 3270001 | 3280000 | 0.2128 | 116 | *Mfsd14a* |
| 8 | 3290001 | 3300000 | 0.2360 | 84 | *SASS6* |
| 8 | 3290001 | 3300000 | 0.2360 | 84 | *TRMT13* |
| 8 | 3310001 | 3320000 | 0.1861 | 93 | *DBT* |
| 8 | 8990001 | 9000000 | 0.2167 | 146 | *Mta1* |
| 8 | 9000001 | 9010000 | 0.2205 | 118 | *Mta1* |
| 8 | 12680001 | 12690000 | 0.2113 | 96 | *Rabgap1* |
| 8 | 15660001 | 15670000 | 0.2083 | 91 | *Rnf2* |
| 8 | 15670001 | 15680000 | 0.2084 | 113 | *Rnf2* |
| 8 | 15910001 | 15920000 | 0.1916 | 147 | *C1orf21* |
| 8 | 16440001 | 16450000 | 0.1963 | 92 | *PKN2* |
| 8 | 25850001 | 25860000 | 0.1961 | 93 | *FAF1* |
| 8 | 25860001 | 25870000 | 0.1927 | 74 | *FAF1* |
| 8 | 25870001 | 25880000 | 0.2069 | 114 | *FAF1* |
| 8 | 25980001 | 25990000 | 0.2161 | 119 | *RNF11* |
| 8 | 26090001 | 26100000 | 0.2134 | 93 | *EPS15* |
| 8 | 26330001 | 26340000 | 0.2506 | 93 | *chr31.523* |
| 8 | 26340001 | 26350000 | 0.2337 | 62 | *chr31.523* |
| 8 | 26340001 | 26350000 | 0.2337 | 62 | *ZFYVE9* |
| 8 | 26340001 | 26350000 | 0.2337 | 62 | *ZFYVE9* |
| 8 | 26350001 | 26360000 | 0.2348 | 55 | *ZFYVE9* |
| 8 | 26360001 | 26370000 | 0.3512 | 81 | *ZFYVE9* |
| 8 | 26590001 | 26600000 | 0.1906 | 66 | *Echdc2* |
| 8 | 26590001 | 26600000 | 0.1906 | 66 | *SCP2* |
| 8 | 26600001 | 26610000 | 0.2052 | 89 | *SCP2* |
| 8 | 26630001 | 26640000 | 0.2099 | 84 | *PODN* |
| 8 | 26640001 | 26650000 | 0.2007 | 83 | *PODN* |
| 8 | 26650001 | 26660000 | 0.1939 | 122 | *SLC1A7* |
| 8 | 26660001 | 26670000 | 0.2206 | 108 | *SLC1A7* |
| 8 | 26670001 | 26680000 | 0.2161 | 104 | *SLC1A7* |
| 8 | 26680001 | 26690000 | 0.2128 | 108 | *SLC1A7* |
| 8 | 26690001 | 26700000 | 0.2356 | 88 | *SLC1A7* |
| 8 | 26730001 | 26740000 | 0.2108 | 156 | *NRDC* |
| 8 | 26820001 | 26830000 | 0.1906 | 72 | *LRP8* |
| 8 | 32500001 | 32510000 | 0.2132 | 41 | *TNNI3K* |
| 9 | 830001 | 840000 | 0.1974 | 78 | *MBNL1* |
| 9 | 5560001 | 5570000 | 0.2162 | 98 | *FNDC3B* |
| 9 | 5600001 | 5610000 | 0.1888 | 100 | *FNDC3B* |
| 9 | 5620001 | 5630000 | 0.2200 | 83 | *chr30.127* |
| 9 | 5620001 | 5630000 | 0.2200 | 83 | *TNFSF10* |
| 9 | 5650001 | 5660000 | 0.2251 | 79 | *NCEH1* |
| 9 | 5660001 | 5670000 | 0.2061 | 108 | *NCEH1* |
| 9 | 5740001 | 5750000 | 0.2354 | 126 | *Spata16* |
| 9 | 7560001 | 7570000 | 0.1893 | 96 | *PIK3CA* |
| 9 | 7570001 | 7580000 | 0.2140 | 74 | *PIK3CA* |
| 9 | 7570001 | 7580000 | 0.2140 | 74 | *PIK3CA* |
| 9 | 7690001 | 7700000 | 0.1917 | 125 | *Actl6a* |
| 9 | 7740001 | 7750000 | 0.1906 | 76 | *USP13* |
| 9 | 8060001 | 8070000 | 0.1905 | 101 | *Fxr1* |
| 9 | 8090001 | 8100000 | 0.1929 | 103 | *DNAJC19* |
| 9 | 8770001 | 8780000 | 0.2206 | 89 | *ATP11B* |
| 9 | 8780001 | 8790000 | 0.2035 | 139 | *ATP11B* |
| 9 | 8840001 | 8850000 | 0.2398 | 128 | *DCUN1D1* |
| 9 | 8920001 | 8930000 | 0.2005 | 80 | *MCF2L* |
| 9 | 8930001 | 8940000 | 0.1937 | 84 | *MCF2L* |
| 9 | 8940001 | 8950000 | 0.2070 | 78 | *MCF2L* |
| 9 | 8950001 | 8960000 | 0.2096 | 138 | *MCF2L* |
| 9 | 8990001 | 9000000 | 0.2024 | 51 | *MCF2L* |
| 9 | 10300001 | 10310000 | 0.1890 | 124 | *PSMD1* |
| 9 | 10320001 | 10330000 | 0.2105 | 85 | *PSMD1* |
| 9 | 10330001 | 10340000 | 0.1963 | 99 | *PSMD1* |
| 9 | 10360001 | 10370000 | 0.2574 | 90 | *PSMD1* |
| 9 | 10510001 | 10520000 | 0.2020 | 97 | *RUBCN* |
| 9 | 10540001 | 10550000 | 0.1902 | 164 | *Muc4* |
| 9 | 10550001 | 10560000 | 0.1922 | 150 | *Muc4* |
| 9 | 10580001 | 10590000 | 0.2131 | 172 | *TNK2* |
| 9 | 10590001 | 10600000 | 0.2008 | 89 | *TFRC* |
| 9 | 11090001 | 11100000 | 0.2078 | 98 | *LPP* |
| 9 | 17070001 | 17080000 | 0.2095 | 110 | *STAG1* |
| 9 | 19760001 | 19770000 | 0.1992 | 183 | *CLSTN2* |
| 9 | 23090001 | 23100000 | 0.1908 | 88 | *IRS1* |
| 11 | 6080001 | 6090000 | 0.1903 | 89 | *Gtf2a2* |
| 11 | 9690001 | 9700000 | 0.1939 | 68 | *REC114* |
| 11 | 9790001 | 9800000 | 0.2404 | 107 | *HCN4* |
| 11 | 9800001 | 9810000 | 0.1946 | 116 | *HCN4* |
| 11 | 9810001 | 9820000 | 0.1880 | 107 | *HCN4* |
| 11 | 9960001 | 9970000 | 0.1936 | 89 | *PCSK6* |
| 11 | 11460001 | 11470000 | 0.1867 | 89 | *USP8* |
| 11 | 11890001 | 11900000 | 0.1923 | 114 | *TM6SF1* |
| 11 | 15700001 | 15710000 | 0.2227 | 87 | *CHD2* |
| 11 | 16080001 | 16090000 | 0.1935 | 92 | *MCTP2* |
| 11 | 16090001 | 16100000 | 0.1925 | 73 | *MCTP2* |
| 11 | 18760001 | 18770000 | 0.2061 | 138 | *LRRK1* |
| 11 | 19590001 | 19600000 | 0.1926 | 76 | *MEGF11* |
| 11 | 19620001 | 19630000 | 0.2112 | 59 | *MEGF11* |
| 11 | 19650001 | 19660000 | 0.1937 | 47 | *MEGF11* |
| 12 | 8270001 | 8280000 | 0.2325 | 125 | *gna0* |
| 12 | 8460001 | 8470000 | 0.2046 | 76 | *NUP93* |
| 12 | 8470001 | 8480000 | 0.2147 | 143 | *NUP93* |
| 12 | 10060001 | 10070000 | 0.1929 | 76 | *Rspry1* |
| 12 | 13080001 | 13090000 | 0.2205 | 94 | *CDH11* |
| 12 | 13090001 | 13100000 | 0.1882 | 85 | *CDH11* |
| 12 | 14060001 | 14070000 | 0.2151 | 47 | *CDH8* |
| 12 | 15130001 | 15140000 | 0.2131 | 105 | *HPRT1* |
| 12 | 15140001 | 15150000 | 0.1896 | 90 | *HPRT1* |
| 12 | 15140001 | 15150000 | 0.1896 | 90 | *NUDT7* |
| 12 | 15180001 | 15190000 | 0.1879 | 109 | *Vat1l* |
| 12 | 15220001 | 15230000 | 0.1909 | 94 | *CLEC3A* |
| 12 | 15720001 | 15730000 | 0.2399 | 97 | *WWOX* |
| 13 | 12950001 | 12960000 | 0.2227 | 108 | *Aff2* |
| 13 | 12960001 | 12970000 | 0.2148 | 85 | *Aff2* |
| 13 | 12970001 | 12980000 | 0.2133 | 66 | *Aff2* |
| 13 | 13000001 | 13010000 | 0.1903 | 125 | *Aff2* |
| 13 | 15780001 | 15790000 | 0.2078 | 88 | *VAMP7* |
| 13 | 15780001 | 15790000 | 0.2078 | 88 | *chr26.372* |
| 13 | 15780001 | 15790000 | 0.2078 | 88 | *wnt11b* |
| 13 | 15800001 | 15810000 | 0.1940 | 117 | *wnt11b* |
| 13 | 15800001 | 15810000 | 0.1940 | 117 | *Rhog* |
| 14 | 6800001 | 6810000 | 0.1871 | 118 | *CTNNA1* |
| 14 | 13020001 | 13030000 | 0.2142 | 63 | *Gria1* |
| 14 | 13030001 | 13040000 | 0.2207 | 56 | *Gria1* |
| 14 | 13420001 | 13430000 | 0.2027 | 126 | *NMUR2* |
| 14 | 17900001 | 17910000 | 0.1975 | 96 | *FNIP1* |
| 14 | 18520001 | 18530000 | 0.1885 | 140 | *Zcchc10* |
| 14 | 19580001 | 19590000 | 0.1924 | 29 | *Tcerg1* |
| 14 | 19580001 | 19590000 | 0.1924 | 29 | *chr25.497* |
| 15 | 5790001 | 5800000 | 0.2397 | 146 | *INTS1* |
| 15 | 8250001 | 8260000 | 0.2119 | 76 | *Mchr1* |
| 15 | 11060001 | 11070000 | 0.1998 | 92 | *KIAA0556* |
| 15 | 11290001 | 11300000 | 0.1872 | 113 | *Tmem8a* |
| 15 | 11520001 | 11530000 | 0.2033 | 169 | *ECI1* |
| 15 | 11520001 | 11530000 | 0.2033 | 169 | *DNASE1L2* |
| 15 | 11730001 | 11740000 | 0.2043 | 86 | *TRAP1* |
| 16 | 2150001 | 2160000 | 0.1954 | 135 | *TOP1* |
| 16 | 3990001 | 4000000 | 0.1919 | 101 | *PTPRT* |
| 16 | 9820001 | 9830000 | 0.2067 | 86 | *MC3R* |
| 17 | 2360001 | 2370000 | 0.1975 | 111 | *MED13L* |
| 17 | 3740001 | 3750000 | 0.2030 | 124 | *Ccdc60* |
| 17 | 3790001 | 3800000 | 0.2149 | 123 | *chr22.120* |
| 17 | 3790001 | 3800000 | 0.2149 | 123 | *CIT* |
| 17 | 3810001 | 3820000 | 0.1925 | 54 | *CIT* |
| 17 | 3860001 | 3870000 | 0.1901 | 139 | *CIT* |
| 17 | 5180001 | 5190000 | 0.1926 | 95 | *GGT1* |
| 17 | 5310001 | 5320000 | 0.2493 | 116 | *Crkl* |
| 17 | 5320001 | 5330000 | 0.1920 | 79 | *Crkl* |
| 17 | 5440001 | 5450000 | 0.1955 | 110 | *Cabin1* |
| 17 | 5460001 | 5470000 | 0.2712 | 82 | *Cabin1* |
| 17 | 5470001 | 5480000 | 0.2750 | 64 | *CABIN1* |
| 17 | 5470001 | 5480000 | 0.2750 | 64 | *CABIN1* |
| 17 | 5480001 | 5490000 | 0.2015 | 79 | *CABIN1* |
| 17 | 8140001 | 8150000 | 0.2488 | 87 | *AUTS2* |
| 17 | 10640001 | 10650000 | 0.2276 | 102 | *Tmem132c* |
| 17 | 12410001 | 12420000 | 0.1979 | 95 | *Ift81* |
| 17 | 12440001 | 12450000 | 0.1889 | 54 | *P2RX7* |
| 17 | 12660001 | 12670000 | 0.1888 | 142 | *RHOF* |
| 18 | 8650001 | 8660000 | 0.1920 | 64 | *ATAD5* |
| 18 | 8740001 | 8750000 | 0.1966 | 73 | *Rhbdl3* |
| 19 | 150001 | 160000 | 0.1880 | 33 | *MAN1B1* |
| 19 | 160001 | 170000 | 0.2114 | 54 | *MAN1B1* |
| 19 | 160001 | 170000 | 0.2114 | 54 | *chr19.8* |
| 19 | 160001 | 170000 | 0.2114 | 54 | *uap1l1* |
| 19 | 170001 | 180000 | 0.2190 | 72 | *uap1l1* |
| 19 | 1120001 | 1130000 | 0.1932 | 108 | *ZNF618* |
| 19 | 1990001 | 2000000 | 0.1889 | 150 | *DDR2* |
| 19 | 3760001 | 3770000 | 0.2275 | 162 | *ASTN2* |
| 19 | 3770001 | 3780000 | 0.2039 | 120 | *ASTN2* |
| 20 | 2070001 | 2080000 | 0.1975 | 72 | *LHX1* |
| 20 | 2560001 | 2570000 | 0.1876 | 114 | *APPBP2* |
| 20 | 2700001 | 2710000 | 0.1942 | 95 | *BCAS3* |
| 20 | 8390001 | 8400000 | 0.1888 | 113 | *Spns3* |
| 20 | 8950001 | 8960000 | 0.1876 | 154 | *GTF2I* |
| 21 | 2500001 | 2510000 | 0.1969 | 129 | *PDPN* |
| 21 | 2590001 | 2600000 | 0.2159 | 77 | *AADACL4* |
| 21 | 2600001 | 2610000 | 0.1971 | 84 | *AADACL4* |
| 21 | 5510001 | 5520000 | 0.1894 | 178 | *ACAP3* |
| 24 | 2540001 | 2550000 | 0.2729 | 51 | *NUCKS1* |
| 26 | 870001 | 880000 | 0.2193 | 74 | *Ap3d1* |
| 26 | 880001 | 890000 | 0.2329 | 69 | *Ap3d1* |
| 26 | 4240001 | 4250000 | 0.1920 | 180 | *ELL* |
| 29 | 750001 | 760000 | 0.1914 | 12 | *col2a1* |

Note: This table lists the genes within the top 1% frequency difference regions, indicating potential areas of positive selection in the CLG genome when compared with five other indigenous goose breeds. The “Frequency Difference” column reflects the allele frequency contrast between CLG and the other breeds, with a higher value suggesting a stronger selection signal. “Number of SNPs” refers to the count of single nucleotide polymorphisms (SNPs) within the specified genomic window.

**Table S6. Associated genes of significant Fst values in the Changle goose.**

| Chromosome | Start Position (bp) | End Position (bp) | Fst | Number of SNP | Associated Gene(s) |
| --- | --- | --- | --- | --- | --- |
| 1 | 8850001 | 8860000 | 0.1670 | 101 | *GRM5* |
| 1 | 16940001 | 16950000 | 0.1769 | 112 | *ELMOD1* |
| 1 | 21100001 | 21110000 | 0.1502 | 82 | *RNF6* |
| 1 | 27850001 | 27860000 | 0.1699 | 81 | *NEK5* |
| 1 | 27850001 | 27860000 | 0.1699 | 81 | *ALG11* |
| 1 | 92300001 | 92310000 | 0.1609 | 62 | *TRAPPC10* |
| 1 | 92360001 | 92370000 | 0.1887 | 91 | *AGPAT3* |
| 1 | 92500001 | 92510000 | 0.2101 | 123 | *Pdxk* |
| 1 | 92520001 | 92530000 | 0.1808 | 78 | *Pdxk* |
| 1 | 95870001 | 95880000 | 0.1522 | 56 | *HLCS* |
| 1 | 95900001 | 95910000 | 0.1513 | 84 | *Sim2* |
| 1 | 110210001 | 110220000 | 0.1513 | 27 | *FSTL1* |
| 1 | 114380001 | 114390000 | 0.1875 | 94 | *chr38.1696* |
| 1 | 114460001 | 114470000 | 0.1523 | 151 | *Gpr161* |
| 1 | 114460001 | 114470000 | 0.1523 | 151 | *TTF2* |
| 1 | 114480001 | 114490000 | 0.2020 | 61 | *TTF2* |
| 1 | 116890001 | 116900000 | 0.1731 | 45 | *ALCAM* |
| 1 | 116900001 | 116910000 | 0.1558 | 20 | *ALCAM* |
| 1 | 116920001 | 116930000 | 0.1857 | 55 | *ALCAM* |
| 1 | 116930001 | 116940000 | 0.2710 | 56 | *ALCAM* |
| 1 | 116940001 | 116950000 | 0.2045 | 32 | *ALCAM* |
| 1 | 116960001 | 116970000 | 0.2646 | 46 | *ALCAM* |
| 1 | 117130001 | 117140000 | 0.1728 | 33 | *CBLB* |
| 1 | 117690001 | 117700000 | 0.1513 | 26 | *BBX* |
| 1 | 117700001 | 117710000 | 0.1546 | 42 | *BBX* |
| 1 | 122510001 | 122520000 | 0.1482 | 89 | *PTGFRN* |
| 1 | 122530001 | 122540000 | 0.2864 | 95 | *PTGFRN* |
| 1 | 122560001 | 122570000 | 0.2540 | 28 | *PTGFRN* |
| 1 | 122560001 | 122570000 | 0.2540 | 28 | *Igsf3* |
| 1 | 135530001 | 135540000 | 0.1577 | 47 | *prr5* |
| 1 | 135570001 | 135580000 | 0.1705 | 36 | *prr5* |
| 1 | 135720001 | 135730000 | 0.1536 | 25 | *ARHGAP8* |
| 1 | 135780001 | 135790000 | 0.2068 | 14 | *Phf21b* |
| 1 | 135790001 | 135800000 | 0.2265 | 14 | *Phf21b* |
| 1 | 135800001 | 135810000 | 0.1565 | 24 | *Phf21b* |
| 1 | 136060001 | 136070000 | 0.1734 | 30 | *NUP50* |
| 1 | 136090001 | 136100000 | 0.1719 | 42 | *KIAA0930* |
| 1 | 136090001 | 136100000 | 0.1719 | 42 | *Adck2* |
| 1 | 145570001 | 145580000 | 0.1580 | 107 | *DENND5B* |
| 1 | 148490001 | 148500000 | 0.1603 | 69 | *KDM7A* |
| 1 | 148530001 | 148540000 | 0.1506 | 73 | *KDM7A* |
| 1 | 151630001 | 151640000 | 0.1580 | 93 | *RFX4* |
| 1 | 155050001 | 155060000 | 0.1610 | 44 | *Cacna1i* |
| 1 | 156520001 | 156530000 | 0.1486 | 81 | *Tmtc2* |
| 1 | 157620001 | 157630000 | 0.1560 | 48 | *LRRIQ1* |
| 1 | 157640001 | 157650000 | 0.1802 | 36 | *LRRIQ1* |
| 1 | 157660001 | 157670000 | 0.1981 | 19 | *LRRIQ1* |
| 1 | 157670001 | 157680000 | 0.1940 | 10 | *LRRIQ1* |
| 1 | 157690001 | 157700000 | 0.1482 | 15 | *LRRIQ1* |
| 1 | 158910001 | 158920000 | 0.1574 | 37 | *WBP2* |
| 1 | 163530001 | 163540000 | 0.1525 | 39 | *Anks1b* |
| 1 | 165960001 | 165970000 | 0.1622 | 76 | *3-Sep* |
| 1 | 166230001 | 166240000 | 0.1487 | 35 | *ZC3H7B* |
| 1 | 166240001 | 166250000 | 0.1740 | 62 | *ZC3H7B* |
| 1 | 166260001 | 166270000 | 0.1860 | 74 | *chr38.2560* |
| 1 | 166260001 | 166270000 | 0.1860 | 74 | *RANGAP1* |
| 1 | 167050001 | 167060000 | 0.1572 | 124 | *Pawr* |
| 1 | 167090001 | 167100000 | 0.1723 | 49 | *Pawr* |
| 1 | 167130001 | 167140000 | 0.1511 | 86 | *SYT1* |
| 1 | 167180001 | 167190000 | 0.1550 | 69 | *SYT1* |
| 1 | 167190001 | 167200000 | 0.2036 | 54 | *SYT1* |
| 1 | 173160001 | 173170000 | 0.1600 | 85 | *Tbk1* |
| 1 | 176230001 | 176240000 | 0.2269 | 80 | *SCAF11* |
| 1 | 176250001 | 176260000 | 0.3087 | 61 | *SCAF11* |
| 1 | 176260001 | 176270000 | 0.1791 | 73 | *SCAF11* |
| 1 | 176270001 | 176280000 | 0.1775 | 94 | *SCAF11* |
| 1 | 176290001 | 176300000 | 0.2084 | 64 | *ARID2* |
| 1 | 176310001 | 176320000 | 0.1596 | 38 | *ARID2* |
| 1 | 176320001 | 176330000 | 0.2083 | 40 | *ARID2* |
| 1 | 176330001 | 176340000 | 0.2025 | 53 | *ARID2* |
| 1 | 176370001 | 176380000 | 0.1579 | 78 | *ARID2* |
| 1 | 178700001 | 178710000 | 0.2291 | 101 | *PNPLA8* |
| 1 | 178710001 | 178720000 | 0.3103 | 71 | *PNPLA8* |
| 1 | 178720001 | 178730000 | 0.2441 | 101 | *PNPLA8* |
| 1 | 178720001 | 178730000 | 0.2441 | 101 | *ARSD* |
| 1 | 178730001 | 178740000 | 0.1504 | 113 | *ARSD* |
| 1 | 178750001 | 178760000 | 0.1696 | 155 | *ARSD* |
| 1 | 178760001 | 178770000 | 0.1621 | 123 | *ARSD* |
| 1 | 179730001 | 179740000 | 0.1496 | 76 | *IMMP2L* |
| 1 | 202260001 | 202270000 | 0.2164 | 111 | *FRMD4A* |
| 1 | 202270001 | 202280000 | 0.1898 | 124 | *FRMD4A* |
| 1 | 204830001 | 204840000 | 0.1963 | 67 | *GATA3* |
| 1 | 204870001 | 204880000 | 0.1875 | 131 | *TAF3* |
| 1 | 204910001 | 204920000 | 0.1841 | 141 | *TAF3* |
| 1 | 204930001 | 204940000 | 0.1880 | 131 | *TAF3* |
| 1 | 204940001 | 204950000 | 0.2209 | 90 | *TAF3* |
| 1 | 205250001 | 205260000 | 0.1577 | 72 | *SFMBT2* |
| 1 | 205690001 | 205700000 | 0.1576 | 94 | *IL2RA* |
| 1 | 205700001 | 205710000 | 0.1670 | 108 | *IL2RA* |
| 1 | 205710001 | 205720000 | 0.1594 | 134 | *IL2RA* |
| 1 | 206950001 | 206960000 | 0.1519 | 153 | *Chchd3* |
| 1 | 206960001 | 206970000 | 0.2091 | 141 | *Chchd3* |
| 2 | 11430001 | 11440000 | 0.1632 | 93 | *Nsmce2* |
| 2 | 11460001 | 11470000 | 0.1856 | 89 | *Nsmce2* |
| 2 | 11470001 | 11480000 | 0.1738 | 69 | *Nsmce2* |
| 2 | 18820001 | 18830000 | 0.1759 | 59 | *EIF3E* |
| 2 | 20850001 | 20860000 | 0.2115 | 52 | *Rims2* |
| 2 | 23930001 | 23940000 | 0.1601 | 77 | *PTDSS1* |
| 2 | 23930001 | 23940000 | 0.1601 | 77 | *MTERF3* |
| 2 | 24460001 | 24470000 | 0.1823 | 114 | *INTS8* |
| 2 | 28350001 | 28360000 | 0.1643 | 44 | *lrrcc1* |
| 2 | 28350001 | 28360000 | 0.1643 | 44 | *LRRCC1* |
| 2 | 28360001 | 28370000 | 0.1512 | 61 | *LRRCC1* |
| 2 | 28470001 | 28480000 | 0.1502 | 40 | *RALYL* |
| 2 | 29870001 | 29880000 | 0.1690 | 113 | *FABP4* |
| 2 | 29880001 | 29890000 | 0.1612 | 83 | *FABP4* |
| 2 | 29890001 | 29900000 | 0.1687 | 128 | *FABP4* |
| 2 | 30060001 | 30070000 | 0.1574 | 83 | *PAG1* |
| 2 | 30080001 | 30090000 | 0.1627 | 104 | *PAG1* |
| 2 | 30210001 | 30220000 | 0.1665 | 86 | *ZNF704* |
| 2 | 31060001 | 31070000 | 0.1755 | 59 | *PKIA* |
| 2 | 36740001 | 36750000 | 0.1523 | 75 | *dnajc5* |
| 2 | 36910001 | 36920000 | 0.1572 | 81 | *ARMC1* |
| 2 | 52520001 | 52530000 | 0.1730 | 39 | *EPB41L3* |
| 2 | 59750001 | 59760000 | 0.1908 | 46 | *SOCS6* |
| 2 | 61550001 | 61560000 | 0.1625 | 76 | *FBXO15* |
| 2 | 61640001 | 61650000 | 0.2083 | 91 | *C18orf63* |
| 2 | 62700001 | 62710000 | 0.1691 | 56 | *ZNF516* |
| 2 | 62710001 | 62720000 | 0.2281 | 67 | *ZNF516* |
| 2 | 63840001 | 63850000 | 0.1589 | 107 | *Bloc1s4* |
| 2 | 64700001 | 64710000 | 0.1743 | 88 | *AHRR* |
| 2 | 65860001 | 65870000 | 0.1776 | 102 | *GALNT12* |
| 2 | 94920001 | 94930000 | 0.1579 | 31 | *TMC2* |
| 2 | 108380001 | 108390000 | 0.1914 | 40 | *KIF13A* |
| 2 | 109670001 | 109680000 | 0.1666 | 57 | *Cdkal1* |
| 2 | 115940001 | 115950000 | 0.1526 | 73 | *SH3BP5* |
| 2 | 123670001 | 123680000 | 0.1558 | 92 | *BTD* |
| 2 | 123670001 | 123680000 | 0.1558 | 92 | *Hacl1* |
| 2 | 123680001 | 123690000 | 0.1551 | 96 | *Hacl1* |
| 2 | 126810001 | 126820000 | 0.1564 | 91 | *KLHL7* |
| 2 | 126850001 | 126860000 | 0.2525 | 85 | *FAM126A* |
| 2 | 130790001 | 130800000 | 0.1857 | 16 | *ETV1* |
| 2 | 134720001 | 134730000 | 0.1670 | 47 | *Ppp1r9a* |
| 2 | 138330001 | 138340000 | 0.1663 | 145 | *FAM171A1* |
| 2 | 138580001 | 138590000 | 0.1660 | 79 | *MINDY3* |
| 3 | 4090001 | 4100000 | 0.1653 | 81 | *PKHD1* |
| 3 | 5120001 | 5130000 | 0.1671 | 74 | *RHAG* |
| 3 | 7620001 | 7630000 | 0.1967 | 120 | *WDCP* |
| 3 | 8140001 | 8150000 | 0.1746 | 112 | *KLHL29* |
| 3 | 10330001 | 10340000 | 0.1878 | 114 | *Pum2* |
| 3 | 10340001 | 10350000 | 0.1506 | 84 | *Pum2* |
| 3 | 15560001 | 15570000 | 0.1922 | 97 | *KCNF1* |
| 3 | 15650001 | 15660000 | 0.2703 | 46 | *PDIA6* |
| 3 | 15650001 | 15660000 | 0.2703 | 46 | *Atp6v1c2* |
| 3 | 15670001 | 15680000 | 0.1558 | 89 | *Atp6v1c2* |
| 3 | 15810001 | 15820000 | 0.1487 | 109 | *ODC1* |
| 3 | 19150001 | 19160000 | 0.1515 | 37 | *Trappc12* |
| 3 | 20120001 | 20130000 | 0.2109 | 31 | *TPO* |
| 3 | 20130001 | 20140000 | 0.2869 | 26 | *TPO* |
| 3 | 20140001 | 20150000 | 0.1615 | 43 | *TPO* |
| 3 | 20270001 | 20280000 | 0.1529 | 95 | *Sntg2* |
| 3 | 20340001 | 20350000 | 0.1634 | 74 | *Sntg2* |
| 3 | 21460001 | 21470000 | 0.1483 | 55 | *FBXO25* |
| 3 | 21490001 | 21500000 | 0.1570 | 101 | *tdrp* |
| 3 | 21620001 | 21630000 | 0.1676 | 134 | *ERICH1* |
| 3 | 28560001 | 28570000 | 0.1528 | 121 | *EYS* |
| 3 | 28870001 | 28880000 | 0.1570 | 92 | *eys* |
| 3 | 28890001 | 28900000 | 0.2059 | 62 | *eys* |
| 3 | 28980001 | 28990000 | 0.1872 | 72 | *eys* |
| 3 | 32770001 | 32780000 | 0.1604 | 104 | *SLC17A5* |
| 3 | 33280001 | 33290000 | 0.2740 | 31 | *chr36.476* |
| 3 | 33520001 | 33530000 | 0.1814 | 22 | *COL12A1* |
| 3 | 45130001 | 45140000 | 0.1782 | 85 | *Grik2* |
| 3 | 48870001 | 48880000 | 0.1537 | 41 | *Ccdc162* |
| 3 | 56770001 | 56780000 | 0.1515 | 72 | *CENPW* |
| 3 | 66620001 | 66630000 | 0.1515 | 39 | *SCAF8* |
| 3 | 66730001 | 66740000 | 0.2100 | 69 | *SCAF8* |
| 3 | 66970001 | 66980000 | 0.1517 | 39 | *IPCEF1* |
| 3 | 67520001 | 67530000 | 0.2328 | 43 | *RGS17* |
| 3 | 67530001 | 67540000 | 0.1552 | 41 | *MTRF1L* |
| 3 | 67540001 | 67550000 | 0.1610 | 48 | *MTRF1L* |
| 3 | 67540001 | 67550000 | 0.1610 | 48 | *FBXO5* |
| 3 | 86180001 | 86190000 | 0.1599 | 80 | *CRIM1* |
| 3 | 86210001 | 86220000 | 0.1516 | 57 | *CRIM1* |
| 3 | 86240001 | 86250000 | 0.2524 | 19 | *CRIM1* |
| 3 | 86250001 | 86260000 | 0.1748 | 21 | *CRIM1* |
| 3 | 86270001 | 86280000 | 0.1496 | 35 | *CRIM1* |
| 3 | 86300001 | 86310000 | 0.1535 | 74 | *CRIM1* |
| 3 | 86360001 | 86370000 | 0.1701 | 74 | *Fez2* |
| 3 | 86370001 | 86380000 | 0.2396 | 55 | *Fez2* |
| 3 | 86410001 | 86420000 | 0.1719 | 83 | *OPN5* |
| 3 | 86430001 | 86440000 | 0.1550 | 104 | *OPN5* |
| 3 | 86480001 | 86490000 | 0.1757 | 110 | *VIT* |
| 3 | 86530001 | 86540000 | 0.1511 | 66 | *STRN* |
| 3 | 87140001 | 87150000 | 0.1493 | 69 | *BIRC6* |
| 3 | 87170001 | 87180000 | 0.1932 | 60 | *BIRC6* |
| 3 | 98470001 | 98480000 | 0.1567 | 73 | *Aida* |
| 3 | 98470001 | 98480000 | 0.1567 | 73 | *MIA3* |
| 3 | 108250001 | 108260000 | 0.1890 | 11 | *CUL9* |
| 3 | 108650001 | 108660000 | 0.1780 | 15 | *Atl2* |
| 3 | 108840001 | 108850000 | 0.2235 | 18 | *SOS1* |
| 3 | 108850001 | 108860000 | 0.1845 | 12 | *SOS1* |
| 3 | 108860001 | 108870000 | 0.1885 | 12 | *SOS1* |
| 3 | 108910001 | 108920000 | 0.1684 | 11 | *Map4k3* |
| 3 | 108950001 | 108960000 | 0.1883 | 11 | *Map4k3* |
| 3 | 111110001 | 111120000 | 0.1640 | 10 | *Rin2* |
| 3 | 111300001 | 111310000 | 0.1782 | 12 | *SLC24A3* |
| 3 | 111550001 | 111560000 | 0.1538 | 16 | *RALGAPA2* |
| 3 | 112160001 | 112170000 | 0.2086 | 24 | *GZF1* |
| 3 | 112180001 | 112190000 | 0.1618 | 31 | *GZF1* |
| 3 | 112180001 | 112190000 | 0.1618 | 31 | *Napb* |
| 4 | 8890001 | 8900000 | 0.1706 | 113 | *POLN* |
| 4 | 8980001 | 8990000 | 0.1687 | 186 | *HAUS3* |
| 4 | 9250001 | 9260000 | 0.1583 | 124 | *CFAP99* |
| 4 | 9300001 | 9310000 | 0.2194 | 78 | *CFAP99* |
| 4 | 9300001 | 9310000 | 0.2194 | 78 | *Rnf4* |
| 4 | 12490001 | 12500000 | 0.1641 | 100 | *PPP2R2C* |
| 4 | 12920001 | 12930000 | 0.1656 | 80 | *CRMP1* |
| 4 | 13230001 | 13240000 | 0.1726 | 77 | *STK32B* |
| 4 | 13240001 | 13250000 | 0.1546 | 34 | *STK32B* |
| 4 | 13250001 | 13260000 | 0.2528 | 65 | *STK32B* |
| 4 | 13260001 | 13270000 | 0.1481 | 50 | *STK32B* |
| 4 | 28120001 | 28130000 | 0.1533 | 60 | *SCFD2* |
| 4 | 29970001 | 29980000 | 0.1621 | 19 | *ADAMTS3* |
| 4 | 30280001 | 30290000 | 0.1831 | 31 | *ALB* |
| 4 | 30310001 | 30320000 | 0.1517 | 34 | *AFP* |
| 4 | 30310001 | 30320000 | 0.1517 | 34 | *RASSF6* |
| 4 | 30440001 | 30450000 | 0.1483 | 51 | *MTHFD2L* |
| 4 | 30500001 | 30510000 | 0.1618 | 47 | *EREG* |
| 4 | 31750001 | 31760000 | 0.1647 | 38 | *PRKG2* |
| 4 | 31850001 | 31860000 | 0.1695 | 58 | *mdn1* |
| 4 | 36570001 | 36580000 | 0.2150 | 28 | *C4orf33* |
| 4 | 41270001 | 41280000 | 0.1560 | 18 | *chr35.636* |
| 4 | 45660001 | 45670000 | 0.1516 | 32 | *TRIM2* |
| 4 | 46180001 | 46190000 | 0.1495 | 40 | *PLRG1* |
| 4 | 60570001 | 60580000 | 0.2647 | 53 | *TUSC3* |
| 4 | 60580001 | 60590000 | 0.2752 | 45 | *TUSC3* |
| 4 | 60670001 | 60680000 | 0.1750 | 27 | *TUSC3* |
| 4 | 61100001 | 61110000 | 0.2645 | 34 | *SGCZ* |
| 4 | 61110001 | 61120000 | 0.2130 | 40 | *SGCZ* |
| 4 | 61120001 | 61130000 | 0.1807 | 24 | *SGCZ* |
| 4 | 61130001 | 61140000 | 0.1638 | 46 | *SGCZ* |
| 4 | 61150001 | 61160000 | 0.2545 | 21 | *SGCZ* |
| 4 | 70880001 | 70890000 | 0.1503 | 119 | *EMCN* |
| 5 | 16020001 | 16030000 | 0.1763 | 63 | *Asb2* |
| 5 | 16030001 | 16040000 | 0.1536 | 90 | *Asb2* |
| 5 | 18510001 | 18520000 | 0.1491 | 58 | *EML5* |
| 5 | 21530001 | 21540000 | 0.2363 | 44 | *CEP128* |
| 5 | 21830001 | 21840000 | 0.1855 | 15 | *TMEM251* |
| 5 | 21910001 | 21920000 | 0.2021 | 45 | *TMEM251* |
| 5 | 23350001 | 23360000 | 0.1512 | 46 | *Irf2bpl* |
| 5 | 23350001 | 23360000 | 0.1512 | 46 | *Irf2bpl* |
| 5 | 31450001 | 31460000 | 0.1881 | 80 | *RAD51A* |
| 5 | 31460001 | 31470000 | 0.1755 | 143 | *RAD51A* |
| 5 | 31510001 | 31520000 | 0.1514 | 107 | *RMDN3* |
| 5 | 33030001 | 33040000 | 0.1669 | 84 | *Zfyve19* |
| 5 | 34580001 | 34590000 | 0.2340 | 57 | *Tmem87a* |
| 5 | 34580001 | 34590000 | 0.2340 | 57 | *GANC* |
| 5 | 34590001 | 34600000 | 0.1824 | 56 | *GANC* |
| 5 | 34600001 | 34610000 | 0.2175 | 72 | *GANC* |
| 5 | 34600001 | 34610000 | 0.2175 | 72 | *CAPN3* |
| 5 | 34610001 | 34620000 | 0.2817 | 63 | *CAPN3* |
| 5 | 34620001 | 34630000 | 0.2092 | 52 | *CAPN3* |
| 5 | 34630001 | 34640000 | 0.1600 | 48 | *CAPN3* |
| 5 | 34640001 | 34650000 | 0.2073 | 50 | *ZNF106* |
| 5 | 34660001 | 34670000 | 0.2113 | 65 | *ZNF106* |
| 5 | 34670001 | 34680000 | 0.1987 | 54 | *Snap23* |
| 5 | 34730001 | 34740000 | 0.1573 | 57 | *STARD9* |
| 5 | 34740001 | 34750000 | 0.2363 | 81 | *STARD9* |
| 5 | 34770001 | 34780000 | 0.1691 | 35 | *STARD9* |
| 5 | 34790001 | 34800000 | 0.2716 | 54 | *STARD9* |
| 5 | 34790001 | 34800000 | 0.2716 | 54 | *STARD9* |
| 5 | 34800001 | 34810000 | 0.1522 | 39 | *STARD9* |
| 5 | 34850001 | 34860000 | 0.2121 | 19 | *TSNARE1* |
| 5 | 34860001 | 34870000 | 0.2355 | 10 | *TTBK2* |
| 5 | 34890001 | 34900000 | 0.1916 | 35 | *Ttbk2* |
| 5 | 34900001 | 34910000 | 0.1880 | 34 | *Ttbk2* |
| 5 | 34910001 | 34920000 | 0.1921 | 38 | *Ttbk2* |
| 5 | 34950001 | 34960000 | 0.1688 | 35 | *Slc35c1* |
| 5 | 34970001 | 34980000 | 0.1769 | 38 | *UBR1* |
| 5 | 35200001 | 35210000 | 0.1660 | 81 | *NUMB* |
| 5 | 35200001 | 35210000 | 0.1660 | 81 | *chr34.840* |
| 5 | 36510001 | 36520000 | 0.1564 | 40 | *Med6* |
| 5 | 37320001 | 37330000 | 0.1612 | 91 | *DCAF5* |
| 5 | 37580001 | 37590000 | 0.1537 | 62 | *RAD51B* |
| 5 | 37930001 | 37940000 | 0.1489 | 76 | *RAD51B* |
| 5 | 38090001 | 38100000 | 0.1933 | 73 | *arg2-a* |
| 5 | 38100001 | 38110000 | 0.1680 | 38 | *arg2-a* |
| 5 | 38100001 | 38110000 | 0.1680 | 38 | *VTI1B* |
| 5 | 38580001 | 38590000 | 0.1668 | 64 | *chr34.900* |
| 5 | 38660001 | 38670000 | 0.1800 | 65 | *Bmf* |
| 5 | 38780001 | 38790000 | 0.2296 | 28 | *EIF2AK4* |
| 5 | 38780001 | 38790000 | 0.2296 | 28 | *Gpr176* |
| 5 | 38790001 | 38800000 | 0.3058 | 65 | *Gpr176* |
| 5 | 38800001 | 38810000 | 0.2487 | 37 | *Gpr176* |
| 5 | 38810001 | 38820000 | 0.1610 | 92 | *Gpr176* |
| 5 | 38810001 | 38820000 | 0.1610 | 92 | *FSIP1* |
| 5 | 38820001 | 38830000 | 0.1532 | 39 | *FSIP1* |
| 5 | 39360001 | 39370000 | 0.2081 | 86 | *KATNBL1* |
| 5 | 39370001 | 39380000 | 0.2062 | 70 | *KATNBL1* |
| 5 | 39380001 | 39390000 | 0.1781 | 71 | *KATNBL1* |
| 5 | 39510001 | 39520000 | 0.1592 | 98 | *Aven* |
| 5 | 39520001 | 39530000 | 0.1638 | 59 | *Aven* |
| 5 | 39530001 | 39540000 | 0.1994 | 27 | *Aven* |
| 5 | 39530001 | 39540000 | 0.1994 | 27 | *RYR3* |
| 5 | 39540001 | 39550000 | 0.2388 | 26 | *RYR3* |
| 5 | 39660001 | 39670000 | 0.2258 | 35 | *RYR3* |
| 5 | 39670001 | 39680000 | 0.2283 | 51 | *RYR3* |
| 5 | 39680001 | 39690000 | 0.1920 | 80 | *RYR3* |
| 5 | 39690001 | 39700000 | 0.2120 | 65 | *RYR3* |
| 5 | 39750001 | 39760000 | 0.1895 | 65 | *RYR3* |
| 5 | 41620001 | 41630000 | 0.1707 | 60 | *DPH6* |
| 5 | 47490001 | 47500000 | 0.1492 | 61 | *TPCN2* |
| 5 | 49800001 | 49810000 | 0.1718 | 100 | *LDLRAD3* |
| 5 | 52910001 | 52920000 | 0.1612 | 118 | *CD82* |
| 5 | 61630001 | 61640000 | 0.1818 | 54 | *SOX6* |
| 6 | 17690001 | 17700000 | 0.1659 | 64 | *Tank* |
| 6 | 20160001 | 20170000 | 0.1971 | 68 | *Stk39* |
| 6 | 20170001 | 20180000 | 0.1706 | 72 | *Stk39* |
| 6 | 20230001 | 20240000 | 0.1513 | 83 | *Stk39* |
| 6 | 31340001 | 31350000 | 0.1507 | 92 | *COL6A3* |
| 7 | 1140001 | 1150000 | 0.1504 | 12 | *ADK* |
| 7 | 1450001 | 1460000 | 0.1550 | 53 | *DUSP13* |
| 7 | 1460001 | 1470000 | 0.1583 | 47 | *chr32.27* |
| 7 | 1460001 | 1470000 | 0.1583 | 47 | *SAMD8* |
| 7 | 1470001 | 1480000 | 0.1544 | 46 | *SAMD8* |
| 7 | 4880001 | 4890000 | 0.1691 | 95 | *RET* |
| 7 | 8940001 | 8950000 | 0.1550 | 38 | *CCSER2* |
| 7 | 15190001 | 15200000 | 0.1841 | 70 | *Pcdh15* |
| 7 | 15230001 | 15240000 | 0.1583 | 59 | *Pcdh15* |
| 7 | 17370001 | 17380000 | 0.1484 | 65 | *GBF1* |
| 7 | 17430001 | 17440000 | 0.1898 | 86 | *GBF1* |
| 7 | 18050001 | 18060000 | 0.1576 | 88 | *SCD* |
| 7 | 19060001 | 19070000 | 0.1692 | 58 | *WDFY4* |
| 7 | 19060001 | 19070000 | 0.1692 | 58 | *WDFY4* |
| 7 | 23890001 | 23900000 | 0.1674 | 99 | *BTRC* |
| 7 | 25280001 | 25290000 | 0.1727 | 73 | *SLK* |
| 7 | 25280001 | 25290000 | 0.1727 | 73 | *COL17A1* |
| 7 | 25290001 | 25300000 | 0.1580 | 32 | *COL17A1* |
| 8 | 3270001 | 3280000 | 0.2160 | 94 | *Mfsd14a* |
| 8 | 3280001 | 3290000 | 0.1569 | 83 | *Mfsd14a* |
| 8 | 3280001 | 3290000 | 0.1569 | 83 | *SASS6* |
| 8 | 3290001 | 3300000 | 0.2884 | 57 | *SASS6* |
| 8 | 3290001 | 3300000 | 0.2884 | 57 | *TRMT13* |
| 8 | 3300001 | 3310000 | 0.1594 | 53 | *TRMT13* |
| 8 | 3300001 | 3310000 | 0.1594 | 53 | *Lrrc39* |
| 8 | 3310001 | 3320000 | 0.1983 | 63 | *DBT* |
| 8 | 3320001 | 3330000 | 0.1485 | 37 | *DBT* |
| 8 | 3320001 | 3330000 | 0.1485 | 37 | *RTCA* |
| 8 | 12660001 | 12670000 | 0.1517 | 127 | *Rabgap1* |
| 8 | 15660001 | 15670000 | 0.1607 | 77 | *Rnf2* |
| 8 | 16390001 | 16400000 | 0.1759 | 39 | *KYAT3* |
| 8 | 25780001 | 25790000 | 0.1638 | 58 | *FAF1* |
| 8 | 25790001 | 25800000 | 0.1999 | 55 | *FAF1* |
| 8 | 25840001 | 25850000 | 0.1790 | 60 | *FAF1* |
| 8 | 25850001 | 25860000 | 0.2100 | 74 | *FAF1* |
| 8 | 25870001 | 25880000 | 0.1870 | 87 | *FAF1* |
| 8 | 25980001 | 25990000 | 0.1511 | 114 | *RNF11* |
| 8 | 26300001 | 26310000 | 0.1695 | 43 | *BTF3L4* |
| 8 | 26330001 | 26340000 | 0.2646 | 85 | *chr31.523* |
| 8 | 26340001 | 26350000 | 0.3683 | 45 | *chr31.523* |
| 8 | 26340001 | 26350000 | 0.3683 | 45 | *ZFYVE9* |
| 8 | 26340001 | 26350000 | 0.3683 | 45 | *ZFYVE9* |
| 8 | 26350001 | 26360000 | 0.3617 | 37 | *ZFYVE9* |
| 8 | 26360001 | 26370000 | 0.3805 | 69 | *ZFYVE9* |
| 8 | 26370001 | 26380000 | 0.2243 | 38 | *ZFYVE9* |
| 8 | 26430001 | 26440000 | 0.1527 | 53 | *ORC1* |
| 8 | 26430001 | 26440000 | 0.1527 | 53 | *PRPF38A* |
| 8 | 26430001 | 26440000 | 0.1527 | 53 | *TUT4* |
| 8 | 26660001 | 26670000 | 0.1565 | 89 | *SLC1A7* |
| 8 | 26680001 | 26690000 | 0.1529 | 81 | *SLC1A7* |
| 9 | 830001 | 840000 | 0.2446 | 52 | *MBNL1* |
| 9 | 840001 | 850000 | 0.1724 | 28 | *MBNL1* |
| 9 | 5190001 | 5200000 | 0.1522 | 112 | *TNIK* |
| 9 | 5560001 | 5570000 | 0.1933 | 84 | *FNDC3B* |
| 9 | 5600001 | 5610000 | 0.1736 | 73 | *FNDC3B* |
| 9 | 5650001 | 5660000 | 0.1578 | 70 | *NCEH1* |
| 9 | 5680001 | 5690000 | 0.1710 | 72 | *ECT2* |
| 9 | 5730001 | 5740000 | 0.1615 | 66 | *Spata16* |
| 9 | 5740001 | 5750000 | 0.1899 | 110 | *Spata16* |
| 9 | 7490001 | 7500000 | 0.1484 | 136 | *KCNMB2* |
| 9 | 8060001 | 8070000 | 0.1693 | 85 | *Fxr1* |
| 9 | 8080001 | 8090000 | 0.1489 | 44 | *Fxr1* |
| 9 | 8080001 | 8090000 | 0.1489 | 44 | *DNAJC19* |
| 9 | 8090001 | 8100000 | 0.1608 | 84 | *DNAJC19* |
| 9 | 8770001 | 8780000 | 0.1651 | 69 | *ATP11B* |
| 9 | 8840001 | 8850000 | 0.2385 | 98 | *DCUN1D1* |
| 9 | 8900001 | 8910000 | 0.1562 | 70 | *MCF2L* |
| 9 | 8920001 | 8930000 | 0.1679 | 68 | *MCF2L* |
| 9 | 8940001 | 8950000 | 0.2215 | 59 | *MCF2L* |
| 9 | 8950001 | 8960000 | 0.1953 | 116 | *MCF2L* |
| 9 | 8960001 | 8970000 | 0.2423 | 42 | *MCF2L* |
| 9 | 8990001 | 9000000 | 0.1505 | 48 | *MCF2L* |
| 9 | 9000001 | 9010000 | 0.1517 | 35 | *MCF2L* |
| 9 | 10300001 | 10310000 | 0.1769 | 106 | *PSMD1* |
| 9 | 10320001 | 10330000 | 0.1941 | 68 | *PSMD1* |
| 9 | 10360001 | 10370000 | 0.2034 | 91 | *PSMD1* |
| 9 | 10580001 | 10590000 | 0.1639 | 148 | *TNK2* |
| 9 | 10590001 | 10600000 | 0.1697 | 75 | *TFRC* |
| 9 | 11050001 | 11060000 | 0.1654 | 73 | *LPP* |
| 9 | 11090001 | 11100000 | 0.2047 | 79 | *LPP* |
| 9 | 15320001 | 15330000 | 0.1975 | 70 | *WDR53* |
| 9 | 15320001 | 15330000 | 0.1975 | 70 | *Fbxo45* |
| 9 | 15330001 | 15340000 | 0.1523 | 76 | *Nrros* |
| 9 | 15340001 | 15350000 | 0.1940 | 57 | *Nrros* |
| 9 | 15340001 | 15350000 | 0.1940 | 57 | *chr30.390* |
| 9 | 15340001 | 15350000 | 0.1940 | 57 | *CEP19* |
| 9 | 15340001 | 15350000 | 0.1940 | 57 | *Pigx* |
| 9 | 15470001 | 15480000 | 0.1592 | 94 | *Slco2a1* |
| 9 | 19860001 | 19870000 | 0.1839 | 33 | *CLSTN2* |
| 10 | 18580001 | 18590000 | 0.1733 | 93 | *CHCHD4* |
| 11 | 11300001 | 11310000 | 0.1843 | 103 | *TRDC* |
| 11 | 11310001 | 11320000 | 0.1528 | 61 | *TRDC* |
| 11 | 19570001 | 19580000 | 0.1485 | 103 | *MEGF11* |
| 11 | 19590001 | 19600000 | 0.1640 | 68 | *MEGF11* |
| 11 | 19620001 | 19630000 | 0.2414 | 47 | *MEGF11* |
| 11 | 19630001 | 19640000 | 0.1865 | 48 | *MEGF11* |
| 11 | 19640001 | 19650000 | 0.2445 | 31 | *MEGF11* |
| 11 | 19650001 | 19660000 | 0.1620 | 34 | *MEGF11* |
| 11 | 19670001 | 19680000 | 0.1482 | 98 | *MEGF11* |
| 11 | 19700001 | 19710000 | 0.1713 | 27 | *MEGF11* |
| 12 | 2760001 | 2770000 | 0.2148 | 68 | *PHKB* |
| 12 | 13070001 | 13080000 | 0.1587 | 46 | *CDH11* |
| 12 | 13080001 | 13090000 | 0.1984 | 59 | *CDH11* |
| 12 | 13090001 | 13100000 | 0.1534 | 61 | *CDH11* |
| 12 | 13980001 | 13990000 | 0.1605 | 69 | *CDH8* |
| 12 | 14060001 | 14070000 | 0.1502 | 39 | *CDH8* |
| 12 | 15720001 | 15730000 | 0.1876 | 74 | *WWOX* |
| 13 | 12630001 | 12640000 | 0.1587 | 54 | *fmr1-b* |
| 13 | 12950001 | 12960000 | 0.1731 | 83 | *Aff2* |
| 13 | 12960001 | 12970000 | 0.1765 | 60 | *Aff2* |
| 13 | 12970001 | 12980000 | 0.1603 | 54 | *Aff2* |
| 13 | 13010001 | 13020000 | 0.1523 | 63 | *Aff2* |
| 13 | 15760001 | 15770000 | 0.1574 | 41 | *POLR1D* |
| 13 | 15760001 | 15770000 | 0.1574 | 41 | *VAMP7* |
| 13 | 17790001 | 17800000 | 0.2053 | 28 | *Trpc5* |
| 13 | 17800001 | 17810000 | 0.1695 | 26 | *Trpc5* |
| 13 | 17990001 | 18000000 | 0.1832 | 61 | *DCX* |
| 13 | 20520001 | 20530000 | 0.1584 | 51 | *Stag2* |
| 14 | 4370001 | 4380000 | 0.1744 | 67 | *WWC1* |
| 14 | 4370001 | 4380000 | 0.1744 | 67 | *RARS* |
| 14 | 12690001 | 12700000 | 0.1690 | 54 | *Gemin5* |
| 14 | 12690001 | 12700000 | 0.1690 | 54 | *CNOT8* |
| 14 | 13020001 | 13030000 | 0.1554 | 52 | *Gria1* |
| 14 | 13420001 | 13430000 | 0.1603 | 90 | *NMUR2* |
| 14 | 19580001 | 19590000 | 0.1735 | 22 | *Tcerg1* |
| 14 | 19580001 | 19590000 | 0.1735 | 22 | *chr25.497* |
| 14 | 19810001 | 19820000 | 0.1900 | 37 | *JAKMIP2* |
| 14 | 19820001 | 19830000 | 0.1707 | 23 | *JAKMIP2* |
| 15 | 5280001 | 5290000 | 0.1584 | 76 | *MAD1L1* |
| 15 | 9970001 | 9980000 | 0.1536 | 18 | *MARF1* |
| 15 | 10210001 | 10220000 | 0.1619 | 62 | *SNX29* |
| 15 | 10240001 | 10250000 | 0.1973 | 69 | *SNX29* |
| 15 | 10320001 | 10330000 | 0.2593 | 50 | *SNX29* |
| 15 | 10370001 | 10380000 | 0.1717 | 43 | *Cpped1* |
| 15 | 10390001 | 10400000 | 0.1800 | 55 | *Cpped1* |
| 15 | 11070001 | 11080000 | 0.1889 | 66 | *KIAA0556* |
| 15 | 11290001 | 11300000 | 0.1593 | 96 | *Tmem8a* |
| 15 | 11520001 | 11530000 | 0.1593 | 144 | *ECI1* |
| 15 | 11520001 | 11530000 | 0.1593 | 144 | *DNASE1L2* |
| 15 | 11730001 | 11740000 | 0.1877 | 67 | *TRAP1* |
| 15 | 11750001 | 11760000 | 0.1661 | 56 | *TRAP1* |
| 15 | 17230001 | 17240000 | 0.1605 | 15 | *Ciita* |
| 16 | 3950001 | 3960000 | 0.1763 | 100 | *PTPRT* |
| 16 | 3960001 | 3970000 | 0.1486 | 110 | *PTPRT* |
| 16 | 3990001 | 4000000 | 0.1975 | 82 | *PTPRT* |
| 16 | 4020001 | 4030000 | 0.1487 | 102 | *PTPRT* |
| 16 | 4040001 | 4050000 | 0.1554 | 112 | *PTPRT* |
| 16 | 4050001 | 4060000 | 0.1543 | 103 | *PTPRT* |
| 16 | 7210001 | 7220000 | 0.1779 | 63 | *STAU1* |
| 17 | 170001 | 180000 | 0.1778 | 10 | *SMTN* |
| 17 | 170001 | 180000 | 0.1778 | 10 | *selenom* |
| 17 | 190001 | 200000 | 0.3026 | 19 | *Inpp5j* |
| 17 | 190001 | 200000 | 0.3026 | 19 | *PLA2G3* |
| 17 | 190001 | 200000 | 0.3026 | 19 | *PLA2G3* |
| 17 | 200001 | 210000 | 0.2639 | 14 | *P2RX2* |
| 17 | 200001 | 210000 | 0.2639 | 14 | *POLE* |
| 17 | 210001 | 220000 | 0.1766 | 20 | *POLE* |
| 17 | 220001 | 230000 | 0.1736 | 24 | *POLE* |
| 17 | 280001 | 290000 | 0.1596 | 25 | *AIFM3* |
| 17 | 3740001 | 3750000 | 0.1518 | 101 | *Ccdc60* |
| 17 | 3780001 | 3790000 | 0.1751 | 75 | *PRKAB1* |
| 17 | 3790001 | 3800000 | 0.1634 | 113 | *chr22.120* |
| 17 | 3790001 | 3800000 | 0.1634 | 113 | *CIT* |
| 17 | 10530001 | 10540000 | 0.1512 | 62 | *TMEM132C* |
| 17 | 10640001 | 10650000 | 0.2086 | 80 | *Tmem132c* |
| 17 | 13460001 | 13470000 | 0.1535 | 66 | *HECTD4* |
| 18 | 920001 | 930000 | 0.1599 | 110 | *DNAH9* |
| 18 | 6600001 | 6610000 | 0.1638 | 88 | *Spag9* |
| 18 | 6890001 | 6900000 | 0.2098 | 32 | *CA10* |
| 18 | 7630001 | 7640000 | 0.1496 | 58 | *TOM1L1* |
| 18 | 8330001 | 8340000 | 0.1897 | 54 | *RAB11FIP4* |
| 18 | 8730001 | 8740000 | 0.1634 | 22 | *Rhbdl3* |
| 18 | 8740001 | 8750000 | 0.2710 | 64 | *Rhbdl3* |
| 18 | 8750001 | 8760000 | 0.1964 | 26 | *Rhbdl3* |
| 18 | 8760001 | 8770000 | 0.2912 | 34 | *Rhbdl3* |
| 18 | 8770001 | 8780000 | 0.2052 | 25 | *Rhbdl3* |
| 18 | 8780001 | 8790000 | 0.1812 | 26 | *C17orf75* |
| 18 | 9090001 | 9100000 | 0.1524 | 79 | *KPNA2* |
| 19 | 620001 | 630000 | 0.1521 | 84 | *EXD3* |
| 19 | 3760001 | 3770000 | 0.1821 | 138 | *ASTN2* |
| 20 | 6240001 | 6250000 | 0.1652 | 95 | *CRCP* |
| 21 | 2500001 | 2510000 | 0.1702 | 106 | *PDPN* |
| 21 | 2590001 | 2600000 | 0.1536 | 82 | *AADACL4* |
| 21 | 2600001 | 2610000 | 0.2087 | 68 | *AADACL4* |
| 21 | 2610001 | 2620000 | 0.1550 | 54 | *AADACL4* |
| 21 | 2620001 | 2630000 | 0.1935 | 42 | *AADACL4* |
| 21 | 5510001 | 5520000 | 0.1626 | 141 | *ACAP3* |
| 22 | 970001 | 980000 | 0.1775 | 98 | *Ctps1* |
| 22 | 4830001 | 4840000 | 0.1485 | 85 | *ZC3H12A* |
| 22 | 7690001 | 7700000 | 0.1925 | 41 | *Rhd* |
| 24 | 520001 | 530000 | 0.1517 | 31 | *Phtf1* |
| 24 | 1340001 | 1350000 | 0.1557 | 133 | *chr15.54* |
| 24 | 1340001 | 1350000 | 0.1557 | 133 | *GUCA1A* |
| 24 | 1360001 | 1370000 | 0.1764 | 67 | *Irf6* |
| 24 | 1360001 | 1370000 | 0.1764 | 67 | *MAGI3* |
| 24 | 1360001 | 1370000 | 0.1764 | 67 | *chr15.59* |
| 24 | 2540001 | 2550000 | 0.2673 | 45 | *NUCKS1* |
| 26 | 4240001 | 4250000 | 0.1751 | 144 | *ELL* |

Note: This table outlines the genes associated with the top percentile of Fst values (top 1%), indicating strong selective pressure within the CLG genome relative to other indigenous breeds. “Fst Value” reflects the extent of genetic divergence, with higher values suggesting stronger selective pressure. “Number of SNPs” denotes the number of SNPs identified within these regions. The “Associated Gene(s)” column lists the genes that coincide with these high-Fst regions, which are candidates for influencing the distinctive traits of the Changle goose.

**Table S7. Associated genes of significant XP-CLR values in Changle goose.**

| Chromosome | Start Position (bp) | End Position (bp) | XP-CLR score | Associated Gene(s) |
| --- | --- | --- | --- | --- |
| 1 | 1615001 | 1625000 | 85.92 | *PAAF1* |
| 1 | 1615001 | 1625000 | 85.92 | *COA4* |
| 1 | 3830001 | 3840000 | 190.30 | *KCTD14* |
| 1 | 3835001 | 3845000 | 225.91 | *chr38.168* |
| 1 | 3835001 | 3845000 | 225.91 | *mid1ip1b* |
| 1 | 3850001 | 3860000 | 119.23 | *mid1ip1b* |
| 1 | 3850001 | 3860000 | 119.23 | *chr38.171* |
| 1 | 3850001 | 3860000 | 119.23 | *ALG8* |
| 1 | 3860001 | 3870000 | 94.21 | *ALG8* |
| 1 | 3870001 | 3880000 | 302.48 | *ALG8* |
| 1 | 7150001 | 7160000 | 94.43 | *Dlg2* |
| 1 | 7835001 | 7845000 | 90.17 | *PCF11* |
| 1 | 12110001 | 12120000 | 102.67 | *Ccdc82* |
| 1 | 16715001 | 16725000 | 128.59 | *VMO1* |
| 1 | 21270001 | 21280000 | 90.94 | *WASF3* |
| 1 | 21525001 | 21535000 | 150.48 | *Rasl11a* |
| 1 | 21530001 | 21540000 | 138.94 | *Rasl11a* |
| 1 | 25735001 | 25745000 | 103.92 | *ALG5* |
| 1 | 30525001 | 30535000 | 180.17 | *Gtf2f2* |
| 1 | 35590001 | 35600000 | 95.14 | *DDB_G0269086* |
| 1 | 52910001 | 52920000 | 92.93 | *GPC5* |
| 1 | 52915001 | 52925000 | 182.17 | *GPC5* |
| 1 | 52920001 | 52930000 | 129.80 | *GPC5* |
| 1 | 53000001 | 53010000 | 186.50 | *GPC5* |
| 1 | 79335001 | 79345000 | 88.39 | *Gpm6b* |
| 1 | 81300001 | 81310000 | 103.21 | *NHS* |
| 1 | 81320001 | 81330000 | 104.33 | *BEND2* |
| 1 | 86815001 | 86825000 | 109.02 | *Gk* |
| 1 | 90580001 | 90590000 | 113.32 | *Cask* |
| 1 | 90585001 | 90595000 | 97.08 | *Cask* |
| 1 | 90610001 | 90620000 | 96.61 | *Cask* |
| 1 | 90625001 | 90635000 | 95.09 | *Cask* |
| 1 | 90755001 | 90765000 | 106.93 | *Cask* |
| 1 | 92315001 | 92325000 | 95.24 | *TRAPPC10* |
| 1 | 92325001 | 92335000 | 136.91 | *TRAPPC10* |
| 1 | 92460001 | 92470000 | 107.82 | *chr38.1225* |
| 1 | 92985001 | 92995000 | 140.31 | *Pknox1* |
| 1 | 92990001 | 93000000 | 113.23 | *Pknox1* |
| 1 | 94050001 | 94060000 | 144.06 | *DSCAM* |
| 1 | 94055001 | 94065000 | 223.16 | *DSCAM* |
| 1 | 94060001 | 94070000 | 152.26 | *DSCAM* |
| 1 | 95850001 | 95860000 | 113.10 | *HLCS* |
| 1 | 98590001 | 98600000 | 112.80 | *GRIK1* |
| 1 | 98595001 | 98605000 | 89.06 | *GRIK1* |
| 1 | 98605001 | 98615000 | 100.99 | *GRIK1* |
| 1 | 99955001 | 99965000 | 134.19 | *Cyyr1* |
| 1 | 100015001 | 100025000 | 113.83 | *app* |
| 1 | 110120001 | 110130000 | 90.83 | *HGD* |
| 1 | 117725001 | 117735000 | 170.13 | *BBX* |
| 1 | 117730001 | 117740000 | 140.94 | *BBX* |
| 1 | 117735001 | 117745000 | 179.63 | *BBX* |
| 1 | 117740001 | 117750000 | 163.97 | *BBX* |
| 1 | 117745001 | 117755000 | 111.98 | *BBX* |
| 1 | 117750001 | 117760000 | 87.83 | *BBX* |
| 1 | 117755001 | 117765000 | 118.63 | *BBX* |
| 1 | 117965001 | 117975000 | 115.58 | *RCJMB04_1n3* |
| 1 | 117965001 | 117975000 | 115.58 | *SH2D1B* |
| 1 | 117970001 | 117980000 | 108.50 | *SH2D1B* |
| 1 | 117980001 | 117990000 | 130.51 | *SH2D1B* |
| 1 | 117980001 | 117990000 | 130.51 | *chr38.1750* |
| 1 | 117985001 | 117995000 | 151.24 | *chr38.1750* |
| 1 | 118030001 | 118040000 | 113.29 | *TRAT1* |
| 1 | 118035001 | 118045000 | 95.45 | *TRAT1* |
| 1 | 118065001 | 118075000 | 111.34 | *Hjurp* |
| 1 | 118930001 | 118940000 | 208.74 | *PHLDB2* |
| 1 | 119125001 | 119135000 | 128.56 | *CD200* |
| 1 | 123395001 | 123405000 | 88.80 | *POU1F1* |
| 1 | 137215001 | 137225000 | 95.92 | *PPARA* |
| 1 | 137220001 | 137230000 | 108.08 | *PPARA* |
| 1 | 137585001 | 137595000 | 87.87 | *ETV6* |
| 1 | 137595001 | 137605000 | 90.86 | *ETV6* |
| 1 | 137610001 | 137620000 | 86.79 | *ETV6* |
| 1 | 142915001 | 142925000 | 91.48 | *DYRK4* |
| 1 | 142915001 | 142925000 | 91.48 | *PEX26* |
| 1 | 145560001 | 145570000 | 94.75 | *DENND5B* |
| 1 | 148505001 | 148515000 | 111.65 | *KDM7A* |
| 1 | 148510001 | 148520000 | 134.62 | *KDM7A* |
| 1 | 150660001 | 150670000 | 121.69 | *HCFC2* |
| 1 | 150670001 | 150680000 | 114.35 | *HCFC2* |
| 1 | 150690001 | 150700000 | 132.48 | *NFYB* |
| 1 | 150690001 | 150700000 | 132.48 | *chr38.2187* |
| 1 | 150815001 | 150825000 | 96.46 | *CHST11* |
| 1 | 151060001 | 151070000 | 94.59 | *ALDH1L2* |
| 1 | 151065001 | 151075000 | 179.41 | *ALDH1L2* |
| 1 | 151120001 | 151130000 | 88.17 | *WASHC4* |
| 1 | 151190001 | 151200000 | 202.38 | *FGF9* |
| 1 | 151195001 | 151205000 | 184.12 | *FGF9* |
| 1 | 151200001 | 151210000 | 127.40 | *FGF9* |
| 1 | 151760001 | 151770000 | 192.94 | *TMEM263* |
| 1 | 151765001 | 151775000 | 210.08 | *TMEM263* |
| 1 | 151775001 | 151785000 | 267.05 | *TMEM263* |
| 1 | 151775001 | 151785000 | 267.05 | *MTERF2* |
| 1 | 151945001 | 151955000 | 87.34 | *btbd11a* |
| 1 | 151950001 | 151960000 | 201.32 | *btbd11a* |
| 1 | 154875001 | 154885000 | 131.65 | *RPS19BP1* |
| 1 | 163925001 | 163935000 | 107.82 | *ANO4* |
| 1 | 164230001 | 164240000 | 96.89 | *CNOT4* |
| 1 | 164235001 | 164245000 | 126.07 | *CNOT4* |
| 1 | 166240001 | 166250000 | 131.87 | *ZC3H7B* |
| 1 | 167090001 | 167100000 | 85.80 | *Pawr* |
| 1 | 171230001 | 171240000 | 112.70 | *FRS2* |
| 1 | 176235001 | 176245000 | 121.92 | *SCAF11* |
| 1 | 176310001 | 176320000 | 90.77 | *ARID2* |
| 1 | 176315001 | 176325000 | 100.88 | *ARID2* |
| 1 | 177515001 | 177525000 | 205.39 | *PRICKLE1* |
| 1 | 178710001 | 178720000 | 166.62 | *PNPLA8* |
| 1 | 178725001 | 178735000 | 159.85 | *ARSD* |
| 1 | 178745001 | 178755000 | 251.65 | *ARSD* |
| 1 | 178755001 | 178765000 | 371.10 | *ARSD* |
| 1 | 178765001 | 178775000 | 141.07 | *ARSD* |
| 1 | 178775001 | 178785000 | 138.00 | *ARSD* |
| 1 | 178780001 | 178790000 | 177.50 | *ARSD* |
| 1 | 178790001 | 178800000 | 112.54 | *THAP5* |
| 1 | 179820001 | 179830000 | 142.94 | *IMMP2L* |
| 1 | 179835001 | 179845000 | 131.58 | *IMMP2L* |
| 1 | 179860001 | 179870000 | 137.60 | *IMMP2L* |
| 1 | 180555001 | 180565000 | 135.19 | *BMT2* |
| 1 | 184645001 | 184655000 | 127.10 | *FAM3C* |
| 1 | 187000001 | 187010000 | 91.21 | *GRM8* |
| 1 | 187070001 | 187080000 | 127.78 | *GRM8* |
| 1 | 189595001 | 189605000 | 90.11 | *chr38.2802* |
| 1 | 189620001 | 189630000 | 101.43 | *BRD1* |
| 1 | 189625001 | 189635000 | 146.18 | *BRD1* |
| 1 | 189665001 | 189675000 | 173.31 | *chr38.2804* |
| 1 | 192660001 | 192670000 | 259.69 | *CERK* |
| 1 | 192665001 | 192675000 | 445.38 | *CERK* |
| 1 | 192670001 | 192680000 | 309.84 | *CERK* |
| 1 | 192675001 | 192685000 | 252.28 | *CERK* |
| 1 | 194900001 | 194910000 | 102.93 | *ATXN7L1* |
| 1 | 195040001 | 195050000 | 126.97 | *PUS7* |
| 1 | 195055001 | 195065000 | 239.95 | *SRPK2* |
| 1 | 195060001 | 195070000 | 159.16 | *SRPK2* |
| 1 | 195065001 | 195075000 | 101.09 | *SRPK2* |
| 1 | 195070001 | 195080000 | 327.15 | *SRPK2* |
| 1 | 195075001 | 195085000 | 138.35 | *SRPK2* |
| 1 | 195085001 | 195095000 | 361.12 | *SRPK2* |
| 1 | 195090001 | 195100000 | 202.81 | *SRPK2* |
| 1 | 195095001 | 195105000 | 265.00 | *SRPK2* |
| 1 | 195100001 | 195110000 | 245.58 | *SRPK2* |
| 1 | 195105001 | 195115000 | 241.21 | *SRPK2* |
| 1 | 195110001 | 195120000 | 127.44 | *SRPK2* |
| 1 | 195115001 | 195125000 | 250.40 | *SRPK2* |
| 1 | 195120001 | 195130000 | 99.61 | *SRPK2* |
| 1 | 195125001 | 195135000 | 283.71 | *SRPK2* |
| 1 | 195130001 | 195140000 | 291.56 | *SRPK2* |
| 1 | 195135001 | 195145000 | 94.43 | *SRPK2* |
| 1 | 195140001 | 195150000 | 112.65 | *SRPK2* |
| 1 | 195145001 | 195155000 | 257.05 | *SRPK2* |
| 1 | 195150001 | 195160000 | 121.01 | *SRPK2* |
| 1 | 195155001 | 195165000 | 106.93 | *SRPK2* |
| 1 | 195160001 | 195170000 | 139.91 | *SRPK2* |
| 1 | 195165001 | 195175000 | 103.80 | *SRPK2* |
| 1 | 195255001 | 195265000 | 96.79 | *KMT2E* |
| 1 | 202860001 | 202870000 | 111.39 | *Sec61a2* |
| 1 | 202870001 | 202880000 | 233.91 | *Sec61a2* |
| 1 | 202870001 | 202880000 | 233.91 | *Dhtkd1* |
| 1 | 202875001 | 202885000 | 131.69 | *Dhtkd1* |
| 1 | 202875001 | 202885000 | 131.69 | *dhtkd1* |
| 1 | 202880001 | 202890000 | 145.62 | *dhtkd1* |
| 1 | 202885001 | 202895000 | 305.71 | *dhtkd1* |
| 1 | 202890001 | 202900000 | 89.45 | *dhtkd1* |
| 1 | 204815001 | 204825000 | 236.51 | *GATA3* |
| 1 | 204820001 | 204830000 | 253.15 | *GATA3* |
| 1 | 204825001 | 204835000 | 649.39 | *GATA3* |
| 1 | 204830001 | 204840000 | 199.28 | *GATA3* |
| 1 | 204855001 | 204865000 | 110.97 | *TAF3* |
| 1 | 204860001 | 204870000 | 111.39 | *TAF3* |
| 1 | 204865001 | 204875000 | 121.91 | *TAF3* |
| 1 | 204870001 | 204880000 | 314.06 | *TAF3* |
| 1 | 204895001 | 204905000 | 244.17 | *TAF3* |
| 1 | 204900001 | 204910000 | 118.62 | *TAF3* |
| 1 | 204910001 | 204920000 | 190.51 | *TAF3* |
| 1 | 204920001 | 204930000 | 187.64 | *TAF3* |
| 1 | 204925001 | 204935000 | 221.98 | *TAF3* |
| 1 | 204930001 | 204940000 | 129.39 | *TAF3* |
| 1 | 204935001 | 204945000 | 88.61 | *TAF3* |
| 1 | 204940001 | 204950000 | 229.53 | *TAF3* |
| 1 | 204955001 | 204965000 | 90.69 | *TAF3* |
| 1 | 205690001 | 205700000 | 88.81 | *IL2RA* |
| 1 | 205700001 | 205710000 | 273.12 | *IL2RA* |
| 1 | 205705001 | 205715000 | 212.67 | *IL2RA* |
| 1 | 206955001 | 206965000 | 138.54 | *Chchd3* |
| 1 | 206960001 | 206970000 | 300.09 | *Chchd3* |
| 1 | 207135001 | 207145000 | 123.59 | *EXOC4* |
| 1 | 207565001 | 207575000 | 94.14 | *LRGUK* |
| 1 | 208720001 | 208730000 | 111.68 | *GCC1* |
| 1 | 208720001 | 208730000 | 111.68 | *chr38.3093* |
| 2 | 9200001 | 9210000 | 94.47 | *ADCY8* |
| 2 | 11700001 | 11710000 | 116.22 | *MTSS1* |
| 2 | 12295001 | 12305000 | 99.79 | *chr37.200* |
| 2 | 13825001 | 13835000 | 92.13 | *Enpp2* |
| 2 | 13885001 | 13895000 | 93.65 | *chr37.237* |
| 2 | 13885001 | 13895000 | 93.65 | *CCN3* |
| 2 | 14355001 | 14365000 | 124.41 | *EXT1* |
| 2 | 16840001 | 16850000 | 88.27 | *Csmd3* |
| 2 | 16850001 | 16860000 | 114.86 | *Csmd3* |
| 2 | 16880001 | 16890000 | 104.08 | *Csmd3* |
| 2 | 21530001 | 21540000 | 86.24 | *UBR5* |
| 2 | 24955001 | 24965000 | 107.39 | *TMEM67* |
| 2 | 26605001 | 26615000 | 112.42 | *slc7a6* |
| 2 | 26610001 | 26620000 | 180.98 | *slc7a6* |
| 2 | 26620001 | 26630000 | 143.37 | *slc7a6* |
| 2 | 26630001 | 26640000 | 183.58 | *Ripk2* |
| 2 | 26640001 | 26650000 | 317.17 | *Ripk2* |
| 2 | 29875001 | 29885000 | 157.52 | *FABP4* |
| 2 | 31835001 | 31845000 | 94.23 | *Pex2* |
| 2 | 31840001 | 31850000 | 109.55 | *Pex2* |
| 2 | 31855001 | 31865000 | 125.07 | *Pex2* |
| 2 | 33545001 | 33555000 | 87.28 | *Stau2* |
| 2 | 33580001 | 33590000 | 102.36 | *Stau2* |
| 2 | 33585001 | 33595000 | 139.83 | *Stau2* |
| 2 | 33590001 | 33600000 | 95.34 | *Stau2* |
| 2 | 33595001 | 33605000 | 120.85 | *Stau2* |
| 2 | 33600001 | 33610000 | 252.43 | *Stau2* |
| 2 | 33605001 | 33615000 | 200.50 | *Stau2* |
| 2 | 33610001 | 33620000 | 180.85 | *Stau2* |
| 2 | 33615001 | 33625000 | 259.24 | *Stau2* |
| 2 | 33620001 | 33630000 | 154.99 | *Stau2* |
| 2 | 33625001 | 33635000 | 243.10 | *Stau2* |
| 2 | 33630001 | 33640000 | 228.84 | *Stau2* |
| 2 | 33635001 | 33645000 | 187.28 | *Stau2* |
| 2 | 33640001 | 33650000 | 150.15 | *Stau2* |
| 2 | 34090001 | 34100000 | 130.02 | *Kcnb2* |
| 2 | 34250001 | 34260000 | 88.12 | *TRPA1* |
| 2 | 34850001 | 34860000 | 161.53 | *tram1l1* |
| 2 | 34855001 | 34865000 | 94.62 | *tram1l1* |
| 2 | 34965001 | 34975000 | 158.45 | *NCOA2* |
| 2 | 34990001 | 35000000 | 146.58 | *NCOA2* |
| 2 | 34995001 | 35005000 | 131.54 | *NCOA2* |
| 2 | 35000001 | 35010000 | 135.71 | *NCOA2* |
| 2 | 35005001 | 35015000 | 158.12 | *NCOA2* |
| 2 | 35015001 | 35025000 | 145.10 | *NCOA2* |
| 2 | 35025001 | 35035000 | 144.10 | *NCOA2* |
| 2 | 35035001 | 35045000 | 337.96 | *NCOA2* |
| 2 | 35040001 | 35050000 | 221.51 | *NCOA2* |
| 2 | 35045001 | 35055000 | 195.46 | *NCOA2* |
| 2 | 35050001 | 35060000 | 123.90 | *NCOA2* |
| 2 | 35055001 | 35065000 | 184.24 | *NCOA2* |
| 2 | 35060001 | 35070000 | 157.34 | *NCOA2* |
| 2 | 35065001 | 35075000 | 134.76 | *NCOA2* |
| 2 | 35070001 | 35080000 | 170.96 | *NCOA2* |
| 2 | 35075001 | 35085000 | 279.42 | *NCOA2* |
| 2 | 35080001 | 35090000 | 287.58 | *NCOA2* |
| 2 | 35085001 | 35095000 | 178.44 | *NCOA2* |
| 2 | 35090001 | 35100000 | 253.25 | *NCOA2* |
| 2 | 35095001 | 35105000 | 169.67 | *NCOA2* |
| 2 | 35100001 | 35110000 | 128.09 | *NCOA2* |
| 2 | 35100001 | 35110000 | 128.09 | *PRDM14* |
| 2 | 35105001 | 35115000 | 329.90 | *PRDM14* |
| 2 | 35110001 | 35120000 | 205.51 | *PRDM14* |
| 2 | 35180001 | 35190000 | 109.37 | *SLCO5A1* |
| 2 | 35865001 | 35875000 | 90.83 | *PREX2* |
| 2 | 36275001 | 36285000 | 86.48 | *ARFGEF1* |
| 2 | 36275001 | 36285000 | 86.48 | *CSPP1* |
| 2 | 39740001 | 39750000 | 182.58 | *NSMAF* |
| 2 | 39745001 | 39755000 | 210.80 | *NSMAF* |
| 2 | 39750001 | 39760000 | 174.93 | *NSMAF* |
| 2 | 39755001 | 39765000 | 385.42 | *NSMAF* |
| 2 | 39760001 | 39770000 | 358.90 | *NSMAF* |
| 2 | 39765001 | 39775000 | 287.39 | *NSMAF* |
| 2 | 39770001 | 39780000 | 124.22 | *NSMAF* |
| 2 | 39775001 | 39785000 | 196.35 | *NSMAF* |
| 2 | 39780001 | 39790000 | 115.19 | *NSMAF* |
| 2 | 39775001 | 39785000 | 196.35 | *SDCBP* |
| 2 | 39780001 | 39790000 | 115.19 | *SDCBP* |
| 2 | 39785001 | 39795000 | 259.21 | *SDCBP* |
| 2 | 42470001 | 42480000 | 99.36 | *ST18* |
| 2 | 54245001 | 54255000 | 132.51 | *TWSG1* |
| 2 | 54255001 | 54265000 | 95.01 | *TWSG1* |
| 2 | 56200001 | 56210000 | 104.91 | *LDLRAD4* |
| 2 | 56345001 | 56355000 | 101.18 | *FAM210A* |
| 2 | 56390001 | 56400000 | 94.25 | *RNMT* |
| 2 | 56435001 | 56445000 | 140.89 | *MC2R* |
| 2 | 56440001 | 56450000 | 240.68 | *MC2R* |
| 2 | 56470001 | 56480000 | 108.00 | *MC2R* |
| 2 | 59310001 | 59320000 | 86.83 | *DOK6* |
| 2 | 59315001 | 59325000 | 95.70 | *DOK6* |
| 2 | 59330001 | 59340000 | 103.43 | *DOK6* |
| 2 | 61550001 | 61560000 | 92.52 | *FBXO15* |
| 2 | 61625001 | 61635000 | 86.68 | *C18orf63* |
| 2 | 61630001 | 61640000 | 87.71 | *C18orf63* |
| 2 | 61630001 | 61640000 | 87.71 | *C18orf63* |
| 2 | 61690001 | 61700000 | 207.78 | *CNDP2* |
| 2 | 61690001 | 61700000 | 207.78 | *chr37.774* |
| 2 | 61755001 | 61765000 | 86.85 | *ZNF407* |
| 2 | 63680001 | 63690000 | 115.45 | *CARMIL1* |
| 2 | 63840001 | 63850000 | 167.21 | *Bloc1s4* |
| 2 | 68380001 | 68390000 | 89.98 | *BAG1* |
| 2 | 68520001 | 68530000 | 190.09 | *PCBP3* |
| 2 | 68525001 | 68535000 | 188.25 | *PCBP3* |
| 2 | 69160001 | 69170000 | 130.77 | *MOCOS* |
| 2 | 69635001 | 69645000 | 105.09 | *Epb41l4b* |
| 2 | 73340001 | 73350000 | 118.91 | *COBL* |
| 2 | 73420001 | 73430000 | 145.14 | *Cobl* |
| 2 | 73425001 | 73435000 | 165.42 | *Cobl* |
| 2 | 73430001 | 73440000 | 113.41 | *Cobl* |
| 2 | 73440001 | 73450000 | 112.66 | *Cobl* |
| 2 | 73445001 | 73455000 | 110.22 | *Cobl* |
| 2 | 74535001 | 74545000 | 86.53 | *ABCA13* |
| 2 | 81225001 | 81235000 | 118.71 | *PIGN* |
| 2 | 81335001 | 81345000 | 242.58 | *RNF152* |
| 2 | 81985001 | 81995000 | 179.09 | *DROSHA* |
| 2 | 85240001 | 85250000 | 90.46 | *CDH10* |
| 2 | 85275001 | 85285000 | 88.86 | *CDH10* |
| 2 | 109655001 | 109665000 | 101.10 | *Cdkal1* |
| 2 | 109670001 | 109680000 | 186.32 | *Cdkal1* |
| 2 | 109675001 | 109685000 | 142.52 | *Cdkal1* |
| 2 | 109680001 | 109690000 | 165.32 | *Cdkal1* |
| 2 | 109685001 | 109695000 | 159.45 | *Cdkal1* |
| 2 | 109690001 | 109700000 | 129.31 | *Cdkal1* |
| 2 | 109700001 | 109710000 | 130.14 | *Cdkal1* |
| 2 | 109705001 | 109715000 | 89.45 | *Cdkal1* |
| 2 | 109705001 | 109715000 | 89.45 | *CDKAL1* |
| 2 | 109720001 | 109730000 | 144.90 | *CDKAL1* |
| 2 | 109735001 | 109745000 | 153.28 | *CDKAL1* |
| 2 | 109740001 | 109750000 | 184.68 | *CDKAL1* |
| 2 | 109745001 | 109755000 | 149.71 | *CDKAL1* |
| 2 | 109750001 | 109760000 | 187.24 | *CDKAL1* |
| 2 | 109755001 | 109765000 | 134.41 | *CDKAL1* |
| 2 | 109765001 | 109775000 | 125.27 | *CDKAL1* |
| 2 | 109790001 | 109800000 | 102.34 | *CDKAL1* |
| 2 | 109805001 | 109815000 | 123.45 | *CDKAL1* |
| 2 | 109825001 | 109835000 | 89.85 | *CDKAL1* |
| 2 | 109835001 | 109845000 | 127.01 | *CDKAL1* |
| 2 | 109840001 | 109850000 | 132.35 | *CDKAL1* |
| 2 | 109855001 | 109865000 | 116.48 | *CDKAL1* |
| 2 | 111025001 | 111035000 | 114.80 | *DCDC2* |
| 2 | 112385001 | 112395000 | 200.98 | *Nfatc1* |
| 2 | 138340001 | 138350000 | 88.31 | *FAM171A1* |
| 2 | 139225001 | 139235000 | 122.58 | *HACD1* |
| 2 | 142355001 | 142365000 | 124.15 | *ARHGAP21* |
| 2 | 142365001 | 142375000 | 115.53 | *ARHGAP21* |
| 2 | 142405001 | 142415000 | 123.97 | *ARHGAP21* |
| 2 | 142410001 | 142420000 | 145.41 | *ARHGAP21* |
| 2 | 142415001 | 142425000 | 93.57 | *ARHGAP21* |
| 2 | 142420001 | 142430000 | 95.10 | *ARHGAP21* |
| 2 | 146525001 | 146535000 | 153.75 | *CREM* |
| 2 | 146530001 | 146540000 | 95.52 | *CREM* |
| 2 | 151385001 | 151395000 | 111.80 | *UBE3C* |
| 2 | 152410001 | 152420000 | 89.58 | *PAXIP1* |
| 2 | 152555001 | 152565000 | 95.66 | *DPP6* |
| 2 | 152565001 | 152575000 | 96.13 | *DPP6* |
| 2 | 152575001 | 152585000 | 144.45 | *DPP6* |
| 2 | 152580001 | 152590000 | 113.61 | *DPP6* |
| 2 | 152600001 | 152610000 | 202.40 | *DPP6* |
| 2 | 152605001 | 152615000 | 129.09 | *DPP6* |
| 2 | 152615001 | 152625000 | 100.48 | *DPP6* |
| 2 | 156170001 | 156180000 | 85.53 | *crhr2* |
| 2 | 156175001 | 156185000 | 85.38 | *crhr2* |
| 2 | 156240001 | 156250000 | 96.59 | *crhr2* |
| 3 | 1380001 | 1390000 | 128.61 | *Otof* |
| 3 | 1400001 | 1410000 | 280.60 | *Otof* |
| 3 | 1540001 | 1550000 | 94.42 | *DNAJC27* |
| 3 | 1540001 | 1550000 | 94.42 | *Adcy3* |
| 3 | 3520001 | 3530000 | 127.65 | *GATA4* |
| 3 | 4090001 | 4100000 | 223.64 | *PKHD1* |
| 3 | 4095001 | 4105000 | 181.11 | *PKHD1* |
| 3 | 5830001 | 5840000 | 91.78 | *RUNX2* |
| 3 | 6100001 | 6110000 | 105.01 | *CLIC5* |
| 3 | 7325001 | 7335000 | 124.88 | *EVA1C* |
| 3 | 7355001 | 7365000 | 125.25 | *EVA1C* |
| 3 | 7365001 | 7375000 | 111.70 | *EVA1C* |
| 3 | 7375001 | 7385000 | 95.04 | *EVA1C* |
| 3 | 7420001 | 7430000 | 114.39 | *GCFC2* |
| 3 | 26280001 | 26290000 | 94.73 | *COL21A1* |
| 3 | 35445001 | 35455000 | 201.69 | *BCKDHB* |
| 3 | 35450001 | 35460000 | 473.72 | *BCKDHB* |
| 3 | 35455001 | 35465000 | 182.23 | *BCKDHB* |
| 3 | 35460001 | 35470000 | 132.32 | *BCKDHB* |
| 3 | 35465001 | 35475000 | 145.93 | *BCKDHB* |
| 3 | 35525001 | 35535000 | 103.43 | *BCKDHB* |
| 3 | 38255001 | 38265000 | 88.92 | *ZNF292* |
| 3 | 38260001 | 38270000 | 107.02 | *ZNF292* |
| 3 | 38275001 | 38285000 | 95.51 | *ZNF292* |
| 3 | 38295001 | 38305000 | 119.35 | *ZNF292* |
| 3 | 38315001 | 38325000 | 151.87 | *ZNF292* |
| 3 | 38315001 | 38325000 | 151.87 | *GJB7* |
| 3 | 38325001 | 38335000 | 175.31 | *SMIM8* |
| 3 | 38340001 | 38350000 | 116.49 | *QtsA-12155* |
| 3 | 43615001 | 43625000 | 369.98 | *FBXL4* |
| 3 | 43765001 | 43775000 | 249.44 | *FAXC* |
| 3 | 43770001 | 43780000 | 169.72 | *FAXC* |
| 3 | 43775001 | 43785000 | 89.97 | *FAXC* |
| 3 | 46970001 | 46980000 | 160.83 | *PREP* |
| 3 | 48325001 | 48335000 | 88.93 | *NR2E1* |
| 3 | 48330001 | 48340000 | 119.56 | *NR2E1* |
| 3 | 48390001 | 48400000 | 113.30 | *AFG1L* |
| 3 | 48395001 | 48405000 | 94.99 | *AFG1L* |
| 3 | 48400001 | 48410000 | 123.66 | *AFG1L* |
| 3 | 48410001 | 48420000 | 134.62 | *AFG1L* |
| 3 | 48415001 | 48425000 | 136.14 | *AFG1L* |
| 3 | 48425001 | 48435000 | 253.18 | *AFG1L* |
| 3 | 48430001 | 48440000 | 278.32 | *AFG1L* |
| 3 | 48435001 | 48445000 | 222.53 | *AFG1L* |
| 3 | 48440001 | 48450000 | 167.31 | *AFG1L* |
| 3 | 48445001 | 48455000 | 325.97 | *AFG1L* |
| 3 | 48450001 | 48460000 | 242.08 | *AFG1L* |
| 3 | 58365001 | 58375000 | 91.93 | *LAMA2* |
| 3 | 58370001 | 58380000 | 95.62 | *LAMA2* |
| 3 | 60855001 | 60865000 | 134.23 | *ALDH8A1* |
| 3 | 60855001 | 60865000 | 134.23 | *HBS1L* |
| 3 | 65445001 | 65455000 | 91.03 | *ZDHHC14* |
| 3 | 65480001 | 65490000 | 209.56 | *TMEM242* |
| 3 | 65485001 | 65495000 | 255.52 | *TMEM242* |
| 3 | 66875001 | 66885000 | 97.18 | *CNKSR3* |
| 3 | 67520001 | 67530000 | 291.68 | *RGS17* |
| 3 | 67550001 | 67560000 | 90.29 | *FBXO5* |
| 3 | 67555001 | 67565000 | 113.00 | *FBXO5* |
| 3 | 67675001 | 67685000 | 203.25 | *VIP* |
| 3 | 67685001 | 67695000 | 106.25 | *VIP* |
| 3 | 67700001 | 67710000 | 280.36 | *myct1* |
| 3 | 67710001 | 67720000 | 102.17 | *myct1* |
| 3 | 67715001 | 67725000 | 144.26 | *myct1* |
| 3 | 67720001 | 67730000 | 164.50 | *myct1* |
| 3 | 67725001 | 67735000 | 263.22 | *myct1* |
| 3 | 67730001 | 67740000 | 302.16 | *myct1* |
| 3 | 67750001 | 67760000 | 101.93 | *SYNE1* |
| 3 | 67760001 | 67770000 | 130.09 | *SYNE1* |
| 3 | 67765001 | 67775000 | 120.22 | *SYNE1* |
| 3 | 67770001 | 67780000 | 146.48 | *SYNE1* |
| 3 | 67780001 | 67790000 | 155.76 | *SYNE1* |
| 3 | 67785001 | 67795000 | 155.44 | *SYNE1* |
| 3 | 67795001 | 67805000 | 112.38 | *SYNE1* |
| 3 | 67815001 | 67825000 | 156.79 | *SYNE1* |
| 3 | 67820001 | 67830000 | 147.40 | *SYNE1* |
| 3 | 67825001 | 67835000 | 112.59 | *SYNE1* |
| 3 | 67830001 | 67840000 | 108.86 | *SYNE1* |
| 3 | 67835001 | 67845000 | 207.81 | *SYNE1* |
| 3 | 67840001 | 67850000 | 221.47 | *SYNE1* |
| 3 | 67845001 | 67855000 | 88.68 | *SYNE1* |
| 3 | 67840001 | 67850000 | 221.47 | *SYNE1* |
| 3 | 67845001 | 67855000 | 88.68 | *SYNE1* |
| 3 | 67850001 | 67860000 | 255.02 | *SYNE1* |
| 3 | 67870001 | 67880000 | 114.79 | *SYNE1* |
| 3 | 67875001 | 67885000 | 129.63 | *SYNE1* |
| 3 | 67870001 | 67880000 | 114.79 | *SYNE1* |
| 3 | 67875001 | 67885000 | 129.63 | *SYNE1* |
| 3 | 67880001 | 67890000 | 195.12 | *SYNE1* |
| 3 | 67880001 | 67890000 | 195.12 | *SYNE1* |
| 3 | 67885001 | 67895000 | 219.85 | *SYNE1* |
| 3 | 67895001 | 67905000 | 167.48 | *SYNE1* |
| 3 | 67900001 | 67910000 | 108.94 | *SYNE1* |
| 3 | 67905001 | 67915000 | 91.41 | *SYNE1* |
| 3 | 67910001 | 67920000 | 105.23 | *SYNE1* |
| 3 | 67920001 | 67930000 | 112.04 | *SYNE1* |
| 3 | 67925001 | 67935000 | 106.04 | *SYNE1* |
| 3 | 67920001 | 67930000 | 112.04 | *SYNE1* |
| 3 | 67925001 | 67935000 | 106.04 | *SYNE1* |
| 3 | 67930001 | 67940000 | 117.71 | *SYNE1* |
| 3 | 67935001 | 67945000 | 105.75 | *SYNE1* |
| 3 | 67945001 | 67955000 | 85.40 | *SYNE1* |
| 3 | 67955001 | 67965000 | 169.83 | *SYNE1* |
| 3 | 67960001 | 67970000 | 106.67 | *SYNE1* |
| 3 | 67965001 | 67975000 | 104.97 | *SYNE1* |
| 3 | 67970001 | 67980000 | 115.99 | *SYNE1* |
| 3 | 67965001 | 67975000 | 104.97 | *SYNE1* |
| 3 | 67970001 | 67980000 | 115.99 | *SYNE1* |
| 3 | 67980001 | 67990000 | 125.72 | *SYNE1* |
| 3 | 67990001 | 68000000 | 91.01 | *SYNE1* |
| 3 | 68005001 | 68015000 | 87.56 | *SYNE1* |
| 3 | 68020001 | 68030000 | 198.53 | *SYNE1* |
| 3 | 68025001 | 68035000 | 184.10 | *SYNE1* |
| 3 | 68025001 | 68035000 | 184.10 | *SYNE1* |
| 3 | 68030001 | 68040000 | 116.11 | *SYNE1* |
| 3 | 68045001 | 68055000 | 95.40 | *SYNE1* |
| 3 | 68050001 | 68060000 | 117.72 | *ESR1* |
| 3 | 68060001 | 68070000 | 126.48 | *ESR1* |
| 3 | 68065001 | 68075000 | 168.57 | *ESR1* |
| 3 | 68070001 | 68080000 | 100.90 | *ESR1* |
| 3 | 68075001 | 68085000 | 162.51 | *ESR1* |
| 3 | 68085001 | 68095000 | 184.77 | *ESR1* |
| 3 | 68090001 | 68100000 | 121.25 | *ESR1* |
| 3 | 68095001 | 68105000 | 130.97 | *ESR1* |
| 3 | 68100001 | 68110000 | 109.65 | *ESR1* |
| 3 | 68115001 | 68125000 | 90.30 | *ESR1* |
| 3 | 68120001 | 68130000 | 110.44 | *ESR1* |
| 3 | 68125001 | 68135000 | 93.93 | *ESR1* |
| 3 | 68130001 | 68140000 | 90.63 | *ESR1* |
| 3 | 68135001 | 68145000 | 95.98 | *ESR1* |
| 3 | 68145001 | 68155000 | 90.07 | *ESR1* |
| 3 | 68155001 | 68165000 | 199.20 | *ESR1* |
| 3 | 68160001 | 68170000 | 180.63 | *ESR1* |
| 3 | 68165001 | 68175000 | 189.73 | *ESR1* |
| 3 | 68175001 | 68185000 | 135.27 | *ESR1* |
| 3 | 68180001 | 68190000 | 125.23 | *ESR1* |
| 3 | 68195001 | 68205000 | 130.84 | *ESR1* |
| 3 | 68270001 | 68280000 | 120.84 | *CCDC170* |
| 3 | 70440001 | 70450000 | 91.88 | *ADGB* |
| 3 | 71595001 | 71605000 | 183.83 | *FNDC1* |
| 3 | 71600001 | 71610000 | 148.81 | *FNDC1* |
| 3 | 71605001 | 71615000 | 131.10 | *FNDC1* |
| 3 | 77120001 | 77130000 | 210.92 | *FAM120B* |
| 3 | 77130001 | 77140000 | 143.06 | *FAM120B* |
| 3 | 77135001 | 77145000 | 291.34 | *FAM120B* |
| 3 | 77140001 | 77150000 | 127.95 | *FAM120B* |
| 3 | 77145001 | 77155000 | 110.59 | *FAM120B* |
| 3 | 77215001 | 77225000 | 95.57 | *Psmb1* |
| 3 | 77370001 | 77380000 | 90.08 | *TTC13* |
| 3 | 80125001 | 80135000 | 110.94 | *LYST* |
| 3 | 80130001 | 80140000 | 109.41 | *LYST* |
| 3 | 80160001 | 80170000 | 90.70 | *LYST* |
| 3 | 81995001 | 82005000 | 91.45 | *FMN2* |
| 3 | 82000001 | 82010000 | 90.79 | *FMN2* |
| 3 | 82115001 | 82125000 | 85.97 | *FMN2* |
| 3 | 82120001 | 82130000 | 85.54 | *FMN2* |
| 3 | 82125001 | 82135000 | 89.56 | *FMN2* |
| 3 | 82125001 | 82135000 | 89.56 | *GREM2* |
| 3 | 82140001 | 82150000 | 126.91 | *GREM2* |
| 3 | 82145001 | 82155000 | 130.33 | *GREM2* |
| 3 | 82165001 | 82175000 | 105.93 | *GREM2* |
| 3 | 82195001 | 82205000 | 113.85 | *Rgs7* |
| 3 | 82200001 | 82210000 | 122.97 | *Rgs7* |
| 3 | 82205001 | 82215000 | 114.27 | *Rgs7* |
| 3 | 82215001 | 82225000 | 123.86 | *Rgs7* |
| 3 | 82220001 | 82230000 | 106.80 | *Rgs7* |
| 3 | 82225001 | 82235000 | 155.30 | *Rgs7* |
| 3 | 82230001 | 82240000 | 120.80 | *Rgs7* |
| 3 | 84040001 | 84050000 | 341.04 | *KIF26B* |
| 3 | 84280001 | 84290000 | 119.67 | *SMYD3* |
| 3 | 84300001 | 84310000 | 146.54 | *SMYD3* |
| 3 | 84305001 | 84315000 | 125.28 | *SMYD3* |
| 3 | 84310001 | 84320000 | 103.39 | *SMYD3* |
| 3 | 84460001 | 84470000 | 128.41 | *SMYD3* |
| 3 | 86365001 | 86375000 | 89.79 | *Fez2* |
| 3 | 86370001 | 86380000 | 119.61 | *Fez2* |
| 3 | 86560001 | 86570000 | 175.40 | *STRN* |
| 3 | 86565001 | 86575000 | 177.99 | *STRN* |
| 3 | 92295001 | 92305000 | 105.99 | *Pigf* |
| 3 | 102490001 | 102500000 | 136.36 | *ANGEL2* |
| 3 | 102715001 | 102725000 | 102.30 | *Tmem63a* |
| 3 | 102950001 | 102960000 | 99.70 | *si:dkeyp-59a8.4* |
| 3 | 105425001 | 105435000 | 94.55 | *NRXN1* |
| 3 | 105430001 | 105440000 | 99.90 | *NRXN1* |
| 3 | 117265001 | 117275000 | 102.87 | *Snap25* |
| 3 | 118510001 | 118520000 | 146.46 | *Macrod2* |
| 3 | 118515001 | 118525000 | 186.96 | *Macrod2* |
| 3 | 118535001 | 118545000 | 112.41 | *Macrod2* |
| 3 | 118545001 | 118555000 | 136.09 | *Macrod2* |
| 3 | 118510001 | 118520000 | 146.46 | *Macrod2* |
| 3 | 118515001 | 118525000 | 186.96 | *Macrod2* |
| 3 | 118535001 | 118545000 | 112.41 | *Macrod2* |
| 3 | 118545001 | 118555000 | 136.09 | *Macrod2* |
| 4 | 455001 | 465000 | 93.95 | *rnf145* |
| 4 | 455001 | 465000 | 93.95 | *rnf145* |
| 4 | 1370001 | 1380000 | 155.59 | *Exoc6b* |
| 4 | 1375001 | 1385000 | 240.27 | *Exoc6b* |
| 4 | 1380001 | 1390000 | 122.97 | *Exoc6b* |
| 4 | 1385001 | 1395000 | 193.45 | *Exoc6b* |
| 4 | 1490001 | 1500000 | 104.03 | *EXOC6B* |
| 4 | 1495001 | 1505000 | 114.26 | *EXOC6B* |
| 4 | 1510001 | 1520000 | 109.75 | *EXOC6B* |
| 4 | 1520001 | 1530000 | 120.37 | *EXOC6B* |
| 4 | 2920001 | 2930000 | 97.41 | *LZTS3* |
| 4 | 8965001 | 8975000 | 141.54 | *POLN* |
| 4 | 9305001 | 9315000 | 134.31 | *Rnf4* |
| 4 | 11380001 | 11390000 | 86.79 | *ABLIM2* |
| 4 | 13190001 | 13200000 | 96.51 | *STK32B* |
| 4 | 13200001 | 13210000 | 117.64 | *STK32B* |
| 4 | 13690001 | 13700000 | 101.78 | *ZBTB49* |
| 4 | 13690001 | 13700000 | 101.78 | *Lyar* |
| 4 | 13710001 | 13720000 | 126.12 | *TMEM128* |
| 4 | 13785001 | 13795000 | 167.42 | *SLC2A9* |
| 4 | 13790001 | 13800000 | 101.53 | *SLC2A9* |
| 4 | 13805001 | 13815000 | 158.86 | *SLC2A9* |
| 4 | 13860001 | 13870000 | 128.73 | *SLC2A9* |
| 4 | 15695001 | 15705000 | 125.36 | *CC2D2A* |
| 4 | 15740001 | 15750000 | 90.47 | *FBXL5* |
| 4 | 15760001 | 15770000 | 96.13 | *FBXL5* |
| 4 | 15760001 | 15770000 | 96.13 | *BST1* |
| 4 | 15785001 | 15795000 | 148.75 | *BST1* |
| 4 | 15785001 | 15795000 | 148.75 | *chr35.304* |
| 4 | 15810001 | 15820000 | 141.36 | *Cd38* |
| 4 | 15820001 | 15830000 | 197.14 | *Cd38* |
| 4 | 15870001 | 15880000 | 94.64 | *prom1a* |
| 4 | 15875001 | 15885000 | 111.11 | *prom1a* |
| 4 | 15880001 | 15890000 | 118.26 | *prom1a* |
| 4 | 15890001 | 15900000 | 94.01 | *prom1a* |
| 4 | 15900001 | 15910000 | 142.23 | *prom1a* |
| 4 | 15960001 | 15970000 | 168.57 | *TAPT1* |
| 4 | 15970001 | 15980000 | 169.69 | *TAPT1* |
| 4 | 16060001 | 16070000 | 168.66 | *LDB2* |
| 4 | 16065001 | 16075000 | 87.90 | *LDB2* |
| 4 | 27490001 | 27500000 | 96.22 | *FRYL* |
| 4 | 27655001 | 27665000 | 187.80 | *CWH43* |
| 4 | 27705001 | 27715000 | 93.10 | *CWH43* |
| 4 | 28005001 | 28015000 | 122.06 | *SCFD2* |
| 4 | 28035001 | 28045000 | 92.06 | *SCFD2* |
| 4 | 28065001 | 28075000 | 123.54 | *SCFD2* |
| 4 | 28070001 | 28080000 | 122.13 | *SCFD2* |
| 4 | 28115001 | 28125000 | 86.23 | *SCFD2* |
| 4 | 28120001 | 28130000 | 179.35 | *SCFD2* |
| 4 | 28135001 | 28145000 | 123.33 | *SCFD2* |
| 4 | 28145001 | 28155000 | 168.44 | *SCFD2* |
| 4 | 28155001 | 28165000 | 130.59 | *SCFD2* |
| 4 | 28155001 | 28165000 | 130.59 | *chr35.460* |
| 4 | 28165001 | 28175000 | 96.46 | *chr35.460* |
| 4 | 28165001 | 28175000 | 96.46 | *FIP1L1* |
| 4 | 28180001 | 28190000 | 117.43 | *FIP1L1* |
| 4 | 34400001 | 34410000 | 102.09 | *Spata5* |
| 4 | 34935001 | 34945000 | 206.17 | *ANKRD50* |
| 4 | 34940001 | 34950000 | 150.76 | *ANKRD50* |
| 4 | 34935001 | 34945000 | 206.17 | *ANKRD50* |
| 4 | 34940001 | 34950000 | 150.76 | *ANKRD50* |
| 4 | 34945001 | 34955000 | 201.29 | *ANKRD50* |
| 4 | 34940001 | 34950000 | 150.76 | *ANKRD50* |
| 4 | 34945001 | 34955000 | 201.29 | *ANKRD50* |
| 4 | 34955001 | 34965000 | 213.04 | *ANKRD50* |
| 4 | 34970001 | 34980000 | 163.20 | *ANKRD50* |
| 4 | 35250001 | 35260000 | 133.75 | *FAT4* |
| 4 | 45295001 | 45305000 | 124.70 | *Fbxw7* |
| 4 | 53740001 | 53750000 | 99.85 | *GPM6A* |
| 4 | 67070001 | 67080000 | 178.11 | *CCSER1* |
| 4 | 67160001 | 67170000 | 109.53 | *CCSER1* |
| 4 | 67200001 | 67210000 | 159.28 | *CCSER1* |
| 4 | 67250001 | 67260000 | 134.30 | *CCSER1* |
| 4 | 67260001 | 67270000 | 154.69 | *CCSER1* |
| 4 | 67270001 | 67280000 | 129.14 | *CCSER1* |
| 4 | 67295001 | 67305000 | 116.83 | *CCSER1* |
| 4 | 69095001 | 69105000 | 162.72 | *BMPR1B* |
| 4 | 69110001 | 69120000 | 122.05 | *BMPR1B* |
| 4 | 69120001 | 69130000 | 92.33 | *BMPR1B* |
| 4 | 69125001 | 69135000 | 105.67 | *BMPR1B* |
| 5 | 2785001 | 2795000 | 86.16 | *L2HGDH* |
| 5 | 2785001 | 2795000 | 86.16 | *DMAC2L* |
| 5 | 3330001 | 3340000 | 153.12 | *Psmc6* |
| 5 | 7705001 | 7715000 | 108.45 | *ESR2* |
| 5 | 8310001 | 8320000 | 117.11 | *chr34.184* |
| 5 | 9065001 | 9075000 | 92.56 | *Cep170b* |
| 5 | 9070001 | 9080000 | 108.90 | *Cep170b* |
| 5 | 9070001 | 9080000 | 108.90 | *Cep170b* |
| 5 | 9070001 | 9080000 | 108.90 | *cep170b* |
| 5 | 9300001 | 9310000 | 110.83 | *adssl1-a* |
| 5 | 9305001 | 9315000 | 89.02 | *adssl1-a* |
| 5 | 13095001 | 13105000 | 169.26 | *ccdc85c* |
| 5 | 13100001 | 13110000 | 230.48 | *ccdc85c* |
| 5 | 13105001 | 13115000 | 183.70 | *ccdc85c* |
| 5 | 13110001 | 13120000 | 164.28 | *ccdc85c* |
| 5 | 13225001 | 13235000 | 150.62 | *SETD3* |
| 5 | 17745001 | 17755000 | 102.27 | *NRDE2* |
| 5 | 17860001 | 17870000 | 155.36 | *KCNK13* |
| 5 | 17865001 | 17875000 | 148.42 | *KCNK13* |
| 5 | 17885001 | 17895000 | 99.59 | *KCNK13* |
| 5 | 17890001 | 17900000 | 137.76 | *KCNK13* |
| 5 | 18670001 | 18680000 | 167.08 | *SPATA7* |
| 5 | 21290001 | 21300000 | 85.45 | *chr34.380* |
| 5 | 22410001 | 22420000 | 89.45 | *FBLN5* |
| 5 | 30685001 | 30695000 | 104.28 | *Ndufv1* |
| 5 | 30685001 | 30695000 | 104.28 | *Cabp2* |
| 5 | 30690001 | 30700000 | 145.53 | *Cabp2* |
| 5 | 30685001 | 30695000 | 104.28 | *Cdk2ap2* |
| 5 | 30690001 | 30700000 | 145.53 | *Cdk2ap2* |
| 5 | 30685001 | 30695000 | 104.28 | *PITPNM1* |
| 5 | 30690001 | 30700000 | 145.53 | *PITPNM1* |
| 5 | 30970001 | 30980000 | 87.11 | *SCT* |
| 5 | 30985001 | 30995000 | 124.18 | *CDHR5* |
| 5 | 31005001 | 31015000 | 134.39 | *BUB1B* |
| 5 | 31395001 | 31405000 | 130.23 | *Ccdc32* |
| 5 | 31415001 | 31425000 | 233.54 | *Rpusd2* |
| 5 | 31415001 | 31425000 | 233.54 | *KNL1* |
| 5 | 31420001 | 31430000 | 128.66 | *KNL1* |
| 5 | 31430001 | 31440000 | 114.87 | *KNL1* |
| 5 | 31435001 | 31445000 | 288.69 | *KNL1* |
| 5 | 31440001 | 31450000 | 302.81 | *KNL1* |
| 5 | 31445001 | 31455000 | 284.64 | *KNL1* |
| 5 | 31445001 | 31455000 | 284.64 | *RAD51A* |
| 5 | 31450001 | 31460000 | 396.47 | *RAD51A* |
| 5 | 31455001 | 31465000 | 294.88 | *RAD51A* |
| 5 | 31460001 | 31470000 | 683.32 | *RAD51A* |
| 5 | 31465001 | 31475000 | 154.64 | *RAD51A* |
| 5 | 31465001 | 31475000 | 154.64 | *CYP1B1* |
| 5 | 31470001 | 31480000 | 182.34 | *CYP1B1* |
| 5 | 31475001 | 31485000 | 208.31 | *CYP1B1* |
| 5 | 31475001 | 31485000 | 208.31 | *RMDN3* |
| 5 | 31480001 | 31490000 | 174.64 | *RMDN3* |
| 5 | 31485001 | 31495000 | 149.16 | *RMDN3* |
| 5 | 31495001 | 31505000 | 191.37 | *RMDN3* |
| 5 | 31505001 | 31515000 | 154.76 | *RMDN3* |
| 5 | 31510001 | 31520000 | 160.90 | *RMDN3* |
| 5 | 31515001 | 31525000 | 137.40 | *RMDN3* |
| 5 | 32130001 | 32140000 | 125.69 | *CKAP5* |
| 5 | 32140001 | 32150000 | 86.55 | *CKAP5* |
| 5 | 32195001 | 32205000 | 92.66 | *F2* |
| 5 | 32235001 | 32245000 | 118.62 | *ATG13* |
| 5 | 32235001 | 32245000 | 118.62 | *ATG13* |
| 5 | 32250001 | 32260000 | 93.31 | *chr34.752* |
| 5 | 32250001 | 32260000 | 93.31 | *chr34.753* |
| 5 | 32250001 | 32260000 | 93.31 | *AMBRA1* |
| 5 | 32295001 | 32305000 | 105.31 | *AMBRA1* |
| 5 | 32300001 | 32310000 | 115.11 | *AMBRA1* |
| 5 | 32310001 | 32320000 | 176.22 | *AMBRA1* |
| 5 | 34915001 | 34925000 | 105.28 | *Ttbk2* |
| 5 | 34970001 | 34980000 | 126.00 | *UBR1* |
| 5 | 35170001 | 35180000 | 108.58 | *Acot3* |
| 5 | 35170001 | 35180000 | 108.58 | *ACOT1* |
| 5 | 35175001 | 35185000 | 86.33 | *ACOT1* |
| 5 | 40755001 | 40765000 | 213.75 | *MEIS2* |
| 5 | 44430001 | 44440000 | 105.48 | *HEATR5A* |
| 5 | 44435001 | 44445000 | 159.78 | *HEATR5A* |
| 5 | 44440001 | 44450000 | 87.62 | *HEATR5A* |
| 5 | 44455001 | 44465000 | 97.98 | *HEATR5A* |
| 5 | 48385001 | 48395000 | 99.36 | *SHANK2* |
| 5 | 52885001 | 52895000 | 166.29 | *CD82* |
| 5 | 52890001 | 52900000 | 138.48 | *CD82* |
| 5 | 52895001 | 52905000 | 315.27 | *CD82* |
| 5 | 52900001 | 52910000 | 164.00 | *CD82* |
| 5 | 52905001 | 52915000 | 404.31 | *CD82* |
| 5 | 53850001 | 53860000 | 127.54 | *Psmc3* |
| 5 | 53850001 | 53860000 | 127.54 | *tmem178b* |
| 5 | 56425001 | 56435000 | 138.84 | *LUZP2* |
| 5 | 56430001 | 56440000 | 135.35 | *LUZP2* |
| 5 | 56435001 | 56445000 | 108.78 | *LUZP2* |
| 5 | 57180001 | 57190000 | 141.04 | *LIN7C* |
| 5 | 57370001 | 57380000 | 94.82 | *KIF18A* |
| 5 | 57375001 | 57385000 | 123.14 | *KIF18A* |
| 5 | 57390001 | 57400000 | 214.30 | *KIF18A* |
| 5 | 57395001 | 57405000 | 136.74 | *KIF18A* |
| 5 | 57400001 | 57410000 | 163.44 | *KIF18A* |
| 5 | 57400001 | 57410000 | 163.44 | *METTL15* |
| 5 | 58145001 | 58155000 | 88.65 | *MPPED2* |
| 6 | 925001 | 935000 | 86.94 | *NR4A2* |
| 6 | 2295001 | 2305000 | 88.18 | *MMADHC* |
| 6 | 2300001 | 2310000 | 171.57 | *MMADHC* |
| 6 | 15040001 | 15050000 | 93.63 | *SEMA5B* |
| 6 | 18110001 | 18120000 | 91.27 | *FAP* |
| 6 | 18120001 | 18130000 | 90.97 | *FAP* |
| 6 | 19545001 | 19555000 | 89.70 | *TTC21B* |
| 6 | 20135001 | 20145000 | 107.35 | *Stk39* |
| 6 | 20160001 | 20170000 | 183.75 | *Stk39* |
| 6 | 20170001 | 20180000 | 119.19 | *Stk39* |
| 6 | 20180001 | 20190000 | 160.72 | *Stk39* |
| 6 | 20225001 | 20235000 | 115.27 | *Stk39* |
| 6 | 20230001 | 20240000 | 219.67 | *Stk39* |
| 6 | 20290001 | 20300000 | 208.95 | *CERS6* |
| 6 | 20380001 | 20390000 | 101.75 | *CERS6* |
| 6 | 20405001 | 20415000 | 102.50 | *SPC25* |
| 6 | 20405001 | 20415000 | 102.50 | *G6pc2* |
| 6 | 21720001 | 21730000 | 95.19 | *Pdk1* |
| 6 | 21770001 | 21780000 | 115.29 | *PPP1R9A* |
| 6 | 21990001 | 22000000 | 91.05 | *RAPGEF4* |
| 6 | 22010001 | 22020000 | 104.55 | *RAPGEF4* |
| 6 | 22625001 | 22635000 | 151.99 | *GPR155* |
| 6 | 29505001 | 29515000 | 98.81 | *HDAC4* |
| 7 | 3035001 | 3045000 | 122.43 | *KCNMA1* |
| 7 | 4875001 | 4885000 | 88.15 | *RET* |
| 7 | 4880001 | 4890000 | 107.97 | *RET* |
| 7 | 4885001 | 4895000 | 120.38 | *RET* |
| 7 | 5220001 | 5230000 | 156.20 | *MBL* |
| 7 | 5225001 | 5235000 | 90.97 | *MBL* |
| 7 | 5230001 | 5240000 | 162.84 | *MBL* |
| 7 | 5245001 | 5255000 | 91.78 | *SFTPA1* |
| 7 | 5415001 | 5425000 | 107.36 | *RAP1GDS1* |
| 7 | 9040001 | 9050000 | 102.04 | *ndnf* |
| 7 | 9050001 | 9060000 | 125.99 | *ndnf* |
| 7 | 9095001 | 9105000 | 126.17 | *BICC1* |
| 7 | 9100001 | 9110000 | 194.66 | *BICC1* |
| 7 | 9105001 | 9115000 | 276.95 | *BICC1* |
| 7 | 16215001 | 16225000 | 100.32 | *PAPSS2* |
| 7 | 16220001 | 16230000 | 120.57 | *PAPSS2* |
| 7 | 16245001 | 16255000 | 159.01 | *PAPSS2* |
| 7 | 16245001 | 16255000 | 159.01 | *ATAD1* |
| 7 | 16265001 | 16275000 | 87.47 | *ATAD1* |
| 7 | 16270001 | 16280000 | 86.68 | *PTEN* |
| 7 | 16520001 | 16530000 | 94.25 | *Lipm* |
| 7 | 16520001 | 16530000 | 94.25 | *Lipm* |
| 7 | 16880001 | 16890000 | 101.20 | *SORBS1* |
| 7 | 16905001 | 16915000 | 90.78 | *SORBS1* |
| 7 | 17360001 | 17370000 | 89.67 | *GBF1* |
| 7 | 17375001 | 17385000 | 89.23 | *GBF1* |
| 7 | 17425001 | 17435000 | 124.89 | *GBF1* |
| 7 | 17430001 | 17440000 | 114.25 | *GBF1* |
| 7 | 21920001 | 21930000 | 88.39 | *BLNK* |
| 7 | 23160001 | 23170000 | 98.76 | *CRTAC1* |
| 7 | 23170001 | 23180000 | 135.66 | *CRTAC1* |
| 7 | 23175001 | 23185000 | 92.77 | *CRTAC1* |
| 7 | 23170001 | 23180000 | 135.66 | *GOLGA7B* |
| 7 | 23175001 | 23185000 | 92.77 | *GOLGA7B* |
| 7 | 23175001 | 23185000 | 92.77 | *SFRP5* |
| 7 | 23505001 | 23515000 | 132.13 | *ARMH3* |
| 7 | 28305001 | 28315000 | 85.39 | *Vti1a* |
| 7 | 29950001 | 29960000 | 90.67 | *Atrnl1* |
| 7 | 29970001 | 29980000 | 102.92 | *Atrnl1* |
| 7 | 29975001 | 29985000 | 91.19 | *Atrnl1* |
| 7 | 30005001 | 30015000 | 113.72 | *Atrnl1* |
| 7 | 30010001 | 30020000 | 95.00 | *Atrnl1* |
| 7 | 30915001 | 30925000 | 94.06 | *EMX2* |
| 7 | 32100001 | 32110000 | 103.00 | *INPP5F* |
| 7 | 32100001 | 32110000 | 103.00 | *INPP5F* |
| 8 | 435001 | 445000 | 132.88 | *ZNF644* |
| 8 | 510001 | 520000 | 124.75 | *HFM1* |
| 8 | 710001 | 720000 | 101.67 | *TGFBR3* |
| 8 | 795001 | 805000 | 110.79 | *KIAA1107* |
| 8 | 810001 | 820000 | 85.55 | *KIAA1107* |
| 8 | 815001 | 825000 | 95.13 | *KIAA1107* |
| 8 | 850001 | 860000 | 125.07 | *GLMN* |
| 8 | 865001 | 875000 | 203.51 | *GLMN* |
| 8 | 865001 | 875000 | 203.51 | *RPAP2* |
| 8 | 875001 | 885000 | 143.67 | *RPAP2* |
| 8 | 890001 | 900000 | 146.76 | *RPAP2* |
| 8 | 900001 | 910000 | 102.61 | *RPAP2* |
| 8 | 905001 | 915000 | 94.82 | *RPAP2* |
| 8 | 945001 | 955000 | 124.81 | *EVI5L* |
| 8 | 995001 | 1005000 | 92.18 | *EVI5L* |
| 8 | 1025001 | 1035000 | 160.29 | *EVI5L* |
| 8 | 1025001 | 1035000 | 160.29 | *RPL5* |
| 8 | 1060001 | 1070000 | 124.35 | *DIPK1A* |
| 8 | 1185001 | 1195000 | 109.05 | *Fnbp1l* |
| 8 | 1190001 | 1200000 | 96.80 | *Fnbp1l* |
| 8 | 1195001 | 1205000 | 230.13 | *Fnbp1l* |
| 8 | 1210001 | 1220000 | 108.08 | *Fnbp1l* |
| 8 | 3075001 | 3085000 | 111.01 | *PALMD* |
| 8 | 3260001 | 3270000 | 123.91 | *SLC35A3* |
| 8 | 3265001 | 3275000 | 330.81 | *SLC35A3* |
| 8 | 3260001 | 3270000 | 123.91 | *Mfsd14a* |
| 8 | 3265001 | 3275000 | 330.81 | *Mfsd14a* |
| 8 | 3270001 | 3280000 | 378.76 | *Mfsd14a* |
| 8 | 3275001 | 3285000 | 157.53 | *Mfsd14a* |
| 8 | 3280001 | 3290000 | 120.17 | *Mfsd14a* |
| 8 | 3275001 | 3285000 | 157.53 | *SASS6* |
| 8 | 3280001 | 3290000 | 120.17 | *SASS6* |
| 8 | 3285001 | 3295000 | 344.76 | *SASS6* |
| 8 | 3290001 | 3300000 | 228.27 | *SASS6* |
| 8 | 3290001 | 3300000 | 228.27 | *TRMT13* |
| 8 | 3295001 | 3305000 | 236.37 | *TRMT13* |
| 8 | 3300001 | 3310000 | 212.99 | *TRMT13* |
| 8 | 3295001 | 3305000 | 236.37 | *Lrrc39* |
| 8 | 3300001 | 3310000 | 212.99 | *Lrrc39* |
| 8 | 3305001 | 3315000 | 288.00 | *Lrrc39* |
| 8 | 3305001 | 3315000 | 288.00 | *DBT* |
| 8 | 3310001 | 3320000 | 274.62 | *DBT* |
| 8 | 3315001 | 3325000 | 173.96 | *DBT* |
| 8 | 8940001 | 8950000 | 89.07 | *Mta1* |
| 8 | 8945001 | 8955000 | 156.44 | *Mta1* |
| 8 | 8960001 | 8970000 | 119.69 | *Mta1* |
| 8 | 9000001 | 9010000 | 151.93 | *Mta1* |
| 8 | 12340001 | 12350000 | 102.20 | *COP1* |
| 8 | 12670001 | 12680000 | 155.71 | *Rabgap1* |
| 8 | 12675001 | 12685000 | 165.93 | *Rabgap1* |
| 8 | 13095001 | 13105000 | 90.90 | *Arpc5* |
| 8 | 17970001 | 17980000 | 108.07 | *PRKACB* |
| 8 | 21225001 | 21235000 | 120.34 | *Hyi* |
| 8 | 22925001 | 22935000 | 105.23 | *MAST2* |
| 8 | 25870001 | 25880000 | 99.47 | *FAF1* |
| 8 | 25875001 | 25885000 | 97.39 | *FAF1* |
| 8 | 25985001 | 25995000 | 126.96 | *RNF11* |
| 8 | 26070001 | 26080000 | 112.26 | *EPS15* |
| 8 | 26315001 | 26325000 | 95.40 | *chr31.523* |
| 8 | 26320001 | 26330000 | 273.82 | *chr31.523* |
| 8 | 26330001 | 26340000 | 162.03 | *chr31.523* |
| 8 | 26340001 | 26350000 | 430.06 | *chr31.523* |
| 8 | 26340001 | 26350000 | 430.06 | *ZFYVE9* |
| 8 | 26345001 | 26355000 | 244.25 | *ZFYVE9* |
| 8 | 26340001 | 26350000 | 430.06 | *ZFYVE9* |
| 8 | 26345001 | 26355000 | 244.25 | *ZFYVE9* |
| 8 | 26350001 | 26360000 | 289.16 | *ZFYVE9* |
| 8 | 26360001 | 26370000 | 497.26 | *ZFYVE9* |
| 8 | 26370001 | 26380000 | 197.03 | *ZFYVE9* |
| 8 | 26425001 | 26435000 | 212.82 | *ORC1* |
| 8 | 26430001 | 26440000 | 179.29 | *ORC1* |
| 8 | 26425001 | 26435000 | 212.82 | *PRPF38A* |
| 8 | 26430001 | 26440000 | 179.29 | *PRPF38A* |
| 8 | 26435001 | 26445000 | 102.71 | *PRPF38A* |
| 8 | 26430001 | 26440000 | 179.29 | *TUT4* |
| 8 | 26435001 | 26445000 | 102.71 | *TUT4* |
| 8 | 26435001 | 26445000 | 102.71 | *TUT4* |
| 8 | 26460001 | 26470000 | 122.28 | *TUT4* |
| 8 | 26480001 | 26490000 | 89.47 | *GPX7* |
| 8 | 26490001 | 26500000 | 145.33 | *GPX7* |
| 8 | 26500001 | 26510000 | 155.22 | *SHISAL2A* |
| 8 | 26505001 | 26515000 | 138.17 | *SHISAL2A* |
| 8 | 26510001 | 26520000 | 113.84 | *SHISAL2A* |
| 8 | 26650001 | 26660000 | 85.57 | *SLC1A7* |
| 8 | 26675001 | 26685000 | 128.23 | *SLC1A7* |
| 8 | 30765001 | 30775000 | 115.05 | *DNAJC6* |
| 8 | 32070001 | 32080000 | 157.33 | *NEGR1* |
| 8 | 32505001 | 32515000 | 92.72 | *TNNI3K* |
| 9 | 775001 | 785000 | 85.35 | *MBNL1* |
| 9 | 7120001 | 7130000 | 128.35 | *Tbl1xr1* |
| 9 | 7490001 | 7500000 | 114.64 | *KCNMB2* |
| 9 | 7685001 | 7695000 | 117.50 | *Actl6a* |
| 9 | 7690001 | 7700000 | 119.13 | *Actl6a* |
| 9 | 7755001 | 7765000 | 122.00 | *USP13* |
| 9 | 7760001 | 7770000 | 102.93 | *USP13* |
| 9 | 7760001 | 7770000 | 102.93 | *PEX5L* |
| 9 | 8990001 | 9000000 | 134.02 | *MCF2L* |
| 9 | 9475001 | 9485000 | 172.64 | *Camk2n2* |
| 9 | 9475001 | 9485000 | 172.64 | *EEF1AKMT4* |
| 9 | 9475001 | 9485000 | 172.64 | *chr30.191* |
| 9 | 9475001 | 9485000 | 172.64 | *ALG3* |
| 9 | 9475001 | 9485000 | 172.64 | *Vwa5b2* |
| 9 | 9490001 | 9500000 | 279.24 | *Vwa5b2* |
| 9 | 9490001 | 9500000 | 279.24 | *AN* |
| 9 | 9495001 | 9505000 | 154.48 | *AN* |
| 9 | 9490001 | 9500000 | 279.24 | *chr30.196* |
| 9 | 9495001 | 9505000 | 154.48 | *chr30.196* |
| 9 | 9495001 | 9505000 | 154.48 | *Phf13* |
| 9 | 9565001 | 9575000 | 128.66 | *dvl3* |
| 9 | 10325001 | 10335000 | 154.06 | *PSMD1* |
| 9 | 10550001 | 10560000 | 143.73 | *Muc4* |
| 9 | 10565001 | 10575000 | 114.69 | *TNK2* |
| 9 | 10565001 | 10575000 | 114.69 | *TNK2* |
| 9 | 12500001 | 12510000 | 108.48 | *ZAN* |
| 9 | 15335001 | 15345000 | 88.25 | *Nrros* |
| 9 | 15340001 | 15350000 | 165.76 | *Nrros* |
| 9 | 15340001 | 15350000 | 165.76 | *chr30.390* |
| 9 | 15340001 | 15350000 | 165.76 | *CEP19* |
| 9 | 15340001 | 15350000 | 165.76 | *Pigx* |
| 9 | 15350001 | 15360000 | 125.17 | *Pigx* |
| 9 | 15350001 | 15360000 | 125.17 | *chr30.393* |
| 9 | 15470001 | 15480000 | 141.41 | *Slco2a1* |
| 9 | 15500001 | 15510000 | 89.93 | *Slco2a1* |
| 9 | 15645001 | 15655000 | 85.91 | *Senp2* |
| 9 | 15665001 | 15675000 | 120.54 | *IGF2BP2* |
| 9 | 17065001 | 17075000 | 158.28 | *STAG1* |
| 9 | 17070001 | 17080000 | 191.61 | *STAG1* |
| 9 | 19815001 | 19825000 | 133.26 | *CLSTN2* |
| 9 | 23860001 | 23870000 | 107.64 | *PID1* |
| 9 | 23875001 | 23885000 | 85.54 | *PID1* |
| 10 | 3615001 | 3625000 | 168.01 | *CNTN3* |
| 10 | 3620001 | 3630000 | 123.86 | *CNTN3* |
| 10 | 3630001 | 3640000 | 116.39 | *CNTN3* |
| 10 | 3650001 | 3660000 | 150.77 | *CNTN3* |
| 10 | 3655001 | 3665000 | 193.64 | *CNTN3* |
| 10 | 3660001 | 3670000 | 142.83 | *CNTN3* |
| 10 | 3665001 | 3675000 | 88.52 | *CNTN3* |
| 10 | 3670001 | 3680000 | 166.16 | *CNTN3* |
| 10 | 7725001 | 7735000 | 127.45 | *CADPS* |
| 10 | 8615001 | 8625000 | 167.66 | *FHIT* |
| 10 | 8770001 | 8780000 | 119.90 | *FHIT* |
| 10 | 9425001 | 9435000 | 160.69 | *PRKAR2A* |
| 10 | 9435001 | 9445000 | 94.43 | *PRKAR2A* |
| 10 | 9440001 | 9450000 | 111.13 | *PRKAR2A* |
| 10 | 9450001 | 9460000 | 162.35 | *PRKAR2A* |
| 10 | 9455001 | 9465000 | 105.26 | *PRKAR2A* |
| 10 | 9650001 | 9660000 | 95.64 | *Usp19* |
| 10 | 9650001 | 9660000 | 95.64 | *LAMB2* |
| 10 | 9650001 | 9660000 | 95.64 | *LAMB1* |
| 10 | 22325001 | 22335000 | 190.86 | *CACNA1D* |
| 11 | 4360001 | 4370000 | 97.65 | *ADAM10* |
| 11 | 4375001 | 4385000 | 124.15 | *MINDY2* |
| 11 | 4380001 | 4390000 | 137.78 | *MINDY2* |
| 11 | 4385001 | 4395000 | 152.78 | *MINDY2* |
| 11 | 4415001 | 4425000 | 221.80 | *SLTM* |
| 11 | 4420001 | 4430000 | 327.57 | *SLTM* |
| 11 | 4425001 | 4435000 | 219.11 | *SLTM* |
| 11 | 4430001 | 4440000 | 170.56 | *SLTM* |
| 11 | 10170001 | 10180000 | 118.19 | *ADPGK* |
| 11 | 11295001 | 11305000 | 112.77 | *TRDC* |
| 11 | 11300001 | 11310000 | 223.13 | *TRDC* |
| 11 | 11345001 | 11355000 | 126.47 | *Sppl2a* |
| 11 | 11915001 | 11925000 | 104.29 | *TM6SF1* |
| 11 | 11915001 | 11925000 | 104.29 | *chr27.304* |
| 11 | 11915001 | 11925000 | 104.29 | *HDGFL3* |
| 11 | 13280001 | 13290000 | 116.10 | *ARNT2* |
| 11 | 13870001 | 13880000 | 108.06 | *RLBP1* |
| 11 | 14075001 | 14085000 | 123.39 | *DET1* |
| 11 | 18745001 | 18755000 | 98.02 | *LRRK1* |
| 11 | 18755001 | 18765000 | 90.67 | *LRRK1* |
| 11 | 19615001 | 19625000 | 104.91 | *MEGF11* |
| 11 | 19620001 | 19630000 | 100.21 | *MEGF11* |
| 11 | 19880001 | 19890000 | 88.37 | *zwilch* |
| 11 | 19880001 | 19890000 | 88.37 | *LCTL* |
| 11 | 20795001 | 20805000 | 107.76 | *K19923spermequatorialseg* |
| 12 | 8165001 | 8175000 | 119.69 | *gna0* |
| 12 | 8290001 | 8300000 | 149.17 | *gna0* |
| 12 | 8305001 | 8315000 | 101.87 | *GNAO1* |
| 12 | 8305001 | 8315000 | 101.87 | *Amfr* |
| 12 | 8535001 | 8545000 | 112.90 | *CFDP1* |
| 12 | 10820001 | 10830000 | 147.73 | *GPATCH1* |
| 12 | 11660001 | 11670000 | 93.94 | *TAF4* |
| 12 | 13960001 | 13970000 | 86.58 | *CDH8* |
| 12 | 14060001 | 14070000 | 90.03 | *CDH8* |
| 12 | 17025001 | 17035000 | 106.25 | *PLCG2* |
| 12 | 17030001 | 17040000 | 247.61 | *PLCG2* |
| 12 | 17035001 | 17045000 | 123.34 | *PLCG2* |
| 12 | 17040001 | 17050000 | 141.31 | *PLCG2* |
| 12 | 17045001 | 17055000 | 104.48 | *PLCG2* |
| 12 | 17050001 | 17060000 | 194.99 | *PLCG2* |
| 12 | 17055001 | 17065000 | 109.01 | *PLCG2* |
| 13 | 1295001 | 1305000 | 121.69 | *EFNB1* |
| 13 | 3255001 | 3265000 | 115.97 | *Dach2* |
| 13 | 3260001 | 3270000 | 154.66 | *Dach2* |
| 13 | 7580001 | 7590000 | 118.60 | *HTATSF1* |
| 13 | 8055001 | 8065000 | 159.65 | *GPC3* |
| 13 | 10380001 | 10390000 | 115.01 | *Glra4* |
| 13 | 10660001 | 10670000 | 115.08 | *GABRG4* |
| 13 | 10690001 | 10700000 | 156.61 | *GABRA3* |
| 13 | 10695001 | 10705000 | 104.91 | *GABRA3* |
| 13 | 15010001 | 15020000 | 85.69 | *CLCN5* |
| 13 | 15015001 | 15025000 | 133.87 | *CLCN5* |
| 13 | 16060001 | 16070000 | 189.88 | *KLF5* |
| 13 | 16060001 | 16070000 | 189.88 | *Klf8* |
| 13 | 16995001 | 17005000 | 166.76 | *SLC16A2* |
| 13 | 17000001 | 17010000 | 101.74 | *SLC16A2* |
| 13 | 17005001 | 17015000 | 105.30 | *SLC16A2* |
| 13 | 17000001 | 17010000 | 101.74 | *chr26.411* |
| 13 | 17005001 | 17015000 | 105.30 | *chr26.411* |
| 13 | 17025001 | 17035000 | 111.70 | *rnf12-a* |
| 13 | 17030001 | 17040000 | 143.62 | *rnf12-a* |
| 13 | 17045001 | 17055000 | 129.22 | *NEXMIF* |
| 13 | 17050001 | 17060000 | 139.48 | *NEXMIF* |
| 13 | 17055001 | 17065000 | 87.07 | *NEXMIF* |
| 13 | 17060001 | 17070000 | 113.44 | *NEXMIF* |
| 13 | 17200001 | 17210000 | 105.84 | *ABCB7* |
| 13 | 17255001 | 17265000 | 87.29 | *UPRT* |
| 13 | 17295001 | 17305000 | 118.83 | *zdhhc15b* |
| 13 | 17970001 | 17980000 | 101.05 | *DCX* |
| 13 | 18480001 | 18490000 | 95.58 | *TMEM164* |
| 13 | 18515001 | 18525000 | 207.05 | *TMEM164* |
| 13 | 19140001 | 19150000 | 166.21 | *Smarca1* |
| 13 | 20525001 | 20535000 | 91.85 | *Stag2* |
| 14 | 7825001 | 7835000 | 112.90 | *PCDHAC2* |
| 14 | 13795001 | 13805000 | 107.18 | *CCDC69* |
| 14 | 13805001 | 13815000 | 96.15 | *ANXA6* |
| 14 | 17050001 | 17060000 | 102.23 | *PPP2CA* |
| 14 | 17065001 | 17075000 | 114.51 | *PPP2CA* |
| 14 | 18190001 | 18200000 | 154.45 | *SLC22A4* |
| 14 | 18190001 | 18200000 | 154.45 | *SLC22A5* |
| 14 | 18840001 | 18850000 | 106.56 | *ARHGAP26* |
| 14 | 18840001 | 18850000 | 106.56 | *chr25.484* |
| 15 | 3755001 | 3765000 | 118.58 | *ARPC1A* |
| 15 | 4100001 | 4110000 | 171.40 | *WIPI2* |
| 15 | 4915001 | 4925000 | 139.64 | *BRAT1* |
| 15 | 6080001 | 6090000 | 90.57 | *ZFAND2B* |
| 15 | 10265001 | 10275000 | 106.93 | *SNX29* |
| 15 | 11475001 | 11485000 | 97.28 | *LUC7L* |
| 15 | 12580001 | 12590000 | 139.35 | *RHBDL1* |
| 15 | 12580001 | 12590000 | 139.35 | *STUB1* |
| 15 | 13465001 | 13475000 | 113.04 | *sec14l1* |
| 15 | 13730001 | 13740000 | 94.83 | *Telo2* |
| 15 | 13730001 | 13740000 | 94.83 | *Ift140* |
| 15 | 13735001 | 13745000 | 125.72 | *Ift140* |
| 15 | 13750001 | 13760000 | 92.67 | *Ift140* |
| 15 | 14455001 | 14465000 | 143.65 | *LMTK2* |
| 16 | 1835001 | 1845000 | 93.11 | *BLCAP* |
| 16 | 2145001 | 2155000 | 109.56 | *TOP1* |
| 16 | 2155001 | 2165000 | 139.93 | *TOP1* |
| 16 | 3955001 | 3965000 | 247.88 | *PTPRT* |
| 16 | 3960001 | 3970000 | 230.99 | *PTPRT* |
| 16 | 3965001 | 3975000 | 336.58 | *PTPRT* |
| 16 | 4015001 | 4025000 | 111.77 | *PTPRT* |
| 16 | 4025001 | 4035000 | 89.10 | *PTPRT* |
| 16 | 4035001 | 4045000 | 116.65 | *PTPRT* |
| 16 | 4050001 | 4060000 | 271.66 | *PTPRT* |
| 16 | 14880001 | 14890000 | 104.57 | *Helz2* |
| 16 | 14880001 | 14890000 | 104.57 | *Helz2* |
| 16 | 15530001 | 15540000 | 91.01 | *MAPRE1* |
| 16 | 15530001 | 15540000 | 91.01 | *chr23.437* |
| 16 | 15545001 | 15555000 | 145.98 | *chr23.437* |
| 16 | 15570001 | 15580000 | 240.41 | *BPIFB4* |
| 17 | 3775001 | 3785000 | 166.88 | *PRKAB1* |
| 17 | 3780001 | 3790000 | 136.23 | *PRKAB1* |
| 17 | 3815001 | 3825000 | 113.60 | *CIT* |
| 17 | 3885001 | 3895000 | 96.58 | *Bicdl1* |
| 17 | 4780001 | 4790000 | 104.24 | *Rsph14* |
| 17 | 4785001 | 4795000 | 87.30 | *Rsph14* |
| 17 | 4850001 | 4860000 | 90.94 | *BCR* |
| 17 | 4865001 | 4875000 | 85.45 | *BCR* |
| 17 | 6980001 | 6990000 | 100.05 | *Txnrd2* |
| 17 | 6980001 | 6990000 | 100.05 | *COMT* |
| 17 | 7005001 | 7015000 | 94.41 | *COMT* |
| 17 | 13490001 | 13500000 | 109.82 | *HECTD4* |
| 17 | 13565001 | 13575000 | 192.00 | *RPH3A* |
| 17 | 13925001 | 13935000 | 94.52 | *MYO1H* |
| 17 | 13945001 | 13955000 | 98.53 | *KCTD10* |
| 17 | 13945001 | 13955000 | 98.53 | *ube3b* |
| 17 | 13945001 | 13955000 | 98.53 | *ube3b* |
| 18 | 2685001 | 2695000 | 139.69 | *Cox10* |
| 18 | 2695001 | 2705000 | 194.96 | *Cox10* |
| 18 | 2715001 | 2725000 | 135.66 | *Cox10* |
| 18 | 8015001 | 8025000 | 114.18 | *ANKFN1* |
| 18 | 8795001 | 8805000 | 122.31 | *znf207* |
| 18 | 9020001 | 9030000 | 137.40 | *SMURF2* |
| 18 | 9025001 | 9035000 | 132.83 | *SMURF2* |
| 18 | 9035001 | 9045000 | 101.70 | *SMURF2* |
| 18 | 9040001 | 9050000 | 169.66 | *SMURF2* |
| 18 | 9045001 | 9055000 | 225.54 | *SMURF2* |
| 18 | 9670001 | 9680000 | 100.95 | *APOH* |
| 18 | 9670001 | 9680000 | 100.95 | *Cep112* |
| 18 | 10175001 | 10185000 | 95.27 | *Fam20a* |
| 19 | 1090001 | 1100000 | 144.94 | *ZNF618* |
| 19 | 1905001 | 1915000 | 141.88 | *SSNA1* |
| 19 | 1905001 | 1915000 | 141.88 | *Anapc2* |
| 19 | 1905001 | 1915000 | 141.88 | *ANAPC2* |
| 19 | 1920001 | 1930000 | 104.96 | *tor4a* |
| 19 | 1925001 | 1935000 | 233.39 | *tor4a* |
| 19 | 1920001 | 1930000 | 104.96 | *tor4a-a* |
| 19 | 1925001 | 1935000 | 233.39 | *tor4a-a* |
| 19 | 2045001 | 2055000 | 96.57 | *ENTPD2* |
| 19 | 2045001 | 2055000 | 96.57 | *NELFB* |
| 19 | 8090001 | 8100000 | 90.43 | *BRD3* |
| 20 | 1910001 | 1920000 | 104.12 | *ACAC* |
| 20 | 1915001 | 1925000 | 124.89 | *ACAC* |
| 20 | 4305001 | 4315000 | 149.02 | *Pigl* |
| 20 | 8395001 | 8405000 | 91.67 | *Spns3* |
| 20 | 8845001 | 8855000 | 90.36 | *GTF2IRD1* |
| 20 | 8925001 | 8935000 | 95.19 | *GTF2I* |
| 20 | 8925001 | 8935000 | 95.19 | *GTF2I* |
| 20 | 8935001 | 8945000 | 91.50 | *GTF2I* |
| 20 | 9060001 | 9070000 | 103.89 | *castor2* |
| 21 | 285001 | 295000 | 87.63 | *USP48* |
| 21 | 2505001 | 2515000 | 85.97 | *PDPN* |
| 21 | 2585001 | 2595000 | 269.29 | *AADACL4* |
| 21 | 2595001 | 2605000 | 214.64 | *AADACL4* |
| 21 | 5535001 | 5545000 | 172.12 | *ACAP3* |
| 21 | 7360001 | 7370000 | 99.43 | *MEGF6* |
| 22 | 385001 | 395000 | 157.43 | *chr16.9* |
| 22 | 385001 | 395000 | 157.43 | *chr16.10* |
| 22 | 385001 | 395000 | 157.43 | *LUZP1* |
| 22 | 390001 | 400000 | 87.51 | *LUZP1* |
| 22 | 505001 | 515000 | 223.99 | *Syncrip* |
| 22 | 510001 | 520000 | 182.97 | *Syncrip* |
| 22 | 515001 | 525000 | 122.98 | *Syncrip* |
| 22 | 520001 | 530000 | 164.61 | *Syncrip* |
| 22 | 525001 | 535000 | 107.18 | *Syncrip* |
| 22 | 540001 | 550000 | 133.11 | *Syncrip* |
| 22 | 545001 | 555000 | 85.90 | *Syncrip* |
| 22 | 560001 | 570000 | 187.82 | *Syncrip* |
| 22 | 560001 | 570000 | 187.82 | *atp5if1* |
| 22 | 565001 | 575000 | 141.73 | *atp5if1* |
| 22 | 560001 | 570000 | 187.82 | *Dnajc8* |
| 22 | 565001 | 575000 | 141.73 | *Dnajc8* |
| 22 | 935001 | 945000 | 86.77 | *chr16.27* |
| 22 | 3295001 | 3305000 | 109.61 | *id3-a* |
| 22 | 3295001 | 3305000 | 109.61 | *E2F2* |
| 22 | 4815001 | 4825000 | 106.40 | *MEAF6* |
| 22 | 6980001 | 6990000 | 211.26 | *MACF1* |
| 22 | 6985001 | 6995000 | 270.24 | *MACF1* |
| 23 | 270001 | 280000 | 86.39 | *ARCN1* |
| 23 | 270001 | 280000 | 86.39 | *IFT46* |
| 23 | 270001 | 280000 | 86.39 | *Tmem25* |
| 23 | 270001 | 280000 | 86.39 | *ttc36* |
| 23 | 705001 | 715000 | 99.98 | *CADM1* |
| 23 | 710001 | 720000 | 240.18 | *CADM1* |
| 23 | 2775001 | 2785000 | 114.81 | *NECTIN1* |
| 23 | 5995001 | 6005000 | 121.59 | *Aplp2* |
| 23 | 6540001 | 6550000 | 91.65 | *KCNJ5* |
| 24 | 1425001 | 1435000 | 115.95 | *CAMK1G* |
| 24 | 2135001 | 2145000 | 89.01 | *PIGR* |
| 24 | 2135001 | 2145000 | 89.01 | *chr15.93* |
| 24 | 2550001 | 2560000 | 148.32 | *NUCKS1* |
| 24 | 5945001 | 5955000 | 88.27 | *PRA1* |
| 24 | 5945001 | 5955000 | 88.27 | *MLN* |
| 24 | 6350001 | 6360000 | 109.04 | *CPNE5* |
| 24 | 6350001 | 6360000 | 109.04 | *SORT1* |
| 24 | 6355001 | 6365000 | 115.80 | *SORT1* |
| 24 | 6350001 | 6360000 | 109.04 | *Sort1* |
| 24 | 6355001 | 6365000 | 115.80 | *Sort1* |
| 24 | 6360001 | 6370000 | 143.85 | *Sort1* |
| 24 | 6360001 | 6370000 | 143.85 | *Psma5* |
| 25 | 1200001 | 1210000 | 115.20 | *FBXL20* |
| 25 | 1200001 | 1210000 | 115.20 | *MED1* |
| 25 | 1205001 | 1215000 | 92.07 | *MED1* |
| 25 | 3465001 | 3475000 | 138.74 | *CDC27* |
| 25 | 5250001 | 5260000 | 121.06 | *Fmnl2* |
| 25 | 5250001 | 5260000 | 121.06 | *Fmnl1* |
| 26 | 2745001 | 2755000 | 138.47 | *MAP2K2* |
| 26 | 4100001 | 4110000 | 109.28 | *Pik3r2* |
| 26 | 4105001 | 4115000 | 92.20 | *Pik3r2* |
| 26 | 4110001 | 4120000 | 130.21 | *Pik3r2* |
| 26 | 4110001 | 4120000 | 130.21 | *chr14.234* |
| 26 | 4110001 | 4120000 | 130.21 | *ifi30* |
| 26 | 4240001 | 4250000 | 167.29 | *ELL* |
| 26 | 4245001 | 4255000 | 113.38 | *ELL* |
| 26 | 5635001 | 5645000 | 110.11 | *PTBP1* |
| 26 | 5640001 | 5650000 | 129.69 | *KIAA1958* |
| 27 | 5325001 | 5335000 | 86.28 | *ADAM9* |
| 27 | 5325001 | 5335000 | 86.28 | *ADAM2* |
| 34 | 240001 | 250000 | 175.23 | *ACHE* |
| 34 | 435001 | 445000 | 129.08 | *ALOXE3* |
| 34 | 500001 | 510000 | 159.53 | *MGAM* |
| 34 | 505001 | 515000 | 91.14 | *MGAM* |
| 34 | 865001 | 875000 | 126.62 | *chr5.81* |
| 34 | 865001 | 875000 | 126.62 | *K06727Fcreceptor-likepro* |
| 34 | 875001 | 885000 | 101.50 | *K06727Fcreceptor-likepro* |
| 34 | 880001 | 890000 | 184.18 | *K06727Fcreceptor-likepro* |
| 34 | 885001 | 895000 | 107.19 | *K06727Fcreceptor-likepro* |
| 34 | 990001 | 1000000 | 288.67 | *Polr2a* |
| 34 | 990001 | 1000000 | 288.67 | *POLR2A* |
| 34 | 1120001 | 1130000 | 88.33 | *naa38-a* |
| 34 | 1120001 | 1130000 | 88.33 | *cyb5d1* |
| 34 | 1125001 | 1135000 | 194.26 | *cyb5d1* |
| 34 | 1120001 | 1130000 | 88.33 | *CHD3* |
| 34 | 1125001 | 1135000 | 194.26 | *CHD3* |
| 34 | 1130001 | 1140000 | 161.11 | *CHD3* |
| 34 | 1155001 | 1165000 | 235.73 | *CHD3* |
| 34 | 1165001 | 1175000 | 88.25 | *CHD3* |
| 34 | 1175001 | 1185000 | 99.86 | *CHD3* |
| 34 | 1175001 | 1185000 | 99.86 | *ADPRH* |
| 34 | 1195001 | 1205000 | 89.07 | *KCNAB1* |
| 34 | 1220001 | 1230000 | 119.41 | *Gucy2e* |
| 34 | 1255001 | 1265000 | 123.09 | *PLOD3* |
| 34 | 1255001 | 1265000 | 123.09 | *VAMP2* |
| 34 | 1270001 | 1280000 | 169.69 | *AURKB* |
| 34 | 1270001 | 1280000 | 169.69 | *chr5.109* |

Note: This table identifies regions in the Changle goose genome exhibiting significant XP-CLR scores, which indicate regions under selection compared to five other indigenous goose breeds. The “XP-CLR Score” provides a quantitative measure of cross-population extended haplotype homozygosity, with higher scores suggesting stronger selection. Associated genes are listed alongside their corresponding genomic regions, signifying potential genetic factors contributing to the Changle goose's distinct characteristics.

**Table S8.** **Genomic windows identified by Fst and XP-CLR in Changle goose.**

| Chromosome | Start Position (bp) of window | End Position (bp) of window | XP-CLR Values | Fst Values | Start Position (bp) of Gene | End Position (bp) of Gene | Associated Gene(s) |
| --- | --- | --- | --- | --- | --- | --- | --- |
| 1 | 166240001 | 166250000 | 131.87 | 0.17 | 166214199 | 166259012 | *ZC3H7B* |
| 1 | 167090001 | 167100000 | 85.80 | 0.17 | 167011880 | 167096099 | *Pawr* |
| 1 | 176230001 | 176240000 | 100.88 | 0.23 | 176225822 | 176273750 | *SCAF11* |
| 1 | 176310001 | 176320000 | 90.77 | 0.16 | 176281216 | 176386539 | *ARID2* |
| 1 | 176320001 | 176330000 | 100.88 | 0.21 | 176281216 | 176386539 | *ARID2* |
| 1 | 178710001 | 178720000 | 166.62 | 0.31 | 178686918 | 178724072 | *PNPLA8* |
| 1 | 178720001 | 178730000 | 159.85 | 0.24 | 178686918 | 178724072 | *PNPLA8* |
| 1 | 178720001 | 178730000 | 159.85 | 0.24 | 178723713 | 178780320 | *ARSD* |
| 1 | 178730001 | 178740000 | 159.85 | 0.15 | 178723713 | 178780320 | *ARSD* |
| 1 | 178750001 | 178760000 | 251.65 | 0.17 | 178723713 | 178780320 | *ARSD* |
| 1 | 178760001 | 178770000 | 371.10 | 0.16 | 178723713 | 178780320 | *ARSD* |
| 1 | 204830001 | 204840000 | 649.39 | 0.20 | 204815975 | 204834322 | *GATA3* |
| 1 | 204870001 | 204880000 | 314.06 | 0.19 | 204864994 | 204980567 | *TAF3* |
| 1 | 204910001 | 204920000 | 190.51 | 0.18 | 204864994 | 204980567 | *TAF3* |
| 1 | 204930001 | 204940000 | 129.39 | 0.19 | 204864994 | 204980567 | *TAF3* |
| 1 | 204940001 | 204950000 | 229.53 | 0.22 | 204864994 | 204980567 | *TAF3* |
| 1 | 205690001 | 205700000 | 88.81 | 0.16 | 205696702 | 205711824 | *IL2RA* |
| 1 | 205700001 | 205710000 | 273.12 | 0.17 | 205696702 | 205711824 | *IL2RA* |
| 1 | 205710001 | 205720000 | 212.67 | 0.16 | 205696702 | 205711824 | *IL2RA* |
| 1 | 206950001 | 206960000 | 138.54 | 0.15 | 206933722 | 207082449 | *Chchd3* |
| 1 | 206960001 | 206970000 | 300.09 | 0.21 | 206933722 | 207082449 | *Chchd3* |
| 2 | 29870001 | 29880000 | 157.52 | 0.17 | 29879847 | 29907271 | *FABP4* |
| 2 | 29880001 | 29890000 | 157.52 | 0.16 | 29879847 | 29907271 | *FABP4* |
| 2 | 61550001 | 61560000 | 92.52 | 0.16 | 61539563 | 61565483 | *FBXO15* |
| 2 | 63840001 | 63850000 | 167.21 | 0.16 | 63841937 | 63852016 | *Bloc1s4* |
| 2 | 109670001 | 109680000 | 186.32 | 0.17 | 109424004 | 109713762 | *Cdkal1* |
| 3 | 4090001 | 4100000 | 223.64 | 0.17 | 59301014 | 59326498 | *PKHD1* |
| 3 | 67520001 | 67530000 | 291.68 | 0.23 | 67520544 | 67527433 | *RGS17* |
| 3 | 86360001 | 86370000 | 89.79 | 0.17 | 86360503 | 86397457 | *Fez2* |
| 3 | 86370001 | 86380000 | 119.61 | 0.24 | 86360503 | 86397457 | *Fez2* |
| 4 | 9300001 | 9310000 | 134.31 | 0.22 | 9249221 | 9301738 | *CFAP99* |
| 4 | 9300001 | 9310000 | 134.31 | 0.22 | 9307548 | 9326642 | *Rnf4* |
| 4 | 28120001 | 28130000 | 179.35 | 0.15 | 28104339 | 28159802 | *SCFD2* |
| 5 | 31450001 | 31460000 | 396.47 | 0.19 | 31454038 | 31465179 | *RAD51A* |
| 5 | 31460001 | 31470000 | 683.32 | 0.18 | 31454038 | 31465179 | *RAD51A* |
| 5 | 31460001 | 31470000 | 683.32 | 0.18 | 31454038 | 31465179 | *RAD51A* |
| 5 | 31510001 | 31520000 | 160.90 | 0.15 | 31480297 | 31524634 | *RMDN3* |
| 5 | 34910001 | 34920000 | 105.28 | 0.19 | 34890559 | 34924662 | *Ttbk2* |
| 5 | 34970001 | 34980000 | 126.00 | 0.18 | 35014533 | 35032365 | *UBR1* |
| 5 | 38580001 | 38590000 | 124.89 | 0.17 | 38577216 | 38584698 | *chr34.900* |
| 5 | 52910001 | 52920000 | 404.31 | 0.16 | 52877549 | 52912088 | *CD82* |
| 6 | 20160001 | 20170000 | 183.75 | 0.20 | 20142042 | 20230736 | *Stk39* |
| 6 | 20170001 | 20180000 | 119.19 | 0.17 | 20142042 | 20230736 | *Stk39* |
| 6 | 20230001 | 20240000 | 219.67 | 0.15 | 20142042 | 20230736 | *Stk39* |
| 7 | 4880001 | 4890000 | 107.97 | 0.17 | 4807404 | 4890454 | *RET* |
| 7 | 17370001 | 17380000 | 114.25 | 0.15 | 17357745 | 17461302 | *GBF1* |
| 7 | 17430001 | 17440000 | 114.25 | 0.19 | 17357745 | 17461302 | *GBF1* |
| 8 | 3270001 | 3280000 | 378.76 | 0.22 | 5797917 | 5807159 | *Mfsd14a* |
| 8 | 3280001 | 3290000 | 120.17 | 0.16 | 5797917 | 5807159 | *Mfsd14a* |
| 8 | 3280001 | 3290000 | 120.17 | 0.16 | 3283810 | 3294000 | *SASS6* |
| 8 | 3290001 | 3300000 | 228.27 | 0.29 | 3283810 | 3294000 | *SASS6* |
| 8 | 3290001 | 3300000 | 228.27 | 0.29 | 3295456 | 3304377 | *TRMT13* |
| 8 | 3300001 | 3310000 | 212.99 | 0.16 | 3295456 | 3304377 | *TRMT13* |
| 8 | 3300001 | 3310000 | 212.99 | 0.16 | 3304280 | 3309825 | *Lrrc39* |
| 8 | 3310001 | 3320000 | 274.62 | 0.20 | 3310919 | 3327693 | *DBT* |
| 8 | 3320001 | 3330000 | 173.96 | 0.15 | 3310919 | 3327693 | *DBT* |
| 8 | 3320001 | 3330000 | 173.96 | 0.15 | 3327730 | 3338545 | *RTCA* |
| 8 | 25870001 | 25880000 | 99.47 | 0.19 | 25751397 | 25915849 | *FAF1* |
| 8 | 25980001 | 25990000 | 126.96 | 0.15 | 130207 | 132402 | *RNF11* |
| 8 | 26330001 | 26340000 | 162.03 | 0.26 | 26322101 | 26344836 | *chr31.523* |
| 8 | 26340001 | 26350000 | 430.06 | 0.37 | 26322101 | 26344836 | *chr31.523* |
| 8 | 26340001 | 26350000 | 430.06 | 0.37 | 26348215 | 26379249 | *ZFYVE9* |
| 8 | 26350001 | 26360000 | 289.16 | 0.36 | 26348215 | 26379249 | *ZFYVE9* |
| 8 | 26360001 | 26370000 | 497.26 | 0.38 | 26348215 | 26379249 | *ZFYVE9* |
| 8 | 26370001 | 26380000 | 197.03 | 0.22 | 26348215 | 26379249 | *ZFYVE9* |
| 8 | 26430001 | 26440000 | 179.29 | 0.15 | 26414286 | 26431021 | *ORC1* |
| 8 | 26430001 | 26440000 | 179.29 | 0.15 | 26432187 | 26437814 | *PRPF38A* |
| 8 | 26430001 | 26440000 | 179.29 | 0.15 | 26441872 | 26474177 | *TUT4* |
| 8 | 26680001 | 26690000 | 128.23 | 0.15 | 1778400 | 1793991 | *SLC1A7* |
| 9 | 5650001 | 5660000 | 114.75 | 0.16 | 5655377 | 5674554 | *NCEH1* |
| 9 | 7490001 | 7500000 | 114.64 | 0.15 | 7473028 | 7494946 | *KCNMB2* |
| 9 | 8990001 | 9000000 | 134.02 | 0.15 | 8906904 | 9023804 | *MCF2L* |
| 9 | 10320001 | 10330000 | 154.06 | 0.19 | 10305716 | 10377907 | *PSMD1* |
| 9 | 15330001 | 15340000 | 88.25 | 0.15 | 15338426 | 15342169 | *Nrros* |
| 9 | 15340001 | 15350000 | 165.76 | 0.19 | 15338426 | 15342169 | *Nrros* |
| 9 | 15340001 | 15350000 | 165.76 | 0.19 | 15346628 | 15346927 | *chr30.390* |
| 9 | 15340001 | 15350000 | 165.76 | 0.19 | 15347116 | 15347906 | *CEP19* |
| 9 | 15340001 | 15350000 | 165.76 | 0.19 | 15349918 | 15354942 | *Pigx* |
| 9 | 15470001 | 15480000 | 141.41 | 0.16 | 15472247 | 15502892 | *Slco2a1* |
| 11 | 11300001 | 11310000 | 223.13 | 0.18 | 4535235 | 4544492 | *TRDC* |
| 11 | 19620001 | 19630000 | 100.21 | 0.24 | 19514896 | 19717603 | *MEGF11* |
| 12 | 14060001 | 14070000 | 90.03 | 0.15 | 13933177 | 14064380 | *CDH8* |
| 13 | 20520001 | 20530000 | 91.85 | 0.16 | 20467769 | 20542646 | *Stag2* |
| 14 | 13020001 | 13030000 | 112.56 | 0.16 | 13026173 | 13148483 | *Gria1* |
| 16 | 3950001 | 3960000 | 247.88 | 0.18 | 3834908 | 4178758 | *PTPRT* |
| 16 | 3960001 | 3970000 | 230.99 | 0.15 | 3834908 | 4178758 | *PTPRT* |
| 16 | 4020001 | 4030000 | 111.77 | 0.15 | 3834908 | 4178758 | *PTPRT* |
| 16 | 4040001 | 4050000 | 111.77 | 0.16 | 3834908 | 4178758 | *PTPRT* |
| 16 | 4050001 | 4060000 | 271.66 | 0.15 | 3834908 | 4178758 | *PTPRT* |
| 17 | 3780001 | 3790000 | 136.23 | 0.18 | 3771732 | 3786130 | *PRKAB1* |
| 21 | 2500001 | 2510000 | 85.97 | 0.17 | 2501949 | 2516580 | *PDPN* |
| 21 | 2590001 | 2600000 | 269.29 | 0.15 | 2572705 | 2632882 | *AADACL4* |
| 21 | 2600001 | 2610000 | 214.64 | 0.21 | 2572705 | 2632882 | *AADACL4* |
| 26 | 4240001 | 4250000 | 167.29 | 0.18 | 4216817 | 4270021 | *ELL* |

Note: XP-CLR and Fst values quantify the strength of the selection signal, with higher values indicating more substantial evidence of selection. Associated Gene(s) column lists genes within these windows that may confer advantageous traits to the Changle goose.

**Table S9.** **Frequency distribution of *GATA3* haplotypes in each goose population.**

|  | AAn | ACy | CLG | FCG | MBW | MGG | XGG | ZDW | SUM |
| --- | --- | --- | --- | --- | --- | --- | --- | --- | --- |
| I | 2 | 0 | 0 | 0 | 0 | 0 | 0 | 0 | 2 |
| II | 0 | 2 | 0 | 0 | 0 | 0 | 0 | 0 | 2 |
| III | 0 | 0 | 0 | 2 | 0 | 1 | 0 | 0 | 3 |
| IV | 0 | 0 | 0 | 2 | 0 | 0 | 0 | 0 | 2 |
| V | 0 | 0 | 0 | 0 | 0 | 0 | 2 | 0 | 2 |
| VI | 0 | 0 | 0 | 2 | 0 | 0 | 0 | 0 | 2 |
| VII | 0 | 0 | 1 | 2 | 0 | 0 | 0 | 0 | 3 |
| VIII | 0 | 0 | 0 | 0 | 2 | 0 | 6 | 3 | 11 |
| IX | 0 | 2 | 0 | 0 | 0 | 0 | 0 | 0 | 2 |
| X | 0 | 2 | 0 | 0 | 0 | 0 | 0 | 0 | 2 |
| XI | 0 | 0 | 0 | 0 | 0 | 4 | 0 | 0 | 4 |
| XII | 0 | 0 | 0 | 0 | 0 | 1 | 1 | 1 | 3 |
| XIII | 0 | 0 | 0 | 0 | 0 | 0 | 0 | 2 | 2 |
| XIV | 0 | 0 | 2 | 0 | 0 | 0 | 0 | 0 | 2 |
| XV | 0 | 0 | 63 | 0 | 0 | 0 | 0 | 1 | 64 |
| XVI | 0 | 0 | 2 | 0 | 0 | 0 | 0 | 0 | 2 |
| XVII | 0 | 0 | 4 | 0 | 0 | 0 | 0 | 0 | 4 |
| XVIII | 0 | 0 | 3 | 0 | 0 | 0 | 0 | 0 | 3 |
| XIX | 0 | 0 | 0 | 0 | 0 | 0 | 0 | 3 | 3 |
| XX | 0 | 0 | 0 | 1 | 0 | 1 | 0 | 2 | 4 |
| XXI | 0 | 2 | 0 | 0 | 0 | 0 | 0 | 0 | 2 |
| XXII | 0 | 2 | 0 | 0 | 0 | 0 | 0 | 0 | 2 |
| XXIII | 0 | 0 | 0 | 0 | 2 | 0 | 0 | 0 | 2 |
| XXIV | 0 | 0 | 1 | 3 | 3 | 0 | 0 | 1 | 8 |
| XXV | 0 | 0 | 0 | 0 | 0 | 0 | 3 | 1 | 4 |
| XXVI | 0 | 0 | 0 | 0 | 2 | 0 | 0 | 0 | 2 |
| XXVII | 0 | 0 | 0 | 0 | 0 | 4 | 0 | 0 | 4 |
| XXVIII | 0 | 0 | 0 | 0 | 0 | 4 | 9 | 0 | 13 |
| XXIX | 0 | 0 | 0 | 0 | 0 | 1 | 1 | 0 | 2 |
| XXX | 0 | 0 | 0 | 1 | 1 | 2 | 5 | 0 | 9 |
| XXXI | 0 | 0 | 0 | 0 | 0 | 2 | 0 | 0 | 2 |
| XXXII | 0 | 0 | 0 | 2 | 0 | 0 | 0 | 0 | 2 |
| XXXIII | 0 | 0 | 0 | 0 | 0 | 0 | 2 | 1 | 3 |
| XXXIV | 0 | 0 | 0 | 0 | 0 | 0 | 3 | 3 | 6 |
| XXXV | 0 | 0 | 0 | 5 | 0 | 1 | 8 | 2 | 16 |
| XXXVI | 0 | 0 | 0 | 0 | 0 | 0 | 3 | 0 | 3 |
| XXXVII | 0 | 0 | 1 | 0 | 0 | 0 | 1 | 0 | 2 |
| XXXVIII | 0 | 2 | 0 | 0 | 0 | 0 | 0 | 0 | 2 |
| XXXIX | 0 | 0 | 0 | 1 | 0 | 1 | 2 | 0 | 4 |

Note: The abbreviations of geese refer to Table S1.

**Table S10.** **Frequency distribution of *CD82* haplotypes in each goose population.**

|  | AAn | ACy | CLG | FCG | MGG | XGG | ZDW | SUM |
| --- | --- | --- | --- | --- | --- | --- | --- | --- |
| I | 0 | 0 | 0 | 0 | 0 | 4 | 0 | 4 |
| II | 0 | 0 | 0 | 0 | 1 | 4 | 0 | 5 |
| III | 0 | 0 | 0 | 0 | 0 | 3 | 0 | 3 |
| IV | 0 | 0 | 0 | 0 | 9 | 0 | 0 | 9 |
| V | 0 | 0 | 0 | 0 | 0 | 4 | 0 | 4 |
| VI | 0 | 0 | 0 | 0 | 0 | 2 | 0 | 2 |
| VII | 0 | 0 | 3 | 0 | 0 | 0 | 0 | 3 |
| VIII | 2 | 0 | 0 | 0 | 0 | 0 | 0 | 2 |
| IX | 0 | 0 | 2 | 0 | 0 | 0 | 0 | 2 |
| X | 0 | 0 | 12 | 0 | 0 | 0 | 0 | 12 |
| XI | 0 | 0 | 0 | 2 | 0 | 2 | 0 | 4 |
| XII | 0 | 0 | 0 | 0 | 0 | 2 | 0 | 2 |
| XIII | 0 | 3 | 0 | 0 | 0 | 0 | 0 | 3 |
| XIV | 0 | 0 | 0 | 0 | 0 | 0 | 2 | 2 |
| XV | 0 | 0 | 0 | 0 | 0 | 3 | 0 | 3 |
| XVI | 0 | 0 | 0 | 0 | 0 | 1 | 7 | 8 |
| XVII | 0 | 0 | 0 | 0 | 0 | 3 | 0 | 3 |
| XVIII | 0 | 0 | 0 | 0 | 0 | 3 | 0 | 3 |
| XIX | 0 | 0 | 0 | 0 | 0 | 0 | 3 | 3 |
| XX | 0 | 0 | 14 | 3 | 0 | 9 | 0 | 25 |
| XXI | 0 | 0 | 0 | 0 | 0 | 0 | 2 | 2 |
| XXII | 0 | 0 | 0 | 0 | 0 | 0 | 2 | 2 |
| XXIII | 0 | 0 | 5 | 0 | 0 | 0 | 0 | 5 |

Note: The abbreviations of geese refer to Table S1.

**Table S11.** **Frequency distribution of *Slco2a1* haplotypes in each goose population.**

|  | ACy | CLG | FCG | MBW | MGG | XGG | ZDW | SUM |
| --- | --- | --- | --- | --- | --- | --- | --- | --- |
| I | 0 | 0 | 0 | 0 | 0 | 0 | 2 | 2 |
| II | 0 | 0 | 0 | 0 | 0 | 0 | 2 | 2 |
| III | 0 | 4 | 1 | 0 | 0 | 0 | 0 | 5 |
| IV | 0 | 2 | 0 | 0 | 0 | 0 | 0 | 2 |
| V | 0 | 23 | 0 | 4 | 1 | 7 | 0 | 35 |
| VI | 0 | 2 | 0 | 0 | 0 | 0 | 0 | 2 |
| VII | 0 | 2 | 0 | 0 | 0 | 1 | 0 | 3 |
| VIII | 2 | 0 | 0 | 0 | 0 | 0 | 0 | 2 |
| IX | 0 | 0 | 0 | 0 | 0 | 2 | 2 | 4 |
| X | 0 | 0 | 4 | 0 | 14 | 5 | 0 | 23 |
| XI | 0 | 0 | 0 | 0 | 2 | 0 | 0 | 2 |
| XII | 0 | 0 | 0 | 0 | 3 | 0 | 0 | 3 |
| XIII | 0 | 0 | 3 | 0 | 0 | 0 | 0 | 3 |
| XIV | 0 | 0 | 5 | 0 | 0 | 0 | 0 | 5 |
| XV | 0 | 0 | 2 | 0 | 0 | 3 | 0 | 5 |
| XVI | 0 | 0 | 2 | 0 | 0 | 7 | 0 | 9 |
| XVII | 0 | 1 | 1 | 0 | 0 | 0 | 0 | 2 |
| XVIII | 0 | 0 | 0 | 0 | 4 | 0 | 0 | 4 |
| XIX | 0 | 1 | 0 | 1 | 0 | 0 | 0 | 2 |
| XX | 0 | 2 | 0 | 1 | 0 | 0 | 0 | 3 |
| XXI | 0 | 0 | 0 | 0 | 0 | 2 | 0 | 2 |
| XXII | 0 | 1 | 0 | 0 | 0 | 0 | 1 | 2 |

Note: The abbreviations of geese refer to Table S1.
